# Ovarian activation delays in peripubertal ewe lambs infected with *Haemonchus contortus* can be avoided by supplementing protein in their diets

**DOI:** 10.1186/s12917-021-03020-7

**Published:** 2021-11-03

**Authors:** Paula Suarez-Henriques, Camila de Miranda e Silva Chaves, Ricardo Cardoso-Leite, Danielle G. Gomes-Caldas, Luciana Morita-Katiki, Siu Mui Tsai, Helder Louvandini

**Affiliations:** 1 Department of Animal Science, ESALQ - University of São Paulo, Piracicaba, São Paulo, Brazil; 2 Laboratory of Animal Nutrition, CENA - University of São Paulo, Piracicaba, São Paulo, Brazil; 3 Science, Technology and Education Federal Institute of São Paulo, Piracicaba, São Paulo, Brazil; 4 Cell and Molecular Biology Laboratory, CENA -University of São Paulo, Piracicaba, São Paulo, Brazil; 5 Zootechnics Institute, Nova Odessa, São Paulo, Brazil

**Keywords:** Peripubertal ewe, Gene expression, Ovary, Dietetic protein, Infection, *Haemonchus contortus*

## Abstract

**Background:**

The ewe lamb nutritional and physiological state interfere with the ovarian environment and fertility. The lack or excess of circulating nutrients reaching the ovary can change its gene expression. A protein deficiency in the blood caused by an *Haemonchus contortus* abomasal infection is detrimental to the organism’s development during puberty. The peripubertal period is a time of intensive growth that requires a high level of nutrients. An essential feature controlling pubertal arousal and female reproductive potential is ovarian follicle growth activation. Protein supplementation improves the sheep’s immune response to helminthic infections. We aimed to determine if supplementing protein in infected ewe lambs’ diet would impact the ovarian environment leading to earlier ovarian follicle activation than in infected not supplemented animals.

**Methods:**

We fed 18 Santa Ines ewe lambs (*Ovis aries*) - bred by the same ram - with either 12% protein (Control groups) or 19% protein (Supplemented groups) in their diets. After 35 days of the diet, they were each artificially infected or not with 10,000 *Haemonchus contortus* L3 larvae. Following 77 days of the diet and 42 days of infection, we surgically collected their left ovaries and examined their genes expression through RNA sequencing.

**Results:**

We found that protein supplementation in infected animals led to an up-regulation of genes (FDR *p*-values < 0.05) and biological processes (p-value cut-off = 0.01) linked to meiotic activation in pre-ovulatory follicles and primordial follicle activation, among others. The supplemented not infected animals also up-regulated genes and processes linked to meiosis and others, such as circadian behaviour. The not supplemented animals had these same processes down-regulated while up-regulated processes related to tissue morphogenesis, inflammation and immune response.

**Conclusion:**

Diet’s protein supplementation of peripubertal infected animals allowed them to express genes related to a more mature ovarian follicle stage than their half-sisters that were not supplemented. These results could be modelling potential effects of the interaction between environmental factors, nutrition and infection on reproductive health. When ovarian activation is achieved in a timely fashion, the ewe may generate more lambs during its reproductive life, increasing sheep breeders’ productivity.

**Supplementary Information:**

The online version contains supplementary material available at 10.1186/s12917-021-03020-7.

## Background

Female reproductive capacity is maintained by the gradual and regular activation of the ovarian follicles population. Ovarian follicle development in ruminants is influenced by nutrition through changes in metabolic hormones and nutrients direct effects on the ovary [1–3]. Primordial follicle activation is independent of pituitary hormones and is influenced by the ovarian tissue [4]. Changes in gene expression enable the communication between ovarian cells and their environment. An example is the gene MTORC1, which senses the ovarian environment’s nutritional and physiological states; its signalling promotes either cell growth or autophagy [5]. The induction of MTORC1 activity in oocytes was associated with primordial follicle activation [4]. Knowing how the environment affects whole-genome expression, we may manipulate it or specific gene pathways of interest if necessary.

A high level of nutrients during pubertal transition is necessary as it involves a series of morphological, physiological and behavioural changes. During puberty, a surge in the luteinising hormone activates meiosis in preovulatory follicles [6]. Environmental changes might interfere with meiotic resumption and genetic quality of the oocyte, affecting reproductive ability [7]. The sheep’s abomasal infection with the widespread helminth *Haemonchus contortus* causes severe blood losses, anaemia, blood coagulation issues, impaired nutrient utilisation and intense antibody production [8]. These symptoms affect productivity and survival and are a product of defences’ performance. In humans, a potential costs of immune activation is the suppression of reproductive function [9]. One of the consequences of a nutritional deficit in ewe lambs is the delayed first ovulation [10]. Environmental resources and risks determine developmental and reproductive strategies. Early in development, the balance of investment in innate versus acquired immunity is optimised in response to local ecological conditions. An abundance of nutrients, high pathogen exposure, and low signals of death likelihood at sensitive periods of immune development, should favour higher levels of investment in acquired immunity and still allow for timely reproductive success [11].

Protein’s supplementation to growing sheep during infection resulted in improved immunity against gastrointestinal nematodes [12]. By providing “nutritional therapy” for the animal to balance its homeostasis and combat the parasite, anthelminthic therapy may be avoided or diminished. *H contortus* develops resistance to anthelmintic drugs shortly after being exposed to them [13, 14]. Besides, anthelminthic therapy increases sheep production costs and may leave residuals in sheep’s milk, meat and waste [15–17]. As residues’ presence in animal products is becoming a significant complaint in public health and environment, more sustainable management to fight helminthic infections should be sought.

So, if a higher protein intake is supplied to fight the organic imbalance and build an immune response against the parasite, it could also benefit ovarian activation in pubertal ewe lambs. The ovulation rate increased in mature ewes fed with high protein or energy [18]. Also, an intermediate level of protein supplementation in adult ewes improved reproductive response [19]. However, there is an explicit lack of studies in peripubertal ewe lambs infected with *Haemonchus contortus* - a worldwide spread situation in sheep breeding generating substantial economic losses. We hypothesised that supplementing protein to peripubertal ewe lambs would benefit the ovarian environment, leading to ovarian follicles reaching meiotic activation earlier than infected not supplemented animals, despite the infection’s detrimental effects.

## Results

### Haematological and biochemical parameters

Plasma protein(*p* = 0.02) and infection(*p* = 0.000) influenced haemoglobin levels as found in the covariance analysis for haemoglobin as the dependent variable, diet and infection as categorical factors and plasma protein as a continuous predictor.

There was significant variation in plasma protein on the fourth date between Control and Supplemented protein diet groups on factorial ANOVA; supplemented groups presented higher levels (*p* = 0.021). Levels of plasma albumin did not vary significantly according to time, diet or infection (repeated measures ANOVA, factorial ANOVA). Glucose plasma concentration varied from 40.3 to 73.3 mg/dL on three collection dates but did not vary significantly with diet, infection status or overtime (factorial ANOVA, repeated measures). Glucose concentration remained inside the typical values for the species [20]. There was significant variation for plasma urea levels between different diets, infection status and interaction diet vs infection on the fourth date; its level was higher in the supplemented groups (factorial ANOVA; respectively *p* = 0.000, *p* = 0.020, *p* = 0.026).

Haemoglobin levels varied significantly overtime after the infection (ANOVA repeated measures *p* = 0.05) between infected and not infected animals. The not infected animals presented higher levels of haemoglobin on the three post-infection dates. Haemoglobin also varied significantly on the fourth date when infection status was considered, being higher in the supplemented group (factorial ANOVA *p* = 0.005).

Red blood cells’ numbers varied significantly according to infection status on the third and fourth dates; supplemented groups presented higher numbers (*p* = 0.032 and *p* = 0.00026). In the white blood cells count, the number of monocytes varied significantly with the interaction diet vs infection on the second date, being higher in the control infected group (*p* = 0.036 factorial ANOVA, repeated measures ANOVA). The neutrophils’ number varied according to the diet on the fourth date, and it was significantly higher in the supplemented protein groups (factorial ANOVA *p* = 0.030). Lymphocytes number varied significantly with diet on the fourth date; it was higher in the control protein groups (factorial ANOVA *p* = 0.044). Despite the variation in white blood cells numbers, the counts were within the physiological range.

The levels of beta-hydroxybutyrate in whole blood ranged from 0.2 to 0.7 mmol/L. It did not vary significantly with diet or infection status, nor did it vary over time significantly (factorial ANOVA, repeated measures ANOVA) during the experiment. Its level remained inside the expected values for the species [21].

### Number of *Haemonchus contortus* eggs in the faeces

**Fig. 1:**
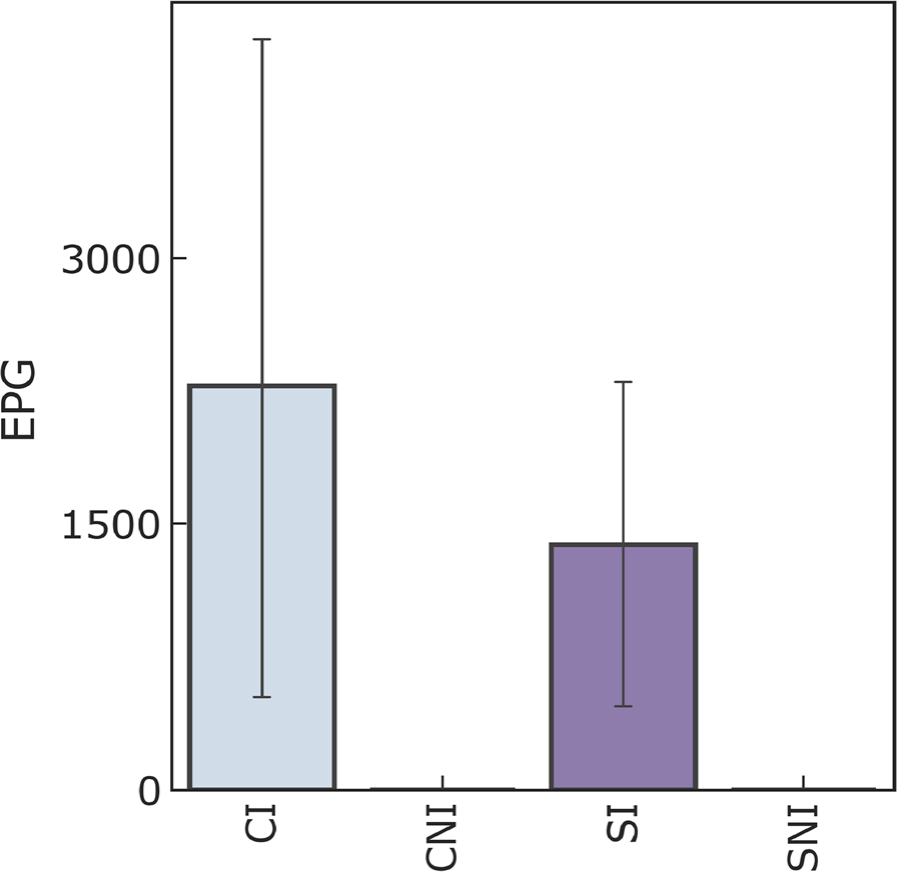
Average number of eggs per gram of faeces in the groups CI (control infected), CNI (control not infected), SI (supplemented infected), and SNI (supplemented not infected). The vertical lines show the individuals variation

The not infected group remained free of *H. contortus* eggs in their faeces during the experiment. The number of eggs in the supplemented infected group varied from 300 to 2800 (average 1380). It varied from 50 to 5400 (average 2190) in the control infected group; this data is represented in Fig. 1. The eggs number in their faeces did not vary significantly between protein supplemented and not supplemented infected groups nor over time (factorial ANOVA, repeated measures ANOVA).

### RNA sequencing’s raw data filtering and sequencing data summary

The filtering removed the reads’ adaptors (3.99%), the reads in which more than 10% of its bases could not be identified, and removed the reads containing low-quality bases. There were no reads removed because of low quality. The clean reads remaining after filtering were 96,01% of the total. The data quality summary is shown in (Additional file 1).

The total generated data was 153.3 G raw data and 148.1 G of filtered data-clean reads. The analysis identified 28.401 expressed genes. The number of sequences generated and sequencing depth in each sample are shown in Additional file 2. The percentage of mapping to gene regions can be seen in Additional file 3.

### Differentially expressed genes between supplemented infected vs control infected groups

**Fig. 2:**
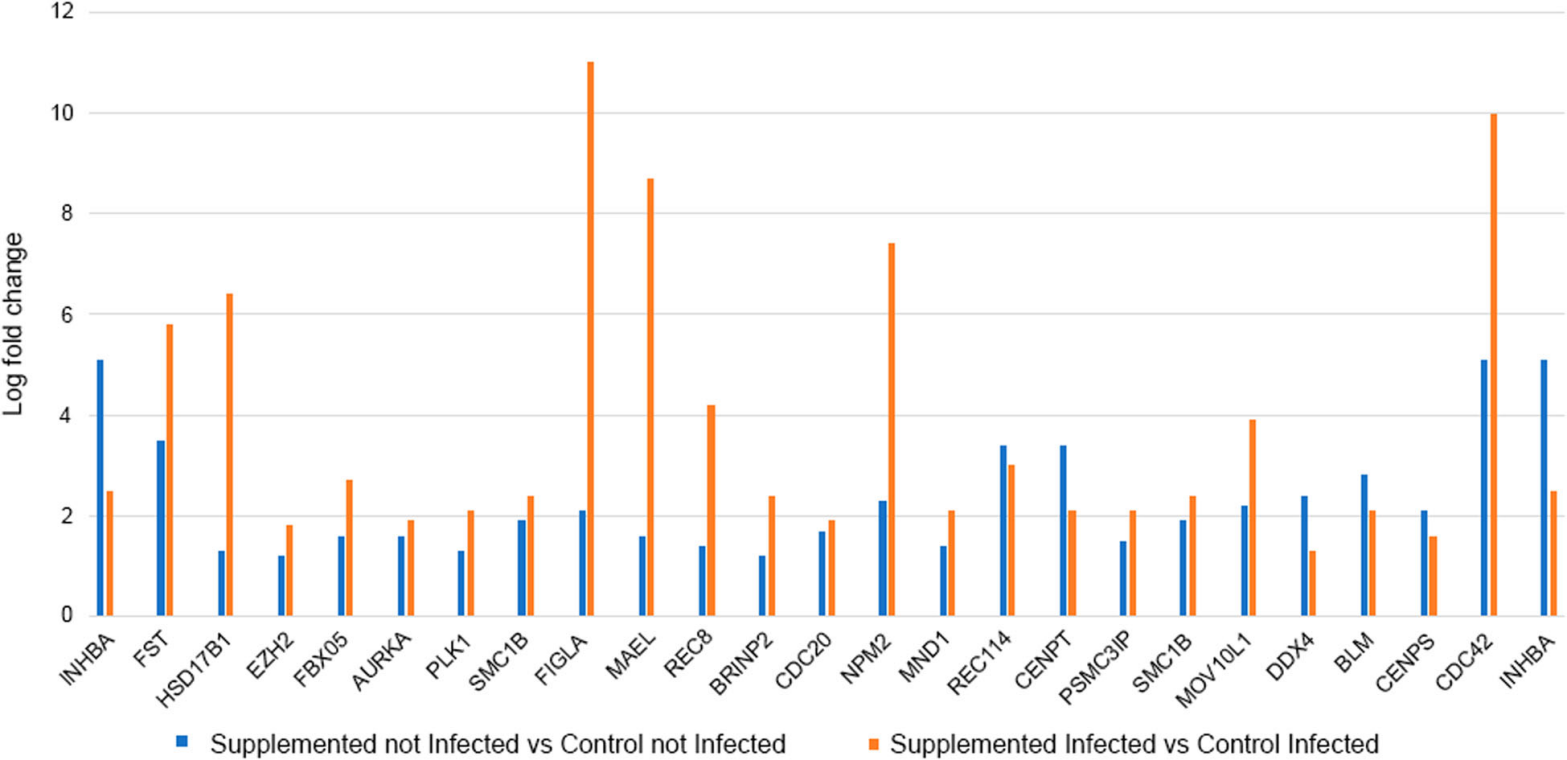
Up-regulation expression level (Log fold change) of genes in common between the comparisons Supplemented not Infected vs Control not Infected and Supplemented Infected vs Control Infected

We analysed the gene expression difference between supplemented infected vs control infected to understand which genes responded to the interaction diet vs infection. This comparison produced a list of 2879 differentially expressed up-regulated genes and 3001 down-regulated genes (log fold change > 1.1 and < − 1.1; FDR *p*-value < 0.05). Some of the up-regulated genes found in the comparison between Supplemented Infected vs Control Infected are shown in Table 1 (found at the end of the text). The complete list of these genes is in Additional file 5. These genes are also pictured in a plot (Fig. 2) comparing the up-regulation level of common genes to the ones in comparison Supplemented not Infected vs Control not Infected.

**Table 1:** Up-regulated genes in the comparison Supplemented Infected vs Control Infected

Gene ID	Gene name /Function	GO/Biological processes/Pathways	Log fold change
INHBA	Inhibin subunit beta A	Gamete generation (GO:0007276); Developmental process involved in reproduction (GO:0003006); First menstruation in humans	5.1
FST	Follistatin	Gamete generation (GO:0007276); Developmental process involved in reproduction (GO:0003006); Body height (Dbgap – Ncbi).	3.5
HSD17B1	Hydroxysteroid 17-beta dehydrogenase	Estrogen biosynthesis process (GO:0006703); Estrogen metabolic process (GO:0008210); Cell hormonal metabolism process (GO:0034754)	1.3
EZH2	Enhancer of zeste 2 polycomb repressive complex 2 subunit	Repression of the transcription (GO:0098532); G2M checkpoint (Hallmark)	1.2
FBXO5	F-BOX protein 5	Spindle assembly involved in female meiosis (GO:0007057); Negative regulation of DNA endoreduplication (GO:0032876)	1.6
AURKA	Aurora kinase	Oocyte meiosis (hsa04114); Positive regulation of oocyte maturation (GO:1900195); Spindle assembly involved in female meiosis I (GO:0007057)	1.6
PLK1	Polo like kinase 1	Oocyte meiosis (hsa04114); Progesterone-mediated oocyte maturation (hsa04914); Mitotic spindle assembly checkpoint GO:0007094)	1.3
SMC1B	Structural maintenance of chromosomes 1B	Oocyte meiosis (hsa04114) Sister chromatid segregation (GO:000081) Nuclear chromosome segregation (GO:0098813)	1.9
FIGLA	Folliculogenesis specific basic helix-loop-helix transcription factor	Oocyte development (GO:0048599); Oocyte differentiation (GO:0009994); Oogenesis (GO:0048477)	2.1
MAEL	Maelstrom spermatogenic transposon silencer	DNA methylation involved in gamete generation (GO:0043046) Synapsis (GO:0007129)	1.6
REC8	REC8 meiotic recombination protein	Oocyte maturation (GO:0001556); Synaptonemal complex assembly (GO:0007130); Seminiferous tubule development (GO:0072520)	1.4
BRINP2	BMP/retinoic acid inducible neural specific 2	Cellular response to retinoic acid (GO:0071300); Response to retinoic acid (GO:0032526)	1.2
CDC20	Cell division cycle 20	Oocyte meiosis (hsa04114); Mitotic spindle assembly checkpoint (GO:0007094)	1.7
NPM2	Nucleophosmin/nucleoplasmin 2	Positive regulation of meiotic nuclear division (GO:0045836); Positive regulation of meiotic cell cycle (GO:0051446)	2.3
MND1	Meiotic nuclear divisions 1	Reciprocal meiotic recombination (GO:0007131) Meiosis I (GO:0007127); Homologous recombination (GO:0035825)	1.4
REC114	REC114 meiotic recombination	DNA recombination (GO:0006310); Meiotic cell cycle (GO:0051321)	3.4
CENPT	Centromere protein T	Kinetochore assembly (GO:0051382); Cenp-A containing nucleosome assembly (GO:0034080)	3.4
CDC16	Cell division cycle 16	Progesterone-mediated oocyte maturation (hsa04914); Oocyte meiosis (hsa04114)	1.2
CDC25C	Cell division cycle 25C	Positive regulation of G2/Mi transition of meiotic cell cycle (GO:0110032); Oocyte meiosis (hsa04114); Progesterone-mediated oocyte maturation((hsa04914)	1.3
NCAPH2	Non-SMC condensin II complex subunit	Female meiosis chromosome separation (GO:0051309); Female meiosis chromosome segregation (GO:0016321); Meiotic chromosome condensation (GO:0010032).	1.4
PSMC3IP	PSMC3 interacting protein	Reciprocal meiotic recombination (GO:0007131); Meiosis I (GO:0007127); Homologous recombination (GO:0035825)	2.1
TRIP13	Thyroid hormone receptor interactor 13	Meiotic recombination checkpoint (GO:0051598)	1.9
SMCB1	Structural maintenance of chromosomes 1B	Oocyte meiosis (hsa04114); Sister chromatid segregation (GO:0000819)	2.4
MOV10L1	Mov10 like RISC complex RNA helicase 1	DNA methylation involved in gamete generation (GO:0043046) Male meiosis I (GO:0007141)	3.9
DDX4	Dead-box helicase	DNA methylation involved in gamete generation (GO:0043046); Hallmark spermatogenesis(M5951); Male meiosis I (GO:0007141)	1.3
BLM	BLM RecQ like helicase	Homologous recombination (hsa03440)	2.1
CENPS	Centromere protein S	Resolution of meiotic recombination intermediates (GO:0000712); DNA replication-independent nucleosome assembly (GO:0006336)	1.6
XRCC3	X-ray repair cross complementing	Homologous recombination (Hsa03440); Hallmark estrogen response late(M5907)	2.1
ANKRD31	Ankyrin repeat domain 31	Meiotic DNA double-strand break formation involved in reciprocal meiotic recombination (GO:0010780) Positive regulation of meiotic DNA double-strand break formation (GO:1903343); Body weights and measures (Dbgap-Ncbi)	1.7
FANCD2	FA complementation group D2	Double-strand break repair involved in meiotic recombination (GO:1990918)	1.8
CDC42	Coiled-coil domain containing 42	GO:0048515 Spermatid differentiation	10
BUB1	BUB1 mitotic checkpoint serine/threonine kinase	Meiotic sister chromatid cohesion, centromeric (GO:0051754); Oocyte meiosis (Hsa04114); Progesterone-mediated oocyte maturation (hsa04914); Hallmark spermatogenesis(M5951); Hallmark MTORC1 signaling(M5924)	1.8
NUCB2	Nucleobinding 2	Negative regulation of appetite (GO:0032099); Negative regulation of response to food (GO:0032096); Negative regulation of response to nutrient level (GO:0032108)	1.4
CLPSL2	Colipase like 2	Response to food (GO:0032094); Digestion (GO:0007586); Lipid catabolic process (GO:0016042)	1.3
CYP19A1	Cytochrome P450 family 19 subfamily A member 1	Positive regulation of estradiol secretion (GO:2000866); Negative regulation of macrophage chemotaxis (GO:0010760)	3.6
VEGF-A	Vascular endothelial growth factor A	VEGF signaling pathway (hsa04370)	2.2
BRIP1	BRCA1 interacting protein C-terminal helicase 1	Meiotic DNA double strand break process involved in reciprocal meiotic recombination (GO:0010705); Chiasma assembly (GO:0051026); Spermatogonial cell division (GO:0007284); Homologous recombination (Hsa03440)	2
RBBP8	RB binding protein 8	DNA double-strand break processing involved in repair via single-strand annealing (GO:0010792); Homologous recombination (Hsa03440)	2
GAST	Gastrin	Response to food (GO:0032094); Response to nutrient levels (GO:0031667) Response to extracellular stimulus (GO:0009991) Gastric acid secretion (hsa04971)	4.6
LRCOL1	Leucine rich colipase like	Response to food (GO:0032094); Digestion (GO:0007586); Lipid catabolic process (GO:0016042)	10

### Differentially expressed genes between supplemented not infected vs control not infected groups

We compared the gene expression difference between the not infected groups to ascertain the changes happening in the ovary’s gene expression due to protein supplementation in the diet. The comparison between groups Supplemented Not Infected vs Control Not Infected generated a list of 2904 differentially expressed up-regulated genes and 2474 down-regulated genes (log fold change > 1.1 and < − 1.1; FDR *p*-value < 0.05). Some of the up-regulated genes found in the comparisons Supplemented Not Infected vs Control Not Infected are shown in Table 2 (found at the end of the text). The full list of these genes is in Additional file 4.

**Table 2:** Up-regulated genes in the comparisons Supplemented not Infected vs Control not Infected

Gene ID	Gene name /Function	GO/Biological processes/Pathways	Log fold change
INHBA	Inhibin subunit beta A	Gamete generation (GO:0007276); Developmental process involved in reproduction (GO:0003006); First menstruation in humans	2.5
FST	Follistatin	Gamete generation (GO:0007276); Developmental process involved in reproduction (GO:0003006); Body height (Dbgap – Ncbi).	5.8
HSD17B1	hydroxysteroid 17-beta dehydrogenase	Estrogen biosynthesis process (GO:0006703); Estrogen metabolic process (GO:0008210); Cell hormonal metabolism process (GO:0034754)	6.4
EZH2	enhancer of zeste 2 polycomb repressive complex 2 subunit	Repression of the transcription (GO:0098532); G2M checkpoint (Hallmark)	1.8
FBXO5	F-BOX protein 5	Spindle assembly involved in female meiosis (GO:0007057); Negative regulation of DNA endoreduplication (GO:0032876)	2.7
AURKA	Aurora kinase	Oocyte meiosis (hsa04114); Positive regulation of oocyte maturation (GO:1900195); Spindle assembly involved in female meiosis I (GO:0007057)	1.9
PLK1	Polo like kinase 1	Oocyte meiosis (hsa04114); Progesterone-mediated oocyte maturation (hsa04914); Mitotic spindle assembly checkpoint GO:0007094)	2.1
SMC1B	Structural maintenance of chromosomes 1B	Oocyte meiosis (hsa04114) Sister chromatid segregation (GO:000081) Nuclear chromosome segregation (GO:0098813)	2.4
FIGLA	Folliculogenesis specific basic helix-loop-helix transcription factor	Oocyte development (GO:0048599); Oocyte differentiation (GO:0009994); Oogenesis (GO:0048477)	11
NOBOX	Oogenesis homeobox	Female gamete generation (GO:0048477); Germ cell development (GO:0007292);	11
MAEL	Maelstrom spermatogenic transposon silencer	DNA methylation involved in gamete generation (GO:0043046) Synapsis (GO:0007129)	8.7
REC8	REC8 meiotic recombination protein	Oocyte maturation (GO:0001556); Synaptonemal complex assembly (GO:0007130); Seminiferous tubule development (GO:0072520)	4.2
BRINP2	BMP/retinoic acid inducible neural specific 2	Cellular response to retinoic acid (GO:0071300); Response to retinoic acid (GO:0032526)	2.4
CDC20	Cell division cycle 20	Oocyte meiosis (hsa04114); Mitotic spindle assembly checkpoint (GO:0007094)	1.9
NPM2	Nucleophosmin/nucleoplasmin 2	Positive regulation of meiotic nuclear division (GO:0045836); Positive regulation of meiotic cell cycle (GO:0051446)	7.4
MND1	Meiotic nuclear divisions 1	Reciprocal meiotic recombination (GO:0007131) Meiosis I (GO:0007127); Homologous recombination (GO:0035825)	2.1
REC114	REC114 meiotic recombination	DNA recombination (GO:0006310); Meiotic cell cycle (GO:0051321)	3
CENPT	Centromere protein T	Kinetochore assembly (GO:0051382); Cenp-A containing nucleosome assembly (GO:0034080)	2.1
NCAPH2	Non-SMC condensin II complex subunit	Female meiosis chromosome separation (GO:0051309); Female meiosis chromosome segregation (GO:0016321); Meiotic chromosome condensation (GO:0010032).	1.5
PSMC3IP	PSMC3 interacting protein	Reciprocal meiotic recombination (GO:0007131); Meiosis I (GO:0007127); Homologous recombination (GO:0035825)	2.1
TRIP13	Thyroid hormone receptor interactor 13	Meiotic recombination checkpoint (GO:0051598)	1.9
SMC1B	Structural maintenance of chromosomes 1B	Oocyte meiosis (hsa04114); Sister chromatid segregation (GO:0000819)	2.4
MOV10L1	Mov10 like RISC complex RNA helicase 1	DNA methylation involved in gamete generation (GO:0043046) Male meiosis I (GO:0007141)	3.9
DDX4	Dead-box helicase	DNA methylation involved in gamete generation (GO:0043046); Hallmark spermatogenesis(M5951); Male meiosis I (GO:0007141)	1.3
BLM	BLM RecQ like helicase	Homologous recombination (hsa03440)	2.1
CENPS	Centromere protein S	Resolution of meiotic recombination intermediates (GO:0000712); DNA replication-independent nucleosome assembly (GO:0006336)	1.6
XRCC3	X-ray repair cross complementing	Homologous recombination (Hsa03440); Hallmark estrogen response late(M5907)	2.1
FANCD2	FA complementation group D2	Double-strand break repair involved in meiotic recombination (GO:1990918)	1.8
CDC42	Coiled-coil domain containing 42	GO:0048515 Spermatid differentiation	10
BUB1	BUB1 mitotic checkpoint serine/threonine kinase	Meiotic sister chromatid cohesion, centromeric (GO:0051754); Oocyte meiosis (Hsa04114); Progesterone-mediated oocyte maturation (hsa04914); Hallmark spermatogenesis(M5951); Hallmark MTORC1 signaling(M5924)	1.8
LHX8	LIM homeobox 8	Female gonad development (GO:0008585)	10
NOBOX	NOBOX oogenesis homeobox	Female gamete generation (GO:0007292); Germ cell development (GO:0007281)	11
SOHLH1	spermatogenesis and oogenesis specific basic helix-loop-helix 1	Oocyte differentiation (GO:0009994); Oogenesis (GO:0048477); Spermatogenesis (GO:0007283)	8.5
Foxl2	forkhead box L2	Positive regulation of gonadotropin secretion (GO:0032278)	4.8
FSHR	Receptor for follicle stimulating hormone	Cellular response to follicle-stimulating hormone stimulus (GO:0071372); Ovarian follicle development (GO:0001541)	2.9
PGR	Progesterone receptor	Progesterone receptor signaling pathway (GO:0050847); Progesterone-mediated oocyte maturation (hsa04914); Oocyte meiosis (hsa04114)	1.1
STAR	Steroidogenic acute regulatory protein	Circadian sleep/wake cycle, REM sleep (GO:0042747); Ovarian steroidogenesis (hsa04913)	2.2
CYP17A1	Cytochrome P450 family 17 subfamily A member 1	Progesterone metabolic process (GO:0042448)	4.1
CYP11A1	Cytochrome P450 family 11 subfamily A member 1	GO:0006700 C21-steroid hormone biosynthetic process; Ovarian steroidogenesis (hsa04913)	2.4
CYP19A1	Cytochrome P450 family 19 subfamily A member 1	Positive regulation of estradiol secretion (GO:2000866); Negative regulation of macrophage chemotaxis (GO:0010760)	9.5
ESR2	Estrogen receptor 2	Intracellular estrogen receptor signaling pathway (GO:0030520)	2.7
INHBA	Inhibin subunit beta A	Negative regulation of follicle-stimulating hormone secretion (GO:0046882); Positive regulation of follicle-stimulating hormone secretion (GO:0046881)	2.5
LHCGR	Luteinizing hormone/choriogonadotropin receptor	Cellular response to luteinizing hormone stimulus (GO:0071373)	1.3
VEGF-A	Vascular endothelial growth factor A	VEGF signaling pathway (hsa04370)	2.5

**Fig. 3:**
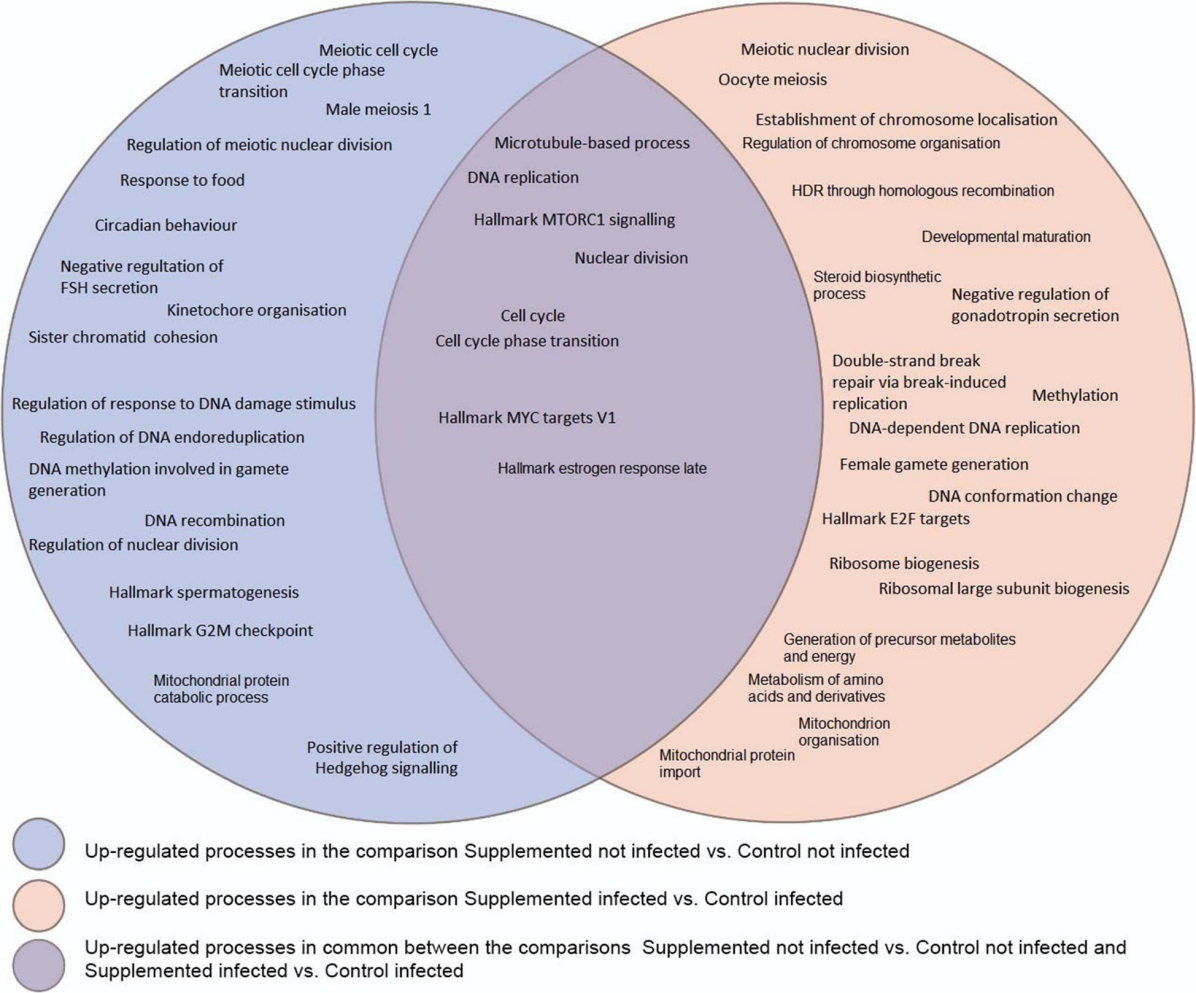
Common and distinct up-regulated processes between Supplemented not Infected vs Control not Infected and Supplemented Infected vs Control Infected. *The processes that were found enriched in common in the two comparisons are represented in the intersection of the two circles

**Fig. 4:**
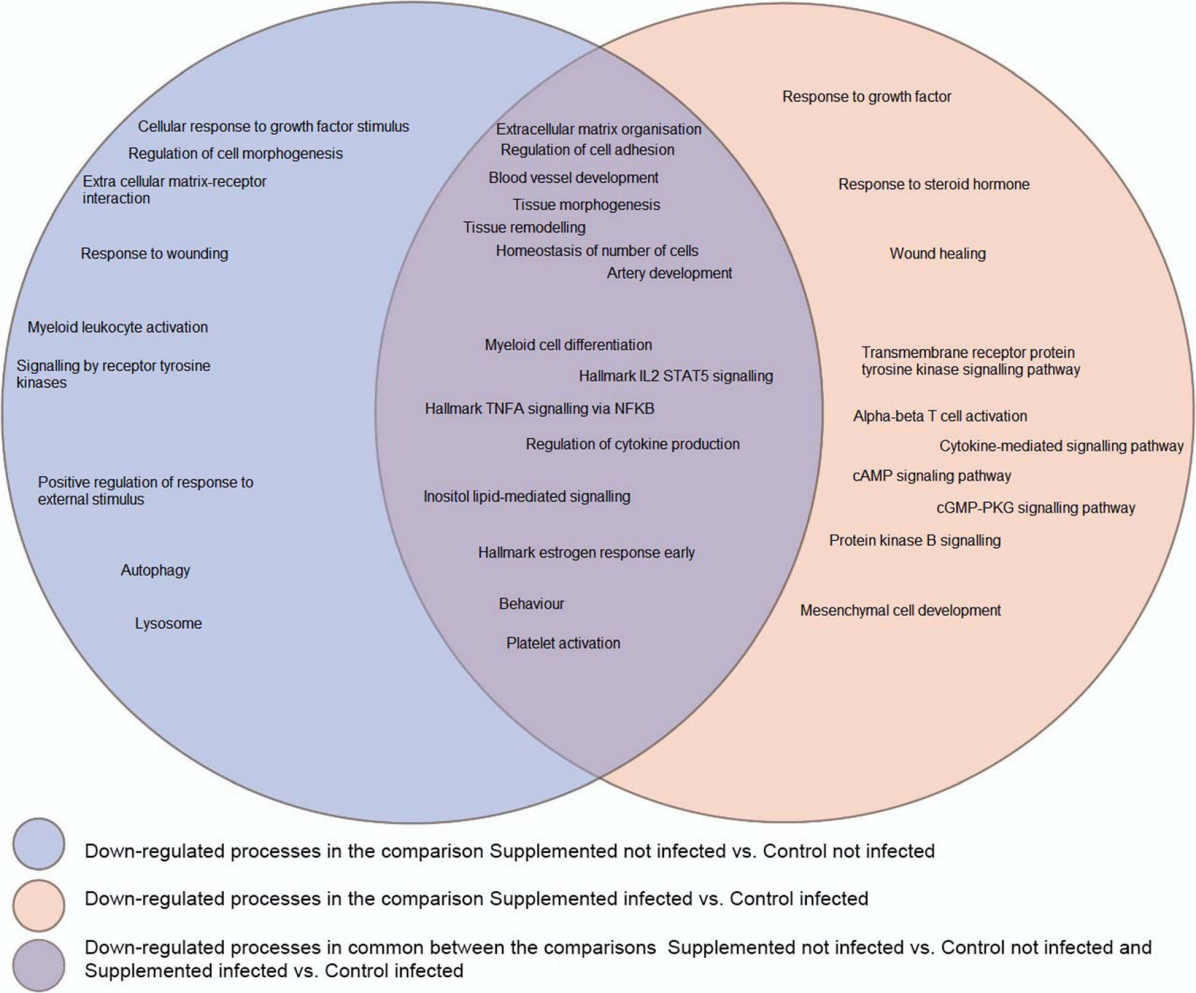
Common and distinct down regulated processes between Supplemented not Infected vs Control not Infected and Supplemented Infected vs Control Infected. *The processes that were found enriched in common in the two comparisons are represented in the intersection of the two circles

In Fig. 3, we pictured common and uniquely processes found enriched in the up-regulated gene lists of the comparisons between Supplemented not Infected vs Control not Infected and Supplemented Infected vs Control Infected. The common and unique processes found enriched in the down-regulated gene lists of the comparisons between Supplemented not Infected vs Control not Infected and Supplemented Infected vs Control Infected is depicted in Fig. 4.

### Enriched biological processes found in the differentially expressed genes

#### Comparison between the supplemented not infected vs control not infected groups

We analysed the full list of genes differentially expressed in the comparison between Supplemented Not Infected vs Control Not Infected to find the differentially enriched biological processes in the ewe lambs ovary due to protein’s level in the diet. The Metascape software identified 1927 identifiers, 1927 human Entrez Gene IDs.

**Fig. 5:**
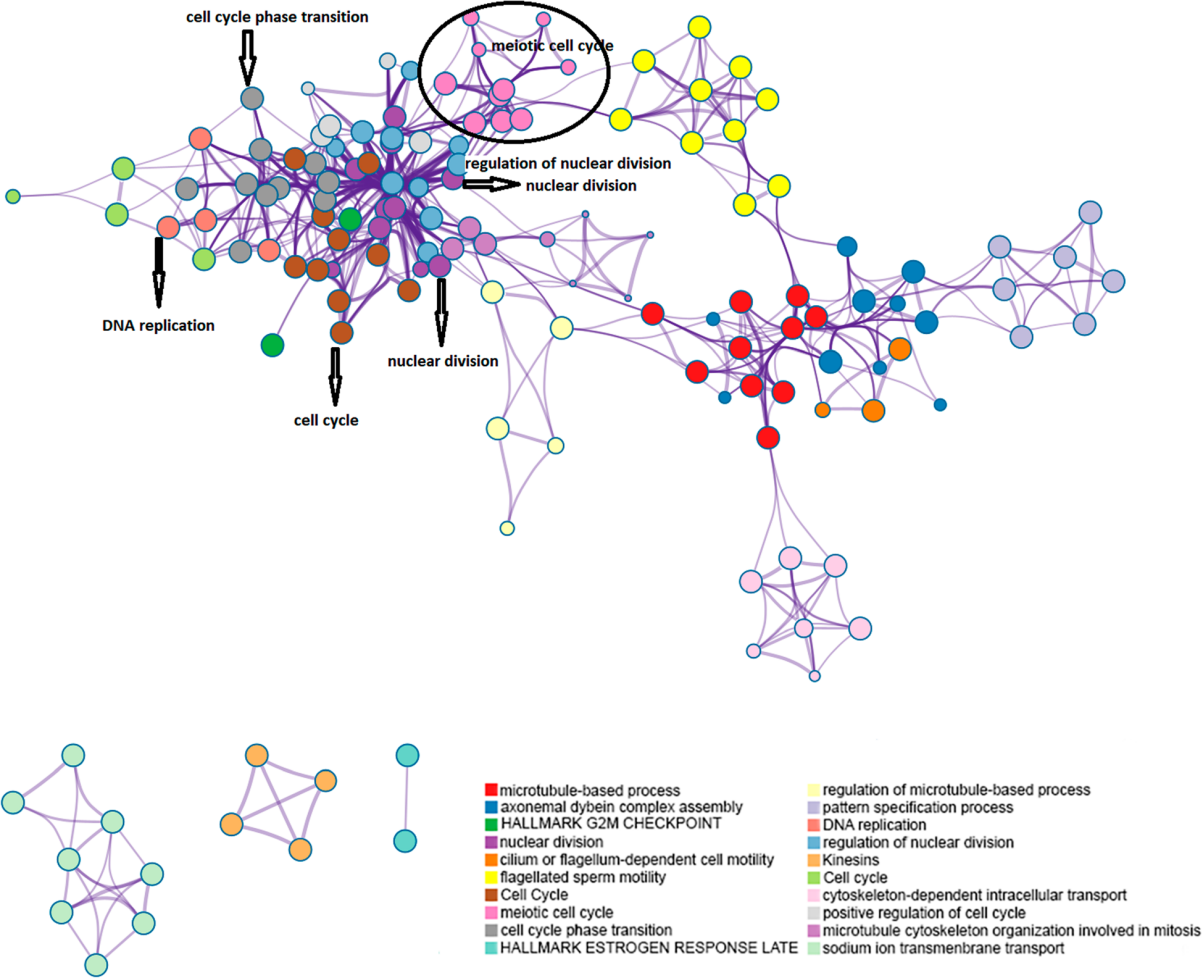
Enriched terms subset in up-regulated genes between Supplemented not Infected vs Control Not Infected. * A node represents each term; its size is proportional to the number of genes belonging to this term. Borders connect terms with a similarity larger than 0.3. Terms represented here have the best *p*-values from each of the 20 clusters. There are no more than 15 terms per cluster and no more than 250 terms in total. The nodes are coloured by cluster-ID, where nodes that share the same cluster-ID are generally close

The enriched terms full list in the up-regulated genes with their respective - log10 (*p*-values) is shown in Additional file 7. In Fig. 5, a subset of these enriched terms is pictured in a different perspective to show the connections among processes; a node pictures each term. Its size is proportional to the number of genes belonging to this term.

In the up-regulated gene list, among other biological processes, we found enriched: Meiotic cell cycle (GO - 0051321), DNA recombination (GO: 0006310); DNA methylation involved in gamete generation (GO:0043046), Regulation of meiotic nuclear division (GO: 0040020), DNA Replication (GO: 0006260), Meiotic cell cycle phase transition (GO:0044771), Sister chromatid cohesion (GO: 0007062); Kinetochore organisation (GO: 0051383), Male meiosis I (GO:0007141), Regulation of response to DNA damage stimulus (GO:2001020), Regulation of DNA endoreduplication (GO: 0032875), Positive Regulation of smoothened signalling pathway (GO:0045880), Response to food (GO: 0032094), Hallmark MTORC1 Signalling (M5924), Circadian behaviour (GO:0048512), Hallmark Estrogen Response Late (M5907); Hallmark Spermatogenesis (M5951).

#### Comparison between the control not infected vs supplemented not infected groups

We analysed the up-regulated genes full list to find which processes were more expressed in animals that were not infected and not supplemented with protein in their diets. Among other terms/biological processes we found enriched: Leukocyte Migration (GO: 0050900), Positive regulation of Cytokine Production (GO: 0001819); Myeloid leukocyte activation (GO:0002274), Leukocyte differentiation (GO:0002521), Hallmark Complement (M5921), Hallmark Inflammatory response(M5932), Cellular response to Growth Factor stimulus (GO: 0071363), Developmental Growth (GO:0048589), Tissue morphogenesis (GO: 0048729), Regulation of Cell Adhesion (GO: 0030155), Negative Regulation Of Cell Adhesion (GO:0007162); Extracellular matrix organisation (GO:0030198), Blood vessel development (GO: 0001568); the enriched terms full list in the up-regulated genes with their respective – log10 (*p*-values) is in Additional file 8. In Additional file 9, a subset of these enriched terms is pictured in a different perspective to show the connections among processes, a node pictures each term, where its size is proportional to the number of genes belonging to this term.

#### Comparison between the supplemented infected vs control infected groups

We analysed the full lists of genes differentially expressed in the comparison Supplemented Infected vs Control Infected to find the enriched pathways in the ewe lambs’ ovary due to the difference of protein’s level in the diet in the presence of an infection. The Metascape software identified 2187 gene identifiers, 2187 human Entrez Gene IDs.

**Fig. 6:**
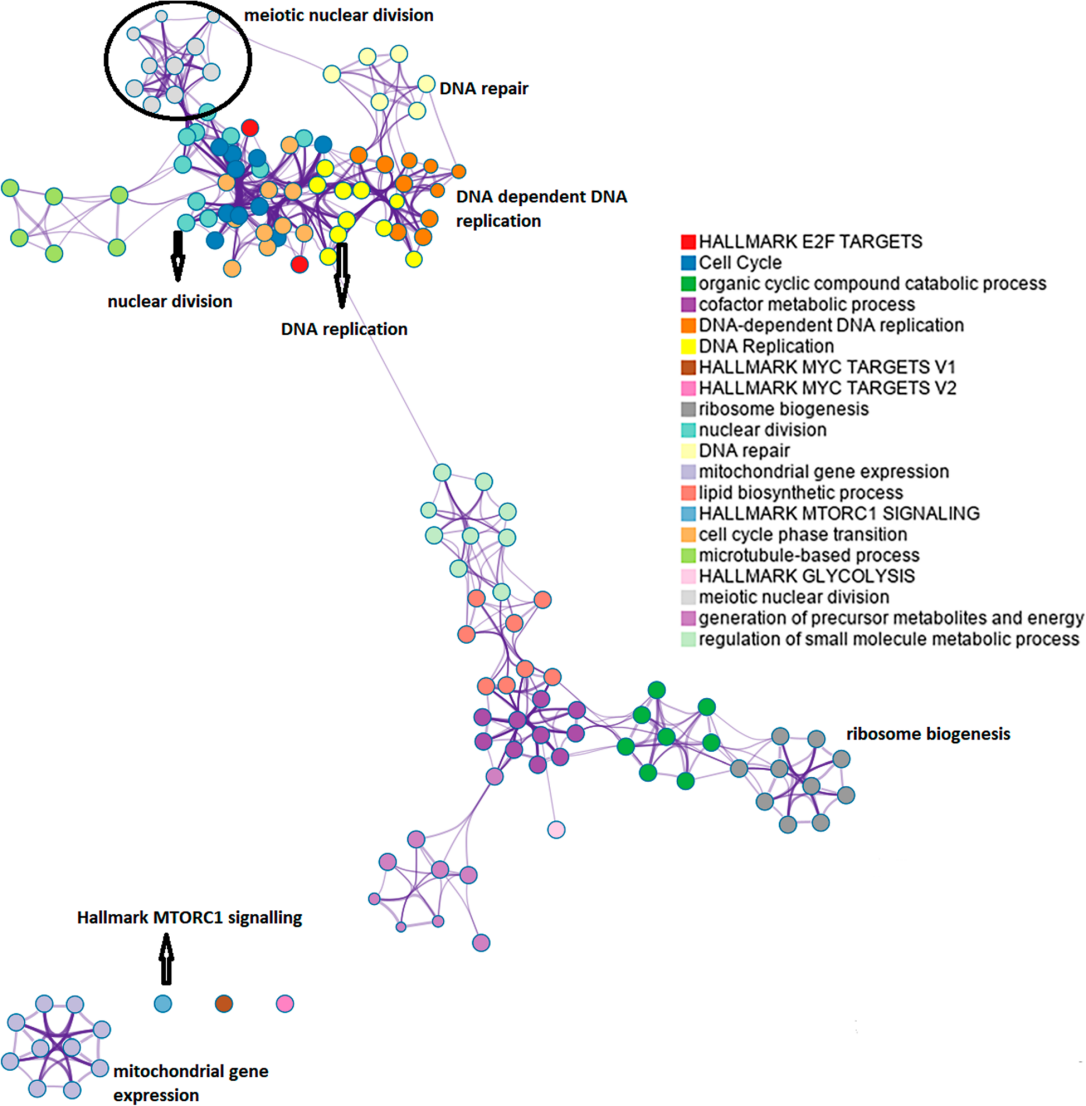
Enriched terms subset in up-regulated genes between Supplemented Infected vs Control Infected. * A node represents each term; its size is proportional to the number of genes belonging to this term. Borders connect terms with a similarity larger than 0.3. Terms represented here have the best *p*-values from each of the 20 clusters. There are no more than 15 terms per cluster and no more than 250 terms in total. The nodes are coloured by cluster-ID, where nodes that share the same cluster-ID are generally close

Among other biological processes and pathways enriched in the up-regulated genes list, we found: Meiotic nuclear division (GO:0140013), Female gamete generation (GO:0007292), Double-strand break repair via break-induced replication (GO: 0000727), HDR through Homologous recombination(R-HSA-5685942), DNA biosynthetic process (GO:0071897), Oocyte meiosis (hsa04114), Establishment of Chromosome localisation (GO:0051303), Methylation (GO: 0032259), DNA conformation change (GO:0071103), Hallmark E2F targets (M5925), DNA - dependent DNA replication (GO: 0006261), DNA replication (R-HSA-69306), Ribosome biogenesis (GO:0042254), Hallmark MTORC1 Signalling (M5924); the full list of enriched biological processes and pathways terms in up-regulated genes is in Additional file 10. Also, in Fig. 6, an enriched terms subset is pictured in a different perspective to show the connections among processes.

#### Comparison between control infected and supplemented infected groups

**Fig. 7:**
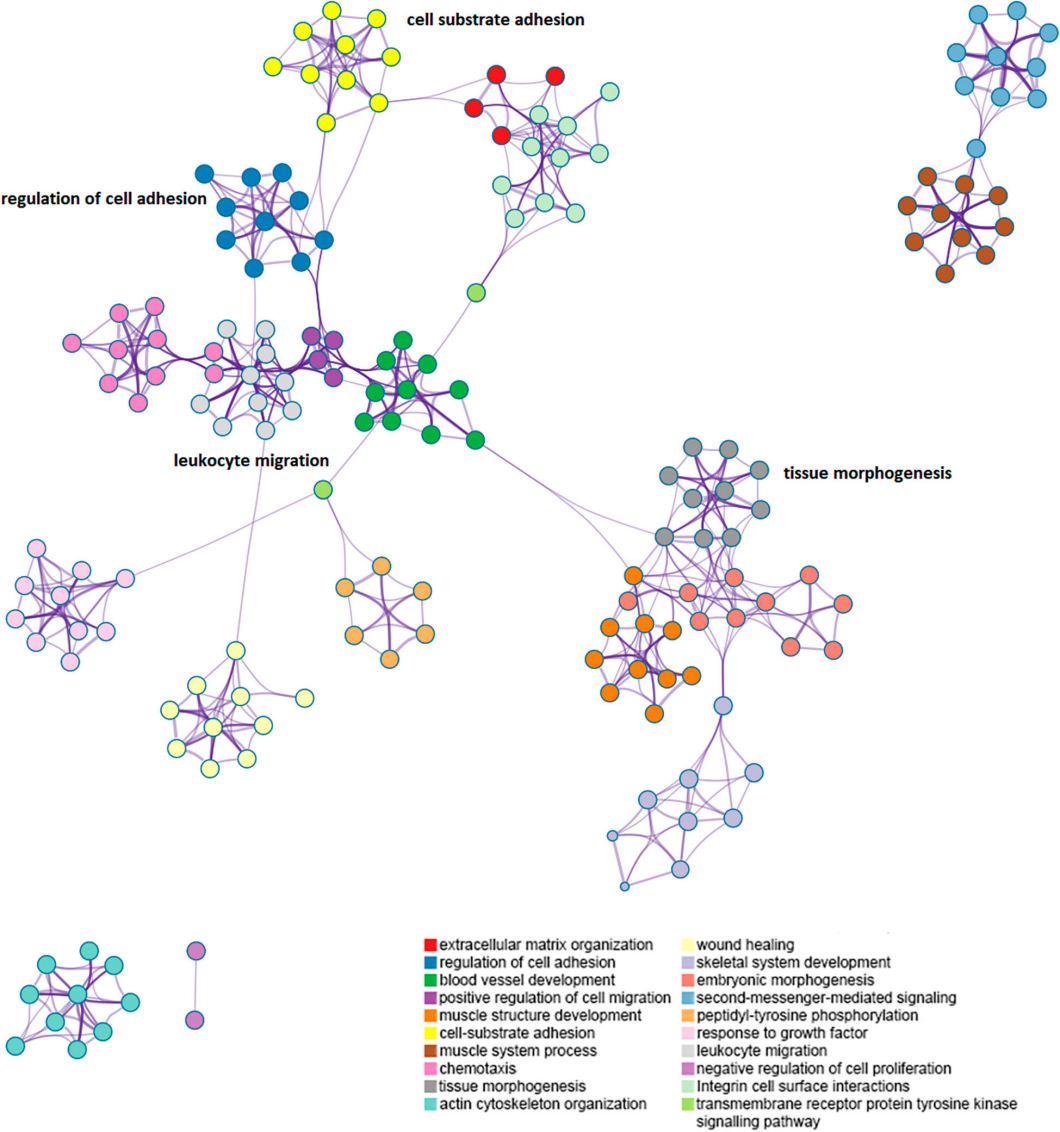
Subset of enriched terms in up-regulated genes between the groups Control Infected vs Supplemented Infected. * Each term is represented by a node; its size is proportional to the number of genes belonging to this term. Terms with a similarity larger than 0.3 are connected by borders, they have the best p-values from each of the 20 clusters. There are no more than 15 terms per cluster and no more than 250 terms in total. The nodes are coloured by cluster identification, where nodes that share the same cluster identification are generally close to each other

This comparison generated a list of 2304 up-regulated genes and 2186 down-regulated genes (Additional file 6). We analysed the up-regulated gene list in this comparison because we wanted to find which processes were enriched in the infected animals in the diet control protein. Among other biological processes and pathways enriched in the up-regulated genes list, we found: Leukocyte migration (GO:0050900), Regulation of cytokine production (GO:0001817), Hallmark Inflammatory response (M5932), Negative regulation of Immune system process (GO:0002683), Hallmark IL2 STAT5 signalling (M5947), Hallmark KRAS signalling up (M5953), Cytokine mediated signalling pathway (GO:0019221), Cytokine-cytokine receptor interaction (hsa04060), Hallmark TNFA signalling via NFKB (M5890), Inositol lipid-mediated signalling (GO: 0048017), Alpha-beta T cell activation (GO:0046631), Chemokine signalling pathway (hsa04060), Regulation of cell adhesion (GO:0030155), Extracellular matrix organisation (GO:0030198), Blood vessel development (GO:0001568), Tissue morphogenesis (GO: 0048729), Response to growth factor (GO:0070848), Wound healing (GO:0042060), Tissue remodelling (GO:0048771), Artery development (GO:0060840) and Endothelium development (GO: 0003158); the full list of enriched biological processes and pathways terms in up-regulated genes is in Additional file 11. In Fig. 7, a subset of these enriched terms was pictured in a different perspective to show the connections among processes.

### Gene expression validation by RT-qPCR

**Fig. 8:**
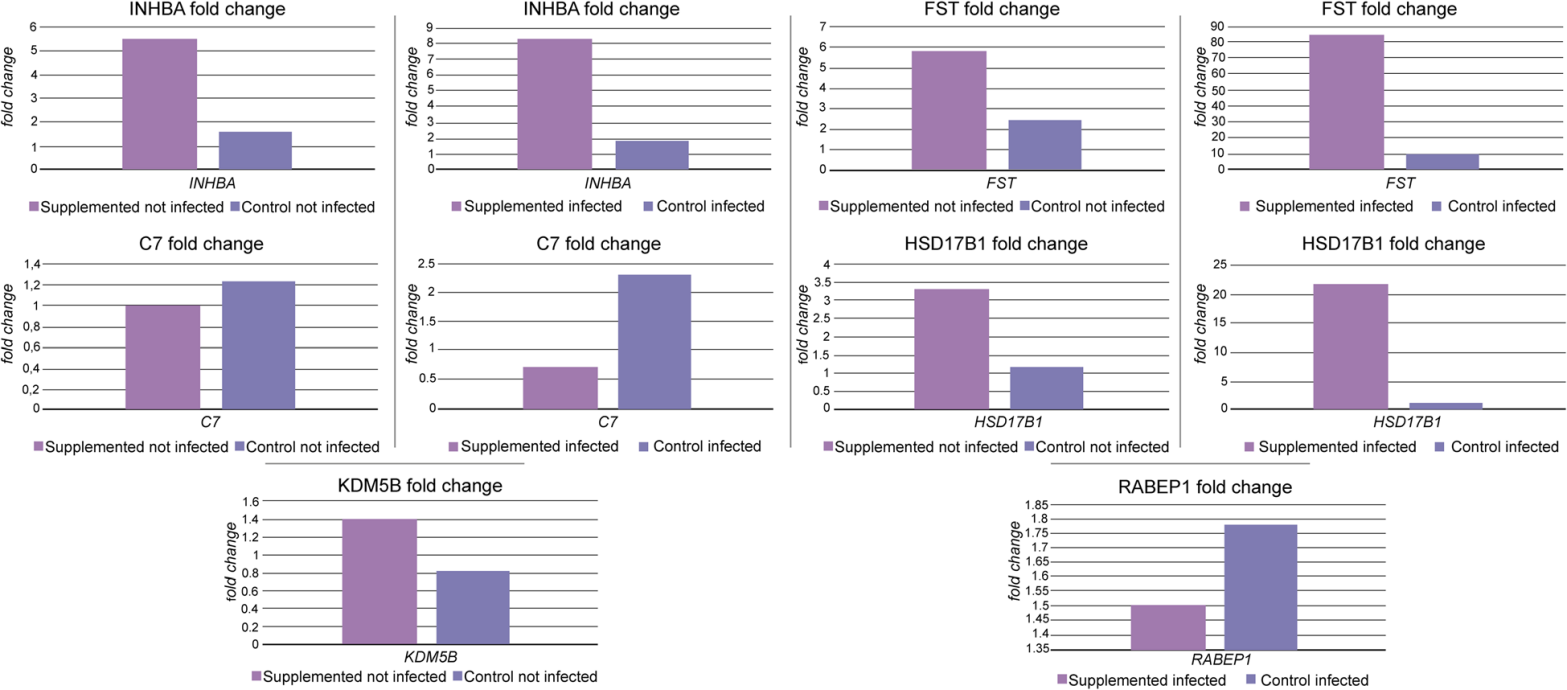
Gene expression differences measured by qPCR

In the genes assessed by RT-qPCR, the differential expression followed the same pattern as RNA sequencing differential expression analysis. While INHBA and HSD17B1 were up-regulated in both supplemented groups, the gene Complement 7 was down-regulated on both of them. KDM5B is down-regulated in Control not Infected, and RABEP1 is up-regulated in the control infected group. The differences in gene expression between groups Supplemented not Infected vs Control not Infected and Supplemented Infected vs Control Infected are shown in in Figure 8.

## Discussion

The immune response acquisition in a natural infection context could have a different metabolic cost generating a different result to our artificial infection experiment. Nevertheless, we based our experiment’s time frame on a same breed lambs’ study (group TST-SI) set in a natural infection scenario, where anti - *H. contortus* L3 IgG levels increased from 20% OD to 40% OD between weeks 3 and 5 post first infection [22]. We expected the lambs could develop a similar immune response 6 weeks after the infection with similar metabolic costs.

We did not find significant differences in body weight and *Haemonchus contortus* eggs number in both groups of infected ewe lambs. However, the supplemented groups presented higher plasma protein levels, haemoglobin, and red blood cells than the control groups. Differences were also noticeable between groups at the ovarian gene expression levels.

Both supplemented groups present enriched biological meiotic processes relating to control groups. Only germ cells are capable of meiotic division. Meiosis resumes at puberty when oocytes are stimulated to undergo the first meiotic division with each oestrous cycle. It is not until the oocyte reaches approximately 80% of its final size that resume meiosis [23]. When puberty begins, in each reproductive cycle, follicles grow to the preovulatory stage and are then stimulated by luteinising hormone (LH) from the pituitary to restart meiosis [24]. The fact that meiosis processes are up-regulated in the supplemented groups compared to control groups could mean that the extra protein favoured meiotic oocyte activation.

There are some differences among processes related to meiosis enriched in supplemented groups. Although both groups had reached a meiotic activation state, they seem to be in different moments of the meiotic activation state. These timing differences could be due to the infection’s impact on the whole organism. The evolutive investment of immune response to an infection covaries with reproductive scheduling (e.g. age at first reproduction). The interaction between supplementation of protein (abundance of nutrients) and pathogen exposure allowed the ovaries to naturally express an evolutive mechanism developed by mammals [11]. Examples of this evolutive mechanism are children that started reproducing at a younger age when exposed to cues of death’s likelihood from exogenous sources in their environment [25–29].

One of the pieces of evidence that supplemented infected lambs may be in a more advanced stage of meiotic activation is PLK1 and CDC42 genes up-regulation. PLK1 and CDC42 are up-regulated in both supplemented groups compared to their control groups. However, the fold change in the Supplemented Infected group is greater than in the Supplemented not infected group. These genes are part of the progression from Metaphase I to Metaphase II. When PLK1 gene expression decreases, it maintains the arrest in Metaphase I, and when it increases, it helps to segregate the chromosomes. When CDC42’s expression is increased, cytokinesis and polar body I extrusion occur in several mammalian species [30, 31].

Meiosis requires highly specialised chromosomal connections. Essential for homolog chromosome segregation during Metaphase I, the gene SMC1B (Structural Maintenance of Chromosomes 1B) is up-regulated in supplemented groups; it regulates the sister chromatid separation process, so sister centromeres are forced to act in tandem at metaphase I. The up-regulation of CENPT (Centromere protein T) that acts in kinetochore assembly) and CENPS (Centromere protein S) that intermediates resolution of meiotic recombination suggest that these forces are occurring in the ovaries of both supplemented groups. Another important event of meiosis is the spindle assembly. The genes F-BOX only protein 5 (FBXO5 or EMI1) and Aurora kinase A (AURKA), which participate specifically in spindle assembly involved in female meiosis, are up-regulated in supplemented groups. FBXO5’s role in oocyte meiosis leads to metaphase arrest of the second meiotic division before fertilisation [32]. AURKA accumulates to microtubules organising centres just before germinal vesicle breakdown, contributing to meiosis resumption in mice’s and bovine oocytes [33, 34].

Gonadotropins are required for antral follicle development. FSHR expression has been detected in follicles with one to two layers of granulosa cells in sheep. As the follicle growth progresses, it accumulates more cell layers, and in the final maturation stage, it becomes more responsive to LH [24, 35]. The Supplemented Infected animals present up-regulation of genes related to later follicle development as FSHR (follicle-stimulating hormone receptor), LHCGR (luteinising hormone receptor), STAR (Steroidogenic Acute Regulatory Protein) and PGR (progesterone receptor). PGR is induced to respond to the LH surge or an ovulatory dose of human chorionic gonadotropin (hCG) in rodents. The PGR mRNA and protein can be detected 4 h post-hCG, peaks at 8 h post-hCG, but not detected by 12 h post-hCG. Its localisation is consistent with LHCGR expression [36–38]. These genes were not up-regulated in the supplemented not infected group. This setting suggests that the follicles in Supplemented Infected - at a more mature stage - negatively regulated both gonadotropic hormones (FSH and LH). The ovarian follicles in Supplemented not Infected, being in a less mature stage, down-regulated only FSH expression but not that of LH because they still needed LH to develop further.

The gene inhibin A (INHBA) is only up-regulated in ewe lambs supplemented with protein in their diet despite all groups being of highly similar age and weight. INHBA expression is higher in antral follicles; it increases during puberty because the elevated levels of follicle-stimulating hormone (FSH) and luteinising hormone (LH) recruit more ovarian follicles to develop beyond the antral stage [39]. Even though INHBA is expressed in the middle phase of the luteal phase, many genes are responsible for orchestrating this phase, so it would be imprecise to determine if these groups were in this specific oestrus cycle phase. Besides, in the gene pathways enrichment analysis – which accounts for all the differentially expressed genes to define which processes are more or less enriched - the ovulation process was not present. Hydroxysteroid 17-Beta Dehydrogenase 1 (HSD17B1) gene expression was up-regulated in both groups fed supplemented protein. When one of the copies of this gene was not functional in female mice, they were subfertile [40], suggesting an essential role for HSD17B1 in female fertility.

Both supplemented groups present in common Hallmark MTORC1 signalling process. MTORC1 (mechanistic target of rapamycin complex 1) is an environmental tissue sensor activated by amino acids. So, the animal’s nutritional and physiological states are integrated by MTORC1 to regulate global protein synthesis rates. Activated MTORC1 phosphorylates proteins involved in mRNA translation to accelerate rates of total protein synthesis in cells. MTORC1 is an essential mechanism of primordial follicle activation in oocytes, although not necessary for the transition from primordial to the primary follicle [5]. Pharmacological inhibition of MTORC1 activity in vivo led to the suppression of primordial follicle activation. The suppressive effect of this inhibition on primordial follicle activation was reproduced in cultured ovaries [4]. Moreover, in pubertal female rats, acute activation of mTOR by l-leucine stimulates Luteinising Hormone secretion and partially rescues LH suppression caused by chronic food restriction [41]. In female mice, the overexpression of MTORC1 signalling caused premature primordial follicles activation [42]. Thus, if the MTORC1 signalling pathway is activated, it could be aiding primordial follicles activation. Excessive MTORC1 activation in older individuals is linked to chronic diseases like cancer, and the inhibition of MTORC1 increased life span. On the other side, its activation is necessary for young individuals for cell growth, proliferation, differentiation and metabolism in response to hormones leading to appropriate development [43].

Specific transcription factors for primordial follicle activation (LHX8, NOBOX, SOHLH1 and FOXL2) were up-regulated in the supplemented infected group compared to the Control infected. The FIGLA (Folliculogenesis - specific basic helix-loop-helix) transcription factor gene is also up-regulated in the supplemented not infected group. FIGLA is a transcription factor regulating the expression of oocyte-specific genes that initiate folliculogenesis. Its increased expression was associated with primordial follicles formation [44–46]. NOBOX (NOBOX Oogenesis Homeobox) deletion in ovaries disrupted the transition of primordial follicles to primary follicles and led to a loss of follicles after birth [47]. LHX8 (LIM Homeobox 8) deficiency in ovaries also caused a problem in the transition from primordial to growing follicles and follicles survival [48]. The ovaries with deletion of SOHLH1 (Spermatogenesis And Oogenesis Specific Basic Helix-Loop-Helix 1) present primordial follicles limited growth, and their granulosa cells did not differentiate into cuboidal cells [49]. Female mice that lack FOXL2 (Forkhead Box L2) had a premature ovarian failure, follicles’ somatic cells failed to develop around growing oocytes [50].

The biological process Response to food was enriched only in the Supplemented not Infected group’s up-regulated processes. Pubertal timing is controlled metabolically to prevent fertility when energy is not sufficient in the body. This correlation between pubertal age and nutrition involves peripheral hormones communicating to the hypothalamus that the animal achieved a reasonable body condition to be fertile [51, 52]. Nucleobinding 2 gene (NUCB2 or Nesfatin 1) is up-regulated and participates in the Response to food process. This gene modulates appetite and energy expenditure, activates the sympathetic nervous system, and plays a crucial role in typical puberty onset in rats. Pubertal female rats fasting for 48 h led to decreased hypothalamic levels of NUCB2 mRNA and protein [53]. Our lambs presented normal beta-hydroxybutyrate levels; its levels reliably indicated energy balance [54] so we may assume that they were not in a metabolic scenario similar to fasting.

Circadian behaviour process was only found up-regulated in the Supplemented not Infected group compared to the Control not Infected group. Maybe the Circadian behaviour process was enriched in supplemented not infected but not infected animals because parasite infections cause disruption of circadian rhythms/behaviour in the hosts. The circadian clock in mice immune cells modulated Leishmania infection’s magnitude [55]. Mice infected with malaria (*Plasmodium chabaudi)* had their circadian rhythms in behaviour and physiology disrupted [56]. There is a relationship between the expression pattern of circadian genes and steroidogenesis in the human ovary [58]. Melatonin receptors are present in the ovary - in human preovulatory follicles, sheep antral follicular fluid and oocytes and cows cumulus cells [59–61]. The circadian melatonin rhythm is essential for synchronising reproductive response to mammals’ environmental conditions [62], especially in sheep – seasonal poliestric animals. The reproductive cycle and diseases can disrupt the timing of clock gene expression in the ovary. Disruption of the circadian rhythm negatively affects reproductive function and fertility in rodent models and women [57, 63]. It is probably a positive finding that the Circadian behaviour process was up-regulated considering these arguments.

The Smoothened signalling pathway (also known as Hedgehog signalling) is a process enriched only in the up-regulated gene list of Supplemented not Infected. The smoothened signalling pathway cooperates in the follicles’ development because it allows the granulosa cells to induce the expression of target genes in theca cells. This communication is fundamental to the ovarian folliculogenesis and steroidogenesis [64]. The genes HHIP (Hedgehog interacting protein), IHH (Indian Hedgehog protein) and DHH (Desert Hedgehog protein) found enriched in the protein network analysis (Additional file 19) are part of the Hedgehog signalling pathway. Female mice lacking DHH and IHH are infertile due to theca cells’ absence and their product androgen [65], so we may deduce that the interaction of these genes/proteins could positively impact the ovarian function of Supplemented not infected animals.

Another enriched process up-regulated in both groups is the Microtubule-based process. The microtubule’s population is found when cell structures composed of microtubules need to be assembled and disassembled quickly. It is a process that organises cell structure and prepares it for division. The meiotic spindle positioning, migration, anchoring, and rotation are determined by microtubule and actin filaments in mouse oocytes. Oocytes rely on microtubules’ intrinsic properties and their associated motor proteins to set up the meiotic spindle [66] . As both groups have many enriched processes related to nuclear division, the microtubule-based process is a necessary previous step. Similarly, DNA replication is a necessary previous step to mitosis and meiosis, so it is not surprising to be enriched in both supplemented groups.

Reproduction and innate immunity are conserved and driving forces throughout evolution - they are integrated and divide common pathways [67]. We found on both up-regulated gene lists of not supplemented groups several enriched processes related to inflammation and immune response. Interactions between the reproductive hormones and the immune system exist. Rheumatoid arthritis and systemic lupus erythematosus symptoms improve in women during pregnancy when hormone levels increase [68]. In both not supplemented groups, the TNFA signalling process is enriched in the up-regulated genes list. TNFA protects immature follicles from gonadotropins employing inhibitory effects on gonadotropin receptors’ expression. These inhibitory effects might be necessary for the maintenance of follicles for future ovulations. Mice lacking the TNFA’s receptor had increased ovarian responsiveness to gonadotropins during the prepubertal period [69]. According to this finding, in theory, the TNFA signalling pathway could be repressing gonadotropin action in the ovarian follicles because they were not mature enough. However, these ewe lambs had already reached between 10 and 11 months of age and were on time to activate their follicles. TNFA signalling enriched may be seen as a sign that the not supplemented groups’ ovarian follicles were in a less mature stage.

On both not supplemented groups up-regulated lists, there were shared processes related to tissue morphogenesis. However, we found more processes related to tissue morphogenesis in the Control Infected than in the Control not Infected. These enriched processes could be a part of the tissue morphological homeostasis - a process responsible for maintaining differentiated tissues. The tissue control system is done by monocyte-derived cells, T lymphocytes and immunoglobulins, and autonomic innervation, which controls the number and volume of cells and some vascular endothelial cells [70]. When there is an intense remodelling of the ovary, some immune processes have to be up-regulated [71, 72]. The processes of “Developmental growth, Reproductive system development and response to growth factor stimulus” were up-regulated compared to supplemented protein animals. We could deduce that, at this moment, the ovary of not supplemented animals is prioritising growing or remodelling its tissue instead of meiotic activation.

It could be argued that earlier primordial follicle activation and meiotic activation of preovulatory follicles could lead to premature depletion of the ovarian reserve. However, these animals were all half-sisters in the expected age for pubertal timing. Furthermore, they were infected with *Haemonchus contortus*, a debilitating infection that could have delayed oocyte maturation. Our findings suggest that protein supplementation allowed follicle activation in the group supplemented with protein despite infection deleterious effects. At the same time, their half-sisters not supplemented did not up-regulate genes and processes related to follicle activation.

## Conclusions

Therefore, in our experiment, protein supplementation enabled the supplemented infected animals to proceed with follicles meiotic activation, which did not occur in the control infected group. Also, the ovaries of ewe lambs supplemented not infected reached a meiotic activation stage when the control not infected did not. Our results show consequences on the reproductive health of the nutrition and infection interaction effects. More importantly, when ovarian activation happens without delays, this ewe will produce proportionately more lambs in her life than the one reaching this activation later.

## Methods

This research project with protocol and procedures employed was ethically reviewed and approved by the Bioethics Commission of the University of São Paulo (CENA-USP, protocol number 004/2017), which complies with animal research ethics principles.

This study was prospective, randomised and controlled. Blinding was used during the steps of outcome assessment and data analysis. The person doing the measurements and analysis did not know to which group the animals belonged.

We aimed to verify how protein supplementation in the diet of peripubertal ewe lambs with an abomasal nematode infection would affect their ovary gene expression. We examined the ovarian gene expression to evaluate if supplementing dietary protein would benefit the ovarian tissue conducting to follicle activation despite damaging effects caused by infection.

### Experimental design, animals and diets

The 18 Santa Ines breed ewe lambs (*Ovis aries*) we used for this experiment were all half-sisters – bred by the same ram – they were six to 7 months old at the beginning of the experimental period. The lambs were randomly allocated to four different groups - Control Not Infected (*n* = 4), Supplemented Not Infected (n = 4), Control Infected (*n* = 5) and Supplemented Infected (n = 5). After the groups were formed, there were no significant differences in age and weight among the groups as verified by one-way ANOVA. Their average weight and age are detailed in Additional file 12.

The housing environment in which the lambs were raised and kept was helminth-free; they were monitored every 2 weeks for the presence of the helminth *H.contortus’* eggs in their faeces. The housing environment was illuminated by natural light and dark periods during the whole experiment.

The animals were housed in individual pens; the feed was given twice a day individually (8 am and 4 pm), and the water was provided ad libitum. They were fed a 12% protein isocaloric diet (control groups) or a 19% protein isocaloric diet (supplemented groups). The composition of the diets is defined in Additional file 13, and their bromatological composition is detailed in Additional file 14. The methodology of the bromatological analysis is described in Additional file 15. We followed the general guidelines of the National Research Council (2007) to formulate their diets. The diet’s amount was calculated for the lambs’ body weight and re-calculated every 2 weeks, observing their current body weight.

After 35 days of consuming this diet, the ewe lambs were either orally infected (Infected groups) or not (Not Infected groups) with 10,000 stage 3 larvae of the nematode *Haemonchus contortus.* The not infected animals went through a sham infection procedure. Following 77 days of consuming this diet and 42 days of being infected (or not), they had their left ovaries collected by surgical ovariectomy to examine the ovary’s gene expression through RNA sequencing.

### Ovariectomy

The animals fasted for 12 hours of food and 6 hours of water. Before the ovariectomy, the ewe lambs were premedicated with intravenous (IV) diazepam at 0.5 mg/ kg. General anaesthesia was induced with IV xylazine 0.2 mg/kg and 10 mg/kg ketamine ten minutes after premedication. The anaesthesia was maintained associating IV xylazine 0.1 mg/kg and IV ketamine 7.5 mg/kg [73]. Respiratory and heart rates were monitored during premedication, surgical and post-operative periods. We applied subcutaneous lidocaine at 2% in the ventral midline coinciding with the surgical incision extension. A surgical incision of approximately 10-centimetres was done in the *linea alba*. After locating the left uterine horn, the ovary pedicle was sutured, and the left ovary was removed. The animals received intramuscular (IM) flunixin meglumine 1.1 mg/kg and IM oxytetracycline 20 mg/kg at the moment of skin suture.

### Absence of oestrus synchronisation in the ewe lambs

To ensure our objective of studying the effects of diet’s protein supplementation in the infected animals’ ovary gene expression, we opted not to synchronise the ewe lambs’ oestrus. Hormonal therapy to synchronise the oestrus would probably affect ovarian gene expression, becoming a confounding factor in our study.

### Blood collection, complete blood count and blood biochemical analysis

Blood samples were collected on four different dates throughout the experimental period. On the first date, the lambs had been on a supplemented or control protein diet for one month but had not been infected yet. The three subsequent measurements were done after the artificial oral infection with the 10,000 Haemonchus contortus L3 stage larvae. We collected 5 mL of blood from the animals’ jugular veins before they were fed in the morning. The blood was collected in Vacutainer tubes with or without EDTA. The blood samples in EDTA were analysed in the automatic analyser Davol Poch-100iV Diff immediately after their collection. We analysed haematocrit, number of red blood cells (RBC), number of white blood cells (WBC) and haemoglobin (HGB). The samples that did not contain EDTA were centrifuged for 15 minutes at 1310g at 4°C to obtain blood serum aliquoted and kept in storage at -20°C until the biochemical analysis was performed. The plasma albumin levels, glucose, urea and total protein were measured with Labtest kits (Labtest Diagnostica S.A.- Brazil) following the manufacturer's instructions and their reading performed by spectrophotometer (Perkin Eusing Elmer – Lambda EZ150 UV/Vis Spectrophotometer).

### White blood cell counts

Counting and differentiation of the white blood cells were done with the blood smears of each animal on the four dates. We counted the first visualised 100 cells in the microscope at a 100X magnification of a Nikon Eclipse E200 microscope [74].

### Faecal egg count

The infection's level was monitored every fourteen days before and after the infection. We monitored them before the infection to ensure they were infection-free and after the infection to measure the infection level. We counted the faecal *H.contortus* eggs of each individual. The faeces were sampled directly from the ewe lambs’ rectum, and the eggs present were counted in a McMaster chamber under the Nikon Eclipse E200 microscope according to the methodology by [75].

### Beta-hydroxybutyrate levels

Beta-hydroxybutyrate's levels were measured on three different dates throughout the experiment to assess the animals’ energetic balance. The first measurement was done after the animals had been one month in the diet but had not been infected yet; the two subsequent measurements were done after the infection. We assessed its concentration with the Freestyle Optium Beta-Ketone test (Abbot) just after its collection in the morning, before feeding the animals.

### Statistical analyses of faecal egg counts, beta-hydroxybutyrate levels, blood cell and biochemical parameters

We used standardised parameters’ values for the following described statistical analyses. Standardisation was done by subtracting the individual values from the group's mean and dividing them by the standard deviation value. Shapiro-Wilks tests were performed on all the datasets to ensure data normality.

We performed Pearson correlation analysis in the plasma parameters (plasma protein, albumin, haemoglobin and glucose), whole blood beta-hydroxybutyrate, and the number of red blood cells to ensure a non-collinear relationship between them. We used a factorial ANOVA to assess the variation due to the interaction of protein in the diet (Supplemented versus Control) with infection status (Infected versus Not Infected). We also performed ANOVA repeated measures to evaluate the effect over time in the four different data collections. We analysed covariance between the measured parameters following a pattern where the protein level on the diet (Supplemented or Control) and Infection status (Infected or Not Infected) were categorical factors; the plasma variables were the dependent variables and AMH the continuous predictor. The analyses above were performed in the software STATISTICA (StatSoft - version 12).

### Ovarian RNA extraction

After the left ovary was removed through surgical ovariectomy, it was snap-frozen in liquid nitrogen and kept in a -80°C freezer until RNA extraction was performed. Total RNA was extracted from the ovary fragment using the Trizol reagent methodology (ThermoFisher, Waltham, Massachusetts, USA). In brief, the frozen ovarian fragments were macerated with a pestle and mortar in liquid nitrogen until they were pulverized. Quickly after that, Trizol reagent was poured over, and the sample was again macerated. The tissue lysate was then centrifuged and incubated subsequentially with chloroform and 2-propanol. The RNA pellet, formed after the 2-propanol incubation, was washed twice with ethanol 75% and resuspended in RNAse free water.

### RNA quality control

RNA Samples were verified for their purity and concentration by absorbance analysis of their 260/280 and 260/230 ratios in a spectrophotometer (NanoDrop 2000, Wilmington, DE) (Additional file 16). To determine its integrity, we analysed 500ng of each RNA sample in an agarose gel. Some samples were not analysed in the agarose gel because there was not enough RNA for the gel and sequencing. The RNA samples were further analysed through Agilent 2100 Bioanalyser at Novogene (San Diego-CA-USA) for precise quantification and integrity determination.

### cDNA’s library elaboration

After the RNA quality control analysis, the RNA was enriched using beads oligo-DT that attach to the poly-A tail of the messenger RNAs to select them specifically. After this step, the messenger RNA was randomly fragmented with fragmentation buffer addition. The cDNA was then synthesized using the messenger RNA as a template and random hexamer primers as building blocks. After this, a buffer specific for the second strand synthesis plus dNTPs, RNase H and DNA polymerase one were added to initiate the second strand synthesis. At the end of this stage, the terminations were repaired, linked with A, linked to the adaptor for sequencing, and the cDNA library was completed after size selection and PCR enrichment. The cDNA library passed three quality controls: a preliminary concentration measurement with Qubit, a test to measure the insert size, and a quantitative PCR to assess the library size effectively. Library Sequencing and RNA sequencing data analysis.

The qualified libraries were put into the Illumina Hiseq 2000 (Illumina- San Diego- USA) sequencer after being grouped according to their effective concentration and expected data volume. The RNA sample of the ewe lamb 1715 did not have enough RNA to perform sequencing.

The reads generated by RNA sequencing were analysed using the software CLC Genomics Workbench v 12.02 (QIAGEN, Aarhus, Denmark) after the sequencing reads were imported to the software environment. The sequencing reads were imported to the software using the section “Illumina High Throughput Sequencing Import”. Inside this section, the selected options were “paired reads; discard reads names; paired-end (forward-reverse); minimum distance 1, maximum distance 1000; remove failed reads”. The distance measurement used includes the complete read's sequence, what in the case of paired-end libraries, the measured distance goes from the beginning of the forward read to the beginning of the reverse read.

### Reads mapping and reference genome

The option used for the mapping step was: “Genome annotated with transcripts”, where the RNA splicing is taken into consideration. The annotations linked to the RNA transcripts were used to define how the transcripts were amended. In this option, RPKM and TPM's expression values were calculated based on the length of the transcripts supplied by the mRNA tracks. It was allowed two mismatches maximum, and in the counting scheme, the broken pairs were included – in which a pair of sequences is counted as two, and a single sequence is counted as one.

We mapped the sequenced reads to the sheep (*Ovis aries*) reference genome version Oar_rambouillet v. 1.0 (2017). The reference genome was imported to the software CLC Genomics Workbench v 12.02 (QIAGEN, Aarhus, Denmark) through its “Import Tracks” tool. The reference genome had been previously downloaded from NCBI (National Center for Biotechnology Information) database (https://www.ncbi.nlm.nih.gov/genome/?term=Ovis+aries - Genbank assembly). The genomes’ reference sequence was obtained in separated FASTA format files and the genome annotations through only one GFF/GFF3 combined file. The sequencing reads’ chromosomes were named in the same way as the reference genome for the adequate files’ association.

### Normalisation of RNA sequencing data

The normalisation was necessary because the sequencing depth differed among samples; therefore, they were compared without bias. The normalisation method used was the weighted trimmed mean of the log expression ratios (trimmed mean of M values-TMM) [76]. This method adjusts the library sizes based on the assumption that most genes are not differentially expressed.

### RNA sequencing analysis

In short, the RNAseq analysis was done according to the following steps. The annotated RNA transcripts were imported to the software environment using the tool RNAm track. The reads were mapped using the complete genome and transcripts. After this mapping, the reads were categorised and assigned to the transcripts using the estimation algorithm EM (its functioning is explained in the Additional file 17 – EM estimation algorithm procedure). The gene counts were obtained by adding over the (EM - distributed) transcript counts. The option chosen to measure the expression level was “Count paired reads as two” to ensure that each read of the pair is counted to the gene's expression to which the read overlaps. If a sequence was paired to multiple distinct places but less than the maximum number of hits established, this sequence was randomly assigned to one of these places. The EM algorithm did this random distribution.

### Differential expression analysis of the RNA sequencing data

The tool we used for the differential expression analysis of the RNA sequencing data performs a statistical test of differential expression for the set of expression tracks with associated metadata using multifactorial statistics based on a negative binomial model of the generalised linear model (GLM).

We used the RNA's sequencing tracks measuring expression at the gene level (GE tracks). The metadata associated was each track sample assignment to its belonging group Control Not Infected, Supplemented Not Infected, Control Infected or Supplemented Infected. For comparison between groups, the “ANOVA all group pairs” was chosen to test the differences between all the groups in one factor. We also used “age” as a controlling factor because, in the peripubertal developmental stage, a difference between the animals’ ages could cause differences in the gene expression.

Once we had the lists of genes differentially expressed identified (FDR p-value < 0.05), we searched on several databases to find out their function and in which biological processes they were found to be involved. We used the following databases for this search: Kyoto Encyclopedia of Genes and Genomes (KEGG) PATHWAY, Gene Ontology (GO) Project at Mouse Genome International (MGI), Molecular Signature Databases v7.0 (MSigDB), Database of Phenotypes and Genotypes at the National Centre of Biotechnology Information (dbGap-NCBI), Genome-Wide Association Studies catalogue (GWAS - National Human Genome Research Institute), Hallmark Gene Sets, Reactome Gene Sets and GeneCards - The Human Database.

### Analysis of differentially expressed gene lists to identify enriched pathways shared or selectively enriched between groups

This analysis was done with the software Metascape [77]. It combined searching for functional gene enrichment, protein-protein interaction analysis, gene annotation and membership using 40 independent databases. Also, a comparative analysis of datasets through orthogonal experiments was performed. The comparison among these datasets allowed identifying pathways/networks coherently and detected accurate signals above the experimental noise [78].

The protocol followed was the same for all the comparisons between gene lists. In the item “Annotation”, we selected the databases: Gene Symbol, Description, Biological process, Database of Genotypes and Phenotypes (dbGap-NCBI), GWAS, Variations, Kegg Pathways and Hallmark gene sets. In the item “Membership”, the selected databases for the analysis were: Reactome Gene Sets, Kegg Pathways, GO Biological process. In the item “Enrichment”, Kegg Pathways, Hallmark Gene Sets, GO Biological Process, and Reactome Gene Sets were selected. For the enrichment of pathways and biological processes, the parameters used were: Minimum Overlap 3, p-value cut-off 0.01, Minimum Enrichment 1.5 and for the enrichment of protein-protein interaction, we used the parameters: Minimum network size 3, Maximum network size 500 using the databases Biogrid, InWeb and OmniPath.

### Enrichment clustering

The whole genome was used as the enrichment background. Terms with a p-value smaller than 0.01, a minimum count of three, and an enrichment factor larger than 1.5 were selected and grouped into clusters based upon their membership affinity. P-values were calculated utilising the Benjamini-Hochberg method to account for multiple testing [79]. Each term within a cluster that was most significant was chosen to represent a given cluster.

During the data post-processing, the Kappa similarities between all the enriched pairs of terms were computed and used to join the terms hierarchically in a tree. They were fused in sub-trees of similar term groups. By absorbing most redundancies in representative groups, the enrichment clustering avoided confounding problems in data interpretation, which may arise when multiple ontologies are reported. However, the bar graph did not capture similarities and redundancies between the clusters. The enrichment network visualisation approach represents each enriched term with a node. These nodes are connected between pairs if their Kappa similarities were above 0.3, producing a network portrayed using Cytoscape [80]. Redundant terms inside a cluster conducted to form local complexes well-adjusted due to their high similarities intra-cluster. Clusters were occasionally linked to similar terms reflecting the relationship of two separate processes. The detailed statistical analysis used in the enrichment analysis and clustering is in Additional file 18.

### RT-qPCR to validate RNA Seq gene expression

To confirm the differential gene expression found in the RNA sequencing analysis between groups Supplemented not Infected vs Control not Infected and between the groups Supplemented Infected vs Control Infected, we performed RT-qPCR for the genes INHBA, HSD17B1, FST, C7, RABEP1 and KDM5B. The mRNA sequences used were obtained on the NCBI website (https://www.ncbi.nlm.nih.gov/nuccore/).To design the primers, we used the tool Primer 3 plus (https://primer3plus.com/cgi-bin/dev/primer3plus.cgi). The primer pairs’ quality was assessed with the tool NetPrimer (http://www.premierbiosoft.com/NetPrimer/AnalyzePrimerServlet), and the best-rated pair was chosen (Table 3). The primers specificity was verified by running the PCR product in gel electrophoresis and a melting curve analysis. According to the protocol described in the section Methods, RNA extraction was performed, and it was quantified with Nanodrop 2000 (Wilmington-USA). The RNA samples were treated with DNAse enzyme (Promega-Madison-USA), and then the reverse transcription reaction was performed to obtain cDNA. The reactions were performed according to instructions of the kit GoTaq- 2-Step RT-qPCR System (Promega-Madison-USA).

**Table 3:** Sequence, annealing temperature and product size of primers used for qPCR. *F = forward primer; R = reverse primer; product size in base pairs

Gene symbol	Accession no.	Species	Primer sequence 5′ - 3’	Annealing temperature(°C)	Product size (bp)
INHBA	NM_001009458.1	Sheep (*Ovis aries*)	F: GGACGGAGGGCAGAAATGAA	63.7	80
			R:TTCCTGGCTGTGCCTGATTC		
HSD17B1	XM_027974501.1	Sheep (*Ovis aries*)	F: CTTCTACCGCTACTGTCGCC	60	82
			R:GAGGAAGACCTCGACCACCT		
C7	XM_004017017.4	Sheep (*Ovis aries*)	F:TGCCTAAATGTCAGCCCTGG	62.6	84
			R:CATGCAAGGAGGACCCACAT		
FST	XM_012096672.3	Sheep (*Ovis aries*)	F:GGATCTTGCAACTCCATTTCG	61.9	119
			R:AACACTGAACATTGGTGGAGG		
RABEP1	XM_015098590.2	Sheep (*Ovis aries*)	F:GCTCAGTTATCAAATGAGGAGGAAC	61.3	87
			R:CCCGGATGGCAACAGTAAGT		
KDM5B	XM_027976024.1	Sheep (*Ovis aries*)	F:CTGCACTGTTGATTGGCTGC	63	98
			R:TGCAGATCATCTCGTCGTGG		
RPL7A	XM_027966154.1	Sheep (*Ovis aries*)	F:CAGCCTTTCAAGATGCCGAAG	62.5	113
			R:TTCTCGAACAGGGGGTTGAC		

In brief, total RNA - 1200 ng of each sample - were incubated with random primers at 70°C for 5 minutes and then at 4°C for 5 minutes. After that, it was mixed with GoScript 5X Reaction Buffer, MgCl2 25mM, PCR Nucleotide Mix - 10mM, Recombinant RNasin Ribonuclease Inhibitor and GoScript Reverse Transcriptase enzyme. The cycles for the reverse transcriptase reaction were annealing at 25°C for 5 minutes, extension at 42°C for 60 minutes and inactivation at 70°C. After this, the samples were stored at - 80°C until the qPCR reactions were performed. We used 15 nanograms of cDNA in 3 microlitres for each qPCR reaction, and each sample was performed in triplicate. The primers for each gene were used in the concentration of 900 nmol. The qPCR reactions followed 1 x 95°C for 5 minutes, then 50 cycles of hold at 95°C for 10 seconds, hold at [primer annealing temperature] for 25 seconds and hold at 72°C for 25 seconds. The melting curve was done with a ramp from [primer annealing temperature] to 95°C, with 90 seconds hold on the first step and 4 seconds hold on the next steps. These reactions were performed on the qPCR thermocycler Rotor-Gene Q 5plex HRM Platform (Qiagen-Denmark). PCR efficiencies were obtained with the LinRegPCR software [81]. The normalized Ct levels for the target genes were obtained from the subtraction of the Ct of the target gene out of the reference gene RPL7A (ribosomal protein L7a). The reference gene was chosen out of the RNA sequencing analysis expression data. We based the reference gene's choice on an analysis selecting the genes most highly expressed in all samples and the ones with the smallest variation (ANOVA) among samples.

## Supplementary Information

**Additional file 1: Table S1.** RNA sequencing data quality summary.

**Additional file 2: Table S2.** Number of sequences and Sequencing depth generated by RNA sequencing.

**Additional file 3: Table S3.** Percentage of mapping to gene regions.

**Additional file 4.** Full list of differentially expressed genes in supplemented not infected vs control not infected groups-converted.

**Additional file 5.** Full list of differentially expressed genes in supplemented infected vs control infected groups-converted.

**Additional file 6.** Full list of differentially expressed genes in control infected vs supplemented infected.

**Additional file 7: Figure S1.** Enriched terms in up-regulated genes between Supplemented Not Infected vs Control Not Infected.

**Additional file 8: Figure S3.** Enriched terms in up-regulated genes between Control Not Infected vs Supplemented Not Infected.

**Additional file 9.** Enriched terms subset in up-regulated genes between Control Not Infected vs Supplemented Not Infected.

**Additional file 10.** Enriched terms in up-regulated genes between Supplemented Infected vs Control Infected.

**Additional file 11.** Enriched terms in the list of differentially up-regulated genes between the groups Control Infected vs Supplemented Infected.

**Additional file 12.** Average liveweight and age (± standard deviation) for each group at the beginning and end of the experiment.

**Additional file 13.** Composition of the control protein and supplemented protein diets.

**Additional file 14.** Bromatological composition (g kg-1 of dry matter at 100 °C) and energy (MJ) of the diets’ ingredients.

**Additional file 15.** Bromatological analysis of the diet.

**Additional file 16.** List of ovarian RNA samples, their concentration, 260/280 and 260/230 absorbance ratios.

**Additional file 17.** EM estimation algorithm procedure.

**Additional file 18.** Statistical methodology used in the Enrichment analysis.

**Additional file 19.** Protein Network analysis.

## Data Availability

The datasets used and/or analysed during the current study are available in the Additional files. The files that are not in this section may be provided under reasonable request to Dr. Helder Louvandini – e-mail address: louvandini@cena.usp.br

## References

[1] Dupont J, Scaramuzzi RJ, Reverchon M. The effect of nutrition and metabolic status on the development of follicles, oocytes and embryos in ruminants. Animal. 2014:1031–44. 10.1017/S1751731114000937. 10.1017/S1751731114000937 24774511

[2] Webb R, Garnsworthy PC, Campbell BK, Hunter MG. Intra-ovarian regulation of follicular development and oocyte competence in farm animals. Theriogenology. 2007. 10.1016/j.theriogenology.2007.04.036. 10.1016/j.theriogenology.2007.04.036 17540442

[3] Gutiérrez CG, Oldham J, Bramley TA, Gong JG, Campbell BK, Webb R (1997). The recruitment of ovarian follicles is enhanced by increased dietary intake in heifers2. J Anim Sci.

[4] Tong Y, Li F, Lu Y, Cao Y, Gao J, Liu J (2013). Rapamycin-sensitive mTORC1 signaling is involved in physiological primordial follicle activation in mouse ovary. Mol Reprod Dev.

[5] Guo Z, Yu Q (2019). Role of mTOR signaling in female reproduction. Front Endocrinol.

[6] Toro CA, Aylwin CF, Lomniczi A (2018). Hypothalamic epigenetics driving female puberty. J Neuroendocrinol.

[7] Hunt PA, Hassold TJ (2008). Human female meiosis: what makes a good egg go bad?. Trends Genet.

[8] Amarante AFT, Bricarello PA, Huntley JF, Mazzolin LP, Gomes JC (2005). Relationship of abomasal histology and parasite-specific immunoglobulin A with the resistance to Haemonchus contortus infection in three breeds of sheep. Vet Parasitol.

[9] Muehlenbein MP, Hirschtick JL, Bonner JZ, Swartz AM (2010). Toward quantifying the usage costs of human immunity: altered metabolic rates and hormone levels during acute immune activation in men. Am J Hum Biol.

[10] Tena-Sempere M (2012). Deciphering puberty: novel partners, novel mechanisms. Eur J Endocrinol.

[11] McDade TW, Georgiev AV, Kuzawa CW (2016). Trade-offs between acquired and innate immune defenses in humans. Evol Med Public Heal.

[12] Mcrae KM, Stear MJ, Good B, Keane OM (2015). The host immune response to gastrointestinal nematode infection in sheep. Parasite Immunol.

[13] Avramenko RW, Redman EM, Windeyer C, Gilleard JS (2020). Assessing anthelmintic resistance risk in the post-genomic era: a proof-of-concept study assessing the potential for widespread benzimidazole-resistant gastrointestinal nematodes in north American cattle and bison. Parasitology..

[14] Jackson F, Varady M, Bartley DJ (2012). Managing anthelmintic resistance in goats-can we learn lessons from sheep?. Small Rumin Res.

[15] Imperiale FA, Busetti MR, Suárez VH, Lanusse CE (2004). Milk excretion of ivermectin and moxidectin in dairy sheep: assessment of drug residues during cheese elaboration and ripening period. J Agric Food Chem.

[16] Tsiboukis D, Sazakli E, Gortzi O, Hadjichristodoulou C, Matara C, Leotsinidis M (2010). Food Additives and Contaminants Assessing quality of raw milk in southern Greece in the aspect of certain benzimidazole residues. Food Addit Contam Part B Surveill.

[17] Sargison ND (2011). Pharmaceutical control of Endoparasitic helminth infections in sheep. Vet Clin North Am - Food Anim Pract..

[18] Davis IF, Brien FD, Findlay JK, Cumming IA (1981). Interactions between dietary protein, ovulation rate and follicle stimulating hormone level in the ewe. Anim Reprod Sci.

[19] Rodrigues M, Moreira Silva L, Manoel Gomes da Silva C, Alencar Araújo A, Célia Sousa Nunes-Pinheiro D, Rondina D (2015). Reproductive and metabolic responses in ewes to dietary protein supplement during mating period in dry season of Northeast Brazil. Cienc Anim Bras.

[20] Braun JP, Trumel C, Bézille P (2010). Clinical biochemistry in sheep: a selected review. Small Rumin Res.

[21] Ermilio EM, Smith MC (2011). Treatment of emergency conditions in sheep and goats. Vet Clin North Am - Food Anim Pract.

[22] Albuquerque ACA, Bassetto CC, Almeida FA, Hildersley KA, McNeilly TN, Britton C (2019). Differences in immune responses to Haemonchus contortus infection in the susceptible Ile de France and the resistant Santa Ines sheep under different anthelmintic treatments regimens. Vet Res.

[23] Albertini DF, Sanfins A, Combelles CMH (2003). Origins and manifestations of oocyte maturation competencies. Reprod BioMed Online.

[24] Jaffe LA, Egbert JR (2017). Regulation of mammalian oocyte meiosis by intercellular communication within the ovarian follicle. Annu Rev Physiol.

[25] Chisholm JS, Quinlivan JA, Petersen RW, Coall DA (2005). Early stress predicts age at menarche and first birth, adult attachment, and expected lifespan. Hum Nat.

[26] Burton LM (1990). Teenage childbearing as an alternative life-course strategy in multigeneration black families. Hum Nat.

[27] Nettle D, Coall DA, Dickins TE. Birthweight and paternal involvement predict early reproduction in British women: Evidence from the National Child Development Study. Am J Hum Biol. 2009;22. 10.1002/ajhb.20970. 10.1002/ajhb.20970 19670389

[28] Low BS, Hazel A, Parker N, Welch KB (2008). Influences on Women’s reproductive lives. Cross-Cultural Res.

[29] Gettler LT, McDade TW, Bragg JM, Feranil AB, Kuzawa CW (2015). Developmental energetics, sibling death, and parental instability as predictors of maturational tempo and life history scheduling in males from Cebu, Philippines. Am J Phys Anthropol.

[30] Leblanc J, Zhang X, McKee D, Wang ZB, Li R, Ma C, Sun QY, Liu XJ (2011). The small GTPase Cdc42 promotes membrane protrusion during polar body emission via ARP2-nucleated actin polymerization. Mol Hum Reprod.

[31] Sharma A, Tiwari M, Gupta A, Pandey AN, Yadav PK, Chaube SK (2018). Journey of oocyte from metaphase-I to metaphase-II stage in mammals. J Cell Physiol.

[32] Homer H (2013). The APC/C in female mammalian meiosis i. Reproduction..

[33] Saskova A, Solc P, Baran V, Kubelka M, Schultz RM, Motlik J (2008). Aurora kinase a controls meiosis I progression in mouse oocytes. Cell Cycle.

[34] Mahdipour M, Leitoguinho ARC, Zacarias Silva RA, Van Tol HTA, Stout TAE, Rodrigues G (2015). TACC3 is important for correct progression of meiosis in bovine oocytes. PLoS One.

[35] Fair T (2003). Follicular oocyte growth and acquisition of developmental competence. Anim Reprod Sci.

[36] Ismail PM, Li J, DeMayo FJ, O’Malley BW, Lydon JP (2002). A novel lacZ reporter mouse reveals complex regulation of the progesterone receptor promoter during mammary gland development. Mol Endocrinol.

[37] Teilmann SC, Clement A, Thorup J, Byskov AG, Christensen ST (2006). Expression and localization of the progesterone receptor in mouse and human reproductive organs. J Endocrinol.

[38] Akison LK, Robker RL (2012). The critical roles of progesterone receptor (PGR) in ovulation, oocyte developmental competence and oviductal transport in mammalian reproduction. Reprod Domest Anim.

[39] Namwanje M, Brown CW. Activins and inhibins: roles in development, physiology, and disease. Cold Spring Harb Perspect Biol. 2016;8(7). 10.1101/cshperspect.a021881. 10.1101/cshperspect.a021881 PMC4930927 27328872

[40] Kemiläinen H, Adam M, Mäki-Jouppila J, Damdimopoulou P, Damdimopoulos AE, Kere J, Hovatta O, Laajala TD, Aittokallio T, Adamski J, Ryberg H, Ohlsson C, Strauss L, Poutanen M (2016). The hydroxysteroid (17β) dehydrogenase family gene HSD17B12 is involved in the prostaglandin synthesis pathway, the ovarian function, and regulation of fertility. Endocrinology..

[41] Roa J, Garcia-Galiano D, Varela L, Sánchez-Garrido MA, Pineda R, Castellano JM (2009). The mammalian target of rapamycin as novel central regulator of puberty onset via modulation of hypothalamic Kiss1 system. Endocrinology..

[42] Adhikari D, Zheng W, Shen Y, Gorre N, Hämäläinen T, Cooney AJ (2009). Tsc/mTORC1 signaling in oocytes governs the quiescence and activation of primordial follicles. Hum Mol Genet.

[43] Shoveller AK, McKnight LM, Wood KM, Cant JP (2018). Lessons from animal nutritionists: dietary amino acid requirement studies and considerations for healthy aging studies. Ann N Y Acad Sci.

[44] Bayne RAL, da Silva SJM, Anderson RA (2004). Increased expression of the FIGLA transcription factor is associated with primordial follicle formation in the human fetal ovary. Mol Hum Reprod.

[45] Fowler PA, Flannigan S, Mathers A, Gillanders K, Lea RG, Wood MJ, Maheshwari A, Bhattacharya S, Collie-Duguid ESR, Baker PJ, Monteiro A, O'Shaughnessy PJ (2009). Gene expression analysis of human fetal ovarian primordial follicle formation. J Clin Endocrinol Metab.

[46] Lei L, Jin S, Mayo KE, Woodruff TK (2010). The interactions between the stimulatory effect of follicle-stimulating hormone and the inhibitory effect of estrogen on mouse primordial folliculogenesis. Biol Reprod.

[47] Rajkovic A, Pangas SA, Ballow D, Suzumori N, Matzuk MM (2004). NOBOX deficiency disrupts early folliculogenesis and oocyte-specific gene expressiong. Science (80- ).

[48] Choi Y, Ballow DJ, Xin Y, Rajkovic A (2008). Lim homeobox gene, Lhx8, is essential for mouse oocyte differentiation and survival. Biol Reprod.

[49] Lim EJ, Choi Y (2012). Transcription factors in the maintenance and survival of primordial follicles. Clin Exp Reprod Med.

[50] Uda M, Ottolenghi C, Crisponi L, Garcia JE, Deiana M, Kimber W, Forabosco A, Cao A, Schlessinger D, Pilia G (2004). Foxl2 disruption causes mouse ovarian failure by pervasive blockage of follicle development. Hum Mol Genet.

[51] Sanchez-Garrido MA, Tena-Sempere M (2013). Metabolic control of puberty: roles of leptin and kisspeptins. Horm Behav.

[52] Fernandez-Fernandez R, Martini AC, Navarro VM, Castellano JM, Dieguez C, Aguilar E, Pinilla L, Tena-Sempere M (2006). Novel signals for the integration of energy balance and reproduction. Mol Cell Endocrinol.

[53] García-Galiano D, Navarro VM, Roa J, Ruiz-Pino F, Sánchez-Garrido MA, Pineda R (2010). The anorexigenic neuropeptide, nesfatin-1, is indispensable for normal puberty onset in the female rat. J Neurosci.

[54] Raboisson D, Mounié M, Maigné E (2014). Diseases, reproductive performance, and changes in milk production associated with subclinical ketosis in dairy cows: a meta-analysis and review. J Dairy Sci.

[55] Kiessling S, Dubeau-Larameé G, Ohm H, Labrecque N, Olivier M, Cermakian N (2017). The circadian clock in immune cells controls the magnitude of Leishmania parasite infection. Sci Rep.

[56] Prior KF, O’Donnell AJ, Rund SSC, Savill NJ, van der Veen DR, Reece SE (2019). Host circadian rhythms are disrupted during malaria infection in parasite genotype-specific manners. Sci Rep.

[57] Sellix MT (2015). Circadian clock function in the mammalian ovary. J Biol Rhythm.

[58] Chen M, Xu Y, Miao B, Zhao H, Luo L, Shi H, Zhou C (2016). Expression pattern of circadian genes and steroidogenesis-related genes after testosterone stimulation in the human ovary. J Ovarian Res.

[59] Tamura H, Nakamura Y, Korkmaz A, Manchester LC, Tan DX, Sugino N, Reiter RJ (2009). Melatonin and the ovary: physiological and pathophysiological implications. Fertil Steril.

[60] Xiao L, Hu J, Song L, Zhang Y, Dong W, Jiang Y, Zhang Q, Yuan L, Zhao X (2019). Profile of melatonin and its receptors and synthesizing enzymes in cumulus-oocyte complexes of the developing sheep antral follicle - a potential estradiol-mediated mechanism. Reprod Biol Endocrinol.

[61] Tian X, Wang F, Zhang L, He C, Ji P, Wang J, et al. Beneficial effects of melatonin on the in vitro maturation of sheep oocytes and its relation to melatonin receptors. Int J Mol Sci. 2017;18(4). 10.3390/ijms18040834. 10.3390/ijms18040834 PMC5412418 28420163

[62] Armstrong SM (1989). Melatonin and circadian control in mammals. Experientia..

[63] Sciarra F, Franceschini E, Campolo F, Gianfrilli D, Pallotti F, Paoli D, et al. Disruption of circadian rhythms: a crucial factor in the etiology of infertility. Int J Mol Sci. 2020;21(11). 10.3390/ijms21113943. 10.3390/ijms21113943 PMC7312974 32486326

[64] Wijgerde M, Ooms M, Hoogerbrugge JW, Grootegoed JA (2005). Hedgehog Signaling in Mouse Ovary: Indian Hedgehog and Desert Hedgehog from Granulosa Cells Induce Target Gene Expression in Developing Theca Cells. Endocrinology.

[65] Liu C, Rodriguez KF, Brown PR, Yao HHC (2018). Reproductive, physiological, and molecular outcomes in female mice deficient in Dhh and Ihh. Endocrinology..

[66] Wang Q, Racowsky C, Deng M (2011). Mechanism of the chromosome-induced polar body extrusion in mouse eggs. Cell Div.

[67] Lutton B, Callard I (2006). Evolution of reproductive-immune interactions. Integr Comp Biol.

[68] Beagley KW, Gockel CM (2003). Regulation of innate and adaptive immunity by the female sex hormones oestradiol and progesterone. FEMS Immunol Med Microbiol.

[69] Roby KF, Son DS, Terranova PF (1999). Alterations of events related to ovarian function in tumor necrosis factor receptor type I knockout mice. Biol Reprod.

[70] Bukovsky A (2011). Immune maintenance of self in Morphostasis of distinct tissues, tumour growth and regenerative medicine. Scand J Immunol.

[71] Wu R, Van der Hoek KH, Ryan NK, Norman RJ, Robker RL (2004). Macrophage contributions to ovarian function. Hum Reprod Update.

[72] Wagner M, Yoshihara M, Douagi I, Damdimopoulos A, Panula S, Petropoulos S, Lu H, Pettersson K, Palm K, Katayama S, Hovatta O, Kere J, Lanner F, Damdimopoulou P (2020). Single-cell analysis of human ovarian cortex identifies distinct cell populations but no oogonial stem cells. Nat Commun.

[73] Ewing KK (1990). Anesthesia techniques in sheep and goats. Vet Clin North Am Food Anim Pract.

[74] Sohani AR. Identifying blood and bone marrow abnormalities in the laboratory. In: Diagnosis of Blood and Bone Marrow Disorders. Cham: Springer International Publishing; 2018. p. 1–15.

[75] Coles GC, Bauer C, Borgsteede FHM, Geerts S, Klei TR, Taylor MA (1992). World Association for the Advancement of Veterinary Parasitology (W.A.A.V.P.) methods for the detection of anthelmintic resistance in nematodes of veterinary importance. Vet Parasitol.

[76] Robinson MD, Oshlack A (2010). A scaling normalization method for differential expression analysis of RNA-seq data. Genome Biol.

[77] Zhou Y, Zhou B, Pache L, Chang M, Khodabakhshi AH, Tanaseichuk O, Benner C, Chanda SK (2019). Metascape provides a biologist-oriented resource for the analysis of systems-level datasets. Nat Commun.

[78] Bushman FD, Malani N, Fernandes J, D’Orso I, Cagney G, Diamond TL (2009). Host cell factors in HIV replication: Meta-analysis of genome-wide studies. PLoS Pathog.

[79] Benjamini Y, Hochberg Y (1995). Controlling the false discovery rate: a practical and powerful approach to multiple testing. J R Stat Soc Ser B.

[80] Shannon P, Markiel A, Ozier O, Baliga NS, Wang JT, Ramage D, Amin N, Schwikowski B, Ideker T (2003). Cytoscape: a software environment for integrated models of biomolecular interaction networks. Genome Res.

[81] Ruijter JM, Ramakers C, Hoogaars WMH, Karlen Y, Bakker O, van den Hoff MJB (2009). Amplification efficiency: linking baseline and bias in the analysis of quantitative PCR data. Nucleic Acids Res.

