# Supplementary material for: Ovarian activation delays in peripubertal ewe lambs infected with Haemonchus contortus can be avoided by supplementing protein in their diets
Source: BMC Vet Res. 2021 Nov 3;17:344. doi: 10.1186/s12917-021-03020-7 (PMC8565066; doi:10.1186/s12917-021-03020-7)
Supplement: Supplementary file 1 — Additional file 1: Table S1. RNA sequencing data quality summary. [file 12917_2021_3020_MOESM1_ESM.pdf]

Ovarian activation delays in peripubertal ewe lambs infected with *Haemonchus contortus* can be avoided by supplementing protein in their diets

Paula Suarez-Henriques, Camila de Miranda e Silva-Chaves, Ricardo Cardoso-Leite, Danielle G. Gomes-Caldas, Luciana Morita-Katiki, Siu Mui Tsai, Helder Louvandini

Additional file 1: Table 1. RNA sequencing data quality summary

Sample	Raw Reads	Clean Reads	Raw Base(G)	Clean Base(G)	Effective Rate(%)	Error Rate(%)	Q20(%)	Q30(%)	GC Content(%)
B4	24254764	23287287	7.3	7.0	96.01	0.03	97.86	94.59	54.38
B8	44927957	44419032	13.5	13.3	98.87	0.03	96.66	92.61	59.63
A9	33463044	32569875	10.0	9.8	97.33	0.02	98.07	94.93	52.69
A18	20745903	19471831	6.2	5.8	93.86	0.02	97.97	94.75	52.85
A19	28202604	27983564	8.5	8.4	99.22	0.03	96.09	90.66	54.48
A20	32232047	31370541	9.7	9.4	97.33	0.03	97.38	93.99	57.19
B21	29113823	27363768	8.7	8.2	93.99	0.03	97.58	94.27	56.51
B23	29536890	28869927	8.9	8.7	97.74	0.03	96.45	91.28	52.69
B24	24821414	24244630	7.4	7.3	97.68	0.02	98.02	94.72	51.55
A28	30591298	29980521	9.2	9.0	98.00	0.03	97.92	94.56	53.42
A32	28459885	27334382	8.5	8.2	96.05	0.02	98.09	94.91	52.88
A33	38037046	36050761	11.4	10.8	94.78	0.03	97.41	93.40	53.97
A35	24058204	22874912	7.2	6.9	95.08	0.02	98.04	94.89	53.22
A40	28344939	26734222	8.5	8.0	94.32	0.02	98.09	94.96	53.74
C22	27175486	24885063	8.2	7.5	91.57	0.03	97.61	94.16	52.34
B24_1	26497214	26099781	7.9	7.8	98.50	0.03	98.00	94.65	53.30
B3	19073483	18755024	5.7	5.6	98.33	0.02	98.09	94.86	52.33
A34	21526666	21229329	6.5	6.4	98.62	0.02	98.62	95.94	52.10

Sample: sample name

Raw reads: total amount of reads of raw data, each four lines taken as one unit. For paired-end sequencing, it equals the amount of read1 and read2, otherwise it equals the amount of read1 for single-end sequencing.

Clean reads: total amount of reads of clean data, each four lines taken as one unit. For paired-end sequencing, it means the amount of read1 and read2, otherwise it equals the amount of read1 for single-end sequencing.

Raw bases: (Raw reads) * (sequence length), calculating in G. For paired-end sequencing like PE150, sequencing length equals 150, otherwise it equals 50 for sequencing like SE50.

Clean bases: (Clean reads) * (sequence length), calculating in G. For paired-end sequencing like PE150, sequencing length equals 150, otherwise it equals 50 for sequencing like SE50.

Effective Rate(%): (Clean reads/Raw reads)*100%

Error rate: base error rate

Q20, Q30: (Base count of Phred value > 20 or 30) / (Total base count)

GC content: (G & C base count) / (Total base count)
