# Supplementary material for: Ovarian activation delays in peripubertal ewe lambs infected with Haemonchus contortus can be avoided by supplementing protein in their diets
Source: BMC Vet Res. 2021 Nov 3;17:344. doi: 10.1186/s12917-021-03020-7 (PMC8565066; doi:10.1186/s12917-021-03020-7)
Supplement: Supplementary file 2 — Additional file 2: Table S2. Number of sequences and Sequencing depth generated by RNA sequencing. [file 12917_2021_3020_MOESM2_ESM.pdf]

Ovarian activation delays in peripubertal ewe lambs infected with *Haemonchus contortus* can be avoided by supplementing protein in their diets

Paula Suarez-Henriques, Camila de Miranda e Silva-Chaves, Ricardo Cardoso-Leite,

Danielle G. Gomes-Caldas, Luciana Morita-Katiki, Siu Mui-Tsai, Helder Louvandini

Additional file 2:

Table 2. Number of sequences and Sequencing depth generated by RNA sequencing

Sample/Animal Identification	Number of generated sequences	Sequencing depth(X)
A18	40.226.422	4,7
A35	48.116.408	5,6
B4	48.509.528	5,7
A32	56.919.770	6,7
A9	66.926.088	7,8
B3	38.146.966	4,5
A34	43.053.332	5,0
A28	61.182.596	7,2
B8	46.062.480	5,4
A19	56.405.208	6,6
A33	73.152.548	8,6
B24	49.642.828	5,8
B23	59.073.780	6,9
A20	44.524.258	5,2
A40	55.056.536	6,4
B21	41.889.154	4,9
C22	54.350.972	6,4
