# Supplementary material for: Ovarian activation delays in peripubertal ewe lambs infected with Haemonchus contortus can be avoided by supplementing protein in their diets
Source: BMC Vet Res. 2021 Nov 3;17:344. doi: 10.1186/s12917-021-03020-7 (PMC8565066; doi:10.1186/s12917-021-03020-7)
Supplement: Supplementary file 3 — Additional file 3: Table S3. Percentage of mapping to gene regions. [file 12917_2021_3020_MOESM3_ESM.pdf]

Ovarian activation delays in peripubertal ewe lambs infected with *Haemonchus contortus* can be avoided by supplementing protein in their diets

Paula Suarez-Henriques, Camila de Miranda e Silva-Chaves, Ricardo Cardoso-Leite,

Danielle G. Gomes-Caldas, Luciana Morita-Katiki, Siu Mui Tsai, Helder Louvandini

Additional file 3:

Table 3. Percentage of mapping to gene regions

Sample/Animal Identification	Mapped to gene regions (%)	Mapped to intergenic regions (%)	Messenger RNA (%)
A18	95,05	4,95	95
A35	95,19	4,81	93
B4	95,39	4,61	84
A32	94,63	5,37	92
A9	94,41	5,59	92
B3	94,93	5,07	94
A34	94,33	5,67	92
A28	95,02	4,98	89
B8	93,33	6,67	73
A19	96,39	3,61	76
A33	95,63	4,37	86
B24	95,08	4,92	91
B23	95,29	4,71	91
A20	94,58	5,42	80
A40	96,18	3,82	81
B21	94,95	5,05	86
C22	85,99	14,01	87
