# Supplementary material for: Ovarian activation delays in peripubertal ewe lambs infected with Haemonchus contortus can be avoided by supplementing protein in their diets
Source: BMC Vet Res. 2021 Nov 3;17:344. doi: 10.1186/s12917-021-03020-7 (PMC8565066; doi:10.1186/s12917-021-03020-7)
Supplement: Supplementary file 4 — Additional file 4. Full list of differentially expressed genes in supplemented not infected vs control not infected groups-converted. [file 12917_2021_3020_MOESM4_ESM.pdf]

Ovarian activation delays in peripubertal ewe lambs infected with *Haemonchus contortus* can be avoided by supplementing protein in their diets

Paula Suarez-Henriques, Camila de Miranda e Silva-Chaves, Ricardo Cardoso-Leite,

Danielle G. Gomes-Caldas, Luciana Morita-Katiki, Siu Mui-Tsai, Helder Louvandini

Additional file 4. Full list of differentially expressed genes in supplemented not infected vs control not infected groups-converted

Up-regulated FDR p-value < 0.05 **Down-regulated FDR p-value < 0.05**

Gene ID	Log fold change	Gene ID	Log fold change
LOC105605968	14.25886681	MED21	-15.01055874
CDC20B	13.72624852	HSPA1A	-14.65601812
MAGOH	13.51528356	LOC114111207	-13.42412678
LOC114113605	13.14575879	GCG	-13.09364735
LOC114108752	12.51281719	LOC114112996	-12.45120181
LOC114118015	12.27581472	RPS29	-12.44888099
ACTL7B	11.99575872	GBP6	-12.3578038
DNAH6	11.99575872	RIDA	-12.17049748
LOC101109601	11.60514207	LOC114117326	-12.13917253
LOC114116052	11.54102927	ADAMTS14	-11.90189342
ENTPD3	11.45799743	LOC101109111	-11.87448665
EFCAB12	11.3060418	XIRP1	-11.83237484
LOC114118377	11.30009449	LOC101110577	-11.79996416
ANKRD66	11.30009449	SCN3A	-11.74805417
MCIDAS	11.28812582	LOC101119648	-11.67447743
ECT2L	11.20144396	C3AR1	-11.61361803
LOC105615270	10.80768995	CNMD	-11.55871327
LOC114113190	10.78231654	LOC114115278	-11.49715051
LOC114112547	10.76514957	LOC101108817	-11.38993579
LOC114109598	10.75648886	LOC101107096	-11.36553514
LOC114112520	10.75648886	COL9A2	-11.23153813
LDLRAD1	10.75648886	LOC114108792	-11.23153813
LOC114116806	10.66689618	ZNF366	-11.21524451
SLC5A9	10.62944093	LOC101122330	-11.20977215
LOC114111483	10.49011483	LOC101120001	-11.20427895
LOC105604928	10.4690655	SELPLG	-8.554603657
LOC101123349	10.4690655	GPNMB	-8.148593027
LOC780488	10.4690655	DECR2	-7.755154941
DUPD1	10.4477045	MMP19	-7.674036089
DTHD1	10.4477045	LOC101102230	-7.648969975
LOC101111962	10.42602247	PROSER2	-7.304266087
FOXQ1	10.41505802	STAR	-7.119729038
LRCOL1	10.4040096	BMPER	-6.857730401
CFAP221	10.4040096	LOC114117782	-6.782199088
HOXA10	10.31243728	NAIP	-6.737841382
LOC114117808	10.31243728	IRF5	-6.578360303
MYT1	10.23972982	TFPI	-6.492951386
PLA2G4F	10.21465628	MSR1	-6.452887177
BICDL2	10.21465628	TPBGL	-6.42686971
CACNA2D3	10.18913924	RUNX1T1	-6.412435328
LOC114112897	10.13670995	ZIC2	-6.370458409
RNASE12	10.10976304	LY75	-6.327948992

CARD14	10.08230322	LOC114112166	-6.18430035
SNTN	10.05431058	GPRC5A	-6.040006826
SPATA17	10.02576403	RELN	-5.993371209
FOXN4	10.02576403	SLC11A1	-5.985889766
ATP5MC1_1	10.0112761	XPNPEP2	-5.896521877
NME5	9.966918373	AK5	-5.888116174
LOC114108763	9.966918373	REEP1	-5.859671089
CATSPER3	9.936570272	LOC105606356	-5.809317468
LOC105607844	9.905570035	LOC105611720	-5.792067032
TMEM233	9.841496648	LOC114113944	-5.704179958
LOC105612393	9.841496648	USF3	-5.61005422
LOC114110615	9.808360245	TNNI1	-5.558015832
LOC114109666	9.791502195	TLR7	-5.481856943
LOC114113124	9.774444824	LOC101109425	-5.447945088
MROH5	9.774444824	C5AR1	-5.443518415
LOC105603461	9.757183365	BTK	-5.415634452
LOC114114068	9.722028224	SDS	-5.397454073
WNT7B	9.667635167	FOXN2	-5.377632033
CA1_1	9.667635167	ITGAM	-5.369746584
CWH43	9.630199345	LOC114109090	-5.303715749
LOC101111513	9.591766181	RPP25	-5.21240301
LOC114112440	9.591766181	TLL1	-5.189420112
LOC105603182	9.552281077	SLA2	-5.185215751
DYDC1	9.552281077	LYZ	-5.185215751
C17H22orf15	9.552281077	LOC114108772	-5.160166275
DBX1	9.552281077	STS	-5.14625759
LOC114118076	9.532125738	PTPN22	-5.14625759
LOC105605457	9.511684823	LOC101103380	-5.090904486
CFAP97D2	9.490950121	MAPK4	-5.05388651
LOC114112979	9.469913066	ZNF527	-5.036073411
LOC114118065	9.469913066	SIGLEC1	-5.033342574
CRB3	9.469913066	CSF3R	-4.994790785
TBATA	9.448564708	MRC1	-4.994243213
LOC105608412	9.426895695	NOX4	-4.979046419
SPOCD1	9.426895695	LOC105608322	-4.97338822
LOC114108622	9.382556135	MSMB	-4.969365867
LOC114109078	9.382556135	RAB3B	-4.927442639
GJB7	9.382556135	LOC101120749	-4.878509306
MYBPHL	9.336810522	LOC101110128	-4.845443831
LOC114116364	9.336810522	TMEM252	-4.811602709
LOC114111507	9.336810522	KLHL40	-4.79347016
LOC105608303	9.289566753	ABCA1	-4.787823064
KCNK16	9.289566753	LOC105607837	-4.776948674
LHX1	9.240723368	LOC114111673	-4.757005852
LKAAEAR1	9.240723368	LOC114114429	-4.742155965

LOC114110465	9.240723368	LOC105602976	-4.741441707
LOC101120702	9.240723368	KLHL3	-4.674019197
LOC114108815	9.240723368	LOC105613870	-4.641733843
LCA5L	9.240723368	DMBT1	-4.605384359
LOC105616575	9.215667236	TREM2	-4.60481275
LOC114109372	9.215667236	LOC114114905	-4.601168071
LOC114113260	9.215667236	RNF43	-4.549481387
OVGP1	9.201561585	NOD2	-4.538097087
LOC114113633	9.190168236	P2RY13	-4.529953496
LOC114108816	9.190168236	S100A8	-4.507822284
LOC114114056	9.164210431	WDPCP	-4.494463898
LOC106990690	9.164210431	LOC105605834	-4.472142243
LOC114109401	9.137777008	MYCN	-4.472142243
LOC114115301	9.137777008	FABP4	-4.469559827
S100A5	9.137777008	NCF4	-4.454057944
C3H12orf56	9.137777008	SOLD1	-4.413022632
LOC114113187	9.137777008	ZC2HC1A	-4.39672262
RCVRN	9.137777008	WDR44	-4.383221462
LRRIQ1	9.137777008	KCNQ3	-4.379232843
LOC114108777	9.110850212	SYN1	-4.362874368
LOC114113912	9.083411273	JCHAIN	-4.361277457
LOC114115026	9.083411273	EVA1A	-4.330433578
LOC114111672	9.083411273	SALL1	-4.330433578
LOC105612394	9.083411273	LOC101118510	-4.328001472
PDIA2	9.083411273	KBTBD6	-4.302038072
LOC114117637	9.083411273	PTGFR	-4.295582565
KCNJ5	9.083411273	GH	-4.279312046
BHLHE23	9.055440333	LOC105605798	-4.27154491
TCTEX1D1	9.055440333	FMNL1	-4.258018415
LOC114114615	9.026916352	LOC101117971	-4.244155281
LOC114117014	8.997817021	RRP7A	-4.216426905
RPTN	8.997817021	LRCH2	-4.194935379
LOC114110563	8.968118649	TGFA	-4.190651977
ONECUT1	8.968118649	MCHR1	-4.173274165
LOC114116679	8.937796053	LOC101113965	-4.17019759
KSR2	8.906822429	LOC114111751	-4.145332157
LOC114112978	8.906822429	TLCD1	-4.14292166
AQP4	8.906822429	LOC105609290	-4.122532649
LOC101112856	8.906822429	KLK4	-4.122532649
LOC114111039	8.906822429	LOC114110969	-4.118052509
LOC105602628	8.906822429	CCRL2	-4.084108036
MNX1	8.906822429	LOC101113335	-4.066243607
LOC114110112	8.906822429	LOC101120392	-4.060364486
LOC114118721	8.906822429	ZBTB37	-4.058104487
LOC105612706	8.906822429	RSBN1	-4.050282569

LOC101108745	8.906822429	LOC114108775	-4.034814196
LOC105610401	8.906822429	CMTM4	-4.027815891
LOC114116068	8.875169206	ZFP69B	-4.007668876
C15H11orf97	8.875169206	LOC105608656	-4.007668876
CLDN8	8.875169206	LOC114114497	-4.007668876
PSMA8	8.842805891	LOC114108628	-4.004272641
SLC6A12	8.842805891	THNSL2	-4.000761905
DNMT3L	8.842805891	ACOX2	-3.99687774
LOC114114804	8.842805891	GDAP1L1	-3.99687774
LOC105602343	8.842805891	CELSR3	-3.99687774
GJA9	8.842805891	AHR	-3.987938368
ZAN	8.842805891	RPS27	-3.982600659
TMPRSS2	8.842805891	CREB3L3	-3.968996963
C22H10orf90	8.842805891	KLHL28	-3.966142425
RPL23A	8.842805891	LOC101120993	-3.965261564
LOC106990291	8.809699893	GABRB2	-3.962004278
LOC114114595	8.809699893	ABTB2	-3.958688763
LOC114114910	8.809699893	MEX3B	-3.952658444
C1QTNF12	8.809699893	CD53	-3.944887479
LOC114113734	8.775816323	LOC101117577	-3.943128714
LOC105611240	8.775816323	NDP	-3.942296075
CA1_2	8.775816323	TACR3	-3.908795474
LOC101120200	8.775816323	CTSL	-3.901315151
TM4SF4	8.775816323	NSL1	-3.896789236
LOC105605025	8.775816323	RUNDC3A	-3.882862638
GAP43	8.775816323	ADAM22	-3.882862638
DPF1	8.775816323	IRX5	-3.876646489
TRIM17	8.775816323	KPNA5	-3.872699821
GPR31	8.775816323	ZNF790	-3.86603038
DGKI	8.775816323	PTER	-3.85703923
LRGUK	8.775816323	LOC114115621	-3.853059213
LOC105610367	8.741117774	LOC101109989	-3.844843375
LOC114118296	8.741117774	GFRA1	-3.830129947
PROZ	8.741117774	PTPRH	-3.816162361
NUPR2	8.705564074	ITGAV	-3.810208105
REG4	8.705564074	SLC37A2	-3.809902118
VEGFD	8.705564074	LOC114108841	-3.806292746
LOC105602450	8.705564074	B3GALT1	-3.800598137
LOC114109675	8.542437228	SCN1B	-3.799393939
LOC101113807	8.530196643	MRAP2	-3.798424221
LOC105604598	8.226121556	TLR2	-3.770717302
PAGE4	8.035681874	LOC114113914	-3.759466431
LUZP6	7.977650566	RRP12	-3.755513468
RSPH6A	7.510857722	LOC106990122	-3.746228206
C1H1orf194	7.487891548	ITIH3	-3.746228206

TFF3	7.448355197	LOC105616822	-3.730708676
LOC114113045	7.20196945	FHL5	-3.723731224
LOC114115623	7.175095682	LOC101112891	-3.722371601
FAM166B	7.140763132	ADCYAP1R1	-3.722121114
MAP3K19	7.039945237	LOC101104297	-3.722121114
LOC101122934	6.989527188	PPFIA3	-3.713571837
ADGB	6.989527188	ARHGAP20	-3.712730046
EFCAB6	6.925035641	LOC101116024	-3.706967383
LOC101114620	6.873656627	PDK4	-3.705692361
LOC105606686	6.864745397	RASGRP3	-3.686988829
MYT1L	6.810033011	MPEG1	-3.683037809
WDR78	6.712751532	LOC114118858	-3.672730544
C3H2orf50	6.629849056	LOC105613472	-3.672730544
LOC114110313	6.610750348	N4BP2	-3.647895523
CFAP299	6.581619691	SLC18A2	-3.643758433
LOC101119591	6.568700272	LRIF1	-3.643244062
LOC105603394	6.498870768	BROX	-3.634914939
IQCA1	6.216370692	SLC25A34	-3.632520353
NGB	6.215404086	LOC100101238	-3.626657221
WDR63	6.19112234	FCGR2B	-3.611258729
LOC105602076	6.171818016	RETN	-3.608453001
CDHR3	6.144707521	MAN1A1	-3.606158897
TRPM5	6.128588512	TNFRSF9	-3.595286626
OSCAR	6.110171171	LOC100125610	-3.595286626
C2H9orf24	6.06878292	LGR6	-3.595286626
TTC21A	6.058781121	PDE11A	-3.595286626
LOC114114071	6.050698285	FAM241A	-3.594298957
LOC105605002	6.050698285	LOC105603904	-3.583164521
LOC105609771	6.050698285	CHMP2B	-3.572647277
LOC105610032	6.026172159	MMP9	-3.569470687
TGM3	6.021763315	MYOC	-3.568060818
LMAN1L	6.005341119	SAMD10	-3.562249955
LOC101118856	5.99633255	GPR3	-3.562249955
LOC101121644	5.958984533	CNTN1	-3.554603642
AQP5	5.918376882	LOC101108321	-3.546847079
LOC114109326	5.868918021	LIPA	-3.545270663
LOC114111520	5.819743705	LOC114117228	-3.544449171
RSPH4A	5.808260308	ZNF300	-3.544449171
PRSS36	5.80271385	VSIG4	-3.525289878
SLC45A2	5.80271385	LOC114116375	-3.513448521
EFCAB1	5.800601932	LOC114116393	-3.513448521
LOC105615227	5.788090482	ELANE	-3.493516398
LOC114113984	5.788090482	BCL2L11	-3.475140266
TEKT4	5.772284599	FOXF2	-3.475139397
LOC105604357	5.755727168	KMO	-3.475139397

SLC7A4	5.749628563	LOC114115339	-3.461462016
C15H11orf52	5.740121002	CD6	-3.454439062
LOC101109935	5.717879119	LOC101111006	-3.452164519
CERKL	5.694515088	TYROBP	-3.449300141
LOC114108680	5.668043295	RHOJ	-3.444740142
LOC101104423	5.668043295	MMRN1	-3.444361972
LRRTM1	5.66048424	LCP2	-3.439705455
C15H11orf88	5.629431274	SEMA3D	-3.436477558
CCDC17	5.622724154	C1H1orf109	-3.432195493
CAPS2	5.61151887	EPM2A	-3.431803543
SOX3	5.610720137	DLGAP1	-3.431544333
LOC105610279	5.58203328	MPPED1	-3.426687419
CACNG2	5.544636261	FPGT	-3.426200077
MUC3A	5.515290487	PPFIA4	-3.422382308
LOC114116353	5.506243995	KCNJ3	-3.419844139
LOC114117882	5.471591992	LOC114110611	-3.410490806
LOC101103021	5.470321195	PTAFR	-3.400040597
LOC114113194	5.466802056	LOC101112936	-3.399954853
LOC114115006	5.466802056	WASF1	-3.395843477
LOC114117611	5.466802056	CYBB	-3.387801066
LRRC56	5.464093938	LOC101123419	-3.387330275
ANKRD65	5.45812593	FGFR4	-3.382429143
CRYBB3	5.439584293	LOC114117779	-3.382429143
LRRC18	5.427880633	ADAMTS13	-3.380205833
LRRC43	5.427880633	LOC114114600	-3.359778281
LOC106991586	5.427303824	TIMP1	-3.358570606
ESRP2	5.397397466	ADAMTS5	-3.345895805
SPAG17	5.383531894	SLC25A21	-3.333404393
LOC114112055	5.382964264	CCDC171	-3.333156664
LOC114110464	5.382106	MAF	-3.333067947
DRC7	5.367585815	LOC114108991	-3.324618157
LOC101105995	5.360272765	DOCK2	-3.320193688
LOC105606717	5.341561838	CEMIP	-3.317899208
PRG4	5.333999951	TBC1D10C	-3.30792944
TCTEX1D4	5.33302684	LOC114111048	-3.307207657
CFAP43	5.321850892	CCN3_2	-3.302844225
CATIP	5.308352144	LOC101113476	-3.299676505
LOC114116060	5.297276703	LOC114117936	-3.297104253
LOC106990158	5.297276703	LOC105612575	-3.295983448
GUCA1A	5.265759883	PTPRE	-3.294173375
MYO15B	5.260243865	AKAP7	-3.291717311
LOC114114538	5.251589019	CHRM3	-3.283349507
LOC105609234	5.241131497	PLCE1	-3.265862963
LOC101108034	5.241131497	LOC101114790	-3.260615997
VWA2	5.222434449	C1QTNF9	-3.249660309

GRM5	5.204407034	LOC114108746	-3.246335574
LOC114110461	5.202702993	GAB3	-3.245248468
APOBEC4	5.184958616	LOC114116177	-3.239447146
FAM81B	5.16278929	RIPOR3	-3.235745146
INHBA	5.149206158	LOC114116635	-3.235745146
CCDC42	5.140187553	SP5	-3.235745146
LOC105604131	5.138185137	KRT27	-3.235745146
TTC25	5.124079617	LOC105614852	-3.226879353
LOC114117875	5.113463129	ZDHHC2	-3.226196911
P2RX2	5.10739343	TMEM86A	-3.213142942
LOC114118765	5.107017449	C1S	-3.208789811
LOC105606929	5.105145296	C7	-3.203884955
FXYD3	5.100535565	RAB3C	-3.201730731
CFAP100	5.081169264	LOC105603377	-3.201277893
DCDC1	5.071989016	LOC105602567	-3.201277893
C8H6orf118	5.071989016	IL6ST	-3.196386682
CFAP45	5.063404814	FTSJ1	-3.184722276
LOC105614840	5.058367299	TDRD6	-3.17696032
CFAP73	5.044905648	FOXD2	-3.17696032
STK33	5.03299016	LOC114116396	-3.17696032
ADIG	5.027324481	PAH	-3.17696032
CCDC180	5.009805538	SLC6A15	-3.17696032
LRRC23	5.007964206	SSTR2	-3.17696032
PPP1R32	5.004661832	SLC38A4	-3.172093866
LOC105607009	4.99822515	LOC101119706	-3.161601417
DNAI1	4.969962981	PLCB2	-3.156099183
LOC105614430	4.945929421	SYT6	-3.143544414
VSIG8	4.945929421	LOC114116078	-3.13213656
TAT	4.942139516	GALNT15	-3.121807212
LOC106991412	4.907230557	LOC114113956	-3.120671278
LOC114109042	4.907230557	LAIR1	-3.118447086
VRTN	4.907230557	LTB4R	-3.112733993
CCDC113	4.902629158	HS2ST1	-3.105943121
CFAP161	4.897045678	LOC114112944	-3.100774589
LOC114116892	4.888678222	COL4A5	-3.094922452
PAX2	4.875577334	NCEH1	-3.094490965
CFAP52	4.872711379	PTPRJ	-3.086878764
ANKLE1	4.871248567	MS4A7	-3.077616346
SLC27A2	4.871248567	KLHL24	-3.067319562
ARMC3	4.860600277	LOC105602033	-3.066781734
LOC114115315	4.854585231	CD2	-3.066730268
TMC5	4.854585231	LOC101123376	-3.063027277
CHRNB2	4.84321402	IGSF9B	-3.062096547
CFAP44	4.841965291	LOC101115729	-3.055572102
IL17RE	4.83772718	AGT	-3.053665985

SEMA3E	4.82066981	CHMP4C	-3.051165713
CCDC78	4.815398165	PMEL	-3.045407373
CCDC13	4.812576275	SLC22A3	-3.033384526
GJB1	4.785937859	SERTM1	-3.030591753
GRHL2	4.78076174	LOC114114581	-3.030591753
LOC114117907	4.776224452	LOC114109613	-3.029798799
LOC105603273	4.776224452	CPAMD8	-3.024881469
FAM183A	4.775522384	CD68	-3.024007274
CCDC81	4.762620476	ITGB2	-3.015802775
FRMPD2	4.735394715	YOD1	-3.014487318
LOC105607801	4.732219978	DOK7	-3.001042617
NXNL2	4.730278702	CX3CR1	-2.998680274
LOC105602963	4.727520437	VGLL3	-2.997817596
DNAI2	4.721761639	LOC106991487	-2.996763573
GAS2L2	4.71584751	THAP8	-2.993297894
SLCO5A1	4.715493827	HOXB3	-2.993265292
LOC105608334	4.705972202	BEAN1	-2.993265292
VWA3A	4.701966741	RUNX2	-2.99081375
XKR5	4.691476693	SYN2	-2.990595001
LOC114109708	4.691476693	MUSTN1	-2.989548087
LOC114116813	4.691476693	RGS5	-2.983945204
MUC4	4.676424331	LOC105602040	-2.973989234
LOC114115610	4.676424331	PDE1B	-2.967288398
IL20RB	4.65744553	OLFM1	-2.962706113
LOC105610999	4.657335727	LOC114111314	-2.960940705
C6H4orf54	4.653073383	FOXC2	-2.956660449
LOC105611998	4.64384697	TMEM245	-2.95400836
BHLHE22	4.637991167	SMPD3	-2.952576685
LOC101122904	4.637991167	LOC101115115	-2.947159778
LRRC74B	4.633929937	BBC3	-2.947103079
TCTE1	4.633091046	B3GNT7	-2.940381396
TEKT1	4.632950694	PLIN1	-2.937290309
CTXND1	4.62881204	LOC100037664	-2.927875231
LOC105602434	4.621360988	ELMO1	-2.9259448
LOC105606428	4.621360988	SYK	-2.92267846
GAST	4.621360988	CPM	-2.922236105
PLA2G3	4.621360988	LOC114112017	-2.915849381
LOC105605424	4.621360988	IFI30	-2.915817534
LOC105607291	4.618383694	MYO1F	-2.91004911
KIF19	4.618204165	ATP7A	-2.900159477
LOC105603166	4.610038394	PTPRC	-2.899696024
DNAJB13	4.607388958	TLR8	-2.898358503
CCDC116	4.598506062	SOX10	-2.896966542
LOC114108793	4.594841231	PDE4C	-2.894570105
BPIFB1	4.582058671	LOC105612882	-2.889230639

CFAP57	4.58009785	STUM	-2.8870551
LOC106990463	4.576863088	NRIP3	-2.886459598
CFAP74	4.568758674	CLDN1	-2.880648854
TACR1	4.561296229	ADGRG6	-2.880572844
LOC114110280	4.557909808	KCNN2	-2.878933247
LOC114116442	4.557909808	ZNF577	-2.878933247
HOXC13	4.554288908	LOC101113636	-2.877318595
HMGN5	4.543776445	NPAS1	-2.870590032
ZNF750	4.53183783	PLP1	-2.868970105
LOC101121777	4.527583703	LIFR	-2.866865289
ERICH3	4.506020379	RRP9	-2.863529823
CACNA1E	4.502627951	LOC101118184	-2.863174984
FAM92B	4.491864324	TRPM2	-2.859434985
LOC101122504	4.48186449	MITF	-2.856503862
LOC114113048	4.472744314	LOC101102414	-2.855597177
STX19	4.472014831	SATB2	-2.855525042
RSPH14	4.458009449	LOC105608413	-2.851608435
LOC114110984	4.453877462	LOC114114838	-2.850619487
CFAP99	4.453258399	ADORA3	-2.845228334
SPDEF	4.444413335	ADRA1D	-2.843457701
LOC114112701	4.438962731	LOC101117706	-2.841638168
IQUB	4.428688041	LOC105602566	-2.832893623
PIFO	4.428568796	ITGB8	-2.831730757
SPATA18	4.419278836	MIGA1	-2.826368834
MAMDC4	4.413434192	KCTD21	-2.825501822
LOC101116286	4.407739498	CHRNA7	-2.824791609
DRC3	4.407683197	GPR34	-2.823506031
PIH1D3	4.394054861	CCN2	-2.819192977
TTLL9	4.385156909	SELENOP	-2.80640004
LPO	4.384763555	KCTD12	-2.805663048
TFF2	4.383035508	NR1I2	-2.800655877
LOC114113983	4.382961603	LOC114114138	-2.800655877
ZMYND10	4.377214772	LOC114116372	-2.798919886
LOC114115412	4.352650391	MARCH1	-2.798825004
LOC105607030	4.350752938	VAV3	-2.796070789
VWA3B	4.347437355	ADCY7	-2.795037413
DAW1	4.345974973	ZCCHC10	-2.794185432
RIBC2	4.344183772	ABCA10	-2.782735152
NECTIN4	4.343489382	LONRF3	-2.782130611
LOC105605961	4.339075871	LNPEP	-2.776689101
LOC114109368	4.339075871	YAF2	-2.774976084
EHF	4.338212542	MEGF10	-2.772355415
LOC105604792	4.333477498	TNFSF10	-2.767700827
SMIM5	4.324522932	SMAD9	-2.765270106
CCDC89	4.323871247	C17H4orf33	-2.764161039

DCDC2B	4.318950337	LOC105607815	-2.761808058
AK7	4.312048515	CD247	-2.759902742
LOC101123537	4.311641173	SLC41A2	-2.758253527
LOC114116627	4.31157674	CLEC7A_1	-2.752864229
CCDC103	4.31142734	AMMECR1	-2.751931055
CCDC65	4.309173646	SVIP	-2.748608868
LOC106991901	4.308314678	SPPL2A	-2.744918121
LOC114118022	4.300868566	GATA5	-2.74022776
LOC105604932	4.300868566	IL16	-2.739238087
PIWIL2	4.296938387	PHF21B	-2.738380474
SLC34A3	4.291893886	CALCRL	-2.736634789
C11H17orf97	4.289680631	MANEAL	-2.736248445
LOC105602901	4.288633853	ANKRD44	-2.734903016
SLC6A11	4.285224166	TMEM154	-2.734602552
NME9	4.280615577	INPP5D	-2.728113019
ODF3B	4.275914747	LOC105614569	-2.727158215
LOC105602212	4.273670704	LOC105602313	-2.727158215
RARRES1	4.268988338	GEM	-2.726658955
STAT4	4.268563725	CDA	-2.72419095
ACSM3	4.263363553	ADAP2	-2.71976656
NKX6-2	4.261346771	LOC101116121	-2.718911916
LOC105606593	4.255631893	SAT1	-2.708681552
CLDN7	4.246737574	NPTX2	-2.706408233
ADAMTS8	4.243116537	LOC114109018	-2.70110439
GCNT3	4.2215676	LOC114116929	-2.70110439
SLC22A4	4.219687845	ARRDC3	-2.695918926
MORN5	4.219011097	CHRM2	-2.69073469
LOC105609355	4.208237402	PLIN2	-2.688046579
LOC105611654	4.208237402	TRIM59	-2.682643796
TEKT2	4.206491911	COBLL1	-2.681752546
PAK6	4.206400815	LOC106990520	-2.680083158
LOC114111341	4.205602167	NHLRC3	-2.679582159
TSNAXIP1	4.195939887	LTBP2	-2.673247245
LOC105609336	4.195422587	ADGRD1	-2.671443365
LOC114110288	4.192632148	RUNDC3B	-2.669203214
LOC105606955	4.192632148	GPM6A	-2.666256744
CLDN23	4.192632148	SVEP1	-2.665544275
LOC105612984	4.192632148	ATAD2B	-2.660869336
LOC114118734	4.192632148	PPM1L	-2.658540028
LOC101111810	4.192632148	ARL5B	-2.65744181
NKPD1	4.192632148	IMPA2	-2.65448811
PLEKHD1	4.191358214	SKIL	-2.654370473
LOC114112985	4.186945736	RNF11	-2.653934548
ANKRD40CL	4.179453908	HGF	-2.653826154
LOC106990170	4.179453908	CD302	-2.650726463

LOC105611754	4.166771933	LOC101108419	-2.649714297
LOC105603051	4.162858912	ACSM5	-2.649714297
FOXJ1	4.147708294	LOC105604626	-2.649714297
ROPN1L	4.146573347	IL1B	-2.649714297
LGALS2	4.144499087	AP1AR	-2.648407656
LOC105607806	4.140316892	ACP5	-2.647608855
C3H2orf81	4.137588115	CUL5	-2.644770493
LOC105609280	4.135619874	SEMA3C	-2.642918659
LOC114113700	4.134157747	CTSK	-2.625156108
CRHR1	4.129636259	PLSCR4	-2.62101289
LOC114117807	4.129636259	CXHXorf21	-2.620781838
LOC101116972	4.129636259	RAP2C	-2.616011544
LOC105607437	4.122076844	NR4A2	-2.615902912
LOC114118293	4.116193627	LOC114114084	-2.615720931
MYH14	4.116179661	LOC114114566	-2.615561623
LOC114116934	4.11335236	LOC114112946	-2.612872897
LOC105604300	4.109248094	LOC101114469	-2.612561791
LOC114117588	4.109248094	ABCA5	-2.611975341
SLC10A1	4.109248094	IGFBP1	-2.610606445
CFAP157	4.109028009	ASB5	-2.605466402
CCDC181	4.103428388	LIN54	-2.604958722
VILL	4.101005323	TMEM81	-2.601640693
RSPH1	4.095439911	LOC100192427	-2.599269285
LOC114114546	4.086032958	IL21R	-2.597830071
LIME1	4.086032958	LOC101117613	-2.597386611
LOC114115565	4.086032958	LRP12	-2.594796445
PSPN	4.086032958	FOSL1	-2.590253683
LOC114114841	4.086032958	LOC105611547	-2.590253683
LOC114110411	4.086032958	SLC24A1	-2.590253683
HPD	4.086032958	LOC105616533	-2.589058847
LOC114112676	4.084827563	PHIP	-2.587684097
LOC114112981	4.070746381	LOC101108354	-2.585171458
LOC114114093	4.068630101	ELOVL7	-2.583686167
LOC114114047	4.064430339	LOC105605916	-2.580757002
NOXO1	4.062830516	LOC114116957	-2.580309193
CCDC151	4.060949702	DOK2	-2.578376825
RAB25	4.058220805	TAF1A	-2.578105456
LOC114112252	4.057061252	ARID5B	-2.574215798
C1H21orf58	4.050488279	PIGM	-2.574213649
LOC106991069	4.03915068	CC2D2A	-2.573490561
MSMP	4.03915068	EVX1	-2.569637258
CAPSL	4.03119598	PPP1R2	-2.569618462
GGT6	4.029144997	LOC114118731	-2.569336178
DUOX1	4.015369761	GPR132	-2.564359056
LOC114112251	4.011302466	LOC114109663	-2.558657548

HPDL	4.004607151	SLC4A7	-2.556859551
LOC101112481	4.002963056	APLP1	-2.555829294
TEX52	3.999708741	LOC105615131	-2.551630278
LOC105608013	3.996385698	EPB41L3	-2.54916534
DNAH12	3.996192365	SPOPL	-2.548573785
CCDC39	3.987540732	CILP	-2.548459476
FFAR4	3.986270518	PHACTR2	-2.546107216
KLF8	3.970924337	LOC114114521	-2.544070005
LOC105607861	3.970924337	C9orf72	-2.543860546
LOC114117610	3.970924337	DKK2	-2.540522268
SLC5A11	3.970924337	FRMD4B	-2.537925038
LOC106991494	3.970924337	ZBTB26	-2.537461746
LOC105605072	3.970924337	FOS	-2.534196788
DNAH2	3.96783277	ARHGAP5	-2.534080708
LOC114114900	3.967814041	CCL21	-2.532006001
FRK	3.967158377	CDK5R2	-2.528984167
SLC28A3	3.964714519	SLC46A3	-2.526293676
DRC1	3.962826461	MYRIP	-2.524228929
PLEKHG7	3.959507354	SHISAL1	-2.523800106
LOC105608127	3.953047414	SGSH	-2.522167351
LOC114114495	3.953047414	PABPC5	-2.520906621
LRRC10B	3.95053077	LOC114116164	-2.516612715
LOC114112973	3.950377945	SPOCK2	-2.515171667
LOC105602881	3.946776986	ENPP6	-2.514569385
PPIL6	3.940340421	LOC114113841	-2.514354349
LOC114116771	3.937925842	RALGAPA2	-2.513436563
SFN	3.931074774	SAMHD1	-2.512986269
LOC114118893	3.930764395	HNF1A	-2.503143126
LIF	3.930133915	LOC114108712	-2.50124884
ESRRB	3.926595383	MMP2	-2.499895629
C19H3orf49	3.919811597	LOC114117625	-2.49754411
LOC101111846	3.909732088	LOC114118036	-2.496465308
ND3	3.907823912	RAPGEF6	-2.496190622
PKP3	3.905929495	LOC101112822	-2.495871676
ALS2CR12	3.903759616	NLRC4	-2.49323634
LOC114117845	3.903759616	LOC105611558	-2.492341573
ROPN1	3.898989964	SLC35E4	-2.49072403
LOC106991594	3.889030877	JPH1	-2.490351366
SCNN1G	3.889030877	EXD2	-2.488905471
TRPM8	3.888220684	CTSH	-2.488041021
CORO2A	3.880718038	IGIP	-2.487790242
LRRC46	3.879594041	IFIT5	-2.483962888
CCKBR	3.8714145	CSRP3	-2.48111509
KISS1	3.859420055	AMER2	-2.48111509
RTP1	3.856368967	LOC114110455	-2.48111509

LOC105609072	3.845828947	MEX3A	-2.478566412
KRT1	3.845828947	CTSS	-2.476785322
LOC114114054	3.845828947	ERRFI1	-2.475270768
CFAP46	3.839453076	SGCA	-2.471556795
FAM83A	3.839157133	FAM13C	-2.469105273
LOC101106237	3.833502845	LOC114112146	-2.466796482
LOC105607350	3.830183388	SHISA6	-2.466796482
LOC105606111	3.830183388	DR1	-2.462885738
LOC105606592	3.830183388	LOC114118468	-2.462192557
HMGCS2	3.817048765	LOC101118514	-2.462112372
LOC114113227	3.811308936	SPATA9	-2.458301405
XCL1	3.806812678	VANGL2	-2.457956242
RNF182	3.800049096	LAT	-2.456807782
CCDC40	3.793888578	RASGRP1	-2.453392597
LOC114115742	3.786380546	LAPTM5	-2.450905525
SIX3	3.778963545	KCNF1	-2.449843941
TDO2	3.778963545	KLHL4	-2.447535529
EXOC3L4	3.778266129	MAFB	-2.444579052
AGR2	3.778184057	FRMD3	-2.441284223
CDHR4	3.777071429	LOC114112948	-2.440876334
CORIN	3.76823068	ANKRD34A	-2.439863009
LOC101110964	3.766430673	ENPP1	-2.438868677
FAM3B	3.765492204	ZNF467	-2.438257365
LOC105604473	3.763557773	RIN2	-2.431263109
RGS11	3.753435784	UBE2D1	-2.429084837
LOC114115631	3.751789059	MAP3K1	-2.428972103
LOC114111030	3.751789059	PLCD3	-2.426089012
LOC101104012	3.744481215	ZFP36L2	-2.425428664
LOC114118755	3.741608213	NUDT13	-2.425145332
MFSD6L	3.737330288	PKLR	-2.421444948
NPVF	3.737313719	LOC114110805	-2.417429958
CCDC33	3.734612437	LOC114112253	-2.417055671
ADAD2	3.727312854	SOD3	-2.416765216
GALNT14	3.7250214	RFX7	-2.415175866
CAPS	3.723199151	LOC101120466	-2.414941776
KCNRG	3.721481635	SHISAL2B	-2.414284099
LOC101108147	3.717587288	LOC114112702	-2.412689448
TSPAN1	3.716171857	GPR146	-2.411014179
FOXA2	3.715312116	LOC114113616	-2.410432872
KLC3	3.713815226	KCNN4	-2.410432872
LHB	3.712463365	STX1A	-2.410432872
FAM81A	3.710372531	GLCCI1	-2.405282047
AQP10	3.70884784	LOC101106326	-2.404332665
CD226	3.70884784	ACVR2A	-2.399148847
LOC105610817	3.70884784	LOC106991803	-2.395231474

SLC44A4	3.708475445	GATA2	-2.394952847
FBXO36	3.707292195	LOC105608665	-2.390516341
CELA1	3.702031007	C8G	-2.390319928
LOC114114502	3.70175614	LOC101118972	-2.388800578
LOC114113031	3.699890211	LOC114112092	-2.388800578
LOC101105114	3.695545625	BICC1	-2.387070567
LOC105609379	3.694448767	CEP85L	-2.386178637
FBXO15	3.690909907	LOC105612071	-2.385434891
EPS8L1	3.689211523	PDRG1	-2.383131716
NEK5	3.685817769	DIO1	-2.382567139
RIBC1	3.685150606	PLEKHA1	-2.380606368
SLC6A2	3.67793997	TMEM65	-2.379621855
C7H15orf65	3.67793997	WDR36	-2.379435333
MARCH10	3.676841727	SPI1	-2.377225623
LOC101103644	3.674603342	RBM24	-2.374107194
DSCAML1	3.673655517	RGS14	-2.369276157
LOC654331	3.672555788	PPP1R16B	-2.369024667
WFDC2	3.668925242	RILP	-2.367578302
LOC101113126	3.667032156	LOC101123672	-2.366863222
DNALI1	3.665077821	CLDN11	-2.361450748
MORN3	3.661137058	RASSF8	-2.360825608
PLA2G4D	3.658072134	DENND5B	-2.358709812
SPATC1L	3.658072134	EFCAB9	-2.355907518
C15H11orf16	3.657991575	GPX3	-2.354766998
LOC101111401	3.655798669	KDM1B	-2.351441692
KIF9	3.643776849	TMPRSS13	-2.349657092
CYP19	3.641849574	LAT2	-2.348479237
LOC114113171	3.641849574	ARL16	-2.347669573
TMEM74B	3.641516352	DACH1	-2.347213663
LOC105615521	3.641516352	C5H5orf24	-2.344565673
LOC114114859	3.641074352	CD1E	-2.340604514
LOC114116883	3.639547704	TMOD2	-2.339269454
SPATA6L	3.638051981	LOC114112675	-2.338247878
LOC114117258	3.631642711	ZSWIM1	-2.338168571
KRT7	3.631480643	ASPN	-2.337140668
EDARADD	3.629345866	KLHL29	-2.336854215
LOC114113167	3.627095126	UBXN2A	-2.331801006
SLC9A2	3.626391117	SIRPB2	-2.327579877
PDZD4	3.625801071	RBM41	-2.326800389
FOLR1	3.621971943	LOC101113531	-2.325762137
ZNF875	3.614640929	NFAM1	-2.32531474
LOC101118751	3.608806697	HNF4A	-2.322137817
BARX2	3.601816604	ADM5	-2.318729229
SAXO2	3.599853889	C18H14orf28	-2.317382179
AP1M2	3.596026798	SERPINE1	-2.316828224

LOC101109268	3.592429959	KCNQ5	-2.316703186
LOC114111379	3.587714132	TUSC1	-2.314927025
CCDC173	3.58605073	PTGDS	-2.312395048
UBXN10	3.585052923	CCN4	-2.312137153
LOC101116843	3.583820527	DRAM1	-2.31207806
CDHR5	3.572342231	SLC38A7	-2.31156588
LOC114117236	3.570032069	CAPN3	-2.311353235
MYO5B	3.565045502	GRIN3A	-2.311353235
CCDC60	3.564726129	KIF26A	-2.306774293
KIAA2012	3.561413333	SEMA6A	-2.305562007
LOC105606524	3.560275193	FBXL3	-2.30534498
LOC114116067	3.560275193	PGM2L1	-2.304530475
FOXH1	3.557483	RPS15A	-2.304259205
ATOH1	3.557483	LOC114117597	-2.304236156
LOC114113666	3.557483	GPR75	-2.303383309
LOC114114513	3.557483	SMNDC1	-2.302921545
FGFBP1	3.557483	FNIP1	-2.301986429
IGSF1	3.557483	BACH1	-2.301724409
LOC101107613	3.557483	STK26	-2.301231894
TTC16	3.550634007	LOC101122744	-2.297093894
TNFSF9	3.546946495	C1H21orf91	-2.297078188
FANK1	3.544582218	LCK	-2.294219469
LOC114117572	3.544004294	LOC114113037	-2.29229129
PDZK1IP1	3.540189981	LMO3	-2.291182899
CPS1	3.539603932	SP4	-2.290745184
KLK12	3.53688581	SPN	-2.289997578
LOC114118264	3.531452792	LOC105602107	-2.289522669
LOC101121190	3.531452792	FAM102B	-2.287599159
CACNG4	3.524821576	RPS6KL1	-2.285847192
LOC114109081	3.52229587	LOC114108739	-2.28268001
LOC105609992	3.517831688	PRICKLE1	-2.281361163
LOC105615960	3.517831688	NCKAP1L	-2.280001437
LOC105609925	3.517831688	GPM6B	-2.279186919
FST	3.514065014	SLC9A9	-2.278843802
MORN2	3.512626219	LOC101122752	-2.278527477
HOXA5	3.503326029	SLAIN1	-2.278178603
CCDC153	3.500704171	FAM171B	-2.277001408
LOC114117601	3.499683584	LOC114109003	-2.27337942
HYDIN	3.494839959	RASSF3	-2.271014534
OSCP1	3.48950344	PPTC7	-2.268689524
S100B	3.480596308	SNTB2	-2.268446138
AK9	3.480225231	MAFF	-2.26806362
LOC114112870	3.476363196	SMAD1	-2.265660346
LOC101111106	3.474680941	WASF3	-2.263423249
LOC101113054	3.474382544	OLFML2B	-2.262538006

RAB36	3.468122157	RASSF5	-2.262390244
TRPV6	3.465343047	KCTD6	-2.259494983
WDR27	3.45955429	CLIP3	-2.259306482
LOC114111377	3.455119747	TNFRSF1B	-2.25291048
PRR15L	3.447881311	STX10	-2.25273972
CFAP70	3.447541996	SUSD2	-2.25217632
TACSTD2	3.445767679	KCNT1	-2.250031666
REC114	3.440518375	LOC105608150	-2.248367292
TTC29	3.435673531	NECAB2	-2.248367292
LOC105603181	3.435645376	SCAMP5	-2.247622287
SCN7A	3.430580412	MYCT1	-2.247585033
DMRT2	3.430580412	LOC101122501	-2.246324158
KCNC1	3.430580412	LOC114116830	-2.246182378
IQCH	3.429555502	TXNDC16	-2.245814194
OPRL1	3.424674923	PTX3	-2.24574896
PDE1C	3.423830752	ANO4	-2.245669622
LOC114113201	3.420137266	LOC105609191	-2.239587998
FAM216B	3.417384721	PGAP1	-2.239451508
ATP6V1B1	3.41620409	TFPI2	-2.238320686
LOC101110741	3.404727789	FOLR2	-2.238034057
TTLL6	3.403084128	PI4K2B	-2.237677425
LOC114115326	3.402749495	PLAUR	-2.237261328
S100A6	3.402749495	WIPF1	-2.236662666
RASSF6	3.399151873	PGFS	-2.235048413
PRR29	3.395562721	FAM189A1	-2.234302002
CCDC146	3.395514521	LOC114114990	-2.234302002
LOC105607442	3.39400579	LOC106990184	-2.234302002
DNAH5	3.39036723	ADD2	-2.228413751
EPCAM	3.383903168	LOC114116930	-2.226753896
SLC24A5	3.383686349	ROR1	-2.224854563
LOC105602163	3.383686349	FBXO32	-2.223160595
LOC105611263	3.383072881	LOC100527962	-2.223082727
CENPT	3.372120402	GNB4	-2.220951228
CCNO	3.371164244	NHSL2	-2.220616971
LOC105607560	3.367999725	C1QTNF3	-2.219932827
LOC114112724	3.365154831	FAM129C	-2.219022849
DNAAF3	3.362545865	DPY19L4	-2.218442201
LOC101120838	3.355151921	EDEM3	-2.218257665
LOC105612662	3.35096161	GOLIM4	-2.216009048
MISP	3.347149156	LOC114115236	-2.215486271
RSPO1	3.346709875	GIMD1	-2.215486271
LRRC31	3.346709875	RTN4RL1	-2.21505756
LOC114108779	3.346709875	COL28A1	-2.214804213
TTC34	3.344113993	THBD	-2.214515695
NYX	3.34237149	LOC114117973	-2.211964345

CA9	3.338991769	TRIM2	-2.211547254
ZNF232	3.337710743	CTSD	-2.209575229
LOC105604203	3.337710743	ELL2	-2.209551258
LOC105604773	3.337710743	SLC25A24	-2.209039715
DNAH11	3.335686124	CHN2	-2.207641436
LOC105605978	3.33504262	ITIH4	-2.206088315
SAXO1	3.334018611	TMEM123	-2.200852029
CNTD2	3.333396867	ATP12A	-2.196542659
AZGP1	3.330688419	GPR63	-2.195468085
GRHL3	3.326821358	LOC114116832	-2.195284799
PHACTR3	3.325714784	BEND6	-2.195284799
ILDR1	3.319014173	ADRA2C	-2.19512491
LOC106991313	3.313989156	LOC114112489	-2.194634317
IRX4	3.312877674	PAK3	-2.19379803
C12H1orf158	3.311532259	APBB1IP	-2.193066649
CCDC157	3.308344525	THSD1	-2.192352916
C19H3orf20	3.308025576	MARF1	-2.186554188
GC	3.308025576	SFRP1	-2.183278386
LOC105616860	3.308025576	JAK2	-2.182672367
LOC114118506	3.308025576	SCG3	-2.181898066
DNAH3	3.306770219	FMO5	-2.181824482
LOC101108945	3.296286758	ZNF800	-2.177966117
LOC114116191	3.295724254	PDE3B	-2.177214405
F2	3.29142858	SRPX2	-2.176411947
LOC101109626	3.288933394	ABCA6	-2.175792887
ACHE	3.28751827	LOC114115224	-2.17415772
LOC114111480	3.28751827	SLC35D2	-2.173604591
LOC114110252	3.286158114	PCMTD2	-2.172865392
LOC114117644	3.285876569	TMEM151B	-2.171771129
LOC105602261	3.285358102	MREG	-2.170952548
MYH1	3.284361001	LIPG	-2.170581482
LOC114116359	3.283949137	TBXAS1	-2.168047789
DPEP1	3.283786884	CD74	-2.167263427
ZBBX	3.282232731	ATOH8	-2.167237119
C3H22orf23	3.269855967	PPFIBP1	-2.165776172
DNAAF1	3.268266038	TAB3	-2.164961459
CFAP77	3.266037455	BRWD3	-2.164947582
TMC4	3.264674783	TRAF3IP3	-2.16289256
CCDC74B	3.263759121	P2RY6	-2.162537824
LOC114114088	3.259859102	ZNF260	-2.162506677
LOC114114564	3.259859102	SCN4A	-2.16080434
ESRP1	3.258425579	CXCR3	-2.159050369
LOC114113918	3.257777969	MAP3K20	-2.158781786
LOC101102411	3.256777237	PROX1	-2.1573444
CPB2	3.25600395	KRT77	-2.156859383

LOC114111251	3.252680243	ISM1	-2.155000536
LOC105610547	3.241656273	TMED5	-2.151867429
CCDC34	3.240305115	HOMER1	-2.149971273
KRT18	3.240212691	LOC101114551	-2.149806887
MAPK15	3.240127426	SOX11	-2.149577889
TTR	3.239207424	PPP1R1B	-2.14844688
LOC114116064	3.238449005	FMNL2	-2.145239829
TBX4	3.238449005	TIMD4	-2.144187658
NRCAM	3.238449005	PIK3CG	-2.143207107
ENKUR	3.235573328	ZEB1	-2.142352744
HOXD11	3.233901298	TRIM50	-2.141439233
LOC105604801	3.232348789	LPL	-2.141324989
LOC114114527	3.227702141	CPEB4	-2.139245278
LOC114113222	3.222415165	KLF9	-2.137667578
UBE2U	3.22073122	HGFAC	-2.136919759
LOC105604936	3.21987575	MED23	-2.135067803
CFAP61	3.218421877	SLC19A2	-2.132386196
CALB2	3.205256662	LOC105611838	-2.131361199
LOC101121563	3.201394251	MANBA	-2.130330003
VSTM2A	3.196734946	LOC101123533	-2.12998235
SLPI	3.196734946	LOC106990141	-2.12998235
OTOS	3.196734946	FAM122B	-2.129250126
RXFP4	3.196734946	HIP1	-2.128811376
LOC101121854	3.196734946	THBS1	-2.126924233
RIPPLY2	3.196734946	LOC105602100	-2.126816769
CRABP1	3.192024828	RNF38	-2.124809886
LOC114115590	3.191331548	HELB	-2.123820988
LOC105603814	3.186852413	ADPRH	-2.123509672
LOC105610844	3.186739347	MED13L	-2.122841058
MUC20	3.186739347	LOC114115371	-2.122015575
LOC101102546	3.186133749	LOC101120093	-2.120080968
LOC105609642	3.182427994	SLC8A1	-2.119235252
LOC114109648	3.182427994	SETD7	-2.118784313
LOC114109690	3.182427994	GRN	-2.118502219
MYRFL	3.182427994	CPEB3	-2.11846813
LOC101112981	3.182427994	SLC5A8	-2.117762894
PACRG	3.181532527	TTYH1	-2.116248787
SLC12A3	3.180541078	SECISBP2L	-2.113436478
HCAR2	3.179894249	PSAP	-2.111521835
NKX1-1	3.179397794	LOC105610214	-2.108216808
EPHA1	3.177666969	RAB26	-2.107693545
TMEM52	3.175810075	NRP2	-2.106511473
KIAA1257	3.175413119	TRPM6	-2.106048428
IHH	3.17080846	MID2	-2.10501463
NEK11	3.163039343	NEIL3	-2.10375084

KLHDC8A	3.159481862	FOSL2	-2.103750378
LOC105602588	3.154267674	LOC114115665	-2.103125351
LOC101112678	3.154267674	ZBTB16	-2.101847978
PP2D1	3.153102139	FBLN1	-2.099195179
LOC114118739	3.153102139	CD83	-2.096243099
ATP2C2	3.149612443	LOC105616540	-2.095129584
FAM133A	3.149298998	LOC106991096	-2.094171347
RASSF7	3.140656976	LOC101102001	-2.09236907
DYDC2	3.136982041	LOC101116896	-2.090100275
KLHDC7A	3.136470121	LOC101109746	-2.089144444
C13H10orf67	3.135058234	FCER1G	-2.087170538
LOC114116609	3.132375146	SLCO2B1	-2.086510782
CXCL11	3.131849815	ADAP1	-2.086266587
SYNGR4	3.131849815	LOC114108727	-2.083647068
ZMYND12	3.124733185	LOC101114456	-2.082131141
DLEC1	3.118990023	CDK17	-2.081887915
LOC114109549	3.117992917	LOC114114809	-2.078182086
SCTR	3.112731723	ARHGAP6	-2.076305486
TTC23L	3.110547042	ABI3BP	-2.07562891
GPR12	3.108578905	LOC101111337	-2.073256623
LOC114117579	3.108578905	EPS8	-2.072317371
AMZ1	3.107332536	TPP1	-2.070225618
TTC12	3.103265402	LOC105615470	-2.070027996
LOC114117876	3.096607219	ITGA4	-2.070023919
LOC101102519	3.096607219	PDE3A	-2.070021917
CLDN3	3.092676873	CITED2	-2.068736031
CROCC2	3.090671819	CIDEA	-2.0683789
LOC105602529	3.090449908	AP5M1	-2.067272855
LOC105604901	3.083817824	PYGO1	-2.066163538
LOC101107261	3.080569458	KBTBD12	-2.064139381
TMEM232	3.080086246	CD5	-2.062536914
LOC114118689	3.072305234	LOC101122940	-2.06213002
LOC114110645	3.070186638	RPS6KA3	-2.060956725
ARHGAP8	3.069352546	SOS2	-2.060689793
LOC114112236	3.067507887	OSBPL8	-2.059743247
SLC7A10	3.065290542	GIMAP6	-2.057023556
LRRC71	3.055666112	NID1	-2.055966294
MARVELD3	3.055502858	LYVE1	-2.055598645
WDR66	3.054940041	ADA2	-2.053938981
LOC101108413	3.053818515	GLA	-2.051700699
SHISA8	3.053351876	RIPOR2	-2.051276874
LOC114114101	3.046955604	LOC105610402	-2.048352426
TLE7	3.046014268	ARL5A	-2.046990686
GPER1	3.044916993	CD44	-2.046298484
LOC105605507	3.044099527	LRRC38	-2.045857158

TPD52L1	3.043653506	LOC114113253	-2.04548192
PLLP	3.040523412	IRS2	-2.043353355
EFHB	3.040022955	GDF11	-2.043165284
TMEM212	3.038761489	IGF2R	-2.042912533
LOC114112644	3.037768486	LOC101114597	-2.040730253
LOC105606472	3.031901005	CCL19	-2.039089846
P2RX6	3.031562107	ADAMTSL4	-2.037801555
LOC105608537	3.030744699	SCPEP1	-2.036138542
LOC114111160	3.030744699	TNNI2	-2.034581955
C3H2orf73	3.030744699	LGR4	-2.032767608
SOSTDC1	3.030744699	CD244	-2.031583322
CCNA1	3.028225515	SH3RF3	-2.031491897
LOC114117971	3.025123249	RRAGC	-2.02892423
LOC105609277	3.02322388	LOC105603201	-2.02854322
NEK10	3.022732341	LOC106990140	-2.028415534
LOC114110815	3.021622084	LOC101103154	-2.028275865
PMCH	3.016741194	RFK	-2.02718724
LOC114111467	3.016741194	PIK3CD	-2.027071054
LOC105608014	3.014916725	HACD1	-2.024876904
LOC105601897	3.014164215	UHRF1BP1L	-2.024560107
TMEM139	3.012223652	MYOCD	-2.024151582
NGEF	3.009741583	ZC3H6	-2.022726141
LOC106991640	3.007486919	MBNL3	-2.020812879
LOC101105860	3.006403235	NABP1	-2.020193839
LOC101109157	3.002902833	BMF	-2.019973173
LOC114108851	3.002902833	LOC101105400	-2.019229301
WDR54	3.002543168	CLIC4	-2.017835236
LOC114114551	2.994539003	LOC101111099	-2.016699298
COL9A1	2.990194072	RGS4	-2.015353487
PLPPR3	2.989704341	NFASC	-2.014747869
PDZK1	2.987138541	LOC114109118	-2.014259021
ATP2B2	2.985808176	STK32A	-2.014097078
KRT3	2.985808176	ITPRIP	-2.013611866
LOC114113895	2.985808176	LRFN3	-2.013339009
LOC114109444	2.984343156	LCP1	-2.013128315
LOC105613573	2.983586038	TGFBR3	-2.011555934
SH3GL2	2.973183316	FMOD	-2.009716288
CASC1	2.970333842	NR1D2	-2.008387059
MAK	2.964943423	WDR97	-2.00747367
LOC105613342	2.956468595	SGIP1	-2.007087455
LOC105607312	2.955548945	GOLPH3	-2.005954946
CCDC68	2.955548945	CSF2RB	-2.005919636
LOC101113369	2.948470622	SETD9	-2.00458315
AVIL	2.948470622	LOC105614110	-2.003463473
LOC114109027	2.948470622	KCTD8	-2.003068267

HOXA9	2.948470622	BTBD11	-2.002294992
LOC114116897	2.948470622	FAM124A	-2.002230518
LOC101117129	2.94623027	LOC101112543	-2.001072465
FAM221B	2.94468023	LOC114114763	-2.00010632
LOC114115662	2.944490699	DIXDC1	-1.999159852
KIF12	2.94363439	MBTD1	-1.99888145
LOC114111410	2.941200624	THAP2	-1.995754582
LOC105611978	2.941200624	ANTXR1	-1.992268651
LOC114109079	2.940145946	CRYBG3	-1.990751533
CFAP65	2.938931651	LOC101103472	-1.989008468
GJB4	2.929313255	DCAF10	-1.988621196
LOC101117493	2.929313255	CCNI	-1.988012267
LOC105605964	2.925861511	IGFBP5	-1.987249838
LOC101121715	2.925861511	CAV2	-1.98450452
LOC114114003	2.923065154	LOC101106542	-1.984259015
DERL3	2.923065154	FAM180B	-1.98204671
EEF1B2	2.921927413	LOC101102726	-1.981123445
GNMT	2.91967379	LPAR5	-1.980679305
TBX6	2.919531612	PACRGL	-1.979709104
PTRH1	2.918437369	UNC80	-1.979411743
SH2D5	2.916712531	PNRC1	-1.979132968
LOC114112967	2.912496757	ERO1B	-1.977676108
LOC114114553	2.912496757	NF1	-1.977656276
PPP1R14D	2.912496757	FAM20A	-1.976473321
KRT19	2.908641847	TAS1R3	-1.975149082
PRR19	2.906749842	TWSG1	-1.974970675
LOC105611875	2.906292588	SSH2	-1.97395155
SYBU	2.904817727	SNX18	-1.973295167
LOC114115264	2.902710124	QSOX1	-1.97110394
LOC114108747	2.900213923	KLF11	-1.969986156
MAPT	2.899546704	ARHGAP9	-1.969004923
RGL3	2.898737668	YJEFN3	-1.965294037
KLRG1	2.891645804	KIF5A	-1.965294037
BCO1	2.891645804	LOC114114537	-1.965294037
KCNJ14	2.891645804	RGS7BP	-1.964877643
NPC1L1	2.891645804	IL2RG	-1.962942402
LOC101116902	2.890675201	SESN3	-1.962489856
LOC114110572	2.890675201	LOC101107365	-1.962053435
TFAP2E	2.890279108	LOC114110326	-1.961960743
KY	2.890063924	LOC114109061	-1.960715836
LOC101108868	2.890063924	BACH2	-1.957839867
MEGF11	2.889928751	PAWR	-1.957614587
LOC114113716	2.88983596	KITLG	-1.95680295
LOC105607199	2.88983596	AADACL3	-1.956480895
LRRN4	2.889768318	LOC101111669	-1.954683588

KRT8	2.887528255	ZMYM1	-1.953987266
LOC105603666	2.887230309	LOC105611737	-1.953701445
S100A1	2.877223477	MFAP4	-1.952848631
CNTNAP4	2.875192052	LOC101117184	-1.952476638
CCN6	2.875192052	AUTS2	-1.951731879
GPR1	2.875192052	TMEM187	-1.951487197
ENPP3	2.874953207	CCDC186	-1.950489017
DNAH7	2.874078264	HSPBAP1	-1.949250691
LOC101111394	2.870267733	SFR1	-1.949120607
SHANK2	2.869752231	BST-2A	-1.948657138
TDRD5	2.867840178	ELK4	-1.948317138
C4BPA	2.866426999	SP110	-1.944899176
WWC1	2.865041298	KLF3	-1.944713639
LOC105609383	2.86427696	PIK3AP1	-1.944049397
LOC101111035	2.861219346	SPON2	-1.94299705
CD72	2.861219346	ITGA9	-1.941449163
KIF6	2.859942536	SLC26A2	-1.93944763
LOC101115174	2.859536918	GAS2L3	-1.938538802
LOC114118856	2.852640808	RAB31	-1.938526453
LOC105602877	2.851044625	NFAT5	-1.938413749
LOC101114379	2.851044625	CDH7	-1.937441524
LOC105602189	2.851044625	TCF7L2	-1.93573676
LOC105603494	2.851044625	ITGAL	-1.935734415
LOC105616290	2.848292167	ZFPM2	-1.935637456
LOC114116808	2.845507189	BGN	-1.935054206
C1H1orf87	2.845507189	TC2N	-1.934904178
PALM3	2.834174444	GRIP1	-1.932622188
LRRC6	2.830814649	PHOSPHO1	-1.931978972
SLC44A3	2.83040251	LOC114111781	-1.93132033
BAIAP3	2.827828725	APOE	-1.931050388
LOC114113184	2.826943809	FAM126A	-1.929058235
LOC106991067	2.826554107	GNAI1	-1.92876473
CLGN	2.825070203	VGLL2	-1.927637311
LOC101118495	2.824780402	LOC114113943	-1.927637311
LOC101106641	2.824780402	HOXD1	-1.927637311
RHPN1	2.822120162	DIPK2A	-1.922943419
KDF1	2.821281379	LOC101116085	-1.922779699
LOC114109647	2.812840995	BCL6	-1.922438096
CBLC	2.80969538	TDRD9	-1.921260017
LOC105609450	2.809219331	FRRS1L	-1.917599476
SPAG6	2.809008968	RO60	-1.916884853
LOC114113961	2.800962187	LOC114111361	-1.916602163
BLM	2.797405556	SCNN1B	-1.916052681
USP44	2.790596672	TRIM33	-1.913903408
OXTR	2.789972755	LOC101106227	-1.912766767

LOC106991047	2.789732878	COL23A1	-1.912331488
CCDC24	2.788556039	THAP5	-1.912331488
KIAA1324	2.784993759	ANPEP	-1.911504067
AGBL2	2.78454166	APPL2	-1.909468742
LOC105611708	2.784195602	DPT	-1.908285069
INKA2	2.783591321	ITGA10	-1.908181941
LOC114109086	2.78220267	LRSAM1	-1.906544118
LEXM	2.779418452	GULP1	-1.905525006
LOC114114916	2.77525494	FAR1	-1.904068453
WDR93	2.77361709	STAB1	-1.903433755
LOC105616364	2.768705527	COL6A1	-1.902717219
KIF25	2.768349677	LOC101105099	-1.901690376
SLC17A9	2.76496208	IGDCC4	-1.900700196
LOC105603421	2.754664697	DUSP26	-1.899880898
LOC114118449	2.754664697	MZT1	-1.899592381
LOC114117563	2.754664697	LOC101107970	-1.899070632
LOC114108814	2.754502843	MTSS1	-1.897996974
LOC105605777	2.754465947	ZNF354A	-1.896859464
SPA17	2.74711361	HOGA1	-1.89641672
MOXD1	2.745505913	STXBP5L	-1.89641672
PPP2R2B	2.742215428	ZNF81	-1.89641672
SLC12A8	2.735156329	TLR3	-1.896188868
LOC114110256	2.733359284	CDH19	-1.895258839
IQCG	2.732471145	SELP	-1.895191826
LIN7A	2.731891762	PAPOLG	-1.895083827
LOC106991743	2.727576046	SCN3B	-1.893704305
LOC106991039	2.724640448	STK32B	-1.892621524
MPIG6B	2.720620748	BTNL9	-1.891045255
CCDC160	2.718974835	PELI2	-1.888784733
GPX2	2.717764903	CTSZ	-1.888382251
ACTL8	2.716985126	C1H1orf162	-1.888354852
LOC114117983	2.715263401	LOC114109611	-1.887880478
LOC101110202	2.715189624	ITPKB	-1.88659695
LOC105608234	2.714581978	CCNG2	-1.88659695
TMPRSS6	2.714581978	PDGFRA	-1.886225682
UTF1	2.714581978	EPHA10	-1.88272343
SEC14L3	2.714581978	CATSPERG	-1.882026773
LOC114113805	2.714581978	FNDC3A	-1.877918667
LOC101109890	2.714581978	SEL1L	-1.875513857
GTL2	2.713765894	LOC101105484	-1.87358845
UBXN11	2.708285912	OXR1	-1.873345495
TP73	2.706448422	LYST	-1.872827616
CACNG7	2.706008756	CCL5	-1.872665584
THRSP	2.7041537	NLRP1	-1.872007975
LOC114115635	2.7041537	LYSMD3	-1.871781947

LOC106991041	2.701405851	ALDH6A1	-1.870497803
LOC105606185	2.701405851	SLC10A7	-1.868424625
ALX3	2.701405851	ZNF516	-1.865685944
LOC105605590	2.700137108	CXCL12	-1.865268065
LOC105616296	2.698597991	TWIST1	-1.864102502
CCDC154	2.690298152	EDN3	-1.862231579
ENO4	2.689726294	ZBTB3	-1.861596231
SLC25A47	2.682804153	HFM1	-1.861596231
SPC24	2.677871545	WIPF3	-1.861277562
FBXO40	2.674329569	THBS2	-1.858948635
LOC114113887	2.672166167	FCGR3A	-1.858376698
LOC114117970	2.669087939	HSD17B7	-1.857994737
EFHC1	2.668735556	PSTK	-1.857182752
LOC114108756	2.664833139	TRPS1	-1.856581768
LOC106991287	2.663749801	RAB27A	-1.8559881
CNTNAP2	2.65976708	LOC105604767	-1.854647961
LOC105607745	2.659324528	SLC38A6	-1.854211351
CALR3	2.659324528	FNBP1L	-1.85367586
LOC105603319	2.659324528	BMI1	-1.851711237
LOC114115994	2.655440548	NINJ2	-1.850372018
TRIM9	2.655066914	SPATA6	-1.850085443
LOC114109056	2.654445563	SUCLA2	-1.849333515
LOC114116371	2.652682172	PLD3	-1.848513236
OIP5	2.646548368	FAM160B1	-1.847234567
FOXR1	2.64609593	IFI44	-1.846561667
LOC101103165	2.643846082	PARVG	-1.846428393
LOC105606509	2.643796084	LCORL	-1.846369411
LOC114109649	2.643730855	LOC114110253	-1.844142339
LOC106991936	2.64296145	C1R	-1.843891163
LOC106991387	2.64296145	ADAT1	-1.843794624
CA14	2.642154779	SSC5D	-1.842157658
LOC114114902	2.642154779	TMEM243	-1.841533568
STMND1	2.641250152	FLRT3	-1.841026414
CDCA3	2.636041873	LOC101109747	-1.839817015
TEX26	2.632610207	COLEC12	-1.839108538
TSGA10	2.632529805	PGBD1	-1.838773808
FAM83G	2.631386506	GALC	-1.838266745
MLIP	2.628663837	RAB19	-1.838081483
IRF6	2.627300689	LOC101116039	-1.838081483
FAM167B	2.624678974	LOC106990145	-1.838081483
LOC105616327	2.623571645	WDFY4	-1.837785671
STOX1	2.623571645	LOC114115660	-1.836754708
LOC114110991	2.623571645	MICU3	-1.836324849
LOC101112606	2.622447726	LOC105608589	-1.836192449
RHBDL2	2.621327166	CD99L2	-1.83614937

AURKC	2.617224325	FAM83H	-1.836086218
LOC106990385	2.617093401	DMXL1	-1.835197738
GRTP1	2.609094602	SPRED2	-1.834793544
TK1	2.607461264	LIN7C	-1.834778157
JAKMIP2	2.603524367	ZFP36L1	-1.832589789
LOC101112834	2.603299857	VCPIP1	-1.831688844
LOC114116706	2.603299857	FBLN5	-1.831423397
CCDC190	2.597627959	GFAP	-1.831219278
TSTD1	2.596643796	VPS37A	-1.830272468
LOC105605855	2.59530799	CPN1	-1.828659605
EXTL1	2.593253328	JRKL	-1.827143874
LOC114115262	2.593253328	NPL	-1.826941754
LOC101115632	2.58805091	CPT1A	-1.823133844
LOC106991099	2.585073839	ANGPTL2	-1.822485713
DNAH10	2.584384145	LOC105612436	-1.822332903
NAALADL1	2.583021018	LOC114114000	-1.819704584
B4GALNT3	2.582890269	SIRT1	-1.819474095
KIF5C	2.582890269	INSIG2	-1.81694904
GPR160	2.582852378	SAMD8	-1.816463168
CLSTN3	2.580049767	IL10RA	-1.816265081
RTN1	2.580022314	TMEM106B	-1.816156139
C12H1orf116	2.57905831	BATF3	-1.815779148
LOC105605499	2.57905831	RNF216	-1.815182194
RGS22	2.575978824	FAR2	-1.814687495
IL3RA	2.575238254	MDFIC	-1.8126889
LOC105604496	2.570387964	CD300LF	-1.812397582
LOC105602706	2.565982583	APAF1	-1.811982021
TMEM61	2.564455496	LOC114117223	-1.8109128
LOC105604743	2.563850048	LOC101111046	-1.8109128
LOC114113900	2.56248875	BICRA	-1.810510154
WDR38	2.561785145	USP46	-1.808641985
LOC114110131	2.55866487	COL21A1	-1.808519792
LOC114113877	2.557553605	MST1R	-1.806374967
LOC105615265	2.557468257	RNF157	-1.806265796
HAND2	2.556439153	FAM180A	-1.804944561
RBMX2	2.552248962	LOC114110434	-1.801795524
A2ML1	2.551481572	TIFA	-1.801795524
LOC114116702	2.550445974	ZBTB38	-1.801123665
TDRD10	2.549130756	PCDH8	-1.800418174
C16H5orf49	2.548694753	LOC101103461	-1.800335547
RSPH9	2.545595941	LOC101105533	-1.799636663
LOC105606221	2.544214705	NLGN3	-1.799636663
MYB	2.54370912	VPS13C	-1.798820538
MAS1	2.541297568	FGD2	-1.798171829
LOC101106041	2.541297568	NRP1	-1.797601054

LOC114113902	2.541297568	LOC101122398	-1.795850585
LOC114108812	2.541297568	SLC24A3	-1.7958145
ZNF488	2.539649483	TRUB1	-1.795353934
LOC114108643	2.538799466	MAMLD1	-1.795074138
CSMD1	2.538291816	HTRA3	-1.794918876
LOC114115288	2.537862533	CTSB	-1.792798349
LOC114117872	2.537862533	TMEM156	-1.792450575
PGP	2.535893976	LOC105605117	-1.792450575
LOC105607019	2.535869741	TKTL1	-1.792450575
NETO1	2.535248703	PCSK6	-1.792234543
LOC105606707	2.534111887	LOC114117868	-1.791814417
LOC114117863	2.533590213	CAVIN2	-1.791717835
CCDC197	2.528318422	LYN	-1.787747709
LOC114109670	2.528318422	ATF7IP	-1.787094068
LOC106991195	2.528318422	CCDC126	-1.786855507
TTLL1	2.517841657	CLDN10	-1.785660194
ASB11	2.513566669	SMAD5	-1.78563793
ELAVL3	2.511222069	TTC5	-1.783276257
LOC105612323	2.51069354	ACACB	-1.783090177
NT5C	2.509061657	ATP1B2	-1.782387975
SAPCD1	2.508464201	PENK	-1.78168044
LOC114108781	2.508464201	ZFP36	-1.779792939
LOC101108102	2.504690389	LOC114112164	-1.778172019
LOC105608311	2.501740592	ELMOD1	-1.777880816
STK31	2.500959075	SCAMP1	-1.777673584
STIL	2.496908418	SMIM14	-1.776108395
PIMREG	2.496151912	PDP1	-1.775006176
ERICH2	2.492104606	PIK3C2A	-1.772725369
LOC105609312	2.490452937	CTSC	-1.772176956
LOC114118404	2.488178015	CAMK2N1	-1.771936216
CFAP206	2.487477239	CLTRN	-1.770959693
STC2	2.484958824	EDNRA	-1.76836953
SPEF1	2.484893592	PPP1R12A	-1.767339672
PLCH1	2.483534478	DOCK8	-1.766778919
LOC105610613	2.482794397	MANEA	-1.764908451
GSTA1-1	2.482733471	HMCN1	-1.764222771
PIH1D2	2.481512557	LOC780455	-1.76384126
MESP1	2.480616288	SGMS2	-1.763741879
C4H7orf57	2.472522837	INSL3	-1.763260112
LOC105604550	2.469765254	PEX13	-1.762202346
LOC101117044	2.469499142	LOC101110131	-1.762070502
LOC101118600	2.468258167	LOC101112109	-1.761979513
LOC105613406	2.466889936	PTPN7	-1.761778293
LOC114111432	2.465250375	TMEM14A	-1.76161228
GAREM2	2.465250375	LOC114114052	-1.761509799

LOC114113872	2.46472047	THEMIS2	-1.761409807
KATNAL2	2.462679015	TNKS2	-1.761117086
KRT5	2.462443439	ACTC1	-1.759954073
LOC443320	2.462371438	AGPAT4	-1.759697823
CA8	2.461550288	ZNF217	-1.75789638
LOC114112163	2.460754565	APOLD1	-1.757789109
LOC105609542	2.460754565	LAMC3	-1.756481253
SLC34A2	2.456175361	VASH2	-1.754539201
SYT13	2.454046292	CREBRF	-1.753113579
ARHGEF16	2.452199883	MAN2B1	-1.753006124
LOC106991997	2.447380128	ZNF711	-1.752875653
FAM240A	2.447380128	C21H11orf95	-1.752207697
KCNJ10	2.447380128	SUSD5	-1.751554292
LOC114114608	2.445095037	LOC114113039	-1.751083653
SH2D3A	2.444629484	NPC2	-1.750594281
MYBL2	2.43873855	CCBE1	-1.750001231
LOC114110484	2.436721211	HECA	-1.749950618
NRTN	2.434754716	ANTXR2	-1.749826858
GSC2	2.434172275	ZEB2	-1.749321681
FRMPD1	2.433753533	ADAM10	-1.748704181
LOC114114545	2.433689067	MTMR1	-1.74664094
SDR16C5	2.432316	UBL3	-1.746631749
LOC114118743	2.43043197	LOC101108113	-1.746285382
SAMD15	2.429636719	COL4A6	-1.746109071
LOC105610935	2.429028255	LOC442995	-1.745626269
C1H1orf189	2.426467902	F13A1	-1.74505204
ATP6V1G2	2.426456111	BIRC3	-1.743785834
RP9	2.423216518	GJA4	-1.743552059
LOC101110417	2.422795705	TMEM80	-1.741998678
LOC105616901	2.421740467	PM20D1	-1.741998678
LOC105603203	2.421703099	DOCK3	-1.740990264
C1H1orf210	2.421614103	PEG3	-1.739998022
LOC114110988	2.421127369	SV2B	-1.739904865
LOC105602161	2.421127369	CALCOCO1	-1.739627991
LOC105611892	2.421127369	LATS1	-1.738604817
LOC114116343	2.421127369	GRIA4	-1.735847421
EIF5B	2.420215411	LOC105610483	-1.732120678
SP7	2.418379028	LGMN	-1.729874146
REEP6	2.417199075	LOC105605861	-1.729323608
MKRN2OS	2.416044038	TGIF1	-1.728084413
HPN	2.410870342	SLC7A2	-1.727629304
CFAP58	2.408930692	LOC105604541	-1.72599544
CCDC162P	2.407940075	MIER3	-1.724412536
LOC114109131	2.405153342	F8	-1.722717775
LOC114111217	2.40250604	LOC101122123	-1.722027662

SPAG8	2.401822002	C1H1orf52	-1.721643657
LOC114111357	2.399458345	LOC105610456	-1.721483689
SPEM1	2.399458345	RASGRP2	-1.721483689
DNAH9	2.399054811	LOC101107401	-1.720405921
GGCT	2.394010661	PDE4B	-1.720220959
SLC47A2	2.393836439	HRH1	-1.720020296
LOC105611533	2.393002466	LOC105607354	-1.720020296
FCRL5	2.392227063	RXRG	-1.720020296
DNAH1	2.390689679	LOC101117622	-1.719139235
N6AMT1	2.390403537	ACOT11	-1.719046949
YDJC	2.388843722	FRS2	-1.718195016
RBMXL2	2.384797008	SLC9B2	-1.717493157
LOC114115641	2.384217084	PRELP	-1.715581605
LOC114109622	2.384217084	DQA	-1.714569157
ELF3	2.38397095	BMPR1A	-1.714476972
LOC105602038	2.382882364	GPC3	-1.71423359
C20H6orf141	2.379597353	AFAP1	-1.713900194
SKP1_2	2.377824226	TBX1	-1.713578882
LOC114110277	2.376196122	LOC114118047	-1.71313183
LOC114116379	2.374476067	ATMIN	-1.712488785
CDKL4	2.36510616	LMO7	-1.712141941
LOC106991033	2.36510616	C3H2orf40	-1.710920534
STC1	2.364904845	SASH1	-1.709404463
STYXL1	2.363218716	LOC105603102	-1.709277035
DDX4	2.361176624	MNT	-1.707080776
LOC105612174	2.361176624	SLC40A1	-1.706165094
LOC101114146	2.361176624	LOC101115787	-1.705887589
RAB11FIP4	2.354697725	OIT3	-1.703335325
TMEM125	2.353537236	ETNK1	-1.70324776
EPN3	2.351988194	ETS1	-1.701115695
LOC114113815	2.347811015	NRROS	-1.700774282
MLPH	2.347708881	STON2	-1.700040935
ADAD1	2.344307164	TAOK3	-1.699852067
FDPS	2.340164263	S100A9	-1.699419995
LOC105616340	2.33210612	PLN	-1.699176889
LOC106990241	2.331408951	MIER1	-1.696947102
DSG2	2.329708274	CTDSPL2	-1.696578849
SEC62	2.329292128	CCNJ	-1.694836878
C13H20orf96	2.327825499	PDZRN4	-1.694761805
LOC101121119	2.327778418	FGFR1OP2	-1.69382014
RPGR	2.327676128	PIGW	-1.693611751
LOC101118224	2.323774785	KCNA3	-1.693037473
LINGO3	2.322284738	DOCK4	-1.69204919
LOC114112176	2.320785668	LOC101109388	-1.690448002
CDH1	2.320214281	FGL1	-1.689944804

NPM2	2.317635257	MMP14	-1.689164408
DCHS2	2.314308795	SFMBT2	-1.688336377
RNF32	2.314308795	COL1A2	-1.687966128
CHST8	2.312870925	TIMMDC1	-1.68667759
LOC114108794	2.312399274	CARNMT1	-1.685688501
APOB	2.312399274	LOC101113211	-1.684838228
LOC105609910	2.311660027	SLC15A3	-1.684120405
C24H16orf71	2.311584985	MYH2	-1.683633292
ATP6V1C2	2.311465415	UCP2	-1.682894799
RSPH10B	2.311160614	CDH6	-1.682883255
LOC114116895	2.306382878	CDK19	-1.682726852
LOC105605602	2.306382878	SCARB2	-1.682666036
ZIM2	2.306382878	COL4A3BP	-1.681098733
LOC114110457	2.306382878	PCYOX1	-1.680303162
ICAM5	2.304691886	PLAC8	-1.680230055
TEX9	2.302401308	CLCN5	-1.679556938
TNFRSF6B	2.300786577	GLS	-1.678961268
KRTCAP3	2.295996228	ROCK1	-1.678323711
LOC114116727	2.293331995	LOC114114463	-1.676703451
PPARGC1A	2.293028454	LOC105603609	-1.676464939
UBE2S	2.291634223	CRISPLD2	-1.676067475
CBY3	2.290712363	SHISA2	-1.674368468
ALKAL2	2.290712363	CFD	-1.673432012
LOC114112857	2.290610194	ELF1	-1.673347484
LOC114113232	2.289353604	LOC101115509	-1.672332393
RHOV	2.289039934	TGFBR2	-1.672302319
PCNX2	2.289039934	RAB40B	-1.671697955
B3GNT8	2.289039934	SLC7A7	-1.671109011
LOC101119236	2.288472931	KBTBD11	-1.671109011
LOC101118315	2.288178456	SEC23A	-1.668998723
LOC106990495	2.286752328	PTPN14	-1.667911079
ARHGDIG	2.283859745	TPT1_2	-1.667651113
DSC2	2.27944202	STON1	-1.667589061
SUGCT	2.275031873	NR2F2	-1.667059445
LOC105608331	2.274431965	PRKCI	-1.666072697
C5H19orf71	2.273532034	PLCB1	-1.665530122
LOC114118448	2.27159361	LOC101117587	-1.664818022
GPBAR1	2.27153565	ERG	-1.66388968
SNX22	2.268441435	FLT4	-1.662989119
ATG9B	2.268309819	C5H5orf30	-1.661528337
CLDN15	2.264646058	RNF44	-1.661497248
LOC114118010	2.261914885	WWC3	-1.659527188
MLN	2.261914885	SNX29	-1.659517383
LOC114116099	2.261914885	DUSP19	-1.658814633
LOC114111205	2.261914885	FAM91A1	-1.658576147

LOC105604649	2.261914885	NADK2	-1.657549321
ARHGEF33	2.261914885	CCN1	-1.657323467
ELAVL2	2.261914885	CD47	-1.657206714
LOC114110654	2.259612294	MEF2A	-1.657165761
FRRS1	2.258112342	YTHDF3	-1.65620028
LOC114117981	2.256633474	KIAA0895	-1.656079532
LOC114113898	2.255419336	BRPF3	-1.653729321
MTFR2	2.255045168	KCNMA1	-1.653521504
LOC106990353	2.253548307	ATXN7	-1.653238284
LOC105609969	2.253548307	RAB27B	-1.652795961
SPIRE2	2.251593869	NDFIP2	-1.652682962
CENPW	2.251518667	ST8SIA4	-1.652395615
CENPN	2.251092256	TIE1	-1.652184051
LOC101112688	2.249779821	BICRAL	-1.651936025
LOC105603818	2.248133775	LOC114110471	-1.650537186
CPT1B	2.247252349	CLCN6	-1.650288762
AURKB	2.245875701	FGD6	-1.650099228
LRRC36	2.245285724	LOC114113985	-1.649032512
AK8	2.244097687	GJB3	-1.649032512
LOC114115334	2.243507019	PLEKHA2	-1.648525192
ONECUT2	2.242551803	NRK	-1.646694987
LOC105606662	2.242551803	RCN2	-1.646066688
VSTM5	2.242551803	MOSPD2	-1.645898936
LOC105606907	2.241064645	MAPKBP1	-1.64537573
LOC105609411	2.240629428	MAN2A1	-1.644528795
TAFA3	2.240299922	ECM2	-1.644014837
LOC114108993	2.237131761	ITPRIPL2	-1.643835468
ZNF446	2.236938414	LOC105607326	-1.640362048
LOC114116886	2.233753691	CDH5	-1.639212211
SYT2	2.229276146	CERS6	-1.638579316
MSLN	2.226538649	ALX1	-1.637807407
KCNJ15	2.224108801	PPM1B	-1.637437784
FAM181A	2.224108801	TNFSF13B	-1.637247385
ADGRG7	2.224108801	SLC18B1	-1.636967989
LOC114118712	2.223600792	DCLK1	-1.635199813
PARP3	2.223149569	RIC1	-1.634066193
BTBD17	2.219345514	XIAP	-1.633272724
CRYBG2	2.213512028	ARX	-1.632284067
LGALS4	2.21047464	CNKSR3	-1.631775282
BHMT	2.210306832	RAP2B	-1.631395163
RHPN2	2.208175462	TBL1XR1	-1.629721849
LOC114116877	2.207982686	LOC114116464	-1.629458234
IZUMO4	2.206059303	FNDC3B	-1.629453484
LOC105605321	2.202536052	GCNT4	-1.628274207
LOC105605445	2.200799371	TBCK	-1.628176988

LOC114111386	2.19819999	AKAP4	-1.627553295
DLX5	2.19819999	C19H3orf62	-1.626720537
LOC105616454	2.196231619	RNASE6	-1.626218316
SKOR1	2.193328526	LOC101117786	-1.625990437
LOC114116443	2.192836689	GRIK2	-1.624306495
PCBD1	2.191964542	HFE	-1.624203942
HDDC2	2.190610627	TNFRSF13C	-1.62388962
CCDC148	2.188923414	AGO4	-1.623654469
LOC101118212	2.188923414	SEMA5A	-1.623641045
PRR22	2.186582041	ATF7	-1.623221383
LOC114117559	2.185303632	LGI4	-1.622169655
SDR42E2	2.185303632	NSG1	-1.621813546
LOC114111487	2.185303632	ARID2	-1.621379793
AKNAD1	2.18497564	NUP58	-1.620934183
TUBB4B	2.184405128	LOC114109398	-1.62022243
LOC114109691	2.181725191	PLEKHA5	-1.620010901
ALDOB	2.178351815	MARCH6	-1.619203825
CABP1	2.178316928	TRDMT1	-1.618326332
LOC101119572	2.177674364	SERPING1	-1.617794119
VEGFA	2.177543327	C8H6orf203	-1.61734639
LOC101108898	2.176895663	PID1	-1.616528202
MYH7B	2.176144962	AVPR1A	-1.616084048
IQCD	2.17445741	RALGPS2	-1.615107547
FRMD5	2.17275172	LOC114110449	-1.614142523
LOC114117859	2.171725968	LITAF	-1.613649502
ANKMY1	2.170823821	EGR1	-1.612517951
ND1	2.169334552	FRZB	-1.612457296
GALNT5	2.168222215	ZBED3	-1.612135407
MOV10L1	2.168222215	LOC105610484	-1.611816944
GPR17	2.166923968	MYOF	-1.611718344
NOXA1	2.165391986	NPY1R	-1.61141354
LOC105607925	2.164979779	DIO2	-1.6112194
LOC114116831	2.164979779	JMJD1C	-1.611180081
CLCN2	2.163861817	RAC2	-1.611073278
LOC105612707	2.163596393	PIK3R5	-1.610812204
LOC101106541	2.1601681	ZFR	-1.610627622
ANGPTL5	2.159384906	OGN	-1.610180062
LOC114117763	2.158881158	GPR155	-1.609668439
RFX2	2.157497546	FBXL17	-1.608243033
LOC114114048	2.155756304	PARD6G	-1.608021997
HRG	2.155315696	TRPM7	-1.607705869
LOC101118452	2.155315696	THRB	-1.607452954
LOC105610334	2.155077753	TMOD4	-1.607349154
CRISP2	2.152869211	PHC3	-1.606861753
TPPP	2.148156639	GBE1	-1.606100276

MYL10	2.147679985	MINDY2	-1.605346655
SLC35G1	2.144975623	LOC105602949	-1.603938327
ASF1B	2.144953729	LOC114108675	-1.603244043
TNFAIP8L1	2.141681615	NUCB1	-1.603202581
TPPP3	2.141614738	GINM1	-1.602343012
DIRAS3	2.140658272	LOC101112639	-1.601767338
ATP4B	2.139908463	DIP2B	-1.600695945
LOC101111694	2.138464139	PLXDC2	-1.600507841
DOK4	2.138448024	HHEX	-1.600416343
SH3RF2	2.137205581	FAM126B	-1.600366665
AQP9	2.137193449	SNRK	-1.598634927
LOC114112917	2.137185288	ETV1	-1.598472338
LOC105611136	2.137117833	LGALS3	-1.598288496
LOC105609002	2.13707811	GM2A	-1.596578702
CD79B	2.136857525	CRIM1	-1.596164284
LOC105603423	2.136857525	DPEP3	-1.596029117
ATP6	2.136277554	LOC114110414	-1.596029117
LOC101104482	2.136266765	S1PR1	-1.595211025
HELLS	2.135627103	PCDH19	-1.592621832
CTH	2.133557435	MYLIP	-1.592399868
FAM161A	2.132068953	LOC101102839	-1.591470069
TRPV4	2.131005313	FOXO3	-1.590873555
SCX	2.130786394	LOC114116066	-1.589516363
LOC114110055	2.130272054	SNX10	-1.588427258
LMO1	2.121892355	LOC554335	-1.588301915
LOC114117590	2.121584781	LOC114113132	-1.588077742
CRB2	2.121584781	SLC25A32	-1.587651887
LOC101108295	2.121493996	CDH13	-1.587347309
LOC114108644	2.120830738	LOC114113204	-1.586686371
LOC105602517	2.120516843	SLC38A2	-1.586242158
LOC114116866	2.115998208	NEDD4	-1.586241458
EPS8L2	2.115545185	TBX3	-1.586014042
CLBA1	2.11548423	LOC105609832	-1.585390018
LOC114114493	2.115316671	TBC1D8B	-1.585253804
CCDC158	2.113614891	NDRG4	-1.584656136
MNS1	2.113553849	GADD45A	-1.583805428
LOC105607964	2.111716632	CASD1	-1.583719404
LOC114118091	2.108484858	OSR1	-1.583397751
F8A1	2.106335383	MTMR11	-1.582675379
FCGBP	2.106294742	NEMP1	-1.581617599
C2H9orf43	2.105993042	STK17B	-1.580903407
CENPS	2.10457139	ZNF214	-1.578855261
LOC101112584	2.104381035	MSX1	-1.577921187
WDR90	2.104162855	RBAK	-1.577523602
PAG1	2.102695233	PITPNC1	-1.577183224

CENPV	2.102529502	GNG12	-1.577167436
PNMA1	2.101740961	SAMD14	-1.576806973
PRRX2	2.101680969	SMPDL3A	-1.575618404
KCNA5	2.101680969	PIK3R1	-1.575198898
LOC101107031	2.098909105	MAP3K9	-1.573569383
LOC101116391	2.098909105	SAT2	-1.572856919
LOC105615388	2.098909105	LOC106991591	-1.570716726
LOC105605730	2.098838241	WHRN	-1.570716726
ERI2	2.097119812	ULK2	-1.569890275
SYTL1	2.09572321	LOC101106528	-1.569255324
LOC105602796	2.094620921	RRNAD1	-1.568365992
LOC105604737	2.094620921	CREBBP	-1.568045238
UCHL1	2.094502408	SH3PXD2B	-1.567162041
MFAP5	2.094132889	NFKBID	-1.566558544
ISM2	2.091169841	OVAR-DRB1	-1.56636272
LOC101110185	2.090680515	GFPT2	-1.566348705
LOC105614928	2.088332537	UBE2W	-1.566128758
B3GNT4	2.087694335	RUNX1	-1.565740475
LOC114113183	2.087465553	LOC114114842	-1.563968404
LOC114113979	2.085003883	PITX1	-1.563745915
E2F2	2.084534781	NRGN	-1.563327373
LOC114117581	2.083927457	QKI	-1.562497487
CEP55	2.082937444	COL24A1	-1.561431643
DYNLRB2	2.080198821	C12H1orf115	-1.56085341
LOC114117243	2.078768801	LEF1	-1.560589852
LOC101111382	2.077451124	LOC101116298	-1.558703163
KCNH3	2.075948257	COL6A2	-1.558504857
LOC114115318	2.075369752	SERPINF1	-1.55804638
APBA2	2.070836284	CALD1	-1.557939178
LOC105607270	2.070440528	RASA1	-1.557530404
SPP1_1	2.067863957	TANC2	-1.557299529
LOC114114137	2.064780097	MAP3K2	-1.556697307
LOC114112074	2.061481581	CBFB	-1.55656215
SRCIN1	2.056466626	ARID4A	-1.555554197
FIGLA	2.056187551	ADGRL4	-1.555267567
LOC114117130	2.05551402	RASAL3	-1.555173259
PBX4	2.053358443	VWCE	-1.555173259
IL11	2.052288983	ADCY2	-1.554473084
LOC105605098	2.050746307	LAYN	-1.554447195
ZYG11A	2.048327103	LOC114112115	-1.554027837
SALL3	2.048327103	SUFU	-1.553673257
COL8A2	2.04790745	TNS1	-1.553142875
MZB1	2.046516695	LOC101107504	-1.552610998
PMM1	2.045937392	IKZF3	-1.552610998
CCDC114	2.045905632	SESN2	-1.552526232

ARMH1	2.04480464	LOC105605783	-1.551941472
LOC101117431	2.043987856	LOC101104162	-1.551907501
LOC105607066	2.043987856	SMG1	-1.551108014
EFCAB10	2.04308303	SPATA13	-1.550741117
LOC105606267	2.04054155	CD2AP	-1.550607472
MBOAT4	2.040207074	CLINT1	-1.549735177
SLC25A41	2.040207074	TMEM19	-1.547128431
BOLA3	2.039777398	ZHX2	-1.546808901
CCDC96	2.035715069	SLC25A16	-1.546500997
LOC105613604	2.033938791	RCSD1	-1.546185366
RASL10A	2.033938791	GHR	-1.544651764
SLC6A16	2.033938791	KDR	-1.544473749
ESPN	2.030961533	PEG10	-1.542808297
MASP2	2.030289536	FAIM2	-1.542775733
PORCN	2.029596442	ALDH3B1	-1.54205833
MUC1	2.026111661	TMOD3	-1.540924202
LOC105607546	2.025707732	TIAM2	-1.540708381
LOC114115951	2.024988189	FAM107A	-1.539975827
C3H12orf60	2.023819511	FOXO1	-1.539889089
LOC105608416	2.022774291	ZBTB1	-1.538990237
CRTAC1	2.021751101	LRRN1	-1.538935723
SHISAL2A	2.021723756	LPAR1	-1.537872749
NCMAP	2.021524393	PMAIP1	-1.537744949
OPCML	2.021524393	SLA	-1.537744949
GHSR	2.021524393	ZNF608	-1.537692286
LOC101120904	2.019364559	ZNF292	-1.537427222
LOC101108414	2.01891927	CD14	-1.536793213
LOC101121590	2.017176581	SLC43A2	-1.536197554
TDRD1	2.015922621	ALDH1L2	-1.532825177
FSD1	2.015922621	ZCCHC14	-1.532475865
CHRNE	2.015440562	WASL	-1.532255063
LOC101102327	2.013133414	NEURL2	-1.532032122
ADAM11	2.012556014	SLC20A2	-1.531880796
LOC101102526	2.012271871	HSPA12A	-1.529877179
LOC105607146	2.010273644	FZD7	-1.52850051
LOC106990188	2.007321277	C18H14orf132	-1.528270314
AMDHD1	2.007043755	ROCK2	-1.527624613
LOC105615329	2.006544041	LIMS1	-1.52675003
JAKMIP1	2.005966069	ZKSCAN4	-1.52474049
MYO1A	2.005966069	IKZF2	-1.524500691
KHDC3L	2.003151117	MFSD14A	-1.524419137
LOC114114005	2.002751086	SLC30A2	-1.524173848
LOC114112790	2.00108126	AGAP2	-1.523933132
SPC25	2.001050164	LOC101109212	-1.52375584
DNAJC12	2.000587914	TPD52	-1.522909101

SLC6A20	1.999592623	SENP7	-1.522642449
NHLRC4	1.996380572	LOC101117112	-1.522451159
BRIP1	1.995682282	LOC101104028	-1.521239771
LOC105608691	1.995210507	LOC105602763	-1.520915298
FHAD1	1.991681488	LOC105603786	-1.520720649
LOC106990346	1.990297952	LY96	-1.519530283
GMNN	1.988850456	RHOBTB3	-1.51938768
PRR7	1.988804452	MEIS2	-1.518956961
LOC105613245	1.988390934	ASPH	-1.518872066
ZCCHC12	1.988390934	TCF12	-1.518856964
NR2E1	1.988021732	LOC101108696	-1.518782475
LOC105608012	1.987425649	TBC1D12	-1.518365466
S100A2	1.987075409	UBQLN2	-1.517880391
TMEM235	1.985259509	LOC101121414	-1.517351503
CDCA5	1.985008843	GAS1	-1.517288644
NOVA1	1.984754944	THSD4	-1.517262686
TNNI3	1.98355025	ANGPT4	-1.515753442
DAPK2	1.979751672	LDB2	-1.515671428
LOC105604659	1.979014825	LOC114118864	-1.515043379
CA3	1.978428565	PPP1R3D	-1.515033776
FOXA1	1.978074853	IRF8	-1.514870311
KCNJ4	1.978074853	PRND	-1.514594461
LOC114116664	1.978074853	RSPO3	-1.511620804
LOC114113685	1.977489457	GTF2A1	-1.511504602
LOC105616444	1.97699537	PAK2	-1.510574614
INHBB	1.976602662	LOC114111456	-1.509810651
KREMEN1	1.976404861	MMD	-1.509698348
LOC101118200	1.975396119	MAP3K13	-1.50871968
LOC101106374	1.974993071	TMEFF1	-1.508653605
LOC105606646	1.974458668	HELZ	-1.508557404
RBBP8	1.973651177	BIN2	-1.507957803
VWA7	1.973470221	LOC114109662	-1.507629315
LOC105602046	1.972571598	AHNAK2	-1.507177308
LRRC73	1.972571598	FZD4	-1.506246641
TM7SF2	1.97164895	RERE	-1.506192437
PKHD1	1.970500115	PLXNA4	-1.505580139
LOC114110120	1.968656752	ETS2	-1.504957085
LOC114110322	1.968193827	LOC105613077	-1.504439495
LOC114109433	1.967399928	TPSB2	-1.504304869
SERPINC1	1.967399928	ZDHHC23	-1.503675218
LOC114110282	1.96576729	LOC114110482	-1.503095736
LOC114111266	1.964242619	NCOA7	-1.502302713
LOC114114850	1.964242619	ACOT13	-1.500590265
APOF	1.964242619	LIG4	-1.499001691
LOC114108821	1.964242619	SLC35F1	-1.498915609

PROM1	1.963461005	FAM117B	-1.498434009
DEUP1	1.957445318	PCNP	-1.497595185
LCA5	1.954699277	LOC105602786	-1.497506044
RAP1GAP2	1.947148359	SPON1	-1.497332693
KCNQ4	1.946969256	PUM1	-1.49696543
LOC101116529	1.946969256	MORC3	-1.496841789
LOC114115734	1.944726118	CFAP97	-1.496723165
LOC105612395	1.944726118	LOC114115243	-1.495623766
LEKR1	1.944726118	PARM1	-1.49557061
LOC114117972	1.944048724	SERPINA14	-1.495261519
PERP	1.94387899	KCNE4	-1.495140979
RLBP1	1.941622783	SCYL2	-1.494244006
AHSG	1.940630873	MXRA8	-1.493970325
LOC105613405	1.939885651	FMR1	-1.493034283
LOC114113051	1.939885651	APP	-1.492889189
MDH1B	1.939885651	PCP4L1	-1.492540913
HSD11B1L	1.939296671	CREG1	-1.491966348
VXN	1.938004353	CHIC1	-1.491782605
HIGD1A	1.936782672	HTD2	-1.49066439
LOC114116108	1.93579556	ANKRD55	-1.490440009
G0S2	1.935427666	KIAA1549L	-1.489832888
RDM1	1.935092052	EEA1	-1.489645385
PHKG1	1.935028872	LOC114114903	-1.489363735
SH3YL1	1.933540009	FGF1	-1.489033466
LOC105603708	1.93225988	WBP11	-1.487753063
LOC114117038	1.929964309	PTPN12	-1.487199519
LOC101119869	1.9264031	DUSP10	-1.486977873
LOC105610181	1.924374397	ATF7IP2	-1.485428742
CATSPER4	1.924374397	LOC101110918	-1.485260833
LOC114113896	1.922865703	EOGT	-1.485238375
BDH1	1.9220932	RNF150	-1.484940098
LOC105613097	1.91900817	SNX13	-1.484429699
LOC105609801	1.918936654	CTSW	-1.484131766
TMEM179	1.918383574	WAS	-1.483839746
LOC105611269	1.916071847	HLF	-1.483231248
KLK10	1.915111684	CLCC1	-1.482813399
LOC114112419	1.915111684	SSPO	-1.482608986
LOC101117527	1.914533873	VWF	-1.482406926
CIR1	1.91320089	LOC114115554	-1.482375286
ZP3	1.911780016	TNFRSF19	-1.481508496
SKA1	1.911037796	ZNF280D	-1.480710949
LOC105610832	1.91078632	EGR3	-1.480443245
STPG1	1.908834976	ZMYM4	-1.480144041
ERBB3	1.908539758	ATF2	-1.477525669
BTG4	1.908211592	CPZ	-1.47733752

DPCD	1.908089567	LOC114117565	-1.477336844
RIPPLY3	1.907599561	CD3E	-1.476641546
LOC114110606	1.907516714	COL3A1	-1.476245696
POP1	1.906749692	ACTR2	-1.47517942
ACSL5	1.906634325	CDKN1B	-1.473218788
DEPDC7	1.9064675	AOX1	-1.472851956
OOEP	1.904767341	CENPI	-1.472065067
SERPINB12	1.904225764	WDFY3	-1.471840736
POU2F3	1.904225764	MSS51	-1.471740964
LOC114113223	1.904225764	LOC114111026	-1.471740964
NACA	1.902970949	RRP1	-1.470953099
HES7	1.901629833	LOC101108198	-1.46936362
CEP72	1.900861641	JAM2	-1.469022124
CACNA1S	1.900270884	LOC114117768	-1.469001595
SMC1B	1.899804727	DIO3	-1.468574831
LOC101122984	1.898759657	LOC114116109	-1.467750476
UBE2C	1.897505018	C1QC	-1.467701897
CXCL14	1.897472516	LOC114117956	-1.467287566
TMEM82	1.897408099	TCN2	-1.465431866
LOC101117485	1.897408099	FCHO2	-1.464879139
HES6	1.897408099	LRP1	-1.463403514
NDUFB4	1.8972315	TUBGCP5	-1.463105179
RANBP1	1.896990291	METAP1	-1.463066017
LOC105610729	1.896861482	MARCH7	-1.462041977
FEN1	1.895881198	JAML	-1.461940799
TNNT2	1.895814643	DENND6A	-1.459478748
ATP4A	1.894759038	KLF10	-1.459121212
MS4A10	1.892410862	MTUS2	-1.458131219
LOC105607532	1.892410862	LOC114117844	-1.457802585
LOC105604784	1.891268399	TMED8	-1.457523157
LOC114109650	1.890338102	USP32	-1.457469823
PNLDC1	1.88969062	ELN	-1.456688457
LOC114110470	1.887497296	DPP10	-1.455640725
LOC114113624	1.886723859	ALG2	-1.455087522
LOC114111513	1.8863084	PIP4K2A	-1.453748536
MYO18B	1.885203067	CACNA2D1	-1.453447808
BORA	1.885014683	PARD6A	-1.452396243
MZT2B	1.884961131	LOC101122210	-1.451823006
GTSF1	1.883829294	LOC101102402	-1.450622204
LOC114117225	1.88354049	SCUBE1	-1.449639706
LOC101117063	1.88172254	GRAP2	-1.449546396
TMEM262	1.881087947	ARRDC5	-1.449546396
UHRF1	1.880059106	FAM217B	-1.449503247
LOC105608566	1.879160089	LRRK2	-1.448524174
TRAF5	1.879046393	CCDC9B	-1.448505395

WDR17	1.877513451	ZC4H2	-1.448148267
DTYMK	1.877086185	NPR3	-1.447566644
DRD3	1.876991342	NGFR	-1.447375425
LOC101119941	1.876991342	MAP4K5	-1.447293304
LOC114116841	1.87286761	SLC25A19	-1.447055452
LOC105604257	1.872859789	COL14A1	-1.446861889
SLC16A11	1.871556578	TIMP3	-1.446433582
LOC114109035	1.87074872	CAP2	-1.446245988
CKS2	1.870070666	CPNE1	-1.445699235
NCKAP5	1.869476442	LOC114116826	-1.445263669
LOC105605336	1.867134146	ZCCHC24	-1.444899467
SPRED3	1.866116655	SGPP1	-1.4427912
FBXW9	1.866058581	BTBD3	-1.442597798
FYB2	1.864811209	RASSF9	-1.442522995
LOC114110304	1.864811209	LOC101104051	-1.442324505
SLC38A8	1.864811209	LOC114108679	-1.442046502
HDGFL1	1.864811209	BATF2	-1.441674848
DPYSL5	1.864811209	LOC105605761	-1.440876047
ETNPPL	1.864811209	TNFSF8	-1.440569645
LOC114117631	1.862441733	PALD1	-1.439679814
LOC101122314	1.860451685	ANKRD1	-1.43967012
LOC106991120	1.859434243	PCMTD1	-1.438513841
PAQR9	1.857384829	CXCL9	-1.438430007
ATP8B3	1.856568998	PRRG1	-1.436399965
LOC114116878	1.855690918	DPY19L3	-1.435924822
LOC105605744	1.854412149	TTC13	-1.435351013
STAC2	1.852113165	C2	-1.43514397
LOC114114032	1.849985804	KLF13	-1.433847565
TTC32	1.849854934	KLF12	-1.433139252
LOC101107463	1.849854934	CCDC149	-1.432200785
ARHGEF39	1.848183756	LOC114116081	-1.428601958
DNAJA4	1.848156505	PWWP2A	-1.428311478
TMEM177	1.844837078	DAB2	-1.427886321
LOC114109440	1.84409659	GGT5	-1.426735303
EPHB6	1.841306034	SMARCA2	-1.425965053
COL11A2	1.839438111	MINDY1	-1.425779732
LOC105611873	1.837613295	LOC114115664	-1.424641249
CSPP1	1.836355455	SP3	-1.424628037
LOC114113894	1.834764023	LOC114110274	-1.42416844
SLC2A10	1.834622775	LOC101111832	-1.423704984
LOC114117865	1.834422459	PCDH17	-1.422226509
FABP3	1.834037491	ACAP2	-1.421740739
ABCC8	1.831303659	CSF1R	-1.421497173
TRIM37	1.831244647	LOC101123290	-1.420981165
TM4SF18	1.83020401	CYTL1	-1.42068692

HTR1E	1.826391558	CTSF	-1.420244546
LOC105611403	1.826391558	VASN	-1.419557566
SHCBP1	1.823877226	UEVLD	-1.419525132
LOC100134870	1.823873084	POU2F1	-1.419461906
NOP58	1.823101624	CCDC112	-1.419300248
ARHGEF5	1.822800266	HSPA13	-1.418653862
LOC114118386	1.819791727	VAT1	-1.418637434
PIAS4	1.818664136	SYNGR3	-1.418533639
IFT22	1.818497987	LOC114117603	-1.418463348
LOC114115626	1.817910908	OSBPL1A	-1.418400142
LOC114116816	1.817910908	LOC101102156	-1.418174038
CASP3	1.817538578	PPM1N	-1.418056253
LOC105604613	1.817468052	BMP6	-1.417170783
LRRC1	1.815161425	CPEB2	-1.416239897
ANXA9	1.813938536	MPZ	-1.416030913
LOC114114494	1.811084384	HMOX1	-1.415762929
LOC105604728	1.810453797	PAQR3	-1.41472183
RPL6	1.80839419	FAM217A	-1.414263752
UPF2	1.804700798	LOC101107486	-1.414263752
LOC114110027	1.804507297	ALDH1A3	-1.414169759
LOC105602066	1.804092787	DCLK2	-1.413454027
LOC101116968	1.803619166	XPA	-1.413423238
LOC114116179	1.802943776	MOB1B	-1.41327926
SLC2A11	1.79841007	FAM199X	-1.412864297
AFG1L	1.79811976	IRX3	-1.412592637
MRPL40	1.797977583	LOC105603195	-1.411798171
LOC114109014	1.797382388	CMYA5	-1.411644078
SMIM1	1.79513655	TXNIP	-1.411048573
CTXN1	1.7936055	CBL	-1.410455433
CITED1	1.793186664	NAV2	-1.408984186
TIGD3	1.791018912	LOC101117087	-1.408603865
NETO2	1.790269739	MGAT4C	-1.407795882
PPP1R36	1.787350635	TUBA8	-1.407795882
LRP8	1.786740654	CASKIN2	-1.405603683
CCNB2	1.786094856	MFNG	-1.404775902
CYB561	1.785395081	LAMA2	-1.404204249
XRCC3	1.785248062	TGFB2	-1.404125966
SCEL	1.784121674	PTGIS	-1.403774401
TTLL8	1.784121674	MAML1	-1.40366248
SLC35B1	1.783232007	MYNN	-1.403505441
MTMR7	1.782193415	ARL13B	-1.403415863
FBXL22	1.779743598	APOD	-1.402646306
IGF2BP3	1.779013214	TAL1	-1.402141273
LOC101102399	1.778130578	PPIL4	-1.402014897
LOC101120961	1.778130578	PELI1	-1.401014221

HEATR4	1.778130578	IL6R	-1.40003626
C25H10orf71	1.778130578	CYYR1	-1.399113205
LOC105616794	1.776068437	NPNT	-1.397815487
ANKRD9	1.776013456	DENND4A	-1.397412221
LRR1	1.775649238	KCNE1	-1.396686976
LOC105602850	1.775504838	DTWD2	-1.396686976
LOC101102204	1.774891289	ABI3	-1.395412195
F10	1.772872714	LOC105603639	-1.394953974
LZTFL1	1.772719719	LOC114116368	-1.394953974
POU5F1	1.772020634	LOC101117600	-1.394953974
PRRG4	1.771353523	CTDSP2	-1.39472883
ALYREF	1.770166212	PDZD2	-1.394567293
WEE2	1.769998687	EPN2	-1.393545274
LOC101111915	1.76895414	DYNLT3	-1.393492709
TG	1.768845433	AFF1	-1.392629168
LOC114115593	1.768279411	MEF2C	-1.392085851
SAPCD2	1.765560664	LTB	-1.3919874
LOC114114099	1.762734527	CCNT2	-1.391293859
LOC101118245	1.759519278	C1D	-1.390861386
SCN8A	1.757437533	LOC105611784	-1.390646826
LOC101115685	1.756634597	ZNF385C	-1.390473551
SLC52A3	1.75577519	PRRG3	-1.389643507
LOC114117264	1.755179365	NIN	-1.38957025
PRSS12	1.7549451	RAB3IL1	-1.389383666
LOC114116624	1.754650227	TOR4A	-1.388910853
CCDC18	1.750963794	LTBP4	-1.38857726
LOC101110974	1.750963794	MTO1	-1.387251671
PYROXD2	1.750963794	HIF3A	-1.387251671
TMEM266	1.750791711	KIAA1147	-1.387020352
LOC114116429	1.750276732	GABPA	-1.385405893
POU2F2	1.75007467	HPCA	-1.38491804
PTPN3	1.750014586	PPM1A	-1.383939593
TMEM126A	1.745630095	LOC101106919	-1.382594493
DKKL1	1.745551472	AFF4	-1.382561271
LOC114115282	1.744329701	UHMK1	-1.382441157
KDELR3	1.742415014	NATD1	-1.381882854
LOC105610869	1.742347809	PILRA	-1.381709227
LOC114116381	1.742050383	CHM	-1.380817306
FBXO4	1.741634735	UBR1	-1.379261165
GRAMD2A	1.741310417	PSD3	-1.37802711
PSRC1	1.7406511	RAB30	-1.37785587
TTN	1.739787437	DZIP3	-1.376845659
RBFOX3	1.739107954	APPL1	-1.37677557
GABBR2	1.738547679	PRDM8	-1.375450748
LOC101107641	1.73614076	VEGFB	-1.374748094

LOC101116577	1.735344325	LOC101112335	-1.374715122
PSMA5	1.734466217	LOC101115808	-1.374544819
LOC105603538	1.733948239	LOC106990526	-1.374346584
LRMDA	1.732535529	AR	-1.373931005
LOC105608648	1.732535529	SNIP1	-1.373812871
HOXA3	1.731025617	MCCC2	-1.373789587
LEO1	1.72926595	AUH	-1.37342243
MOGAT1	1.726644939	MYLK	-1.373157681
TSACC	1.72621129	ATF3	-1.373078714
LOC105602007	1.725322148	WWTR1	-1.372984079
BUB1	1.725313101	G3BP2	-1.372964034
PNMT	1.723018307	SOX18	-1.372293374
PLK5	1.723018307	GASK1B_2	-1.37215521
SEZ6	1.722533798	NAAA	-1.371597747
CATSPER2	1.722533798	COL6A5	-1.371419945
LOC101116852	1.722533798	METTL25	-1.3712265
CFAP298	1.722073712	DEPP1	-1.369856403
RRM2	1.721597743	KANSL1L	-1.369737829
IQGAP3	1.72155522	JADE2	-1.369389328
COPS5	1.721143205	CAPG	-1.367925621
EFCAB7	1.719328684	TP53INP1	-1.36764104
GPR157	1.719009917	NCALD	-1.367588625
FATE1	1.717743543	DNASE1	-1.367248794
LOC101123632	1.714451501	NUDT12	-1.36716222
LOC114113864	1.71328349	PCSK1	-1.366574803
LOC105606461	1.713121131	DAB2IP	-1.366226808
TMEM238	1.712838333	COL1A1	-1.365504982
LOC114109443	1.712380191	ZBED8	-1.364777344
LOC114109595	1.712380191	MYZAP	-1.362192555
ANKRD45	1.710289586	LOC101105018	-1.362087477
CDC20	1.709266855	NRAP	-1.3618885
LOC114108997	1.708936898	FAM49B	-1.361779282
LOC101113705	1.708804561	AKAP12	-1.361291635
ACTA1	1.708445105	SMIM15	-1.36080526
REPS2	1.707617411	AHCYL2	-1.359643373
MOS	1.707614786	RFTN2	-1.359377157
TMEM151A	1.706229785	GNPNAT1	-1.359245969
SFRP4	1.706164228	ARHGAP31	-1.35908604
C16H5orf34	1.705336183	SH3KBP1	-1.359045567
ZNF827	1.70475908	LOC101110545	-1.35835763
LOC101106791	1.704311436	TBC1D4	-1.358203549
PCYT2	1.702735559	SOCS6	-1.357369115
CCDC138	1.70267074	RP2	-1.356352924
ANKRD31	1.702350664	GPATCH2	-1.356223851
ECI2	1.700976356	TMEM221	-1.355465161

ERCC6L	1.699965955	GPC6	-1.355006251
DDX39A	1.699558312	ND2	-1.354997858
LOC114115395	1.699417988	ADAM9	-1.354489281
RIPK4	1.697543038	BCDIN3D	-1.354339502
WDR5	1.696445421	VNN2	-1.3540521
SLC16A3	1.694479405	SNTB1	-1.353647818
CCNE1	1.691350539	TAF4	-1.353195095
SUV39H2	1.691287674	ALKBH8	-1.353166176
NUF2	1.69078861	SMC5	-1.353131169
LOC114113884	1.690755285	TIGD6	-1.352755638
C12H1orf112	1.690006288	RAB20	-1.352278801
LOC105603919	1.689980425	EDNRB	-1.352207016
LOC101102549	1.689980425	G2E3	-1.352207016
LOC105610030	1.688785622	UNC5B	-1.351996478
LOC101105754	1.683929225	CHD9	-1.351107744
ASIC4	1.683305402	TRPC1	-1.351098266
MYLK3	1.682735686	KCND1	-1.350229716
PCLAF	1.682270529	C1QTNF1	-1.349756542
INSRR	1.681435617	RAMP3	-1.348858965
LOC101103401	1.680952318	IDS	-1.348647173
CCDC150	1.679725252	LOC101109964	-1.347591942
LOC101117650	1.675650213	SLC30A1	-1.347544107
SH2B2	1.674208797	LOC105603132	-1.347114143
SCG5	1.674195298	PPP1CB	-1.346837616
NDUFB1	1.673736941	MGARP	-1.346347949
SPERT	1.673190738	LOC101122457	-1.346202927
LOC105605471	1.673190738	GNG2	-1.346191579
LOC114117284	1.673190738	ANKRD46	-1.345720862
CAV3	1.673190738	ZSWIM4	-1.345346358
CELSR1	1.671770126	PAOX	-1.345292769
FKBP6	1.671680671	ATRNL1	-1.344427075
LOC105607182	1.671590414	CLIC2	-1.3442343
POLE2	1.670979367	LOC105612077	-1.3442343
CCDC57	1.670913884	ADNP2	-1.344194024
LOC114116943	1.670517922	TSC22D3	-1.342511838
LOC114111231	1.669864859	RASAL2	-1.341612988
NSUN7	1.669852723	FAM89A	-1.34132898
OTX1	1.668850147	CELF2	-1.339130077
LOC114109668	1.668850147	TGFBR1	-1.338580829
CATSPERD	1.668850147	SCARF1	-1.338339149
C6H4orf48	1.668198884	FBXO30	-1.337200007
TMEM231	1.667772292	UST	-1.337060317
BRSK2	1.66774186	ASH1L	-1.336205472
LOC114114607	1.666547686	FIBIN	-1.335587836
CISD1	1.666378369	CDK12	-1.335501604

LOC105613853	1.664052596	UBR3	-1.335129731
MTFMT	1.663955458	EPS15	-1.334281359
LOC114117862	1.661913974	ELF4	-1.332720166
CBLN2	1.661890224	UAP1L1	-1.332660767
ZNF511	1.661410837	IQCK	-1.332399543
WDR34	1.66132043	FXYD1	-1.33218417
LOC105613248	1.661292534	LOC101109513	-1.332112792
LOC105615197	1.659552427	STXBP4	-1.331619644
MAT1A	1.657679554	PIK3CB	-1.331566686
APCDD1L	1.656866205	KIAA0355	-1.331480745
GPLD1	1.656538326	MAP3K7CL	-1.331046398
UNC5CL	1.654087073	ZFAND5	-1.330770683
LOC105604618	1.654087073	FAM135A	-1.330540666
ROGDI	1.652505488	SPATA5L1	-1.330363389
IGDCC3	1.65230964	RHCG	-1.330044764
CAMK2N2	1.65230964	SERINC4	-1.329361028
MANF	1.651976773	TGFBI	-1.329225202
SHROOM3	1.651960112	MDGA1	-1.328047378
LOC101111769	1.651732611	KCNC3	-1.328047378
NDUFA12	1.649217055	FNDC10	-1.327565486
PTTG1	1.647166242	STAU2	-1.327522711
MRPL55	1.646258539	LOC114114014	-1.326814683
CEP89	1.645643722	ACAT1	-1.326316964
CPVL	1.64548972	LOC101117299	-1.326233125
FANCD2	1.645295284	LOC105601981	-1.325789676
IFT88	1.644960943	SLC25A40	-1.325422781
LOC114116722	1.643008492	CD180	-1.325167997
CFAP36	1.639954152	QSER1	-1.324935759
LOC101118761	1.639285023	ZNF148	-1.324810051
LOC105612888	1.638953046	MAGI3	-1.324730915
BSPRY	1.637625527	MTURN	-1.324419909
GBX2	1.637196924	TCEAL1	-1.323532946
LOC105607239	1.635449285	SHE	-1.323349748
PCSK1N	1.633911667	LOC105614674	-1.323180886
LOC101110178	1.633809342	RNF168	-1.322799675
LOC105610886	1.632544679	BTBD7	-1.322331895
MRPS14	1.632525084	CRLF3	-1.322313384
LAMC2	1.631567965	CCSAP	-1.32174599
SQLE	1.631551655	LHFPL2	-1.321742372
ARMC2	1.631473124	LOC101109476	-1.321215887
TSSC4	1.631254491	CAAP1	-1.321093572
LOC114113056	1.629730467	PHTF2	-1.320539974
ETV4	1.629269343	CDH11	-1.320314638
ZC3H12B	1.626765737	LOC105603222	-1.319020351
LOC114114824	1.626765737	ABI1	-1.318613916

UPK3A	1.626765737	LOC101110605	-1.318591306
LOC105604916	1.626765737	LIMCH1	-1.318486019
RSAD2	1.626522388	ELMSAN1	-1.317859045
PAQR5	1.626446644	RHOBTB1	-1.317752434
MAEL	1.626446644	NUPR1	-1.317313925
TMEM258	1.625259261	PLD4	-1.317166868
LOC101109593	1.62223394	NGF	-1.316539524
CA11	1.621400963	RAMP1	-1.315906338
TCF19	1.620613401	EPHA4	-1.315663093
RNASEH2A	1.620410058	LOC105615308	-1.315422167
PET100	1.618898851	EPHB4	-1.315347701
CRELD2	1.618831182	HACE1	-1.314589215
PLA2G2F	1.618533372	LOC101113735	-1.313187834
CCDC71L	1.618533372	PTPN4	-1.312693487
LRRC4	1.618533372	PTN	-1.312606006
POLR2L	1.618023923	LOC114117771	-1.312536189
PRR15	1.617607302	NR1D1	-1.311737586
LOC114112817	1.616144611	CCPG1	-1.311692416
NUDT14	1.612191517	TTC33	-1.310974557
A4GALT	1.611095748	SMARCC2	-1.310837886
LOC114117577	1.610836614	HIVEP3	-1.310809293
ZWILCH	1.609021015	ASPA	-1.309796891
LUC7L3	1.608390066	TET1	-1.309105142
LOC101121054	1.608315165	KLF6	-1.30854425
PROB1	1.607184527	LOC114118453	-1.307412709
FGF9	1.606215106	HIPK3	-1.306880549
DHCR7	1.60621131	NRIP1	-1.30675676
SMKR1	1.60593663	HOXA6	-1.306676866
LOC114111075	1.60483361	NOX1	-1.306676866
EFEMP1	1.603178554	LOC114108820	-1.3066065
KCP	1.602810073	LOC114117254	-1.306475423
LOC105603956	1.602500129	ZBTB44	-1.305994632
C6H4orf19	1.602223251	ULK1	-1.305981255
PIF1	1.601402806	FBN1	-1.3056807
AURKA	1.601067262	TTC14	-1.305135908
COL17A1	1.600868731	IREB2	-1.305096192
LOC101109027	1.600392608	PICALM	-1.304390041
CDCA2	1.599750124	ATG14	-1.304024039
RHBDL1	1.598915993	TCFL5	-1.302693095
MEFV	1.597922917	SLC15A4	-1.302068918
LOC114117886	1.597922917	LOC101121244	-1.302065391
LOC105616394	1.597922917	SHLD1	-1.301899546
RHEBL1	1.597922917	FTH1	-1.30181451
PWWP2B	1.59691528	NOTCH2	-1.301683884
BNC1	1.596664992	LOC101122306	-1.301558767

LOC101118470	1.596664992	AEBP1	-1.301071615
KIF22	1.596315103	ITPR1	-1.300679938
TVP23B	1.595886744	UTP15	-1.300425862
CEP83	1.595071823	KDM6A	-1.300324762
PRC1	1.595030061	RCAN3	-1.299858004
B3GALT6	1.59392068	TMEM167B	-1.299514488
CRIP1	1.593032487	SLC16A9	-1.299321512
GNL3	1.59293615	SDCBP	-1.29925107
LOC101107098	1.591505671	LOC105607533	-1.29872423
WFIKKN1	1.591199887	AJAP1	-1.298521443
BCHE	1.588672787	GMFG	-1.297510636
LOC114110673	1.588591297	RASA3	-1.29705043
LOC114114820	1.588557332	TLE4	-1.296619822
MYO19	1.587967542	DNAJB9	-1.296466434
NTMT1	1.587043281	BRWD1	-1.296420328
E2F1	1.586558377	PDZRN3	-1.296106921
APOBEC2	1.584676393	TANC1	-1.293843754
LOC114116058	1.584377546	ANXA13	-1.293753568
EPOP	1.583952767	TXLNB	-1.293393342
AP3M2	1.582097552	LOC114110472	-1.292633165
SNRPD1	1.581020998	TCAF2	-1.292554398
NFIB	1.579979402	SNCAIP	-1.29253253
LOC114109333	1.578260746	LOC114108710	-1.291419002
LOC105603595	1.578260746	FTL	-1.29132944
ZCCHC18	1.578260746	GAB2	-1.290918033
AIFM3	1.578260746	MICAL1	-1.290201966
LOC105606270	1.578260746	ZNF503	-1.290140618
THEM4	1.578251585	LOC101109939	-1.288760113
SGO1	1.576792155	ATP13A3	-1.288483685
LOC105605988	1.576643155	C1QL1	-1.28830528
CCNA2	1.576321222	TNFRSF21	-1.288080501
MED11	1.57510867	RUSC1	-1.287602308
ZSCAN12	1.571364177	YPEL2	-1.287483175
CCDC170	1.57104356	ANGPT2	-1.287396646
LOC114114030	1.570603121	SERINC3	-1.286902346
KLHL41	1.570603121	ARHGAP12	-1.2862575
LOC105613348	1.570603121	ATG13	-1.28508922
LOC114116384	1.570603121	UBE2H	-1.284514313
LOC105608026	1.569885278	LOC114108630	-1.284424721
LOC105604543	1.569720216	LOC101114275	-1.284287455
EXOSC4	1.569713877	SYT1	-1.282360942
NPM3	1.568545366	LOC101108987	-1.282274562
LOC101111664	1.567746998	PRPSAP1	-1.281983365
LOC101112257	1.567323371	JMY	-1.281392651
REEP4	1.567217302	PRKAR2B	-1.281293688

TPX2	1.566226053	ADAMTSL1	-1.279925451
LSM4	1.565632142	RCBTB2	-1.279522014
LOC114114613	1.563996537	POLR2A	-1.279501156
ADGRF1	1.563996537	RECK	-1.279067355
RPH3A	1.56318934	FUCA1	-1.277673404
GALE	1.562831945	BNC2	-1.277450595
ADGRG5	1.562810588	RNF144A	-1.277200259
CALML4	1.561610494	TACC1	-1.276776575
PUS1	1.561459335	ARHGAP42	-1.276659538
LOC105607267	1.56040053	CYTH4	-1.276618731
LOC101121036	1.56040053	LOC101117013	-1.27645406
CCNF	1.560167463	ZNF70	-1.276332179
DCC	1.560050432	TRIM23	-1.275831844
LLGL2	1.559705392	CREBL2	-1.275688711
SDF2L1	1.559506154	SLITRK5	-1.275084845
HSD11B1	1.559458	LRRC2	-1.27496453
IGSF5	1.558316413	LOC101116597	-1.274737455
TMC7	1.558238444	UBTD2	-1.273571247
LOC114114548	1.558238444	RERG	-1.273557809
AK6	1.555170301	GABRA3	-1.273001212
LMNTD1	1.554574329	DUSP1	-1.272715128
RNF208	1.55446212	KMT5B	-1.272686793
ZMYND15	1.553175208	CTTNBP2	-1.272243952
LOC105613123	1.553175208	LOC101122689	-1.270865629
MYO16	1.553175208	LOC101114063	-1.270046569
FBXO5	1.552389869	RASSF2	-1.269836727
CMSS1	1.550336693	USP53	-1.269136489
LOC114109667	1.549933707	RGMB	-1.269081395
JSRP1	1.549628209	TSHZ3	-1.269060467
PRDX5	1.54923348	TRIM35	-1.268918928
KIF24	1.544853166	LOC114113853	-1.268805478
PSD2	1.544684283	BST1	-1.268003115
SLC15A1	1.544684283	TM4SF1	-1.267361895
PSMB9	1.544107647	LOC101114033	-1.266264972
OVAR	1.543949566	KMT2C	-1.265925757
ALDH1A2	1.543866037	KDM5A	-1.265598965
LSM7	1.542515642	WDFY1	-1.26528842
ALOX12	1.542145197	ADAMTS7	-1.264489136
LOC114118103	1.541870289	SCIN	-1.264401431
LOC114115556	1.541259043	LSP1	-1.264167375
KIF2C	1.540203162	DNAJB4	-1.261952796
ACSL6	1.539667496	MEGF9	-1.260881428
ENO2	1.538245857	GAB1	-1.259802985
OVOL2	1.537844042	RASGRF2	-1.259336274
TRAIP	1.536746214	RDX	-1.25869711

FOXRED2	1.536719191	ITGA3	-1.258407043
ANKRA2	1.535138111	FAM43A	-1.258171724
PCDH12	1.534316559	LOC114110426	-1.257886375
PLCXD3	1.534210672	FRMPD3	-1.257886375
LOC114112591	1.532900267	MAP6	-1.257886375
MROH6	1.532724229	CD300E	-1.257886375
DZIP1L	1.532653664	PRKAG2	-1.257769295
TRIP13	1.532410972	SPRYD7	-1.257662909
SOGA3	1.532215716	LOC101123010	-1.256729319
LOC114113252	1.532215716	LOC105610137	-1.256303628
B4GALNT4	1.530855476	ATP2A3	-1.25603283
APLN	1.530345291	ME3	-1.256027483
UTS2R	1.529063232	PANK3	-1.255964277
CFAP54	1.528861345	ARL6IP6	-1.255940093
CREB5	1.528352997	FAM151B	-1.255572231
ATP5ME	1.528102375	LOC101106719	-1.255403986
TSHZ2	1.527378516	LOC101103413	-1.255033035
MRPL17	1.526879166	PARD3B	-1.253873933
ITPKA	1.526662012	DIAPH2	-1.253588109
TEDC1	1.526586438	AXL	-1.253459482
LOC105605319	1.525423592	TGFB1	-1.253397548
ACSBG1	1.524975682	UTRN	-1.252113551
LOC101106086	1.523997583	TLN1	-1.250612406
LOC114116349	1.523500284	TTBK2	-1.249318771
PRDX1	1.523040752	PDS5B	-1.249294086
MRPL18	1.521214954	ZFHX2	-1.249008286
LOC105612939	1.51790391	LAMA1	-1.248441651
WDR31	1.517223409	ARHGAP15	-1.246742271
LOC101112491	1.514439894	BMP2K	-1.246015758
LRRC45	1.513466133	EXOC8	-1.245920766
TMEM54	1.512623243	BCL9L	-1.245151444
LOC114113182	1.512118463	ZNF281	-1.244349546
MYL6B	1.51202946	LOX	-1.243974871
IL1R2	1.511937035	ATP6V0A2	-1.243927215
LOC105609232	1.511903056	CBFA2T3	-1.242950961
SPINT1	1.511580121	NNT	-1.242866388
PSMC3IP	1.511222778	GALNT16	-1.242174633
TMEM158	1.510580115	PLBD2	-1.242070959
TIMM10	1.510346096	ST3GAL6	-1.241932969
NEURL3	1.509099605	FAM120C	-1.24192381
TRMT112	1.508979647	LTBP3	-1.241461054
IGSF9	1.506527091	EFNB2	-1.241390837
LOC106991659	1.506292017	PREPL	-1.241259116
HS3ST1	1.506021815	RAB21	-1.241231883
NEXMIF	1.505013461	LOC101105208	-1.240872219

FAM181B	1.504981964	RUSC2	-1.24072777
NFIC	1.504634899	FSD2	-1.240557043
GNAZ	1.503434881	ITIH5	-1.240525718
PYCR1	1.502096819	LOC101110160	-1.240177207
KIF17	1.501781472	MTMR12	-1.239580735
LOC101118990	1.500666282	KLF7	-1.239473226
LOC114116344	1.499437032	CCNG1	-1.238427122
MPP2	1.498261104	PLPP3	-1.23771604
LOC106991044	1.495925731	TMEM173	-1.237668914
LOC114109439	1.495687418	PXK	-1.237452308
CCDC30	1.494424394	ZKSCAN8	-1.237267886
LOC105613171	1.493636467	SPHK1	-1.236963203
LOC114113921	1.493369838	PADI2	-1.236702198
SRP19	1.492927605	PKD2	-1.236491385
KIFC1	1.491738878	LOC105607754	-1.236488384
SLC23A1	1.491383762	ATG12	-1.235475919
ACP6	1.491038209	GSTP1	-1.234601507
EIPR1	1.490184646	INSR	-1.233879247
SLC4A8	1.48960116	LDLRAD4	-1.233425803
TONSL	1.488859637	SNN	-1.232963524
C20H6orf52	1.487302772	TIAM1	-1.232853281
LOC114117762	1.487048372	LOC101104855	-1.23279001
SNRNP35	1.484171496	FSBP	-1.232483321
CCND2	1.483874834	RAB8B	-1.231904365
NRIP2	1.483863237	RASGRP4	-1.231768436
DSN1	1.48360298	SKAP2	-1.231639557
FBP1	1.483283456	NCOA3	-1.231591046
SYNGAP1	1.4814984	AMOTL2	-1.230837016
UBE2T	1.480138315	LOC114113923	-1.23062312
MIF	1.479573608	FILIP1L	-1.229458101
SOCS1	1.478363561	COX2	-1.229307964
TIRAP	1.478280808	UBE4B	-1.228855802
SOX6	1.476278901	SHOC2	-1.228583751
GNL2	1.475773237	THADA	-1.227823259
RPS19BP1	1.474143238	CSRNP3	-1.227454719
VRK1	1.473925177	VPS26C	-1.22707904
HEPH	1.473713772	ADAMTS1	-1.226218332
LOC105606460	1.472867343	ZNF646	-1.225906413
FAM184B	1.472573925	CA2	-1.225687652
SBSN	1.472376978	ZBTB2	-1.225502574
GMPR	1.472279509	YAP1	-1.224253042
DHRS7B	1.471179349	TJP1	-1.224170223
LOC114108699	1.470852332	RTN4RL2	-1.22264328
RTL10	1.470761827	LOC105615181	-1.22264328
ARL14EPL	1.470100334	CSGALNACT2	-1.221898743

LOC101114167	1.470100334	PAM	-1.221363188
OLIG1	1.469821206	PLSCR1	-1.221246546
LOC101119597	1.468199179	MARCKS	-1.221193387
LOC114112697	1.467591792	NAPEPLD	-1.220959989
LOC101120174	1.466850077	TRAPPC8	-1.220598003
LOC114116077	1.465923932	VSIG10	-1.218151964
MPDU1	1.465833119	RAD54B	-1.217956455
LOC114116376	1.464734798	SLC25A23	-1.217941002
KIF27	1.464591008	SIRPA	-1.217504345
RRAGD	1.464182756	PALM	-1.21746708
FAM184A	1.463158396	VIPR2	-1.21744828
LOC114112473	1.463152125	CHST7	-1.217150538
LOC105613829	1.463152125	LOC105604472	-1.21686337
C2H8orf58	1.462998585	FBLN7	-1.214860573
K38	1.462146653	SNX14	-1.213213459
LOC101103752	1.462146653	STRN	-1.213184193
KATNB1	1.461436301	RHOG	-1.212967707
KCTD19	1.460941811	STX7	-1.211161347
CCNB1IP1	1.460941811	RASD2	-1.211087499
ASTE1	1.460246951	CHML	-1.210675111
MRRF	1.460190596	LOC106991366	-1.210675111
HSF5	1.459471773	FSTL3	-1.210653949
SLC10A5	1.458771299	TOP1	-1.210530332
KPNA2	1.458725624	AKAP11	-1.210433061
PRAG1	1.458020351	TRIM45	-1.210283199
LOC105606408	1.457638155	LAMC1	-1.210188071
SLC26A3	1.457638155	ATP8B4	-1.209902299
LOC114114839	1.457638155	PER2	-1.209450711
ZBTB32	1.457638155	LOC101107282	-1.209208704
LOC114116917	1.457638155	VTI1A	-1.209080691
IL13RA2	1.457638155	ADGRF5	-1.208976655
LOC105602391	1.457638155	SEC22C	-1.208354718
CDNF	1.457621386	ZNF319	-1.20784617
LOC101104866	1.457312477	SLC7A8	-1.20753472
KIFC2	1.456950752	LTBP1	-1.207378125
LOC101113893	1.456403229	PDE7B	-1.207195664
HMMR	1.455882794	MBP	-1.207063295
BAIAP2	1.454776302	DLK1	-1.20682296
SCML2	1.453043467	ATP11C	-1.206751568
LOC105606441	1.452255843	KLHL5	-1.206606644
FAM167A	1.451951518	CEP162	-1.206210579
SGSM3	1.451334045	ZDHHC17	-1.20619307
LOC101120455	1.451157599	TLR4	-1.205792816
LOC114116409	1.449724659	LOC101110593	-1.205070552
UFSP1	1.448137045	SLC35F5	-1.204219027

LOC114114004	1.447825784	NPEPPS	-1.204185931
SELENOS	1.446361953	LYSMD1	-1.203005206
LOC105604469	1.445362078	PTP4A1	-1.202795857
C4H7orf25	1.445311683	LOC114109664	-1.202167645
LOC101108158	1.444953524	HTR2B	-1.201934954
SLIT1	1.444861394	ATXN2	-1.200990607
MRPL22	1.441084696	LOC101106330	-1.200899613
NDUFA3	1.440683037	SLC35D1	-1.200351898
CDH23	1.440396842	PCYT1A	-1.200252652
TEX30	1.439277162	DCP1A	-1.19942132
NHP2	1.437324172	SEMA3A	-1.199242088
C26H8orf48	1.436109617	TET2	-1.199122584
RASSF10	1.435899168	CPNE5	-1.198756416
LOC101116315	1.435619352	CHST2	-1.198529877
CHAF1A	1.43484772	HDAC11	-1.198455335
FANCF	1.433940881	PKMYT1	-1.198186277
ENTPD2	1.433830024	KIRREL3	-1.197267375
LOC114113163	1.433492394	RTN4R	-1.19698153
KCNH1	1.43319744	FOXD3	-1.196858075
LOC101110771	1.430193878	LOC101117683	-1.196743966
GALNT6	1.430068659	MED9	-1.19618222
ZBTB48	1.428767295	VMA21	-1.195847614
LOC114118418	1.428189825	LAMP2	-1.195292822
TTLL11	1.427169567	IGFBP3	-1.195291443
PCTP	1.427034248	CDKN1A	-1.194989624
HNRNPA1	1.426459062	LOC114110477	-1.194887926
LTV1	1.426416423	TTC23	-1.194855796
LOC114110833	1.425887683	C20H6orf62	-1.194248347
SRM	1.425860215	CLSTN2	-1.194217972
NOCT	1.425523748	NEFM	-1.194116488
SLC25A29	1.425255034	LOC101108528	-1.194011258
LOC101115605	1.425147281	KLHL33	-1.193961239
LOC114114091	1.424854556	NCOA6	-1.193365038
TMEM161B	1.424840488	FAM214A	-1.193120688
TMEM198	1.424407097	LOC106991292	-1.192981572
LOC114113940	1.423916251	ZC3HAV1L	-1.192024585
TYMS	1.42376015	KAT6A	-1.190959601
RGS9BP	1.420901759	CRYBG1	-1.190824198
SELENOH	1.420729232	RAD21	-1.190674662
CDKN2D	1.420655788	PABPC1	-1.190435531
PNP	1.420448641	PDGFRB	-1.190116392
THYN1	1.420166543	RCOR3	-1.189377962
TESC	1.420081869	PLEKHO2	-1.189079373
ELF5	1.419995458	TTC8	-1.188210842
GAS8	1.419441136	PXYLP1	-1.188130626

LOC101105090	1.419257794	TMEM64	-1.187768008
SEL1L3	1.419112115	LOC101114226	-1.187195814
RUVBL2	1.417933534	APOBR	-1.187124947
CENPO	1.41715314	KIAA1324L	-1.186559016
LOC101114442	1.417111098	CNIH1	-1.186007905
ANKRD29	1.416339287	ZFYVE1	-1.185648453
DIPK1C	1.416106958	NMRK1	-1.185494228
DNAJC24	1.414221031	FOXK1	-1.184847882
LOC101106384	1.413829923	MYSM1	-1.184613386
PIGF	1.413470701	CEP170	-1.184459131
SLC39A8	1.41272119	LOC105611518	-1.184293402
ABHD11	1.411364799	LOC114112646	-1.183948174
POLD1	1.409654833	PRDM1	-1.183709704
DCDC2	1.40959599	PARP8	-1.183373522
SEM1	1.409241586	USPL1	-1.182394986
KCNK5	1.409209662	SEMA4D	-1.182151822
LOC114109697	1.408557192	PHF20	-1.181945934
SRSF7	1.40836525	CPD	-1.181656713
GPT	1.408199426	ZNF507	-1.181006882
CDC25A	1.408080607	JAZF1	-1.180720801
SERP2	1.407597341	ZBTB24	-1.179702719
LOC114116071	1.407264611	LOC101107224	-1.17948147
TNK1	1.406552997	VEGFC	-1.1787871
SKIDA1	1.406374952	FAM222B	-1.178685755
LOC101103616	1.4063444	LAMA4	-1.177400204
BNIP3	1.405590697	LOC105613374	-1.177133219
ACTG2	1.405349032	JADE3	-1.176559458
MCM4	1.405042575	FANCC	-1.175623087
MRPL42	1.40480139	LRRC40	-1.175623087
TTLL3	1.404576254	PODN	-1.17506002
LOC105602957	1.404100713	FNDC5	-1.172863285
INSIG1	1.403512341	LARP6	-1.171918095
TMEM205	1.402676307	SELENOI	-1.171842452
TNFRSF4	1.402655369	TMTC2	-1.170296823
MRPL23	1.402035929	NAA30	-1.17018079
LOC101111670	1.401263529	SEC24A	-1.170179897
CCDC187	1.400975528	PARS2	-1.170160352
C11H17orf53	1.400777318	ZHX3	-1.169964086
LOC106990841	1.398383278	SLC25A36	-1.169193746
NUCB2	1.397125257	ACO1	-1.168960832
DNAAF4	1.397013563	PPP1R18	-1.168900957
NCAPG2	1.396273581	MCTP2	-1.168810304
SNRNP70	1.39616874	NSMAF	-1.168614668
NCS1	1.395708937	TMEM43	-1.167703591
TRPC6	1.39514397	ARIH1	-1.167439684

CETN2	1.39451919	TMX3	-1.167351189
HMGB3	1.393594316	ZNF704	-1.167309737
GNB3	1.392298896	MAPK8	-1.166626457
LOC105604827	1.39099399	SPARCL1	-1.16647569
DNAJC17	1.390895534	LOC105610122	-1.166182142
UBL4A	1.389989685	PCGF2	-1.166052179
LOC114117325	1.389406159	AKT3	-1.165942152
LOC114115330	1.389406159	SGK1	-1.165706079
FLRT1	1.389406159	METTL15	-1.165107541
TMEM104	1.389218522	ABAT	-1.164901996
LOC101106806	1.388417085	LHX9	-1.16417111
LOC101109079	1.388197759	HOMER3	-1.163459498
ORMDL2	1.386676006	SPRED1	-1.163253445
EFHC2	1.386050641	MBD6	-1.162380205
ST3GAL4	1.384739248	MEF2D	-1.161955193
MAB21L4	1.384576001	RAP1B	-1.160971725
DLX4	1.384576001	EFNB3	-1.160830693
LYRM2	1.384507989	SHC2	-1.160630607
LOC105610932	1.382989776	METTL24	-1.160356247
GPD1	1.382964355	HSPB6	-1.159997713
LOC101109652	1.37916283	USP20	-1.159753995
TRDN	1.378678429	ANKRD12	-1.158249929
MPZL2	1.378451062	LOC101112038	-1.157914487
SUMF2	1.378309392	LOC101123275	-1.157436197
LOC114110160	1.377384924	SH3BP5	-1.156972786
ALG8	1.377381383	PHYHD1	-1.155708889
PDCD2L	1.375849909	ZNF654	-1.155666358
NR1H4	1.375206262	TIPRL	-1.155211542
LRRC4B	1.374312746	PRDM11	-1.154938361
LOC114110432	1.3741834	RAB12	-1.15449741
GPX4	1.37371026	ANKIB1	-1.153680525
CELF4	1.373520791	FAM210B	-1.153465403
TACC3	1.373390585	TM6SF1	-1.15332966
HES2	1.373138469	ZBTB33	-1.153286822
LOC114112974	1.373138469	SHBG	-1.15315644
NCAPH2	1.373065481	NDRG1	-1.152885526
VWA5B2	1.373054263	ZNF326	-1.152363296
RAD51	1.372573642	PDGFD	-1.151899912
ARMC6	1.37064374	RGS2	-1.151474724
LOC106991954	1.370422689	CHAC2	-1.151324173
ATL1	1.370422689	TRMT1L	-1.151187843
RELT	1.370019238	NLRC3	-1.150734361
PPIA	1.368779904	FAM129A	-1.150661943
LOC105612914	1.368728701	GLUL	-1.150502681
LOC114115366	1.368654257	DLG1	-1.149997246

LOC105608579	1.368614183	SLC36A4	-1.149557549
SEPT4	1.36799222	MBNL2	-1.149443707
LOC114110119	1.36713687	MCMBP	-1.14937049
PCP4	1.366230006	LOC105609869	-1.148722791
LOC105609197	1.366107037	ELOVL4	-1.148722791
OBSCN	1.366107037	ZNF423	-1.148330096
LOC114111298	1.366107037	SLC39A6	-1.148270283
LOC101116228	1.365539009	SEC31B	-1.147574365
LOC114114087	1.365061105	SEMA4C	-1.146951874
MND1	1.36434302	ZSWIM6	-1.146950029
NUDT17	1.364135386	ACKR3	-1.146898831
HCN4	1.362745363	RNF115	-1.146140438
LOC105605453	1.362362852	LAMB1	-1.145983008
LOC106991918	1.362204877	ARHGAP29	-1.145851246
LOC114113868	1.361610644	OLFML1	-1.145771148
LOC114112968	1.361205568	ZBTB45	-1.145677422
TMEM144	1.360298277	PLD2	-1.14564505
REC8	1.360008367	SH3TC1	-1.14426827
DCTPP1	1.359844367	ABHD5	-1.143902344
MT3	1.358580399	ARRB1	-1.143463869
UNC5A	1.358135609	ZNF521	-1.143438343
LOC105605439	1.358135609	STMN3	-1.142731668
LOC105603399	1.358135609	PRXL2C	-1.142616353
LIPT2	1.357158304	DDX3X	-1.142047059
CIART	1.357061087	FCHSD2	-1.141728615
LOC114111037	1.356297649	ACP2	-1.141215808
DOLPP1	1.356266435	PTPN23	-1.14104299
PCNA	1.356134503	SIPA1L2	-1.140955899
ZC2HC1C	1.356039948	DUSP3	-1.140319295
BIRC5	1.355144555	DPY19L1	-1.140264377
LOC101123341	1.355129578	LOC114116116	-1.140228734
MSMO1	1.354865392	RENBP	-1.140000228
PSMD8	1.354586679	ARHGEF26	-1.139120468
ACOT7	1.354564212	ZCCHC9	-1.139022406
PRF1	1.353244809	COL6A3	-1.13826098
SERPINB5	1.352247305	ASXL2	-1.13726345
TNNT3	1.352247305	DCN	-1.13724895
LOC114118505	1.352205985	TSPAN11	-1.137135532
LDHD	1.35151932	BORCS7	-1.136911507
CNKSR1	1.351415367	FERMT1	-1.135622149
ZFHX3	1.350492666	PUM2	-1.135444484
MRPS25	1.34922627	CHRD	-1.134424131
CDO1	1.34906549	VPS41	-1.134149272
LOC114110821	1.34889531	SGCE	-1.134125361
LOC114113871	1.348834747	KLF5	-1.134025186

LOC114118092	1.348834747	PDE4D	-1.13385687
LOC105603395	1.348813327	LARP4	-1.133418371
CDC25C	1.346075757	INPP4B	-1.133325615
SEPT5	1.346036753	PECAM1	-1.133291088
EYA1	1.345180389	LOC114110585	-1.133008486
CSPG5	1.343483573	SBK1	-1.132871097
HEMK1	1.343343587	DGKB	-1.13190382
LOC106991580	1.34322795	INO80D	-1.130289598
MCRIP2	1.342778513	LOC106991947	-1.129992673
TMEM184A	1.34160986	GASK1A	-1.128974815
ADGRL3	1.341330673	DLC1	-1.128863142
C7H15orf48	1.340661392	LMOD1	-1.128049985
LETM2	1.340461327	ERBIN	-1.127686494
LOC106991767	1.34025068	SOCS5	-1.12767773
LOC114113240	1.339794091	ANKFY1	-1.127541964
LOC114116389	1.339514986	RPL30	-1.127435635
SOWAHB	1.339136023	ZNF385D	-1.126983216
CLCA2	1.338056735	BMPR2	-1.125256718
LOC105609364	1.338056735	EVI2A	-1.124803463
PCDH11X	1.338056735	NPTN	-1.124480128
SLC8A3	1.338056735	STAT3	-1.124439091
LOC114117523	1.338056735	ACTN2	-1.12359823
LOC114116647	1.337457303	LOC114110265	-1.123240881
CHP2	1.336191987	CMTR2	-1.122902746
LAMA3	1.335757063	CNEP1R1	-1.121874792
LOC101111166	1.335342903	KIAA1841	-1.121475297
MITD1	1.335327129	FBXL2	-1.121452467
ALPK2	1.334774422	MARS2	-1.121202699
RARS2	1.334654071	EIF3F	-1.121125403
LOC114116646	1.333656603	ITSN1	-1.121064941
TSSK3	1.333475146	PTPDC1	-1.120377821
HSD17B1	1.33325873	ABCG2	-1.119939237
ZNF114	1.333073961	PRKAG3	-1.119904975
NUDT1	1.332908821	RNASEL	-1.119572102
LOC114113953	1.330957361	CGNL1	-1.119455937
ADRB1	1.330957361	CABLES1	-1.119221486
DNASE1L3	1.330957361	FCGR1A	-1.118202441
FGB	1.330943475	LOC105605937	-1.118042107
CDCP1	1.330626924	HEXA	-1.117938738
SNRPD3	1.329724913	RUBCN	-1.117732237
TRAPPC6A	1.328809301	ZNF469	-1.117320534
TIMM17B	1.328441083	IKZF5	-1.11702961
SIGLEC11	1.328267566	RAPGEF5	-1.116646355
E2F7	1.327109208	PLA2G7	-1.116129269
ENKD1	1.326968193	SPX	-1.116086275

LOC105606525	1.32685256	IKZF1	-1.115533076
LOC114114912	1.3263699	LOC101122718	-1.115478498
SLC7A9	1.325884605	CYP17	-1.115316477
PNPO	1.325832451	LOC101117099	-1.114633145
CCDC106	1.325396617	GPSM3	-1.114419686
LOC114110579	1.325165546	TTC17	-1.114060601
DUT	1.3250131	ADAM12	-1.114055784
MALSU1	1.323992295	CYTH3	-1.11341263
LOC101118849	1.323930294	CLASP1	-1.113338997
CLPSL2	1.323322973	MERTK	-1.113328109
CCDC84	1.322612399	SMAD3	-1.11317075
LOC101119721	1.322473104	MTA3	-1.112472004
PPA1	1.322167939	P3H2	-1.11147966
THOC3	1.322067139	RIOK3	-1.111267277
SLF1	1.319381855	LDLRAD3	-1.110529066
LIN7B	1.319226889	LOC101107455	-1.110390741
LOC101109728	1.319095295	SOCS2	-1.110281012
EME2	1.319071026	IL33	-1.110205989
ROMO1	1.317826694	LRRC25	-1.110094314
RPS26_1	1.316531855	LOC105603910	-1.109875737
LOC105604882	1.316409999	ANKRD50	-1.10941956
LOC106990432	1.315908497	LOC106991502	-1.108958982
LOC114114888	1.315908497	LOC114109555	-1.108534039
PRKCG	1.315908497	UHRF2	-1.108267363
LOC106991991	1.315741611	LOC101110181	-1.108109536
RABL2B	1.315680431	RAB33B	-1.107974215
UPP1	1.313815656	BMPR1B	-1.107498906
SNRPA	1.312647103	ZNF703	-1.107103883
NCAPD3	1.311232126	STYX	-1.106804222
CEP44	1.311156992	NCF2	-1.106566558
BTC	1.310475548	SOX8	-1.1064159
DHH	1.310475548	OTUD4	-1.106046031
LOC105616708	1.310286942	FYCO1	-1.105906849
SUSD3	1.310202005	ZNF367	-1.104743727
KIF11	1.309777096	ZDHHC20	-1.10347768
ANO9	1.30957697	ZNF274	-1.103363249
LOC105608615	1.30957697	TNKS	-1.103272666
ZC2HC1B	1.30957697	HYKK	-1.103199527
FGF16	1.30957697	MAN1C1	-1.10248799
LOC114115603	1.30957697	IMMP2L	-1.101955349
LOC105607786	1.309312529	SLC29A4	-1.101596287
COL6A6	1.308977075	ITPRID2	-1.101454412
F5	1.307761071	KIF3A	-1.100759062
HHIP	1.307515956	RMND5A	-1.100670145
LOC114110278	1.307132083	FN1	-1.100363712

LOC101102485	1.30690272	FEZ2	-1.100242312
HSD11B2	1.306489789		
SLC39A3_2	1.30431715		
CPXM2	1.303754929		
RDH11	1.303421299		
HDAC10	1.302667151		
PLCXD2	1.302016725		
LOC105602034	1.301953369		
LOC101113583	1.300576625		
LOC105601929	1.300039933		
ZMYND19	1.299031659		
KCNB1	1.298304009		
MED29	1.298300311		
ARMC9	1.297711826		
CDRT4	1.297381786		
ATP10B	1.297324725		
KCNK1	1.296777475		
PPCDC	1.296717883		
UNC13D	1.296606612		
LOC101118481	1.295835243		
CD1D	1.295266795		
LOC114111323	1.294979466		
MCM5	1.294972447		
LOC105611592	1.294285782		
FGFR1OP	1.29426025		
PIM2	1.293634177		
CCDC14	1.293326941		
ANKEF1	1.292545705		
LOC114108830	1.292545705		
PIH1D1	1.292091932		
ALB	1.291861302		
RPS28	1.291296782		
HYLS1	1.289508019		
SERTAD1	1.288879791		
PUM3	1.286771971		
PLK1	1.286480418		
KIF23	1.286103405		
C7H15orf61	1.286072863		
DRP2	1.285967976		
RSPH3	1.285640021		
LOC114110588	1.285128654		
SNRPF	1.284863192		
TPRN	1.283306551		
NKX3-1	1.282742385		
LOC105609854	1.282308869		

TMEM132A	1.282211962
NOL7	1.281379628
SLC25A13	1.280630036
LRRC3B	1.280329298
LOC114116360	1.280277258
METTL26	1.279855192
DGKG	1.279399095
SURF2	1.279365104
HMBS	1.279356865
NLRP3	1.279023933
BCAS1	1.277712435
LOC114112643	1.277250588
ZCWPW2	1.276971079
CFAP126	1.276906608
PITPNM3	1.276403372
LSR	1.275674839
LOC114116341	1.275341896
SHROOM2	1.274925072
NLRP9	1.274580578
LOC114109630	1.274169568
METRN	1.273792525
TNFRSF11B	1.273792525
LOC101111922	1.272879132
ACYP1	1.27280419
GMPPA	1.272315938
LGALS1	1.272260174
FGF14	1.271965944
MYO7B	1.271191333
MYO15A	1.271191333
ARID3B	1.270625296
CWF19L2	1.270565034
LOC114110424	1.269937543
IFT27	1.269459969
LOC101113599	1.269194304
PGBD5	1.267203521
LOC106990101	1.266680576
SNX20	1.265753973
NDC80	1.265260621
ANKRD37	1.263068152
COQ10B	1.26245746
ENPEP	1.25982884
KIF20B	1.259813992
LSM1	1.258890951
LOC114110099	1.258282119
PURA	1.258270813

VAMP1	1.257108272
FAM111B	1.257108272
LOC105611804	1.257108272
CCDC88C	1.256725736
COQ7	1.25596892
RDH5	1.254205577
ZFP69	1.254178627
IFT20	1.25409879
PTGES	1.252983679
LOC101115083	1.252274693
CD3EAP	1.252074756
UPK1B	1.251813567
CD164L2	1.249517698
HSCB	1.249309127
SNRPB2	1.248834709
LOC114115272	1.248645808
PNOC	1.247845363
ACY1	1.245946574
KHDRBS3	1.245836837
LARGE2	1.245809866
MMP17	1.244004949
TCTEX1D2	1.24384752
MKI67	1.243291782
LOC101107465	1.243026988
PPP1R3E	1.242984831
STX8	1.242700159
MTG1	1.241531372
LOC101111026	1.241333405
LOC101105010	1.240170852
MRPL24	1.239263485
CDKN3	1.239056076
CLEC1B	1.238370646
LOC114110797	1.238370646
POLN	1.238370646
NOP56	1.238017901
LOC106990546	1.237761332
LOC101118433	1.237275729
LOC105616457	1.23715591
CDC45	1.236709098
LYRM1	1.236256888
DUSP8	1.236209165
LOC114112987	1.23554957
OCLN	1.234945453
LOC114113858	1.233656318
TRIM66	1.233634421

CAMK2B	1.233634421
CCNE2	1.233387037
RNASEH1	1.232461726
LOC105611406	1.232096716
TECR	1.23180114
ADHFE1	1.231700215
ZNF502	1.231700215
FKBP2	1.230518717
CEP170B	1.230312866
RIOX2	1.230189924
LLPH	1.229363393
CCDC134	1.228756252
LOC114118718	1.228529167
ABHD18	1.228218391
MGME1	1.227829706
STMN2	1.226924146
MTERF2	1.226894597
MAPK13	1.226831482
LOC101111911	1.226223621
LOC114110614	1.225993159
FDXR	1.225497043
TH	1.225321207
ART3	1.224743291
NXT1	1.224224378
MTX1	1.223452541
SLC1A7	1.2215128
TNFAIP6	1.2215128
TRMO	1.221149002
LOC105603282	1.220386675
RPL26L1	1.219031795
BPGM	1.218589968
NELL1	1.216748497
MATN4	1.21656601
KLC2	1.21601096
GARNL3	1.215499663
CRYM	1.214908076
CNTRL	1.211093139
RWDD2A	1.210532029
TST	1.210169148
CD320	1.209898334
ZFAND2A	1.209539662
S100A14	1.209305112
NDUFS8	1.209287844
RABL6	1.208975662
PHTF1	1.207966594

RMND1	1.207516463
LOC114109632	1.207170805
MVD	1.207059024
MVK	1.206607567
ADCK1	1.205575257
CASKIN1	1.204929647
INHA	1.20466448
HEPACAM	1.204651755
LOC106990930	1.204455056
CHST11	1.204414732
PSMB1	1.204360109
PRRT4	1.204254528
LOC114113995	1.203494983
POLR2J	1.201037076
KMT5C	1.200321155
LOC114109707	1.200304394
CENPA	1.198733598
BLOC1S2	1.198195553
LOC105603432	1.197491287
LOC106990143	1.197476299
NSFL1C	1.197202242
EMID1	1.196974266
ARHGAP44	1.196800121
SPOCK1	1.196525431
ATP5IF1	1.19643072
LRFN1	1.196358269
GALNT3	1.196214882
LOC101111397	1.196001321
TRAF3	1.195829547
LOC105604599	1.195517027
RUBCNL	1.194608867
EEF1AKNMT	1.194275474
LOC105603436	1.193979396
FAM216A	1.193853204
LOC101112559	1.193709914
NUTF2	1.193706896
C3H9orf116	1.193559557
KIF15	1.193549301
NOTUM	1.193355964
MRPL30	1.193228883
TOMM40	1.192857282
APOPT1	1.19241417
LOC114118398	1.191339991
TATDN1	1.191086366
CREB3L4	1.190601877

GGT7	1.190542544
B9D2	1.189691705
KIF4A	1.189112925
CDC6	1.188833647
EXO1	1.188443497
RFC3	1.188254236
GINS3	1.187768544
MCP1	1.187031423
ABHD6	1.187018611
ABCD1	1.186662159
SLC27A5	1.186440397
EXOSC9	1.186308407
SKA3	1.186170553
LOC114110097	1.186168261
LOC114114012	1.186156518
BRINP2	1.184435305
IMP4	1.184085815
DEGS2	1.183685341
DYNLL1	1.182217599
ESYT3	1.179274066
LOC101116389	1.179274066
SLC7A3	1.179274066
LOC105603509	1.178567011
NDUFA5	1.177153846
LOC101111868	1.176595
ZSWIM3	1.176171803
SHROOM1	1.175491211
CARS	1.175306269
LOC101122545	1.174678034
LOC105608946	1.174534803
TTLL5	1.174383199
SHD	1.172214275
LOC105608435	1.172214275
MYCBPAP	1.17200668
LYPD6B	1.170955472
ENO1	1.17064569
MED8	1.170259358
ATP5MC1_2	1.169625205
LOC101103584	1.169515197
FUS	1.168889728
CDCA8	1.168687136
ATP1A3	1.168467014
TMEM35B	1.168337585
LOC114118362	1.167125347
C26H4orf47	1.166729073

C24H16orf91	1.166667092
SAMD13	1.163893398
SLITRK4	1.163854255
CFI	1.163674378
EZH2	1.162714893
LOC105609220	1.16249671
VMO1	1.161872357
LOC114113859	1.16166825
ABCA4	1.161539599
LOC114118397	1.160908343
MUS81	1.160681822
ZGRF1	1.160679575
NMRAL1	1.160325729
MEST	1.160177642
MCM2	1.159738202
USP2	1.159346168
GRAMD1B	1.158817962
BRMS1	1.15825605
LOC106991070	1.158202946
HPCAL4	1.15791532
LOC114108659	1.157858691
PGAM5	1.157014273
CSNK1G2	1.156474962
PSME2	1.15600399
C11H17orf107	1.155677707
POP5	1.155634782
NEK2	1.155364591
EMC2	1.154300468
C2H2orf72	1.15418176
LIPC	1.153944497
SAMD11	1.153862179
CDC16	1.151766359
DXO	1.15139753
TDP1	1.151170819
FAM162A	1.150550285
FBXW7	1.150404029
LENG9	1.150255687
LOC114113000	1.150187418
GXYLT2	1.149969434
GTSE1	1.149301855
TICRR	1.148507622
PPIL3	1.148416859
LOC105603426	1.148196073
UFD1	1.148108772
UBOX5	1.147832869

PLAC9	1.147777223
LOC101117458	1.14680844
CCK	1.146383144
PKD2L1	1.146383144
FAM71E1	1.146383144
LOC101119459	1.145343484
ZW10	1.145291376
MTCP1	1.144399226
LOC105611303	1.144399226
CERS3	1.144399226
SIVA1	1.1426049
HAUS6	1.14216904
ENTPD5	1.142128635
PAGR1	1.141646646
PLA2G2C	1.14148919
BRCC3	1.141205342
PSMG3	1.140982787
PTGER4	1.14019427
MRPL36	1.140185059
DNAL4	1.13988962
COL7A1	1.137494086
NAT10	1.137347672
WFDC3	1.137317312
PINX1	1.136173398
SLC20A1	1.136093866
PLCG2	1.134690013
RRS1	1.134593569
GRHL1	1.13371435
KIF14	1.133627186
MIOX	1.132725159
CFB	1.132239717
CAMSAP3	1.132067074
TSR3	1.131898028
CMC1	1.131389865
PSMB3	1.13131659
UGGT2	1.131286158
C17H12orf65	1.130953117
LOC114114542	1.130129236
ELMO3	1.128771469
RGS7	1.128144159
CCDC136	1.127897634
TBC1D31	1.127687273
PTBP2	1.127675403
NLK	1.127071094
LOC105603244	1.126742821

GIPC2	1.126678854
SGPP2	1.126554418
E2F8	1.1265378
LOC114112963	1.126512394
OXSM	1.126432535
HHAT	1.124964126
PDSS1	1.124538967
MTERF3	1.123938619
RAB6B	1.123442928
CCDC59	1.123173127
CCDC28B	1.123015102
RIC3	1.123006427
GMPPB	1.123000073
NANS	1.122804675
LOC101121216	1.122800544
FAM110A	1.122717263
HIGD2A	1.12268102
LOC105602864	1.121223308
GRB7	1.12069674
EMP3	1.12015999
HASPIN	1.119378151
RHBDL3	1.118565163
LOC106991883	1.11852122
ATOX1	1.118498529
ZNF628	1.118466135
LOC105601873	1.118274738
LOC105603863	1.117931598
STARD10	1.117294617
POLM	1.117229792
SRSF2	1.11718461
NAA38	1.116999748
ARHGAP11A	1.115908945
ST7	1.115820265
TJP3	1.115769092
YIF1B	1.114878042
ADI1	1.114684892
DMRTA2	1.114460551
ZNF473	1.114370125
CYP2J	1.114267821
MICOS13	1.113608733
ING2	1.112608728
RPP38	1.112422153
LOC114113938	1.111376801
DIPK1A	1.111182041
LOC114108811	1.111124322
LOC114117767	1.110786745
NASP	1.110415963
LOC101110116	1.110088998

ARMC4	1.110088998
LOC114116676	1.109049149
NNAT	1.108950924
ZNF584	1.108938018
LOC114109073	1.108906967
NCAPG	1.108886773
LOC114114490	1.108682333
EWSR1	1.107242634
NDUFB9	1.107058266
RIN1	1.106889729
DDX20	1.106122929
IMP3	1.106090108
ATP5MF	1.105964561
LOC101107381	1.105358417
LOC114116669	1.105112696
XRCC2	1.105074241
SMIM26	1.104842291
SOD1	1.104639011
XKR8	1.103959108
PTCRA	1.10362474
MRPL34	1.103218134
LOC114110037	1.102006225
CHEK1	1.100578583
