# Supplementary material for: Ovarian activation delays in peripubertal ewe lambs infected with Haemonchus contortus can be avoided by supplementing protein in their diets
Source: BMC Vet Res. 2021 Nov 3;17:344. doi: 10.1186/s12917-021-03020-7 (PMC8565066; doi:10.1186/s12917-021-03020-7)
Supplement: Supplementary file 5 — Additional file 5. Full list of differentially expressed genes in supplemented infected vs control infected groups-converted. [file 12917_2021_3020_MOESM5_ESM.pdf]

Ovarian activation delays in peripubertal ewe lambs infected with *Haemonchus contortus* can be avoided by supplementing protein in their diets

Paula Suarez-Henriques, Camila de Miranda e Silva-Chaves, Ricardo Cardoso-Leite,

Danielle G. Gomes-Caldas, Luciana Morita-Katiki, Siu Mui-Tsai, Helder Louvandini

Additional file 5. Full list of differentially expressed genes in supplemented infected vs control infected groups-converted

Up-regulated FDR p-value < 0.05 **Down-regulated FDR p-value < 0.05**

Gene ID	Log fold change	Gene ID	Log fold change
LOC114108772	13.44933118	LOC114111207	-13.18399101
LOC101121777	12.77918542	MGAM	-12.31905165
LOC114113596	12.06126329	LOC114113605	-12.14560596
LOC114114910	11.9961834	LOC114114475	-12.07372978
ADAD2	11.69792094	LOC114116592	-11.9392055
FOXR1	11.40980676	LOC105605968	-11.71995455
POU5F1	11.39064134	LOC114114036	-11.56380878
FIGLA	10.98059255	LOC101114750	-11.19321822
CSMD1	10.9612366	MAGOH	-11.16976964
NOBOX	10.76683504	LOC114113045	-10.98954597
LOC105607182	10.71384677	LOC101120732	-10.86853867
LOC114113671	10.68267039	DIO1	-10.8440577
LOC114109667	10.4245256	LEPR	-10.74704651
MYT1L	10.41464805	LOC114108756	-10.44239521
LOC114114905	10.37662558	SCN7A	-10.3824584
LOC101113054	10.30677591	CCDC190	-10.23208908
LOC114112675	10.30677591	SOSTDC1	-10.1465888
CCDC42	10.27577307	LOC105603423	-10.13048385
MED4	10.25472808	LRRC15	-10.10598424
TMSB15B	10.21169412	LOC114116112	-9.206068764
HSPA1A	10.189686	ASB10	-7.867907138
LOC114110588	10.15603123	COL8A1	-7.651651372
LHX8	10.14463621	FNDC1	-7.002518178
OPALIN	10.12157257	LOC101114319	-6.812386483
KLK12	10.09813422	LOC101122744	-6.788432441
CDHR5	10.0743088	LOC105607367	-6.74974722
FBN3	10.01296478	PRKG2	-6.62432056
PLA2G1B	9.974865984	CLDN1	-6.498707568
CORIN	9.948896101	LRP1B	-6.436629848
ASTL	9.935733723	ACAN	-6.329546166
LOC101111769	9.935733723	LOC106991302	-6.304916187
MAPK15	9.854133293	LOC114117879	-6.296297272

GLT1D1	9.840072955	KCNA6	-6.281536796
LOC114115662	9.840072955	MAP6	-6.1972481
STK31	9.825874237	LOC114114489	-6.054501745
DNAH10	9.797050575	CAV3	-6.018806383
FMR1NB	9.782419876	C1QL3	-5.978200413
SEZ6L	9.737615902	SPINT1	-5.940757532
LOC114109057	9.691375787	LRRC58	-5.821742686
LOC114109046	9.643604407	ELN	-5.750748467
ESRP1	9.627322508	RNF183	-5.746120782
MOS	9.627322508	SUSD5	-5.708184079
TDRD5	9.627322508	CLCA2	-5.670022311
LOC101105265	9.627322508	PI15	-5.653518471
HS3ST3A1	9.610854757	LOC105602432	-5.631968899
FRMD5	9.59419686	GRIP2	-5.506311418
LOC114109676	9.577344376	CDH8	-5.47400988
CYP19	9.507894046	SLC6A2	-5.469414066
KCNC2	9.489996203	HOXC10	-5.421492123
SALL3	9.453520308	TRIM66	-5.371502964
LOC106990690	9.416098212	LOC105612842	-5.345396253
HAL	9.377679515	MCF2L2	-5.301113602
PCLO	9.377679515	LOC114113180	-5.278516322
ATP1A3	9.358079573	C7	-5.242055614
FATE1	9.358079573	LOC101117431	-5.22863565
LOC101111382	9.358079573	CDA	-5.202770795
OXTR	9.338209681	PRR15L	-5.159475064
ADAD1	9.318062299	LOC114116917	-5.14299534
SKOR1	9.318062299	LOC101118459	-5.126731447
LOC101102411	9.318062299	GLP1R	-5.10316644
NLRP14	9.318062299	LOC114111483	-5.088767129
RSPH14	9.297629568	ITIH4	-5.078595108
CCDC105	9.276903287	SORCS1	-5.059999556
AWAT1	9.2558749	FMOD	-5.042140741
ADRB3	9.2558749	LOC101108519	-5.029797283
FOXJ1	9.212875653	HNF4A	-5.015874685
DPEP1	9.122830229	LTBP2	-4.994008221
LOC114112978	9.122830229	SCG3	-4.957079172
DCHS2	9.122830229	LOC101103260	-4.880046157
LOC114109518	9.104684282	LOC101107282	-4.871506495
DDX25	9.075608319	SLC8A2	-4.85147736
ATP2C2	9.075608319	MFSD4A	-4.851342674
TMEM52B	9.051404804	ANOS1	-4.816991776
LOC105610274	9.026788298	LOC114117319	-4.815314039
GABRG2	9.001744461	RGL3	-4.798666782
LOC101103165	8.92389385	CCDC150	-4.798666782
LOC114108622	8.92389385	SEMA3E	-4.762216223

LOC101109652	8.92389385	LOC114112975	-4.754032986
SALL4	8.92389385	BMX	-4.739688167
RGR	8.92389385	SRPK3	-4.730413392
HRK	8.896981253	TRIM63	-4.694535544
LOC101117443	8.869557059	LOC105614868	-4.694535544
LOC105615770	8.869557059	SLC6A17	-4.673939725
LOC101120595	8.869557059	SGCA	-4.64902021
LOC105603234	8.869557059	FN1	-4.630027763
LOC114109005	8.813093395	HOXD10	-4.602141398
LOC114114062	8.813093395	TNC	-4.561805542
LOC101119111	8.813093395	LOC101122263	-4.553271984
LOC101117493	8.784010646	LOC105611006	-4.548402341
TRIM67	8.754329547	SBSPON	-4.544805989
GAP43	8.754329547	P2RY12	-4.542859544
ACTL8	8.754329547	LOX	-4.541726605
LOC105604251	8.724024958	SFRP1	-4.536741041
CLEC1B	8.693070121	LOC114117807	-4.513248664
UBE2U	8.693070121	SERPINF1	-4.504756302
LOC101120961	8.693070121	COL24A1	-4.489456704
MAEL	8.661436519	SELL	-4.475275356
LOC114115688	8.661436519	RET	-4.45968754
GABRA5	8.661436519	GPR34	-4.455716065
PIWIL2	8.629093715	RYR2	-4.443749735
NR0B2	8.629093715	MFAP5	-4.436534665
LOC114110304	8.629093715	LOXL4	-4.42328131
RBMXL2	8.629093715	SNX20	-4.420233763
LOC114111539	8.629093715	LOC105613001	-4.417321487
DNAAF1	8.629093715	STMN2	-4.398470486
KRT77	8.629093715	SIGLEC1	-4.398432165
LOC114110833	8.629093715	LOC105612542	-4.39771997
TRIM50	8.629093715	HRCT1	-4.394862863
RHOV	8.59600918	LOC114113140	-4.391818387
NUTM1	8.562148092	LOC114117594	-4.390028598
LOC101103335	8.562148092	LGI2	-4.372760534
BFSP2	8.562148092	LOC114110252	-4.359209178
SLC10A4	8.562148092	LOC101122488	-4.352256482
CPB2	8.562148092	THSD7B	-4.33095962
CCDC188	8.52747312	AP5Z1	-4.328251433
LOC101103644	8.52747312	CCDC9B	-4.326434821
DSCAML1	8.491944172	NPPC	-4.304261532
RTL10	8.491944172	KAZALD1	-4.29930421
CRH	8.491944172	LOC105605457	-4.287500664
LOC105604203	8.491944172	PLA2G5	-4.280525776
LOC114112907	8.491944172	LOC114113974	-4.267192267
TCL1A	8.491944172	CRLF1	-4.266662931

LOC105604801	8.491944172	LOC114110654	-4.259220254
LOC114115291	8.491944172	CCL1	-4.231704766
LHFPL4	8.491944172	PLCD4	-4.231704766
SLFNL1	8.491944172	NOXO1	-4.231704766
WNT7A	8.491944172	CCDC80	-4.221781249
SOHLH1	8.488292104	LOC101106041	-4.212166965
MLN	8.455518121	FHL5	-4.187298485
BNC1	8.418148487	SH3RF2	-4.181223607
LOC105603863	8.418148487	CDH13	-4.177255656
FAM71F1	8.418148487	OSCAR	-4.155304801
GPR84	8.418148487	CFI	-4.148234878
CRYBB2	8.418148487	HBB	-4.139814769
CCDC181	8.418148487	DLK1	-4.134690946
ACHE	8.418148487	LOC101103244	-4.1315134
LOC101118216	8.418148487	ADRB1	-4.128911818
CNTN3	8.379785082	THRB	-4.128717833
MYPN	8.340373603	KCNA5	-4.123762002
LOC114115313	8.340373603	ANGPTL1	-4.1001006
SHISAL2A	8.340373603	LOC105603244	-4.095968211
LOC106990997	8.340373603	CHI3L1	-4.090856996
ELANE	8.340373603	LOC101119226	-4.081395176
LOC105608334	8.340373603	CCN3_2	-4.079001701
LOC101109545	8.340373603	NR4A3	-4.074631619
KRT28	8.340373603	LOC101102227	-4.074631619
CCDC87	8.340373603	LNPEP	-4.073985315
DNAAF3	8.340373603	LOC105616533	-4.06293882
PABPC1L	8.299855174	LOC114115282	-4.061032999
RBFOX1	8.299855174	LOC114114528	-4.060630572
GSTO2	8.299855174	ADGRL3	-4.057560564
SMIM32	8.258165809	LOC114113918	-4.056857459
GBX2	8.258165809	LOC105616901	-4.053392505
CERS3	8.258165809	AOC2	-4.046705956
LOC105601887	8.258165809	LOC114112092	-4.046705956
LOC101102519	8.258165809	STS	-4.036218521
CRYBG2	8.258165809	LOC114114563	-4.006919355
LOC105612031	8.258165809	LOC105606212	-4.006919355
RNF208	8.251541415	SLC38A5	-4.006919355
INHA	8.062709998	PKP1	-4.006211888
GCG	7.956999461	LOC101117706	-3.999125951
ZAR1	7.555178824	LOC106990096	-3.993698409
ZP3	7.522251795	LOC114117243	-3.989178729
LOC101117395	7.490597194	RPL3L	-3.989178729
NPM2	7.402969572	LOC101107463	-3.989178729
MINAR2	7.401501955	IL17B	-3.987402431
IHH	7.355141382	LOC101113122	-3.986851894

CD164L2	7.293297909	FGF2	-3.982800204
PHACTR3	7.248924904	LOC114114043	-3.979515934
LOC114115650	7.144464004	HTR2B	-3.976795184
CKM	7.104203843	FCER1A	-3.959531395
NLRP5	7.096788434	GHSR	-3.959531395
LOC106990495	7.092048172	LOC114110089	-3.955579549
TPT1_1	7.057802182	LOC114113034	-3.929262004
JSRP1	7.053540041	PRDM16	-3.902932162
GALNT14	7.020459415	TIGD4	-3.902790947
LCN8	7.019119191	TMEM71	-3.901651199
ACTL7B	7.003941434	LOC114113672	-3.900530417
LOC101109939	6.960439858	PDE3A	-3.900199069
ZP4	6.90207521	HAND1	-3.89834389
CCNO	6.868710934	CASQ2	-3.89834389
ATG9B	6.86326852	LOC106990575	-3.89834389
LOC105609277	6.772816906	SGCG	-3.897208228
LOC105604950	6.744914276	CCN3_1	-3.896235168
FAM167B	6.52948361	LOC101108520	-3.887257425
LOC114116088	6.527677593	S100A1	-3.887081326
GPX2	6.442092471	DPT	-3.875852601
LOC101111664	6.421703691	LOC101105989	-3.875135398
HSD17B1	6.353286646	FOXC2	-3.86336977
SLC47A2	6.324838815	LOC114110605	-3.857566615
KHDC3L	6.310948231	TGFB3	-3.852439499
TAFA3	6.300199556	VAT1L	-3.850319224
FAM83A	6.27737725	EFHD1	-3.84959404
LOC105613214	6.245206339	SLIT3	-3.839958297
LOC101121371	6.240731146	LOC114113913	-3.834445924
LOC114114998	6.221002824	FBN1	-3.831844411
AMH	6.196871357	CNTN2	-3.825656975
CHADL	6.196386318	NGFR	-3.820425895
LOC105607956	6.145856216	HTR1B	-3.817167354
ZGLP1	6.10460407	LOC101112671	-3.816726071
KCNJ5	6.09349187	LOC114115665	-3.813283965
CCDC60	6.067150426	GFRA1	-3.803073725
B3GNT4	6.067150426	LOC105608837	-3.801403337
LOC114113051	6.020363422	ND6	-3.801403337
GALNT9	5.966131317	GPR156	-3.795883899
LOC114113227	5.930700038	GXYLT2	-3.794303225
OOEP	5.902078038	FOSB	-3.791375059
LOC101106720	5.90159786	NFASC	-3.791254308
INSRR	5.893137465	CDH7	-3.7831464
UTS2R	5.875258328	MCTP2	-3.774809017
PADI6	5.824817962	MYOM1	-3.774792721
LOC114118734	5.824754927	LOC114108991	-3.774778517

FST	5.821499631	HOXC11	-3.774736207
ELAVL3	5.800328099	LOC101123341	-3.772828997
LOC114115359	5.798455722	TEKT3	-3.758691402
SLC1A6	5.729425292	SLITRK6	-3.758007628
LOC114113186	5.679449627	PARM1	-3.755090684
NELL1	5.665973214	BCL2	-3.753273905
LOC101106086	5.612965211	LOC114110464	-3.749938924
LOC105610869	5.553374924	RAMP1	-3.748655615
BSPRY	5.551658817	SHISA3	-3.742539533
LOC101111397	5.538661544	LOC114114545	-3.734960805
GSTA1	5.533447692	LOC101122501	-3.731423478
BBOX1	5.53063043	CHAD	-3.72357918
LOC105610712	5.509971623	HRC	-3.709389967
STC2	5.479923843	REEP1	-3.707662561
LOC105602721	5.465641216	LOC101104661	-3.703523561
PIP5KL1	5.443310877	LOC114115405	-3.700943259
LOC114109675	5.422144186	LOC114109650	-3.700943259
MYBPC3	5.400877253	SHOC1	-3.697476713
FAM163A	5.377522891	LOC114114537	-3.697476713
LOC114109433	5.37022511	RBM20	-3.693886546
LOC101123158	5.350363849	BTC	-3.693827906
LOC101121518	5.330732324	CCN4	-3.693161082
INSL3	5.317405443	RGS5	-3.691450474
MAPK8IP1	5.269466892	ACTC1	-3.688443543
KIF17	5.269038115	LY75	-3.686162056
RHBG	5.254079565	GJC3	-3.662386803
ARL14EPL	5.251013726	PODN	-3.65832319
NTRK1	5.232151311	SRL	-3.654715217
CXCL14	5.22614568	CNTN1	-3.653893837
SH3GL2	5.203929354	LOC101111069	-3.644402186
F2	5.193848873	LPCAT2	-3.636445835
LOC106991580	5.188998477	TMEM38A	-3.634875423
LOC101106541	5.171736783	LOC114118858	-3.63111142
LOC105607437	5.171736783	LOC114110816	-3.63111142
ZIC3	5.148636616	LOC114108767	-3.63111142
LOC114116813	5.148636616	LOC114114600	-3.629576659
GCNT3	5.141890073	DQA	-3.629465511
CLCNKA	5.134418648	CYB561	-3.628352539
LOC105608949	5.116356971	PI16	-3.624803741
LOC101111695	5.114358647	FAM180B	-3.624136881
PLPPR1	5.112729075	LOC106990432	-3.623785379
MSMB	5.10447332	LOC101109482	-3.623785379
LOC114112981	5.102887947	LOC101104401	-3.616575402
LOC105601897	5.095729226	ADRA1A	-3.611713054
LOC114108759	5.071189065	NTRK3	-3.609981061

HKDC1	5.052722296	DDR2	-3.60472935
SNX22	5.052722296	LRRC39	-3.600232118
IL17RB	5.052240261	ATP6AP1L	-3.599715369
LOC114108618	5.046030128	KBTBD11	-3.595956633
MGARP	5.044132607	FGF7	-3.593738411
LOC114113648	4.985804382	PROX1	-3.590151044
LOC114109368	4.973960092	FGL1	-3.584298649
TTC36	4.936269422	EFEMP1	-3.583086861
LOC101102259	4.936192049	SOX7	-3.580377363
LOC114117971	4.927169804	PTGER3	-3.57598506
KLK4	4.927169804	KCNMB1	-3.573113601
LIME1	4.903849245	LOC105610709	-3.57225392
LOC114116367	4.890648389	TNFSF13B	-3.566096623
GDF5	4.885985729	LOC114108725	-3.56508682
DNAH11	4.882154277	LOC114110615	-3.560245545
LOC105602330	4.866050849	RGS20	-3.558362595
LOC114109349	4.866050849	TNFSF8	-3.551433056
LOC105612144	4.866050849	LOC114114280	-3.546126399
LOC114114534	4.866050849	LOC105604745	-3.546126399
COLQ	4.860806713	SERPINB5	-3.546126399
LOC101122984	4.855576481	CELA1	-3.546126399
LOC101118990	4.854861775	FZD6	-3.539412272
NLRP9	4.836903622	POPDC2	-3.534864136
MYL6B	4.814085189	PRRX1	-3.533314686
NLRP13	4.809065389	LOC114117356	-3.529993646
FOXL2	4.804626827	SNCG	-3.525343555
LOC114115315	4.80222865	LOC114116179	-3.51911901
LOC105616091	4.802227638	DOCK8	-3.518765904
WNK3	4.778025507	LOC101123419	-3.515718296
LOC105605002	4.759195515	TPSB2	-3.514421842
LOC101121119	4.751054551	HCAR1	-3.513005519
RIPPLY2	4.735449743	FBLN2	-3.509623316
ZACN	4.730273651	TMTC1	-3.498764083
GTSF1	4.713309259	LOC114110437	-3.498107576
SHANK2	4.7080352	DDO	-3.498107576
SHISA8	4.696107013	HOGA1	-3.49672865
LOC101104943	4.692904017	RASA2	-3.494158759
PRRT3	4.692904017	LOC114116078	-3.481155937
LOC101108295	4.690275348	FAM151A	-3.480408298
LOC114112724	4.685574112	LOC114111010	-3.480408298
KCNE1	4.669282262	LOC114116827	-3.480408298
MMP1	4.668565365	LOC105607091	-3.480408298
HYDIN	4.665430256	LOC114115572	-3.478328834
LOC114116953	4.665430256	GASK1B_1	-3.476667454
TNNI3	4.663162732	GJA5	-3.472820088

GRHL1	4.659923515	LOC114114058	-3.464048089
LOC105602163	4.654540612	GRIA3	-3.458222864
LOC114111372	4.654540612	LOC114116061	-3.45816972
CADPS	4.628102946	DKK2	-3.454440523
LOC114108609	4.625919237	FAP	-3.451702455
PNLDC1	4.618546451	NAV3	-3.446149769
LOC105610401	4.615129133	MAP3K7CL	-3.446057782
RIPK4	4.615129133	LOC101118470	-3.439365006
C6H4orf19	4.615129133	COL4A5	-3.436657466
DMRTA2	4.615129133	LOC114114030	-3.43563291
DRC7	4.615013837	FFAR4	-3.433204467
ALDOB	4.609869774	ADAMTSL5	-3.427178777
LOC105607655	4.591838336	LOC105616215	-3.42566646
LOC114112736	4.591838336	WIF1	-3.42498554
DCLK3	4.590287395	LOC114112977	-3.421188696
KIAA1211L	4.583588674	HAS2	-3.41774735
TMEM266	4.570674635	ITGA7	-3.413483514
LARGE2	4.564028942	PDGFRL	-3.411660249
LOC114115623	4.548884058	SLC24A5	-3.410861124
PHYHIP	4.545628568	LOC114108813	-3.39871028
CHRNA2	4.54462673	LOC105605293	-3.39871028
CITED1	4.540950833	LOC101122940	-3.396348861
GLIS1	4.539714098	GPR21	-3.396239232
LOC114116861	4.532921339	ITGA1	-3.395956321
LOC106991313	4.517308201	TSTD1	-3.395152466
SLC35G6	4.514289756	MPPED2	-3.393740381
NXPH2	4.514289756	LOC101109425	-3.392090453
LOC101119941	4.514289756	LOC114113690	-3.385293383
LOC105602473	4.514289756	DPY19L3	-3.381717411
LOC114115326	4.512256233	NRK	-3.377819356
GRHL3	4.489991343	LOC114115593	-3.377016956
LOC105603132	4.488786867	LOC105616812	-3.377016956
OVGP1	4.47690143	LOC114114096	-3.377016956
LOC105611998	4.472566757	RGS7BP	-3.376461637
ATP6V1C2	4.470373283	LOC101112335	-3.376235192
AURKC	4.457114736	KLHL29	-3.374522611
TVP23A	4.455788945	RNASEL	-3.370741388
LOC101108898	4.445744606	CD109	-3.363601036
OTX1	4.445744606	LOC101112822	-3.360154094
LOC105603748	4.445744606	ADRA2A	-3.355168991
ANO5	4.445744606	THSD4	-3.35384826
TDRD1	4.445251205	MYO18B	-3.35028857
LOC114116087	4.443011749	CALHM5	-3.344117055
NMB	4.440386681	LOC114118067	-3.340944407
LOC105610334	4.432334763	PPARGC1A	-3.338629389

CLEC2L	4.426415177	LOC114114853	-3.338284551
CHDH	4.413044755	HAND2	-3.337246043
TLE6	4.408017565	PPFIA4	-3.335943257
LOC114116771	4.388057221	LOC114113976	-3.331449956
MRO	4.381686258	LOC114113068	-3.331130065
PLCH1	4.357143982	HDAC9	-3.323292894
NEFM	4.354809052	LOC105602647	-3.321858499
DRC1	4.352959389	LIMCH1	-3.32087622
CAMK2B	4.352959389	C3H9orf50	-3.317996921
FOXG1	4.345442271	LOC101117577	-3.315623886
LOC101122710	4.345442271	SCML4	-3.312106615
FAM189A2	4.325921648	LOC101102057	-3.310951128
KCNK12	4.322811192	FAM124B	-3.310835233
LOC114115610	4.322629883	HSF5	-3.297052643
LOC114110984	4.320919989	LOC101107224	-3.285750257
SCN5A	4.307876613	FBXO15	-3.284396666
HES6	4.306613881	LOC114118363	-3.280782738
LOC114114056	4.304228619	ART4	-3.278699145
NKAIN3	4.304228619	CDKL1	-3.275416994
OGDHL	4.302172386	LOC114116678	-3.271790656
LOC105611558	4.27700347	RAB17	-3.269895738
LOC114108815	4.254490869	PRUNE2	-3.269311843
NSUN7	4.254249534	ZSCAN31	-3.269222586
LTF	4.253794126	CCDC89	-3.269222586
CEACAM19	4.253794126	EDIL3	-3.26790751
LOC114114547	4.253794126	KCNC1	-3.263948034
SHISA9	4.252978991	GLT8D2	-3.263913172
PIPOX	4.252978991	TMEM26	-3.259646363
LOC101122577	4.252978991	DNHD1	-3.257779946
LOC114114850	4.252978991	CLDN10	-3.257624104
LOC106991918	4.243578202	ATP8B4	-3.24948641
SHISA6	4.229067479	SVEP1	-3.2490431
LOC114116870	4.222832368	LOC114116862	-3.248575417
MTHFD2L	4.218564875	MID1	-3.245770288
LOC101108627	4.212868259	ADARB1	-3.24462556
LOC101103584	4.192648031	CHMP4C	-3.237218346
LOC114118758	4.192194329	MRC1	-3.23679563
REC8	4.187181776	CCM2L	-3.235773183
SAXO1	4.166443677	LOC114117315	-3.235756851
RSPO2	4.164418744	DHRS9	-3.235756851
UPP1	4.156632518	LOC114116172	-3.235756851
LOC114113253	4.15418139	OLR1	-3.230065918
LOC114113601	4.15418139	PIANP	-3.218549736
AMBP	4.15418139	FAM217A	-3.207991915
PLCH2	4.15418139	UNC13C	-3.197956084

SLC16A8	4.15418139	COL5A2	-3.195417226
TDO2	4.1473062	KMO	-3.185419778
NOX5	4.1473062	LGR5	-3.185419778
TAS1R3	4.1473062	XDH	-3.185419778
SLC5A5	4.1473062	LOC105613828	-3.185419778
LOC114109549	4.1473062	CCR8	-3.183380509
CLIC6	4.146824501	PPP1R14C	-3.179660469
LOC105608648	4.143449079	LOC105604156	-3.179338231
TMIE	4.143449079	METTL24	-3.179238601
LOC105610844	4.141167492	CNGA3	-3.173416658
CALY	4.122492731	FAM20A	-3.141999239
LOC114117885	4.091653353	AQP7	-3.139881903
CYP17A1	4.085999904	CD80	-3.139544804
LOC114114001	4.084685231	COL15A1	-3.138398808
CYP17	4.082057137	ADAMTS1	-3.135356911
LOC114115115	4.068528931	LOC114116365	-3.1332627
SLITRK1	4.062042725	CXCL12	-3.123370546
TEKT1	4.062042725	CHML	-3.116099004
APOB	4.054380642	CD44	-3.110224318
ENPEP	4.035096505	LOC101112834	-3.104789945
NEFL	4.032346626	GFI1	-3.09655511
LOC105602037	4.032327314	TMEM255A	-3.096170175
MOGAT1	4.032327314	FRMD6	-3.092662787
LOC114117581	4.032327314	FBXO48	-3.084223776
SV2A	4.02804926	RASSF9	-3.081662448
CDH2	4.024475441	LGR6	-3.079149057
LOC106991743	4.023425805	COLEC10	-3.079149057
LOC105615197	4.014525076	DLX5	-3.079149057
CRISP2	4.0010217	TNS4	-3.079149057
STC1	3.995079844	NIPAL1	-3.079149057
NR5A2	3.984426805	B3GALT2	-3.079028963
CPNE5	3.983466922	NEK11	-3.078316916
ALOX15	3.977432973	LOC101120386	-3.077893037
DNAH12	3.971208359	LOC100144429	-3.077275764
LOC114117299	3.971208359	LOC114118855	-3.077138292
LOC114116443	3.941429397	STK32A	-3.070618105
FAM83F	3.936324888	LOC101110521	-3.067165556
EPHA8	3.933632502	CNTNAP1	-3.067106102
FMO1	3.933632502	FCMR	-3.066903656
LOC105604908	3.933632502	FLT3	-3.066903656
LOC114113830	3.933632502	GAS2	-3.066519065
NKX2-1	3.933632502	HECTD2	-3.058920609
SCRT2	3.933632502	LOC114113733	-3.056698308
KPNA7	3.930083574	IGF1	-3.055545914
GCLC	3.925054474	MEDAG	-3.054044243

ADAMDEC1	3.919273217	NDRG4	-3.042449231
EIF4E1B	3.910904095	C1QL1	-3.039903185
HMGCS2	3.907385148	RNF150	-3.037575908
CCDC13	3.892503777	SORBS1	-3.036155312
MOV10L1	3.889366517	HAPLN1	-3.030037214
HS6ST2	3.875071093	PCDH15	-3.02799555
SLC16A3	3.866402008	POSTN	-3.02761219
LOC114109056	3.860209275	GALNT16	-3.027222367
CPAMD8	3.848976715	LOC101115508	-3.026696549
LOC105616575	3.845934127	SLC24A1	-3.025419026
LOC101102096	3.840607253	SHCBP1L	-3.025419026
LOC105603379	3.840021477	MEGF10	-3.023856613
LOC101118224	3.839842466	NUDT9	-3.018529395
SSTR1	3.81250082	NHLRC3	-3.017207775
NPVF	3.79744095	DTWD2	-3.017094171
FDXR	3.79327622	ARHGAP15	-3.00930156
C2H2orf72	3.785873805	AKAP6	-3.006181537
LOC105604541	3.785873805	LOC105612625	-3.005492971
LOC114109553	3.776242557	NRIP2	-3.004420471
SPRN	3.776018114	RASSF10	-3.000776796
LOC114117603	3.774139004	C3AR1	-2.999813831
CDH12	3.772561694	LOC114113956	-2.998647177
LOC114109337	3.770587766	RERGL	-2.997198385
TMEM191C	3.770587766	LOC114110414	-2.997049215
LOC105604928	3.770587766	FXYD7	-2.997049215
GPR3	3.770587766	STAC2	-2.994406054
LOC114110815	3.769615767	LOC105611786	-2.988008976
ADGRB1	3.766887325	LOC105612432	-2.986067059
LOC105609280	3.766157619	LOC114109038	-2.986067059
CHAC1	3.765681169	ADAM12	-2.983233277
LOC443320	3.764571196	CCDC102B	-2.978450557
FGFR4	3.760377045	CAMK2A	-2.978450557
INHBB	3.757249965	CRIP1	-2.972780305
ALPL	3.756427596	ARHGAP29	-2.97077571
LOC105603166	3.755997257	PADI4	-2.964423372
LOC114111330	3.753345885	LOC114117257	-2.964423372
LOC114109701	3.748504171	ADAMTS15	-2.961482691
GPT	3.740472579	LOC105611988	-2.954916572
SCN10A	3.722842393	ABCD2	-2.952434395
IGSF11	3.711636248	LOC114116372	-2.946945693
LOC114118386	3.710140766	LOC101111669	-2.946882441
LOC114110143	3.708705611	PNMA2	-2.946882441
TNFRSF13C	3.706818418	SOWAHC	-2.94319696
LOC106990570	3.705659298	LOC100134870	-2.942757845
GDF9	3.703467444	PTGIR	-2.93747207

CCDC155	3.702550774	SSC5D	-2.935139593
TNNT2	3.697190192	CEACAM16	-2.934333245
LOC105605961	3.696995846	LOC114109535	-2.930504203
LOC105609125	3.696995846	ANKRD6	-2.927247022
LOC114116384	3.692730374	BEAN1	-2.925871008
LOC106990117	3.691160002	SLC22A3	-2.919595762
PLEKHH1	3.684699051	HOXA10	-2.915325064
LOC114115412	3.682267594	RNF125	-2.913029691
LOC114111236	3.677042811	FSTL1	-2.907832035
GPR162	3.677042811	LDLRAD4	-2.905861881
LOC114113201	3.67317509	ZNF831	-2.903447434
LOC114109099	3.67317509	NPAS4	-2.903447434
BTLA	3.670729288	LOC105608584	-2.896742932
TEX15	3.670729288	S100B	-2.892968834
EMID1	3.665547259	CREB5	-2.887424626
LOC114113975	3.653397621	HSPB6	-2.884793581
RIMKLA	3.650472734	NCALD	-2.881879062
LOC114112250	3.621232686	ABCA10	-2.881553698
UNC79	3.619447266	PAQR7	-2.88099571
LOC101120447	3.619447266	FBXL22	-2.880587435
LOC114113260	3.619447266	LOC114117274	-2.873858424
LOC105606717	3.619447266	LOC114111267	-2.871963744
LOC114110131	3.609271479	MALL	-2.861698079
NOS2	3.609039855	LOC106991530	-2.860935777
TMEM179	3.608592793	DOCK10	-2.850111359
LOC106991070	3.603138878	LOC105605028	-2.849623402
LRRN4CL	3.589513424	TP53INP2	-2.848513808
LOC105611015	3.588521493	CCR4	-2.839780161
NOTUM	3.585883255	ABRA	-2.839780161
CDCA3	3.579906236	PERM1	-2.839780161
DHCR24	3.576033197	NAALADL1	-2.838474252
CCL25	3.567508963	ZNF502	-2.838383418
LOC114117782	3.562218295	SLC9A7	-2.838383418
LOC105603076	3.542827065	DMRT2	-2.838220715
SLC44A3	3.542827065	NTN4	-2.833096488
C2CD6	3.537492273	TMEM59L	-2.831054604
ALDH8A1	3.537492273	PCNX2	-2.828845036
MYBL2	3.523041551	LOC101116441	-2.826932555
LOC114112722	3.52288744	ECM2	-2.825895087
LOC105604743	3.52288744	RASGRP1	-2.825426317
LOC105612436	3.520534299	P2RY13	-2.824384026
FABP3	3.515051449	FCHO2	-2.821724546
RTL1	3.50134476	CHODL	-2.819601932
TCTEX1D4	3.50134476	FILIP1	-2.817620671
LOC105606685	3.49781498	LOC105616380	-2.816957617

F10	3.496366481	ANO3	-2.815623241
PROM1	3.475566481	NUDT13	-2.815451882
MTFP1	3.474651031	PDE5A	-2.813168326
MCRIP2	3.471015347	PTPRB	-2.812081081
C15H11orf87	3.470496028	DSC2	-2.810609633
BAIAP3	3.458944385	MSC	-2.808107684
LOC106991954	3.457483279	LEKR1	-2.805104007
DNAI2	3.455697944	LOC114111493	-2.805104007
CFAP157	3.450599781	C1S	-2.803676525
LOC105601991	3.450599781	SESN3	-2.801186175
AK9	3.450599781	LOC105607925	-2.797843424
LOC105612071	3.450599781	COL3A1	-2.797079261
LOC101114089	3.450599781	P2RX1	-2.795894449
LOC114113194	3.450599781	NEXN	-2.792420209
LOC105615767	3.450599781	GASK1B_2	-2.790252166
TMEM229A	3.450599781	CLEC12A	-2.786736706
PHYHIPL	3.450599781	KCNT2	-2.780451303
SLC29A4	3.450074223	SUSD2	-2.779705901
CHGA	3.447103708	ANKS6	-2.777277093
TACR3	3.443391839	LOC114109398	-2.773172961
LOC101122142	3.442419352	BST1	-2.772523531
LOC101110855	3.441617883	C1QTNF2	-2.772083982
PPARG	3.430225094	VSIG10L	-2.769542783
CDH1	3.425849676	ACAP2	-2.769479186
LOC101104074	3.425849676	RASL10A	-2.76927325
LOC114112870	3.425849676	KL	-2.768401129
LOC101118452	3.423073532	C3H12orf75	-2.768233327
LOC114116336	3.423073532	LOC101113086	-2.768208374
LOC114115022	3.423073532	CTNNA3	-2.768111728
C11H17orf97	3.420525688	LYZ	-2.762117065
ATF7IP2	3.411515173	LOC101108006	-2.761731214
CCDC116	3.411325019	GPR37	-2.75663676
TRIM9	3.410385316	PLEKHD1	-2.753793095
JAKMIP1	3.410385316	GPR17	-2.751184607
PCSK4	3.408559543	LOC114118743	-2.751184607
LOC114113912	3.408559543	LOC101110777	-2.751184607
CFAP52	3.408559543	LOC101112891	-2.751062636
ANKLE1	3.400925746	SEMA3G	-2.74513547
DNASE1L3	3.394893082	GPR20	-2.744916946
PC	3.386406561	CD7	-2.744182206
DNA2	3.377884098	EPB41L3	-2.741671079
EPS8L2	3.368222761	BCHE	-2.737595267
LOC114116963	3.360594786	TMEM220	-2.735182819
LOC114115006	3.358136501	IL2RA	-2.734994532
LOC114111295	3.358136501	LOC114118016	-2.731896457

LOC114118888	3.355103877	BRINP3	-2.731669172
DIRAS1	3.355103877	KCNQ4	-2.729578102
LOC114112835	3.355103877	ADIRF	-2.728176234
PLSCR5	3.355103877	SLC41A2	-2.725763412
LOC114112825	3.355103877	LOC101112688	-2.724337327
GLIPR1L2	3.355103877	CNN1	-2.723740115
LOC101117799	3.355103877	TBX21	-2.722885285
LOC114117857	3.355103877	SYNM	-2.722299001
LOC114118691	3.355103877	LOC105602254	-2.721579294
GBGT1	3.344353972	WFDC1	-2.721236909
MSLN	3.335863275	MFAP4	-2.720568119
PPIF	3.335154357	SULF1	-2.719857764
ORC1	3.32978634	LOC105613002	-2.719682596
SLAIN1	3.32950141	CHST3	-2.712027834
FAM155A	3.326779652	LOC114110341	-2.71058206
CREB3L3	3.322445544	LOC105605766	-2.710021427
AZIN2	3.30939428	ZNF366	-2.704289234
IQANK1	3.30939428	LOC105609508	-2.703341122
TMEM262	3.30939428	LOC101118212	-2.703341122
TRHDE	3.309242486	LOC101118024	-2.703341122
NPTX2	3.306974375	LOC114115581	-2.703341122
ESPN	3.300352807	LOC114114542	-2.703341122
EMILIN3	3.296002721	LOC105610178	-2.703341122
SLC5A8	3.292472426	DLGAP1	-2.703341122
SSTR3	3.284916649	ANKRD31	-2.703341122
CHRNB4	3.282267045	LOC105610161	-2.698619324
EPHX2	3.279568793	LOC114111026	-2.698619324
HCN2	3.26959822	RXFP4	-2.696986017
TTR	3.265534825	FAM83E	-2.696986017
SAP25	3.265123998	LOC114110991	-2.696986017
LOC114110618	3.2593389	IL2RB	-2.695537356
RBPJL	3.2593389	LOC101119706	-2.695184813
LOC114113256	3.2593389	MYOCD	-2.693830489
SLC45A1	3.2593389	ADAMTSL3	-2.688730497
TMIGD2	3.2593389	EOGT	-2.685062448
RPRML	3.2593389	CILP	-2.683911112
DUPD1	3.2593389	MLKL	-2.678064436
GAPDHS	3.257132603	MST1R	-2.677338973
HPN	3.25635733	COL21A1	-2.675873097
UNCX	3.255560769	IL15	-2.675229684
CALB2	3.25395975	LOC114114762	-2.674040304
MRPS25	3.242652186	ABHD16B	-2.671966224
MAP7D2	3.233687021	RUNX1T1	-2.671053088
LOC105609715	3.228537571	ADPRH	-2.669897005
LOC105606524	3.222452939	SAMD4A	-2.666208842

LOC114116353	3.222452939	MICAL2	-2.665482326
LOC105611989	3.222452939	ADAMTS12	-2.664627041
F5	3.222185153	LOC101110922	-2.661303199
MYO15A	3.219931279	LOC105605321	-2.661303199
CTNNA2	3.21208182	LOC114112171	-2.661303199
SGPP2	3.20893791	LOC101115931	-2.661303199
GSTA1-1	3.204814952	ARSB	-2.657395067
LOC101117691	3.204306935	LRRC10B	-2.656410108
CHRNA3	3.202906355	CAMK1G	-2.655677445
PLS1	3.200581953	LAIR1	-2.654352192
MESP1	3.197015016	LOC114116423	-2.653734168
CHST11	3.194107268	SRPX	-2.653475155
LOC106991666	3.194015852	MAP2	-2.651757737
BHMT	3.190798533	KRT5	-2.650495852
FAM162B	3.185043152	LOC101119773	-2.647092371
LRRC38	3.182561645	ATL1	-2.646504858
TNNC2	3.182561645	MEGF6	-2.644961537
LOC105612366	3.182561645	CXCR6	-2.640827033
DBF4B	3.177597409	LOC114113970	-2.640287493
EEF1B2	3.166509301	LOC114117877	-2.638033182
HMGB3	3.164137888	LOC101115787	-2.634875357
SLC25A10	3.159350324	LOC114114463	-2.633228979
LOC105616340	3.15496302	LTB4R2	-2.632971919
LOC101123010	3.154103181	LOC101123290	-2.630885731
LOC114116205	3.15327485	LOC114116965	-2.629956462
LOC114115603	3.15327485	ST18	-2.629956462
LOC114115731	3.15327485	LOC105610651	-2.629956462
LOC114118689	3.15327485	LOC105616288	-2.629956462
LOC114111030	3.144198239	PNOC	-2.629867943
MYBPC1	3.144198239	LDB2	-2.627418529
ZNF674	3.143510571	INPP4B	-2.626912684
ACSBG1	3.121252425	LOC114109118	-2.62584266
LRRC2	3.120623972	ANTXR1	-2.625816302
UBE2QL1	3.119205654	PRR22	-2.625360438
GPRIN2	3.116750847	LOC105611362	-2.625360438
CCNI2	3.113310059	EGR2	-2.624221357
DUSP9	3.113310059	LOC101111764	-2.622996185
LOC105609364	3.113310059	LOC105604270	-2.622996185
CARD14	3.113310059	SCN2B	-2.622907289
C16H5orf49	3.110833908	TPO	-2.618953143
LOC101117785	3.108258608	LOC105604676	-2.616831729
GJB4	3.104786761	PPP2R2B	-2.616311049
PHOX2A	3.098340938	BGN	-2.615962742
LOC101122645	3.096092759	COL5A1	-2.614655736
NCKAP5	3.094250977	CPZ	-2.613939486

LOC114115267	3.087927469	LOC105603904	-2.612802001
LOC114118467	3.087927469	RAB7B	-2.612733795
FOXO6	3.084252029	SHE	-2.612448879
LOC114108805	3.083955673	LOC101121244	-2.608713713
NLRP2	3.083005469	CX3CR1	-2.606063009
LOC114109441	3.083005469	ST6GAL1	-2.605107612
LOC105614699	3.083005469	MXRA5	-2.603629348
PCSK1	3.077573637	WDFY4	-2.602517317
MIF	3.076715522	CGREF1	-2.602445232
FKBP6	3.074974511	VAV3	-2.601522445
LOC105606568	3.069634471	NLGN4X	-2.600580909
RMI2	3.066889223	ND3	-2.594289522
LOC101108413	3.064268331	CLEC5A	-2.594193069
LOC114109422	3.064185545	GRID2	-2.594193069
INPP5J	3.054676147	TNFSF18	-2.59333153
NUDT11	3.052050633	DUXB	-2.591451761
LOC105609012	3.052050633	ADGRD1	-2.588317045
LOC105609925	3.049282546	MAF	-2.587219949
LOC114110481	3.045280036	ITGA2	-2.585366127
FAM222A	3.042181657	IRAK3	-2.58506654
MCEMP1	3.038790012	LOC101105553	-2.584490292
LOC101112469	3.038790012	PNMT	-2.583644218
KRT36	3.038790012	LOC101108745	-2.583644218
LOC101119591	3.038790012	IPCEF1	-2.583644218
SCG2	3.038790012	LOC105603273	-2.583644218
SLC17A7	3.038790012	TNFRSF13B	-2.583644218
LOC114109578	3.038790012	LOC114114519	-2.583644218
LOC114111034	3.038790012	LOC114116356	-2.583254577
LOC114109344	3.038790012	LOC114116412	-2.583247754
FAM57B	3.038790012	ROR1	-2.582682358
C8B	3.038790012	SETBP1	-2.580212236
LOC101113487	3.038160653	LOC101101976	-2.579498576
FAM83G	3.030285481	KANK1	-2.577341428
FZD5	3.029876262	NCS1	-2.575083251
LOC106991659	3.024945666	LOC101121256	-2.57420379
FAM169A	3.022982896	PTPN22	-2.57420379
LOC101122753	3.022578967	LOC105604226	-2.573203361
REC114	3.022578967	STUM	-2.567787424
LOC114114985	3.022578967	AMZ1	-2.563657741
LOC114114490	3.020811562	LOC105604471	-2.563246704
LOC105602963	3.02041703	LOC114118385	-2.563229821
LOC105611804	3.02041703	CALN1	-2.55765773
HK2	3.019901598	GCNT4	-2.555420071
KRT19	3.017682847	ALDH1L2	-2.554431132
LOC105612323	3.006867724	MOCOS	-2.553328166

NLRP8	3.000831272	FER1L5	-2.552638
LOC101111541	2.998999991	LOC101105731	-2.552638
PTPRN2	2.99563296	SLC39A2	-2.552638
PDZD4	2.988074227	NUP210L	-2.552638
PLAC1	2.988074227	LOC114116118	-2.552638
LOC114112700	2.987889119	LOC114115675	-2.552638
STYXL1	2.987765662	LAMA3	-2.550105596
LOC114113171	2.979648971	ITGB4	-2.549951214
LOC114108727	2.976282037	ADCY7	-2.549291353
LOC105611014	2.974117969	ITGB3	-2.549211525
KLC3	2.971976456	WHRN	-2.544036218
LRRC46	2.966740023	ADA	-2.543621137
PKMYT1	2.966282149	OLFM1	-2.543574648
LOC105608946	2.966020983	OLFML2B	-2.543403926
LOC114110609	2.962985302	ITGB1BP2	-2.543188688
TRPV6	2.954989692	LOC101105017	-2.543188688
LRRC73	2.954469092	RNF144B	-2.539688872
MYO5B	2.95343064	NIPAL4	-2.538611732
CRYBB3	2.952242585	XYLT1	-2.537814231
NGEF	2.950119438	RCAN3	-2.535724735
PRR15	2.948513424	ELF4	-2.535009082
LOC114113257	2.945127052	EYA4	-2.531275864
BEGAIN	2.93424355	LOC105613245	-2.524615142
FSHR	2.930942909	PTPRC	-2.524462772
OIP5	2.930839955	LOC101114456	-2.523793498
SLC35G1	2.925176489	EVI2B	-2.522514598
ANKRD37	2.92256188	MILR1	-2.522006993
ANKS1B	2.914431061	JPH2	-2.520530273
LOC101104222	2.914431061	LOC101112726	-2.520403038
FBXL16	2.914431061	MGP	-2.520260229
LOC114108680	2.914431061	SLC7A2	-2.519694149
LOC105602877	2.914431061	LOC114112840	-2.519089717
LIG1	2.90959644	LOC114110650	-2.519089717
RASSF7	2.908421887	FBLN5	-2.510790321
LOC114109660	2.908421887	MYCBP2	-2.509008514
MAL2	2.906164603	RHOJ	-2.507004646
LOC114114854	2.902464954	EDNRB	-2.505414422
UBE2S	2.90231934	ITPRID2	-2.502307876
LOC106990546	2.901706351	LYPD6	-2.502180281
E2F2	2.901680584	LOC101122306	-2.501800572
PRR29	2.900355448	KLF13	-2.501754696
INA	2.896207408	RAPGEF5	-2.501622187
PAH	2.896207408	TJP3	-2.501565908
LOC114109125	2.895527901	TESPA1	-2.501565908
LOC114117509	2.892360128	CUBN	-2.501565908

ENO1	2.890040968	SLC22A4	-2.501565908
LOC114116606	2.889755418	LOC105604792	-2.501099821
AP3B2	2.889755418	PTGS2	-2.500486691
LOC114111311	2.887412932	LOC114111015	-2.499141361
MCM5	2.870207112	RNF24	-2.496791608
LOC101110185	2.869377979	LOC101114082	-2.492259974
LOC101115605	2.868272612	OXCT1	-2.492228734
LOC114110420	2.86550452	NPTXR	-2.491551858
CRHR2	2.864911682	LOC105601852	-2.491127205
GPR35	2.861698966	LOC105602828	-2.490797993
NR1H4	2.859295557	CFAP54	-2.490367046
CSRNP3	2.857566661	LOC101106330	-2.489138952
GTF2A1L	2.850924684	LOC101103178	-2.488759004
CHRNB2	2.850924684	NEK1	-2.48748905
PAPSS2	2.846447926	DUSP4	-2.483981531
LOC105608303	2.842617016	LOC101106166	-2.483510311
LOC105606413	2.842617016	IL33	-2.481225567
CAMK2N2	2.842617016	OSMR	-2.480008379
TPH2	2.842617016	LOC101122689	-2.479292436
KRTCAP3	2.840972685	BMPER	-2.47715212
FZD9	2.834496491	MMRN2	-2.476240536
LOC105603181	2.834450081	HGF	-2.475856289
PMM1	2.833545562	TMEM116	-2.474275017
LOC114118047	2.831349102	CADM3	-2.471916483
LOC114118700	2.826187921	LOC101102967	-2.470939982
LOC114109532	2.826187921	FCGR1A	-2.469417736
LOC101110277	2.826187921	LOC105608279	-2.466612767
LOC114113588	2.826187921	ZNF333	-2.465448776
SLC22A16	2.826187921	CLEC6A	-2.464896865
NMRK2	2.826187921	NKD1	-2.463609562
DNAH2	2.826187921	SYP	-2.46219066
LOC114112919	2.824745075	APOBEC2	-2.460153114
CEP72	2.822587574	STON2	-2.458956479
ADAM11	2.822098421	TMEM252	-2.458706516
EML5	2.816938714	TNFSF10	-2.457795174
CDCA5	2.816673315	PRND	-2.457289108
LOC105610279	2.814498633	LOC106991067	-2.454459981
MSI1	2.811846503	VSTM5	-2.454459981
DNAH9	2.80528575	LOC114113240	-2.453230497
LOC105612338	2.79717572	EPHA7	-2.452574814
GGT6	2.79717572	CACNB2	-2.452164295
LOC101112093	2.793886247	DOCK4	-2.451278145
LOC114117596	2.793886247	KCTD12	-2.450870169
MATN4	2.793886247	EHD3	-2.450423696
LOC105609938	2.793886247	PDE4C	-2.450261238

FOXP3	2.793886247	SBSN	-2.450124649
CHRDL2	2.793798348	CPEB4	-2.449143447
PKP2	2.79211986	SETD6	-2.447479654
CDCA7	2.791134195	LOC101121563	-2.447143677
POMC	2.787593219	LOC101115808	-2.444633961
ERP27	2.779120427	MYRIP	-2.443881205
ERICH3	2.7783326	PLN	-2.440591522
LOC105613571	2.7783326	PECAM1	-2.439412388
RADX	2.7783326	PDE1A	-2.435920834
LOC114114024	2.7783326	LAT2	-2.435580053
LYPD6B	2.7783326	CPXM2	-2.43454762
RUFY4	2.7783326	SNX10	-2.433891199
FAM227A	2.7783326	PQLC3	-2.433098922
FAM221A	2.7783326	ALOX5	-2.430439698
LOC105606289	2.7783326	LOC101121825	-2.429992367
MS4A10	2.7783326	FAM180A	-2.429017368
PCDHAC1	2.7783326	XCL1	-2.428644305
LRRC4	2.7783326	MTMR11	-2.427139409
DIRAS2	2.7783326	RASGRF2	-2.42683188
LAMA1	2.777424433	LRRK2	-2.426579449
PRRT1B	2.777381242	LOC101119638	-2.426553974
KRT80	2.777381242	PRSS35	-2.426371007
LOC105610886	2.777128999	PRRG4	-2.424812646
COL6A6	2.773498241	MSRB3	-2.421840593
LOC105605590	2.76996389	ACER2	-2.419242787
SERPINA1	2.768940032	PRKG1	-2.41728655
LCN6	2.766871131	CSRP2	-2.416725044
GNB1L	2.762970265	IL1R1	-2.415544532
NTNG2	2.761812693	LOC101123625	-2.415003314
GNMT	2.759945434	BATF	-2.415003314
C12H1orf100	2.759945434	SPAG17	-2.414534776
PHF24	2.755459781	LOC105603539	-2.414534776
PCBP3	2.753890484	LOC114115664	-2.414534776
DNAJC27	2.753076121	LOC105605117	-2.414534776
STK32B	2.75300482	MEOX2	-2.412431676
LOC101102714	2.752596	THBS1	-2.410935979
TFAP2E	2.752596	LOC105603604	-2.408347705
LOC105604110	2.752596	NPY1R	-2.407439918
LOC114114551	2.749056693	IL1B	-2.407330675
TOX2	2.747768799	LOC105611518	-2.407330675
PHGDH	2.744448347	ZNF577	-2.404878179
RGS11	2.743939156	CFAP206	-2.404364033
ESR2	2.743451753	LOC101107098	-2.401858659
SH2D5	2.743451753	TSKS	-2.399635283
BICDL1	2.740016454	LOC101108705	-2.398694558

FAM166B	2.735916294	COL11A1	-2.39807989
KLK1	2.734483375	GPR83	-2.396737732
KIF22	2.729730046	LRAT	-2.396663007
TTN	2.726925761	FOXN2	-2.39467056
KRT18	2.723940797	TFAP2A	-2.394543255
LOC114110058	2.722820258	GATA3	-2.392609277
ALDOC	2.717062367	LOC101112639	-2.389288226
LOC106990346	2.717061515	CLIC2	-2.388697755
LRP8	2.71291769	LOC105603436	-2.388448397
LOC114114548	2.711204073	FBN2	-2.38577525
LOC114112236	2.709943331	RGS18	-2.384307328
LOC114114480	2.707581088	RINL	-2.384302996
CNTD2	2.707581088	CD3G	-2.384299309
CATIP	2.699354115	LOC114114896	-2.383478083
FBXO5	2.696054767	PDE3B	-2.382318091
EFHC1	2.69254855	PTPRJ	-2.382073399
LOC114112899	2.691465898	CCDC88A	-2.381357002
ETNK2	2.687886888	PKD1L3	-2.378427061
MVD	2.686961941	LOC101120001	-2.37597512
CXXC4	2.686899627	SHISAL1	-2.372127958
SH2D4A	2.686242466	ALX1	-2.371050936
LOC101117129	2.684215563	LOC101108102	-2.37046669
OTOA	2.680668491	DAPP1	-2.369812646
LOC101118307	2.677136502	LOC114118066	-2.368967776
BNIP3	2.676301423	LOC101107486	-2.368967776
SPACA4	2.67504742	FAM241A	-2.368815691
LOC114110490	2.67504742	LPXN	-2.368574691
ADGRE2	2.67504742	LOC101103973	-2.360614429
BMP5	2.67504742	PDE4D	-2.358520144
LOC101110202	2.67504742	LOC105612674	-2.358206456
OSGIN1	2.67411615	PTHLH	-2.357845911
C3H2orf81	2.671939888	PAK3	-2.357713681
KCND3	2.671939888	PTCHD1	-2.357377118
ACBD7	2.671939888	LOC101119832	-2.357377118
LOC101106395	2.671882848	SHC4	-2.35632066
LOC105605445	2.6709257	CNTNAP4	-2.35632066
CLDN15	2.67042257	THBS4	-2.354342915
TDRD10	2.670092671	ITGA5	-2.351840752
HDC	2.658835685	AKAP12	-2.351704085
LHB	2.653562008	ROCK1	-2.351476539
UPK1B	2.651129881	KLHL28	-2.350949092
LOC106991576	2.650808206	LOC105604063	-2.349455256
RECQL4	2.650424438	LOC101117971	-2.349285679
LOC101108787	2.648883736	SULF2	-2.349106854
CDT1	2.643857277	GUCY1A1	-2.348647921

BCL2L10	2.636963828	CPE	-2.3473202
SLC8A3	2.636963828	CC2D2B	-2.345246533
LOC101117021	2.636963828	PDZRN4	-2.343161788
VWA3A	2.636963828	SYNPO2	-2.342618499
LOC105609379	2.634384329	KCNMA1	-2.342155136
PTGES	2.632840446	HOXB3	-2.342086716
LOC114116105	2.62804495	COL12A1	-2.341896852
LOC114115655	2.62804495	LOC101118514	-2.3398702
LOC114115236	2.62804495	THSD7A	-2.338035576
DNAH7	2.62570525	MRVI1	-2.337408857
PALM3	2.622042213	SMAD7	-2.333517374
ZNF875	2.617146321	LOC114110119	-2.33202989
SLC15A1	2.617146321	GOLIM4	-2.33172701
LOC105611997	2.617146321	NCF1	-2.331196562
RPP25	2.613770869	KCNT1	-2.330160434
LOC105611533	2.611766176	ARSJ	-2.329455107
LOC114113943	2.610419878	LACC1	-2.325903549
COCH	2.607960728	TRMT9B	-2.325577197
SMG9	2.607948123	LOC114115264	-2.325241994
SLITRK4	2.602465683	STK38L	-2.324983386
CARMIL2	2.601392894	ZNF554	-2.321914486
STRA6	2.600494321	LOC105603417	-2.321914486
TMEM233	2.593092427	LOC101110403	-2.321914486
TFR2	2.592050714	CCDC3	-2.321302404
CLDN3	2.590857696	LST1	-2.320612734
LOC105607520	2.586521857	SYNC	-2.320485746
GLP2R	2.583349409	LPAR3	-2.320322609
PCDH20	2.582173153	POLK	-2.31773913
KMT5C	2.580886721	PLCB2	-2.316301854
ELOVL4	2.580745718	APBB2	-2.312790513
LOC105602434	2.58061952	LOC105603695	-2.312597792
TFF2	2.58061952	TBX18	-2.310361781
LOC106991901	2.58061952	CXCR3	-2.30989183
LOC114114884	2.58061952	MCAM	-2.309634368
LOC114118361	2.58061952	LOC101119426	-2.307423152
RPGR	2.579123445	PLXNA4	-2.304899567
MCM2	2.576345778	NTRK2	-2.304723849
ZYG11A	2.574216325	RELN	-2.304495274
TKTL1	2.574206381	RSPO1	-2.302971826
MAT1A	2.570319293	JAZF1	-2.300652594
NPAS3	2.570319293	FAM13B	-2.300383836
NEURL1	2.568729896	PIP5K1B	-2.300251575
TLL2	2.566220337	GNG12	-2.297874824
FCGBP	2.566127094	MUSTN1	-2.297841941
LLGL2	2.565495022	ANGPT1	-2.297645557

ST8SIA5	2.56439445	ACTN1	-2.296988177
KCNH1	2.56439445	TRIM36	-2.293596507
YBX2	2.560748556	FOXC1	-2.29357874
CDK7	2.555504047	EDNRA	-2.288656342
XKRX	2.544500161	CAMK2G	-2.28546678
ADM	2.536881548	LOC554335	-2.285176593
LOC114117260	2.534790345	KIAA1549L	-2.284459316
SLC25A53	2.531969342	SPARC	-2.283653867
RGS17	2.529969588	KCNQ3	-2.283511839
LOC114109026	2.529969588	LOC114111673	-2.281749042
RTL9	2.529969588	DMD	-2.281467333
LOC114115365	2.527709201	LOC114117355	-2.281437393
ASF1B	2.525769257	SMOC2	-2.281024136
CHRNA7	2.524808308	TMEM156	-2.27678722
LOC114118859	2.521984942	GALNT17	-2.275308822
PITX3	2.521984942	CALCRL	-2.274627509
FAM167A	2.521984942	GPC6	-2.273461365
LOC114116820	2.521984942	NTF3	-2.272363229
LOC105610222	2.521984942	PLPP1	-2.271752265
RPL23A	2.52141618	LOC105612707	-2.27092978
ZCWPW1	2.51578832	MCTP1	-2.270891026
S100A3	2.506199936	MSX2	-2.268879957
LOC106991594	2.506199936	RIMBP2	-2.266684588
GPC2	2.506199936	FAM84A	-2.266180996
LOC105602343	2.506199936	OGN	-2.26576192
LOC106991069	2.506199936	KCNE4	-2.26431077
CA1_1	2.506199936	PTGIS	-2.263432493
LOC114112904	2.506199936	RPS6KA6	-2.261778109
LOC114109042	2.506199936	SEMA3D	-2.258669518
INHBA	2.504242927	OPRL1	-2.258498369
RIC3	2.500473606	LOC114109035	-2.258237023
REEP4	2.500457003	DSTN	-2.257418449
LOC114112176	2.496338993	GPR62	-2.257207205
THOP1	2.496334867	GLS	-2.255788657
LOC105606461	2.494914824	FXYD1	-2.252749686
PSD	2.493074416	LOC114115224	-2.251348785
NPM3	2.490519615	TEK	-2.249589751
LOC101106791	2.488502266	TSPAN2	-2.248738345
RHCG	2.487963248	HOXA3	-2.248355366
LOC114110160	2.484322772	LOC114110611	-2.247609909
AURKB	2.484070526	CCSER2	-2.24725047
CDC6	2.482604363	IL20RA	-2.245431604
LOC106991804	2.480750955	ADHFE1	-2.244347976
ARHGAP22	2.478705522	PTPRZ1	-2.242905105
VEGFA	2.478292108	TGFB2	-2.240661002

LOC114115385	2.475746598	SORCS2	-2.239762777
LOC105605507	2.472794874	NKAIN2	-2.237872669
C5H19orf57	2.47060217	GABRG1	-2.237872669
KIF21B	2.461445949	LGALS12	-2.237872669
LOC101108158	2.460258876	LOC114116676	-2.237823909
SLC6A20	2.457461847	ZC3HAV1L	-2.234766678
KIF12	2.451839151	LOC101108001	-2.232867439
LOC114117543	2.4504372	PRELP	-2.231251665
LOC114116706	2.449117083	SCN3B	-2.22959181
SMC1B	2.449117083	LOC105614722	-2.227768978
BSN	2.446335306	LOC114116957	-2.225155857
BRINP2	2.445518603	PLEKHA6	-2.224813394
LOC114118712	2.444212566	LOC101105840	-2.222937597
CYP11A1	2.443084613	LOC105615501	-2.222937597
CHST13	2.442049773	LOC106991117	-2.222937597
GRM8	2.441272278	HOXA2	-2.222937597
COL9A1	2.439742549	LOC114110426	-2.222937597
TICRR	2.433291031	LOC114117773	-2.222589464
BEX5	2.427823705	LOC101116756	-2.22252609
LOC114116631	2.426646504	ICOS	-2.219474653
LOC105605730	2.425958965	NHSL1	-2.218156796
HMOX1	2.424792987	LOC105610529	-2.21573675
TEDC2	2.419419018	XCR1	-2.214650721
ST3GAL4	2.418532339	CASS4	-2.214650721
EYA2	2.41625134	RAP1GAP2	-2.213492427
LOC114110476	2.414072823	CYTIP	-2.213492427
LOC106991640	2.413736655	LOC105609509	-2.213113621
C7H15orf61	2.409182137	LOC105604044	-2.213113621
ADAM8	2.403855758	TGFBI	-2.213013001
LOC114109326	2.399894142	FAR2	-2.212890227
CITED4	2.399345462	EOMES	-2.211953864
CCDC197	2.397434728	SASH3	-2.211333121
TBPL2	2.397042776	PLCE1	-2.210654303
LOC105604036	2.397042776	EEF1A1	-2.209114537
KCNJ12	2.397042776	ADAMTS13	-2.209092625
ALX3	2.397042776	LOC114118409	-2.208208081
IL11	2.397042776	LOXL2	-2.20413657
WWC1	2.395738607	PKIB	-2.202028027
ARG2	2.393792901	LOC114110128	-2.200320469
TMEM198	2.390421407	TAGAP	-2.199794048
OBSL1	2.388872998	AVIL	-2.199794048
TIMELESS	2.38829566	KDM5A	-2.197857349
UHRF1	2.385263526	PCP4	-2.197662502
FOXP2	2.38237379	SLA	-2.1968736
LOC114118000	2.3822452	LOC114112946	-2.193008644

BAIAP2L1	2.381163854	LOC101112084	-2.192954117
EXO1	2.380967344	LOC101106534	-2.192458352
MT3	2.379696418	DST	-2.192317576
LOC101116286	2.375253225	LCP2	-2.186033385
LOC114110487	2.37249507	LRRC31	-2.185560881
LOC114110812	2.366296234	LOC106990881	-2.185560881
TSPAN33	2.362057059	LOC114118466	-2.185560881
TG	2.358362762	LOC105606280	-2.184329291
LOC106990141	2.356354894	NDST3	-2.184329291
PDE6B	2.352133526	LOC101116336	-2.183395556
LOC105609492	2.351081014	LOC101113965	-2.183332998
C11H17orf53	2.350308437	ITGA9	-2.183071807
DSP	2.346974601	LOC105609969	-2.179641992
CNRIP1	2.345992596	DGKG	-2.178319838
LOC101111401	2.34583533	WDR47	-2.177986584
LOC101123533	2.338019107	FCHSD2	-2.177932354
C2H2orf88	2.338019107	XKR4	-2.177845633
PUSL1	2.332549164	COL1A2	-2.176229975
LOC101102327	2.331824132	KDM7A	-2.174995018
CPM	2.331699481	UTRN	-2.174399139
LOC106991772	2.331139882	LOC114114616	-2.172184117
CCDC38	2.328037309	RBPMS2	-2.171606549
SRGN	2.322293098	LOC101102231	-2.17078052
TMEM92	2.314939055	LOC114117866	-2.17078052
C11H17orf50	2.314939055	SYPL2	-2.17062577
LOC114113250	2.314939055	LOC114117972	-2.168726721
LOC101106282	2.314939055	AK5	-2.165759032
CHRNA5	2.314939055	HIVEP2	-2.162791123
INHBE	2.314939055	LOC114117779	-2.162757964
DPYSL5	2.314939055	SORBS2	-2.161171513
TREM1	2.314939055	ADRA1D	-2.160111741
SCAP	2.311701708	LOC114117808	-2.15951715
SURF2	2.310648532	HES4	-2.159046431
ASPHD2	2.307448655	LOC105607326	-2.158643589
LOC114116062	2.301011982	CLEC4A	-2.156193448
SLC27A6	2.301011982	PAQR6	-2.156191761
PRKAA2	2.299322651	BCAT1	-2.153356882
TMEM132A	2.2992865	CACNA1C	-2.152512499
CBARP	2.297494583	LOC105611136	-2.152462473
CCDC85C	2.295290805	PLD4	-2.15225831
NKAIN1	2.294945596	NRGN	-2.150065329
HHIP	2.294640174	ITGAV	-2.148553565
CDK5R2	2.289039427	CCR5	-2.147514588
LOC114116607	2.28901852	RHBDL3	-2.146001207
SBK1	2.288920543	LOC106991852	-2.14567328

KRT3	2.287240334	AEBP1	-2.145184524
PLEKHA7	2.286013223	LOC101102310	-2.144889415
LOC101111058	2.28575743	LOC114108849	-2.144400502
E2F8	2.284861536	PLCL1	-2.143466903
LOC105611500	2.2825462	ESM1	-2.143185777
LOC101105609	2.281483034	ITGA3	-2.141853487
PLPPR2	2.2803055	FRRS1	-2.141157752
BCL11A	2.279813893	DUSP27	-2.138510163
AKR7A2	2.276143159	SGIP1	-2.137586632
CNIH2	2.27563141	SELP	-2.137446982
NXNL2	2.27563141	FHL1	-2.137290065
SELENOH	2.274683819	NLRC3	-2.136640319
UPK3B	2.272892884	TCP11L1	-2.135039484
LOC114118731	2.272892884	LOC101120574	-2.134765157
ADAMTS7	2.271336805	EMP1	-2.133990441
TK1	2.268831872	GANC	-2.133503566
LOC114116623	2.265712543	LOC101109388	-2.13293352
PVALB	2.265138989	PLCG2	-2.132249097
LIN7B	2.262500752	LOC101112038	-2.132062397
LOC105607692	2.261575407	OBSCN	-2.131280681
PHKA2	2.261421721	LOXHD1	-2.131280681
LOC114109372	2.260245394	LOC114114539	-2.128995566
CCDC148	2.260245394	RHOH	-2.128420266
LOC114117323	2.260245394	CRYAB	-2.128361747
FBXL21P	2.260245394	COL8A2	-2.127936415
CDC45	2.260023487	PPFIBP1	-2.127614463
MSMO1	2.259259044	RGS6	-2.127342196
PMEL	2.258780694	CD84	-2.127137631
LOC114114502	2.258081986	SGCD	-2.126473593
LOC114109428	2.258081986	FAM131B	-2.126416169
PPP4R4	2.256366912	LOC105605337	-2.126382756
ENTPD2	2.250875636	LOC105615470	-2.12476106
COL7A1	2.250518459	KLHL3	-2.12264376
LOC106991936	2.249655699	STEAP1	-2.121704928
TUBA4A	2.247271492	PHLDB2	-2.120539552
TMEM120A	2.242871185	PROB1	-2.120091419
LRFN1	2.241903059	STEAP2	-2.119011266
ZNF536	2.238872744	FCGR3A	-2.118359325
POLA2	2.238819133	RNASE6	-2.11833526
CTXN1	2.23714529	LOC114117554	-2.11832025
LOC114111040	2.236581914	HLF	-2.117910292
SLC27A1	2.23411281	MAMDC2	-2.117729587
STAR	2.232336655	LOC105603376	-2.116666876
EIPR1	2.232009923	UGT8	-2.116666876
MGAT5B	2.231851455	LOC101108131	-2.116603544

ARHGEF16	2.231851455	NUAK1	-2.115653653
LOC105615212	2.231531182	ERBB3	-2.113874833
LOC105610729	2.231531182	TCAP	-2.112654738
LOC114110303	2.231531182	TNS1	-2.109140616
LOC106991287	2.231531182	FGL2	-2.106985885
PKIA	2.231414617	LOC101112356	-2.106524834
TUB	2.231353622	APP	-2.106236691
CDKN1A	2.231144007	SYT12	-2.106189316
APOA5	2.230197773	LOC101112584	-2.104790389
LOC105606686	2.230197773	GYG2	-2.104351377
ATP5MC1_1	2.227419847	DEPTOR	-2.10379716
LOC105611883	2.225505917	TRDMT1	-2.103249236
NR5A1	2.224120196	MAOB	-2.103204875
DCTPP1	2.222929266	SLC46A3	-2.102748155
LOC101105090	2.218267759	HAPLN3	-2.102160403
PFKFB3	2.216843087	ND5	-2.098810349
GAPDH	2.216182285	RDH10	-2.098623172
TMCO2	2.213835249	TFPI2	-2.098417669
GNRH2	2.212477219	METRN	-2.098254926
ISG15	2.211286015	COL28A1	-2.098006274
COL9A2	2.210502251	LOC114118418	-2.095730053
RUVBL1	2.209543283	LOC105613405	-2.095550473
LOC105608705	2.20951992	THY1	-2.094919622
SCP2D1	2.208875005	NOG	-2.093548588
GRK1	2.208875005	KIAA0408	-2.092326273
LOC114109673	2.208875005	LOC105612017	-2.091432948
LOC101122717	2.207310426	LOC114117625	-2.090449257
KIF1A	2.206468547	PDK4	-2.090258175
CRELD2	2.202881589	CACNA2D1	-2.08973093
LOC105602107	2.201835725	CARNS1	-2.089389538
DOCK3	2.200840816	LOC101106844	-2.089066631
CD320	2.200697065	GPER1	-2.089066631
RASAL1	2.197915412	LOC114114031	-2.089066631
EIF4EBP1	2.197168935	LOC105612881	-2.08553169
TREM2	2.196627691	PCGF5	-2.085501737
STRADB	2.194213239	ADCY5	-2.083975106
LOC105613573	2.193163113	LOC101121762	-2.082682688
PGP	2.193054961	MFAP3L	-2.080477347
LOC105602517	2.190658855	OLFML2A	-2.079579407
OIT3	2.189307349	PCMTD1	-2.078685662
LOC101117419	2.189295905	CD244	-2.077309473
LOC105605231	2.186653474	HTR1D	-2.076584119
RFC3	2.18586925	SKIL	-2.076267255
ROR2	2.185411316	FLNC	-2.075053108
NLRP6	2.184875085	HEY1	-2.074916647

SYT15	2.181302587	LOC114114565	-2.074878122
DNALI1	2.179100816	MMP28	-2.074550021
SRM	2.176543587	BFSP1	-2.073399465
CDH4	2.175186874	LOC105612575	-2.072966896
DDC	2.175186874	QPRT	-2.072885588
LOC105602076	2.175186874	LOC101109747	-2.070884134
CACNG7	2.173939819	TENT5B	-2.070012088
LOC105601929	2.171478916	LOC105616290	-2.069897123
POPDC3	2.171101223	SYTL2	-2.068413906
TOMM40	2.170889184	LAMA4	-2.066524061
GNLY	2.170460912	PMEPA1	-2.066046473
GAMT	2.169939532	C1QTNF5	-2.060963118
SRRM5	2.168692227	LOC101103472	-2.060415009
TSSK3	2.168188433	LOC101116085	-2.059024932
LOC101102735	2.168188433	CD86	-2.058865435
FAM186B	2.165621061	LOC101114216	-2.058072113
GJA1	2.164077453	LOC105605770	-2.057834651
ATP6V1E2	2.16347232	CRIM1	-2.056881348
DPF1	2.160078578	MTMR7	-2.055883083
MYO15B	2.160078578	PTAFR	-2.054836702
ISYNA1	2.153581473	LOC114110105	-2.054661762
XRCC3	2.14529193	SEC14L4	-2.053087095
EXOSC5	2.14269997	LOC114114863	-2.050759616
MAP1LC3C	2.142414762	PIWIL3	-2.050759616
GRIN1	2.14149294	CRABP1	-2.050446231
BMPR1B	2.140860588	RGS9	-2.049366257
MCM4	2.133763944	SLITRK5	-2.049219711
LOC114110430	2.127081571	MGAT4A	-2.048241488
TM7SF2	2.126715651	USF3	-2.047034997
LOC114116930	2.124131184	IL1RN	-2.046785571
CACNA1E	2.124060842	DAAM2	-2.04627665
LOC114110477	2.123981644	COL14A1	-2.045828941
CFAP44	2.121050032	SKAP2	-2.044011587
LOC114109729	2.120714689	LOC101107504	-2.042316638
SHMT2	2.119132425	TMED8	-2.042034551
LOC114118406	2.119048309	DUSP19	-2.038278077
LOC105611550	2.116980278	LOC106990281	-2.037907832
TMEM144	2.116552296	LOC105602261	-2.036795414
OLIG1	2.116552296	LOC101120630	-2.036605879
LOC114110669	2.116552296	KLHL42	-2.036268598
SLC18A2	2.113626617	TLCD2	-2.034794115
LOC105604901	2.113353115	CIDEB	-2.034505512
LOC114114902	2.113353115	C1QTNF6	-2.03383795
AP1M2	2.113353115	SETD7	-2.029255535
AKR1B1	2.112365708	MAPRE2	-2.028191154

MRPL40	2.112046578	AFAP1	-2.02782608
STARD10	2.109888449	RGS14	-2.026178866
C14H19orf48	2.109763088	BMPR2	-2.026078922
DHCR7	2.10951404	KLF7	-2.025109759
MORN2	2.109340947	SPARCL1	-2.024150551
LOC114117239	2.109104894	SCN2A	-2.023815812
LOC101103439	2.109104894	STIM2	-2.022409739
LOC114108718	2.109104894	HEPH	-2.022109276
C22H10orf90	2.109104894	CD274	-2.020979982
LOC114114824	2.109104894	UTP14A	-2.019953592
C6	2.109104894	RNF168	-2.019797579
CYP2A6	2.109104894	AOC1	-2.01905695
LOC105602454	2.109104894	ABCA4	-2.017777318
UCN2	2.109104894	KCNJ10	-2.017777318
MCMDC2	2.109104894	RBM24	-2.017289091
IPO4	2.106524142	ALDH1A3	-2.017048943
ARHGAP44	2.105604872	F3	-2.017045019
LOC105602212	2.10440951	S100A4	-2.016244919
CDCA8	2.103932199	UBASH3A	-2.016180458
CHAF1A	2.103873103	CREBL2	-2.014643245
BPNT1	2.103613468	SLC1A4	-2.014599069
LOC114118389	2.103190428	LOC114108830	-2.014131712
CENPT	2.103050594	LOC101109513	-2.013533979
TMEM177	2.101843629	LOC101117786	-2.011923375
ANGEL1	2.101014421	LOC114115866	-2.01140765
SGO1	2.097103461	LOC105609992	-2.01140765
MRPL55	2.096977897	MAS1	-2.01140765
LOC101111911	2.096504614	LOC114116396	-2.01140765
HSPBP1	2.095723086	GRIA4	-2.01131701
ENTPD6	2.094552215	COL4A6	-2.010014722
LOC114117580	2.094390166	TBC1D32	-2.009237106
ABCC6	2.094390166	QPCT	-2.007608472
PEAK3	2.094390166	DHH	-2.005812683
CCKBR	2.094390166	SPOCK2	-2.005622546
SPATA22	2.094390166	COBLL1	-2.003556807
STAC3	2.094390166	ATP7A	-2.003367747
GPX1	2.092090399	NEBL	-2.003202438
MOK	2.091542518	LRCH1	-2.002634853
ZFAND2A	2.090213998	GPX3	-2.002585395
CMBL	2.08995678	C1QTNF7	-2.001971782
DDX39A	2.088614036	LOC105608435	-2.001941192
LOC105607154	2.088393117	LOC114112697	-2.001941192
UCK2	2.087163497	PDZD3	-2.001941192
A4GALT	2.086040262	LOC114116440	-2.001941192
LMNB2	2.085093367	LOC105609002	-2.000624749

CCDC134	2.083757369	PRDM10	-2.000474893
LOC114114526	2.083542412	PLEKHG4	-2.000417061
LOC105609898	2.082074742	LOC101119648	-2.000016412
PSMC3IP	2.07915997	FRMD7	-1.998133914
POLE	2.079072304	NLRP1	-1.997268752
PLK1	2.076870936	FOS	-1.996807801
WRAP73	2.071413664	P2RY6	-1.996669822
SRRM3	2.069798703	CDK14	-1.996334053
NACA	2.066890355	CDH19	-1.99421752
LOC114114095	2.066578884	LOC114116177	-1.993925162
PLEK	2.064755559	WIPF1	-1.993461485
ANKRD9	2.064377871	CALD1	-1.993083866
CHST8	2.063938852	MET	-1.99306841
BLM	2.063876581	VSTM2L	-1.991702383
CENPV	2.063753276	FAM129C	-1.990545341
RIMS4	2.062421506	ATP10A	-1.990045566
MND1	2.061825782	SEPT5	-1.989315545
LOC114111193	2.061436275	LOC101123578	-1.988982138
SEC14L2	2.058363851	CCPG1	-1.988229996
TGFBR3L	2.056509853	NELL2	-1.98613903
TMEM229B	2.056495952	LOC101121570	-1.986096044
LOC114115994	2.056455225	ROCK2	-1.984819659
RYR3	2.05601357	TFEC	-1.98432551
LOC101106024	2.055433341	MMRN1	-1.981210538
LOC101120838	2.055433341	PEAK1	-1.980921894
CNMD	2.055231623	PDCD4	-1.980569654
TRAPPC6A	2.054225282	LOC105604155	-1.979502814
TMEM37	2.051794942	DKK3	-1.978554494
LAPTM4B	2.051793595	PDLIM5	-1.978435314
PRKCG	2.051791637	B4GALT1	-1.976809292
PEX16	2.051263316	PLEKHA1	-1.97637322
PCYT2	2.049147612	ZNF461	-1.972641411
TMEFF1	2.047884485	CD24	-1.97049532
NDOR1	2.045947842	IMPG2	-1.969830768
SKA3	2.045588326	CLIC4	-1.968047177
HES7	2.044298833	FAM160B1	-1.964854192
LOC101114167	2.044298833	BANK1	-1.962933263
LOC114109091	2.043333628	LOC105613357	-1.962189524
LUZP6	2.041751759	STAT3	-1.961889116
MXD3	2.041489719	CD74	-1.959959338
BUD23	2.035363585	TMEM245	-1.959771088
TMEM54	2.034161782	LOC114113122	-1.958337419
LOC105612888	2.033800885	NOTCH3	-1.956759001
ABO	2.033599557	FBLIM1	-1.955734614
SPATS1	2.032548335	DIAPH2	-1.955226488

POLD1	2.030558431	CACNA1B	-1.954992953
B3GALT1	2.030316578	LOC105606473	-1.954992953
CELSR2	2.029567692	LOC114116588	-1.954992953
LOC114113979	2.0271499	ASB4	-1.954992953
LOC105603051	2.0271499	PHC3	-1.953043034
MCM3	2.026505141	LOC114114564	-1.952107094
SHISA2	2.025743235	ABHD3	-1.949646457
LOC105610397	2.024869871	MECOM	-1.949031114
HIPK4	2.021884769	TACC1	-1.943966006
HAS1	2.021884769	NUDT4	-1.943933328
GRM4	2.021884769	DYRK3	-1.943243855
LRP2	2.021884769	PDGFD	-1.942764117
ELOVL6	2.019038711	NIPAL2	-1.942725366
FDPS	2.018793141	CD302	-1.941129057
ROPN1L	2.018300475	KLHL30	-1.940965254
PEMT	2.017427579	LOC105606370	-1.940599138
RDM1	2.015161546	CAV1	-1.940011972
SLC16A1	2.012316924	PPP1R12A	-1.939359606
CATSPER2	2.010930056	LOC106990149	-1.938710194
LRRC8D	2.010926722	NHS	-1.937482825
STMN1	2.010093793	EML1	-1.935039049
SNRPA	2.009583536	MATK	-1.932606504
SERPINA14	2.009264468	LOC101111868	-1.932517477
MISP3	2.009264468	KLF9	-1.930625997
GPR137C	2.008477753	LOC114110142	-1.929674332
VWCE	2.006297361	LOC101104568	-1.92932934
PPM1J	2.006297361	MYBL1	-1.929326236
PRKAG3	2.004932751	LOC101109492	-1.92910809
SPC25	2.002722425	NR3C2	-1.928932741
LOC114118072	2.002261895	CCL21	-1.92882478
LOC114110578	1.998923052	TAGLN	-1.926619493
ADGRG1	1.997501785	ADAMTS5	-1.925910713
TRAP1	1.996429865	MBNL3	-1.925469743
KRT7	1.993673238	TNK1	-1.925005182
LOC105602706	1.99161013	MYL9	-1.924218172
GRIK3	1.99161013	RCAN2	-1.924146918
LOC114110462	1.99161013	MYH11	-1.922836914
LOC105615257	1.99161013	CNST	-1.922581693
KIAA1257	1.99161013	PTGR2	-1.922254875
UBE2C	1.989657191	LOC101123120	-1.920800385
LOC106991047	1.989550931	ENDOD1	-1.920566638
LOC114118856	1.989217727	LAMA5	-1.919867802
CERS4	1.984863636	LOC101103090	-1.919801456
CMSS1	1.984842919	LOC101104012	-1.919690941
PRKAR2B	1.984734988	LOC101123612	-1.919660532

RPL18A	1.983557519	LPP	-1.919444582
LOC105615393	1.98188577	LOC114113971	-1.915656707
LOC114116891	1.98159317	ADAMTS18	-1.915429542
MGMT	1.978848624	LOC101104297	-1.914353793
TVP23B	1.976721678	PTGS1	-1.909248497
SKA1	1.975944272	COL22A1	-1.909035667
CRMP1	1.974132656	PLEKHA2	-1.908781344
VPS37D	1.972749311	FAM217B	-1.907881643
GTSE1	1.971366591	ARL5A	-1.905479739
NUP205	1.9704381	COL6A3	-1.904761968
PCK1	1.970031216	PENK	-1.904350877
SHC2	1.970005504	LOC101120326	-1.903943446
LOC114118506	1.969623091	OSBPL3	-1.903678769
LOC101107475	1.969469723	TNFRSF11B	-1.902954462
FBXO17	1.966752358	TGFBR2	-1.902580038
HOPX	1.966415229	CSGALNACT2	-1.901144204
MARCKSL1	1.963334275	TES	-1.899419128
DNASE1L2	1.961977055	LOC101106372	-1.899017796
KIFC1	1.961534829	LOC114109079	-1.89808206
TECR	1.960823652	F13A1	-1.897456091
B3GALT6	1.959264095	FBXL20	-1.897349046
SAPCD2	1.959080995	IGSF10	-1.897068615
TROAP	1.958820186	YPEL2	-1.896702366
YDJC	1.958352353	DMPK	-1.896317483
APBA2	1.954676314	CACNA1G	-1.896098594
LOC101105400	1.954676314	LOC101122496	-1.895746392
NUDT16L1	1.953904598	PLVAP	-1.895569925
ALDOA	1.9519779	CD4	-1.895362623
SCRN2	1.950135717	LIMA1	-1.89521997
RUNDC3A	1.949843175	SCIMP	-1.89388265
RRP9	1.947100991	ZNF157	-1.891094987
FAAH	1.946883765	LOC114116895	-1.891094987
GPX4	1.946881228	LOC106991039	-1.890772828
TRIB2	1.945503982	LOC114110571	-1.890562501
DTYMK	1.945118069	LOC101114959	-1.890201521
ECI1	1.944743877	ESR1	-1.889943254
TMEM97	1.942134535	PGM5	-1.889420238
TRIP13	1.941542777	VIPR1	-1.888996041
FAM234B	1.941420238	TFPI	-1.888528972
LOC114114570	1.940982013	TRPM6	-1.888041623
LOC101120174	1.940257409	LOC105608393	-1.88694037
HSD17B14	1.940257409	MBNL1	-1.885169569
SERPINB12	1.940257409	C15H11orf96	-1.884918922
LINGO2	1.940257409	PPIC	-1.882031928
CLCN1	1.940257409	LOC114116407	-1.881624707

LOC105603310	1.940257409	SHOX2	-1.881240853
LOC114116191	1.938186441	LOC114114549	-1.878044229
KCNF1	1.93652226	TMEM169	-1.877297981
DUSP26	1.936464897	LOC114117632	-1.877297981
LOC105609026	1.935656856	LOC114116378	-1.877297981
ENDOG	1.935365762	ZMAT1	-1.877297981
ZMYND10	1.934570923	CDRT1	-1.877297981
GPR68	1.934570923	ARHGAP24	-1.876643578
MVK	1.934359312	HOXA6	-1.87641126
LOC114113887	1.930853244	LOC101111623	-1.876304179
PLIN5	1.926968722	ANKRD50	-1.875870976
SLCO3A1	1.926961536	LOC105605611	-1.875850772
CATSPER3	1.924832235	PRNP	-1.874511597
SPATA21	1.924832235	PLCB1	-1.874439031
LOC105610832	1.922617813	PPP1R1B	-1.874046292
MSH4	1.921163174	NPR1	-1.873512456
ABHD11	1.919517531	TUBE1	-1.871752098
DEGS2	1.916251937	ALDH1A2	-1.871560866
ZPBP2	1.915396843	SEMA3F	-1.871396466
PSD2	1.915396843	LOC101114551	-1.871195053
KCNK7	1.915396843	CLOCK	-1.870712107
FA2H	1.915396843	SLC3A1	-1.869467395
CDC20	1.912565468	KIAA0040	-1.869291761
NOCT	1.912340311	RASGRP4	-1.867299079
EMG1	1.911527014	TRPC6	-1.867188628
NRTN	1.911283655	ARRB1	-1.867004294
PRDX1	1.910136297	UEVLD	-1.865637453
KLHL25	1.909365586	PLOD2	-1.864881315
ISLR2	1.908762779	DNAL1	-1.864614381
ADI1	1.908388338	TBC1D9	-1.863200318
LOC114112996	1.907507498	HOXD11	-1.860416064
RANBP1	1.905072736	ID4	-1.860096491
GCAT	1.905067676	ANKS4B	-1.859654007
LOC114111217	1.904443001	LOC105603814	-1.859464334
LOC105616822	1.903474114	C3H12orf60	-1.859193954
PSMB3	1.902860011	LOC101109733	-1.858052399
CCDC85A	1.901943218	LOC105613784	-1.858052399
MARVELD3	1.900981883	SAV1	-1.856585217
DAPL1	1.900981883	TINAGL1	-1.853790132
RHBDD1	1.89936177	KCNB1	-1.853233434
LOC101103967	1.898277201	GTF3C4	-1.851665606
VAX2	1.898277201	P4HA3	-1.85160471
ENO2	1.89644791	BATF2	-1.851463742
CPS1	1.896105399	CDK17	-1.85009103
CCNF	1.894162434	MARCH3	-1.848617671

ACCSL	1.893791166	SLC51B	-1.847903214
MTG1	1.893251551	DAAM1	-1.847590766
LOC106991653	1.892193128	HEY2	-1.846359448
LOC114117644	1.892193128	GABRA2	-1.846128252
LOC114114826	1.891571677	LOC105615521	-1.846128252
ZNF365	1.889505561	CPNE8	-1.845010805
MRPS18B	1.889324614	DIXDC1	-1.844813948
LOC114110282	1.889189648	CD22	-1.844474298
GAL3ST1	1.88872422	ITGB1	-1.843726844
IQCG	1.885338633	SCAI	-1.842298208
PCBD1	1.883224837	SH2D4B	-1.842298208
LOC105612127	1.882914279	CCDC7	-1.842298208
GINS1	1.879701527	C11H17orf67	-1.842298208
E2F1	1.874196023	NHSL2	-1.842238019
DEPP1	1.874097346	CLIP1	-1.840585005
ALYREF	1.870745077	PILRA	-1.840321824
FSD1	1.87005487	LOC105607239	-1.839871061
LOC105608370	1.867597353	OMD	-1.839018316
LOC114109536	1.867597353	SERPINI1	-1.838885076
NTMT1	1.866347997	C5AR1	-1.838884563
SLC5A6	1.865850244	KLHL24	-1.838088273
NOP56	1.865839964	STX1B	-1.835789289
LOC101111394	1.865452935	LOC114113898	-1.834184154
LOC101117087	1.864484002	LOC114114004	-1.834053594
LOC105607734	1.863924572	TLR7	-1.834053594
CHTF18	1.862388595	STAC	-1.834021441
LOC106991502	1.860560574	SLC4A8	-1.833395252
SLC16A9	1.86029791	COL18A1	-1.8333198
SLC39A5	1.859052536	LOC105606919	-1.832474488
SLC41A3	1.858052485	ATP2B3	-1.83083235
AURKA	1.857006784	JCAD	-1.83000686
LOC105607546	1.856312798	VAV1	-1.829306552
LOC101111922	1.856142108	PGAP1	-1.828979596
F2RL3	1.854812748	ITGA4	-1.828309386
SSPO	1.854812748	CHP2	-1.825709506
LOC105604106	1.854812748	LOC114110944	-1.825531452
FITM2	1.854446688	CDC37L1	-1.82535339
LOC114112936	1.854229179	LOC101122014	-1.824220925
TMEM151B	1.85324822	TRPV4	-1.824214829
RPS29	1.853021476	LOC114114000	-1.82421327
LOC105603203	1.851952101	LOC114110969	-1.824212643
NSD2	1.851315143	LOC101108887	-1.823489751
ACOT7	1.848872002	PALLD	-1.822931568
LOC105605798	1.847675583	HOXD3	-1.821692232
GLDC	1.846371072	PROS1	-1.821515526

GATA4	1.842999824	FRK	-1.820285848
EXOSC4	1.842394037	LOC105605988	-1.820285848
RHBDL2	1.841958769	LOC101115905	-1.81971267
LIPT1	1.838706183	SKAP1	-1.819640744
C1H1orf194	1.838514605	HSPA12A	-1.818748832
KATNB1	1.837650233	EFNA5	-1.817817781
PIH1D3	1.836834074	HTRA1	-1.817540237
EXOSC6	1.834953561	ACTA2	-1.815701043
CYTL1	1.83423217	LOC105611082	-1.81431246
PFKL	1.831932539	PDCD1LG2	-1.81385113
TACC3	1.830687397	MAFB	-1.813706061
ROBO2	1.829904312	CCNYL1	-1.811372679
GPI	1.829741182	CRISPLD2	-1.811017645
BUB1	1.829556022	MB	-1.810545625
EBF3	1.82800765	LOC114114527	-1.810309305
SH2B2	1.827176148	COL1A1	-1.810055196
MRPS2	1.826767974	IRF5	-1.809817701
WDR5	1.82650688	LOC101120395	-1.809372165
GABRB2	1.826486066	UBE2H	-1.808721894
LOC114109018	1.826273561	LOC105608991	-1.808578017
FAM169B	1.824901009	LRP6	-1.807863424
LOC114117637	1.824901009	C3H2orf40	-1.807639432
DBF4	1.821819017	CAST	-1.807177739
CARS2	1.816928877	ABCC3	-1.80655493
TMC4	1.816726407	RECK	-1.806148602
CKS2	1.81641051	SHPRH	-1.805606285
LOC101120455	1.816363941	ARHGAP30	-1.804678278
MPV17L2	1.816001435	SNX18	-1.804497013
SUSD4	1.815971122	NEGR1	-1.8035824
CCNB2	1.81531195	SLC2A12	-1.801451656
FAM110B	1.814938211	SOX6	-1.801427883
RAD51AP1	1.813606693	GJA4	-1.7974783
WNT11	1.813351431	AIF1L	-1.797045181
TRAIP	1.813052235	USP43	-1.796731207
TCEA3	1.811346362	LOC114111361	-1.796731207
PPCDC	1.809377982	L1CAM	-1.796495207
KCNIP1	1.809078753	GIPC2	-1.796398312
RUVBL2	1.808854569	JAG1	-1.796038424
CNTN4	1.808275382	CCL5	-1.795701825
LHPP	1.80737164	SLCO2A1	-1.794639084
C2CD4B	1.806960771	CELSR1	-1.793854073
PTCH2	1.806464046	FGFR3	-1.793452836
LOC114110579	1.806018656	LOC114109610	-1.793248516
PHETA1	1.805985171	CORO2A	-1.792671923
NAA38	1.805905439	LOC114111468	-1.792540935

CENPW	1.805848316	ABCA6	-1.792418042
ABCD1	1.804842286	F8	-1.792259758
ORC6	1.803463163	LOC105603639	-1.789641216
SLC5A2	1.803272308	LOC101110257	-1.789606352
GINS4	1.802478667	LOC114114546	-1.789606352
KCNA4	1.80121626	PLA2R1	-1.789455538
CEP78	1.80087664	TRAF5	-1.787873816
NOB1	1.800536261	DNAJB4	-1.787563705
PLA2G2F	1.800417958	HSPA2	-1.786246883
LOC114116101	1.800417958	LOC105606290	-1.786134916
DEUP1	1.800417958	PTPRR	-1.785921669
LOC114110643	1.800417958	ARNT	-1.785802634
SLC25A13	1.799763506	FAM185A	-1.785484347
RTN1	1.799689224	LOC106990140	-1.78507257
STK32C	1.798127196	ABLIM2	-1.785044512
DDR1	1.795452161	LOC114116381	-1.7848259
DGKD	1.793400394	KLHL20	-1.784692429
LOC101112491	1.790522554	SMIM14	-1.783500978
LOC101116059	1.790287796	HYKK	-1.783190117
LOC105607560	1.789463527	CLDND2	-1.783090743
PGLS	1.785500781	HEATR5A	-1.782709486
FANCD2	1.783629378	LOC114114015	-1.78259618
LOC114109085	1.782734979	TPPP	-1.781739131
KIF2C	1.782085031	IFIT1	-1.781496976
CGN	1.781289823	THEMIS2	-1.781048974
MLXIPL	1.781220828	TAFA5	-1.780299274
LOC101114535	1.780005922	F2RL2	-1.779857928
FIGNL1	1.776947903	HS2ST1	-1.779857928
PEX7	1.776753553	LGALSL	-1.777508917
CCND2	1.775682485	EPSTI1	-1.777126988
LOC114115318	1.775425436	FNIP1	-1.776576043
SQLE	1.775334564	CAPN3	-1.775376538
GPNMB	1.774943659	SLC35F1	-1.774962091
GPSM1	1.774428522	TENT5A	-1.774529251
TRAF4	1.771224082	RUBCNL	-1.774000845
WDYHV1	1.769951028	ANK1	-1.773387276
LAMC2	1.769679904	ZNF185	-1.771515462
NDUFA4L2	1.768059403	OVAR-DRB1	-1.768383467
ZMAT4	1.767355173	SLC4A7	-1.76707011
TAFA4	1.763547529	DPY19L4	-1.766388696
SLC27A4	1.763229323	RASAL2	-1.766185447
LOC105613348	1.762725834	CAV2	-1.765906498
LOC105605165	1.762725834	RDH5	-1.765682678
KLHDC4	1.760640977	LOC105607593	-1.765036062
B4GALNT4	1.759507465	LOC101106719	-1.764398805

LOC114112674	1.758123114	SSTR2	-1.763744452
EZH2	1.757507928	SLMAP	-1.763732928
MAPK13	1.757098242	ZEB1	-1.761374933
LOC114110821	1.756982333	CDKL5	-1.760846213
CHAF1B	1.756804514	IGF2R	-1.760500321
LOC105603432	1.755534187	MX2	-1.760396353
TOR2A	1.755335964	FKBP7	-1.759396361
ZMYND19	1.755211732	LOC101115927	-1.759173169
ZFPM1	1.754318933	LOC101123244	-1.758581404
CD226	1.753293845	BTK	-1.75815505
DOHH	1.752705821	PHACTR2	-1.75748851
LOC114118505	1.752580797	LOC114112948	-1.757157347
SCARB1	1.752533589	GALNT5	-1.756884796
RABL6	1.752176406	LOC114110806	-1.756806909
SLC30A2	1.751744155	CH25H	-1.756394166
LOC101116841	1.748996528	LOC114110574	-1.755614139
LOC114113146	1.748996528	LOC105603536	-1.755614139
MYRFL	1.748996528	TMEM61	-1.753905169
GFRA4	1.748996528	PRR16	-1.753245245
LOC101109422	1.748996528	IL7R	-1.751241061
TBX15	1.748996528	LMBRD2	-1.75075594
LOC101112434	1.748996528	THBD	-1.750696435
TMEM212	1.748996528	NFAT5	-1.75023997
MTCP1	1.748996528	GCGR	-1.749677918
PSRC1	1.746149231	NAB1	-1.749480472
PRR5	1.745947523	PAG1	-1.74867078
NUDT8	1.744755104	LOC105608443	-1.748329146
GNA14	1.743824198	ST6GALNAC2	-1.748295654
EEF2KMT	1.742542885	ANLN	-1.747573166
LOC114117767	1.742423219	FBXO32	-1.747568956
NETO2	1.74098065	NRXN1	-1.747370975
IL1RAPL1	1.739299003	KCNJ3	-1.746332899
LOC114117970	1.739299003	LOC114112473	-1.746138119
TIMM17B	1.73810772	MID2	-1.746093671
LRFN5	1.736962722	TAL1	-1.745735188
GUCA1A	1.736583151	LOC105607466	-1.74550352
WDR34	1.735526152	FAM43B	-1.744002396
LOC101116828	1.735058474	TCP11L2	-1.741458742
RAB15	1.734736342	SYTL3	-1.73876974
KCNK2	1.734016043	SMOC1	-1.738737463
MRPL12	1.733725715	PCOLCE	-1.738663676
MACROD1	1.733129202	NTNG1	-1.73813387
SESN2	1.732398554	CASQ1	-1.735952342
IGF2BP3	1.731158314	MARCH1	-1.734554457
IGFN1	1.731158314	BCAS1	-1.734418481

PSMB9	1.729735012	PALMD	-1.733707074
AMACR	1.729308951	CHRNE	-1.732553195
LOC114110581	1.729269398	CYBB	-1.731349701
DCPS	1.728295409	RXRG	-1.7298064
GFER	1.725321177	GFRA2	-1.729377856
LOC105611240	1.723810862	ATP2A3	-1.729225915
TAZ	1.722474719	LYST	-1.728397366
COQ6	1.721733174	TAC3	-1.727733857
MCOLN1	1.721228262	TRAF1	-1.727707734
ZBTB45	1.72051042	SYNPO	-1.725849416
EEF1D	1.72041014	LOC101112990	-1.725779519
HPDL	1.719037804	B3GNT7	-1.72450523
MELK	1.718390669	RETN	-1.724395826
LOC114114137	1.716420228	DSG2	-1.723311424
LNX2	1.716326394	HCN3	-1.723311424
CCDC34	1.71613526	PCP4L1	-1.7184581
NME3	1.715411594	LOC114113871	-1.718161306
STX1A	1.713882367	MADCAM1	-1.718161306
LOC105607506	1.710464156	ARL4D	-1.716765729
LOC105605439	1.710464156	LOC101122718	-1.716731329
LOC105610613	1.710464156	HCRTR1	-1.716483589
APRT	1.710278828	SOX5	-1.716195119
TMEM176B	1.709902527	CDC42BPA	-1.714271849
TONSL	1.707489757	EGFL8	-1.7139874
LOC105616404	1.70569452	PRRX2	-1.713317891
EFHC2	1.705484321	ANO6	-1.712856483
HDAC10	1.705229419	GNB4	-1.710797455
NXT1	1.704866919	SGK1	-1.710667771
CSNK1G2	1.704589243	LOC114114846	-1.710593613
LOC101123403	1.703672248	VLDLR	-1.710442972
PITPNM3	1.703672248	CSGALNACT1	-1.710418249
LOC114110838	1.703672248	CD59	-1.710375335
WEE2	1.703672248	LOC114117609	-1.710112629
SMPDL3B	1.703672248	SYN1	-1.709993891
PRMT5	1.703568646	LOC114117268	-1.709825204
EEFSEC	1.703271121	DES	-1.709357671
RASD2	1.703193996	EFNB2	-1.709162751
DHODH	1.702586453	ZFC3H1	-1.708995825
MRPL38	1.702216026	CTSW	-1.708559193
CDC25A	1.701252342	PLP1	-1.707606789
FBL	1.70101725	PTGES3L	-1.706955424
LOC101117955	1.700785932	CHRM3	-1.705819362
LOC114110474	1.699243476	WDR66	-1.705819362
LOC105603708	1.699176452	LOC114113048	-1.705819362
LOC101108147	1.699176452	CCDC171	-1.705325463

SURF6	1.699119793	LOC101102642	-1.704938788
ODC1	1.698223416	LOC101118645	-1.704506373
TSSK6	1.697151252	CCDC186	-1.704334875
TRMT1	1.696811887	DLL1	-1.702832352
C13H20orf27	1.696132874	PROCR	-1.701613928
CFAP45	1.695255532	LOC101123376	-1.700720632
LOC114115334	1.695255532	LOC114114830	-1.699942591
C4BPA	1.695255532	LOC101121769	-1.698109244
MFSD2A	1.694868593	LOC114109386	-1.698109244
WDR91	1.694101623	CASK	-1.697941999
LPAR2	1.693049768	PGF	-1.69741245
KRT8	1.691543494	SYK	-1.696358205
ADRA2B	1.68975983	TUBA8	-1.696129966
LOC114112112	1.689411658	TLL1	-1.69586229
KCNJ4	1.689411658	LOC101108113	-1.695858926
MOCS1	1.68935596	LGR4	-1.695250285
CPEB1	1.688224796	DPH6	-1.694955535
LOC101111035	1.688103627	HPGD	-1.690788285
LOC101103726	1.687690876	LTBP1	-1.690515131
NXPH3	1.68761682	AFAP1L2	-1.69050877
LRFN2	1.686324067	LOC101112543	-1.69031159
RGS9BP	1.686324067	LOC114113838	-1.690268837
TTLL4	1.685752914	G6PC	-1.687635178
LOC114116722	1.684463201	IGFBP7	-1.687055427
CHCHD4	1.684131449	SP7	-1.686830402
LOC105603710	1.683597673	CLEC7A_1	-1.686295894
ELOVL2	1.683597673	AHR	-1.685973948
LRRC3B	1.683143765	ACTG2	-1.685570908
EPN1	1.682940442	FILIP1L	-1.684610004
SYTL1	1.68163468	THBS2	-1.683908757
SCAND1	1.68154975	RAB3B	-1.683714659
KCNH2	1.681193463	PSTPIP2	-1.683361662
DGKI	1.680440485	CCDC88C	-1.683087191
TAMM41	1.680371509	SPEG	-1.682491804
ASXL3	1.67774224	PDLIM7	-1.682329514
PMPCA	1.675769161	SLC20A2	-1.682223608
LOC114115351	1.675475791	ERG	-1.682080697
LOC105616860	1.675475791	DYNC1LI2	-1.681925295
DCDC1	1.675475791	GPR146	-1.681687252
FOXA3	1.675475791	MYLK	-1.681265481
KIF5A	1.675475791	BROX	-1.681008399
ATPAF2	1.674879767	ASB12	-1.680434442
LOC114110055	1.674866065	LMO7	-1.679548122
FGF9	1.674755698	XIRP1	-1.679288373
MRPL28	1.674484951	KLHL14	-1.679288373

ABAT	1.673516317	UACA	-1.677041656
LOC101115115	1.673226497	CERCAM	-1.67655499
C13H20orf96	1.67317018	LOC101117657	-1.676497758
ALDH4A1	1.672301475	LOC101122475	-1.674880672
SLC2A1	1.669620687	SNTA1	-1.674346329
NT5DC2	1.669495564	CPT1C	-1.673454168
LOC105602007	1.669409504	AKAP9	-1.673105151
DDX11	1.669356282	WDR17	-1.670751401
LOC101114310	1.668953594	LOC101113728	-1.669942747
LSM4	1.668608319	RAPGEF6	-1.669784
PSTK	1.6675059	LOC105609220	-1.669146625
CAMSAP3	1.665885994	TULP4	-1.668279718
NOP14	1.665665222	LOC101112168	-1.667975693
STAG3	1.665238989	LOC105613298	-1.667734221
IFRD2	1.66497899	LOC114109442	-1.667734221
LOC114114091	1.664787168	LOC114108820	-1.667472945
LOC105608597	1.6643341	RARB	-1.667400372
LOC114114903	1.6643341	LOC114114763	-1.667022717
C6H4orf48	1.660618795	PTPRE	-1.666022834
TKT	1.659646891	PARPBP	-1.66516318
TSSC4	1.65811197	MEIS1	-1.664958889
SHD	1.657731411	PCDH19	-1.664548315
JMJD7	1.654917058	LOC114109713	-1.664156382
METTL27	1.654565228	LOC101114469	-1.663438203
TIGD6	1.654165617	GDAP1	-1.662716357
LOC101105860	1.649033211	ADAMTSL2	-1.662614107
SYT2	1.644918458	PDE4B	-1.661582856
LOC106991803	1.6438658	SIRPB2	-1.661577762
PAXX	1.643560502	LOC101116845	-1.66092504
CLEC19A	1.641953747	SCARF1	-1.660677639
PDSS1	1.641808472	ZBTB32	-1.659344044
TPI1	1.641712269	SOX8	-1.656573863
KIF4A	1.637050647	IL1RL1	-1.656451688
RPP21	1.634299644	FYB1	-1.655857642
PGBD5	1.633397205	CYLD	-1.654704499
LOC114109437	1.631674573	KIF25	-1.653226075
LOC114114101	1.631674573	CGNL1	-1.652690478
CA13	1.631674573	TMEM68	-1.651426115
BIN1	1.631560334	RFX5	-1.651276897
LOC114113893	1.630882362	CCL22	-1.650701029
FAM181B	1.630702036	SLC44A5	-1.650701029
PABPC4	1.630199614	IL12RB2	-1.650701029
AHCY	1.629979278	MAP3K20	-1.650579759
RFC2	1.628448489	EPAS1	-1.649637603
MYC	1.627975896	CRYBG1	-1.648852342

MKI67	1.627641912	ALKAL2	-1.64861018
STAMBPL1	1.627538049	LOC101118600	-1.64861018
CLCN2	1.625665308	CD200R1	-1.648120571
CBLN2	1.623355567	TTC32	-1.647902098
NNAT	1.621193507	PTN	-1.647150687
PAGR1	1.621148117	MRGPRF	-1.646913544
LOC105614707	1.620738815	LEPROT	-1.646514092
SPR	1.620150088	LOC114115302	-1.646225845
LOC101121054	1.619743107	PWWP2B	-1.646022148
RRP1	1.617830403	FRMD4A	-1.645711022
BLVRB	1.617724135	LOC114114073	-1.645005351
MEIOC	1.61665205	COL20A1	-1.645003645
CSKMT	1.615999045	TF	-1.644646782
LOC114108599	1.615595002	LOC101105484	-1.6441147
ANO4	1.614774943	NBEAL1	-1.643346496
FAT2	1.614511707	SOX9	-1.642769771
LOC101108817	1.613765835	LOC101105533	-1.642108016
LOC101109593	1.613740281	CXHXorf21	-1.642108016
B9D1	1.611905543	ARSK	-1.6420996
NOL6	1.611759676	ACVR2A	-1.640563727
RAP1GAP	1.61113504	PMAIP1	-1.639526274
ATP13A2	1.611113999	RNF112	-1.639460355
MST1	1.610624902	ITPKB	-1.639278907
ADGRA3	1.604184371	PLAGL1	-1.637025696
LOC114108707	1.60382538	USP49	-1.636266479
GABBR2	1.603799247	BICC1	-1.635928057
MRPL13	1.601872104	PARD3B	-1.634282915
CLPP	1.601546389	LOC101106528	-1.633705234
CENPS	1.60008703	CYP2J	-1.633349997
XIRP2	1.599893601	LYSMD3	-1.633126578
LOC114116429	1.599893601	NFIA	-1.633096651
SRD5A1	1.599005349	AMOT	-1.631602312
MYCBPAP	1.597222956	LOC101103343	-1.630590886
LOC105607806	1.596278643	LOC101103720	-1.630567668
FPGS	1.594656822	ADGRG6	-1.629683746
SLC28A1	1.594350365	ETFBKMT	-1.629024967
SLC6A4	1.593791494	HMCN1	-1.627969854
CFAP100	1.593542487	LOC101120993	-1.627550827
LOC105613621	1.593542487	ARHGEF6	-1.626789955
THAP7	1.593216271	ADAM33	-1.626187093
ZNF296	1.592531284	ADAP2	-1.625543943
FAM83H	1.589580812	XPNPEP2	-1.625170577
PCNA	1.588759146	GULP1	-1.624191274
PRMT1	1.588119688	NAPB	-1.623841527
LOC114114003	1.586760272	YY1AP1	-1.622808538

LOC101116852	1.585940936	TMOD3	-1.622633318
LOC105602080	1.585306635	SERTM1	-1.622374426
NOC2L	1.583678356	HIPK3	-1.622000669
BTBD6	1.583636467	SCRN1	-1.621758532
LONP1	1.583136337	KLHL6	-1.621616963
LOC101105019	1.581977078	LOC114114566	-1.62102027
LOC114112914	1.581977078	MLLT3	-1.620780695
KLC2	1.581355878	KHDRBS3	-1.620718074
TPBGL	1.580733561	GDPD5	-1.619662559
LOC105607931	1.580733561	CLCC1	-1.618414737
RAD51	1.580472561	SEL1L3	-1.618069674
VARS	1.580438236	LOC114116167	-1.617708866
BRCA1	1.579791492	RHOB	-1.617409235
CCDC154	1.579501438	LOC114114793	-1.617233761
POMT2	1.578123749	ZBTB1	-1.617230528
LOC101106040	1.577943325	EEA1	-1.616279574
TALDO1	1.577672187	CD3E	-1.616018093
BEND6	1.577432354	ZBTB46	-1.615380425
SETD9	1.576604134	BACH1	-1.615279373
SLC7A1	1.576587277	MANBA	-1.614786837
LOC105609312	1.574895968	CXCL9	-1.613809119
TDP1	1.572612287	ALCAM	-1.613481237
ASPSCR1	1.571757153	LOC114114876	-1.613269601
RPUSD4	1.567973442	SMAD6	-1.612902757
PLEKHJ1	1.567683778	SLC27A5	-1.612656892
LOC101121639	1.567600948	C24H16orf54	-1.612389182
NAA10	1.56694116	RHOQ	-1.611514524
BIRC5	1.566241874	STK17B	-1.610643022
TNFRSF6B	1.566206992	XG	-1.610587777
GADD45GIP1	1.56602677	SH3TC1	-1.61025993
PLXNC1	1.564788169	ATP2B4	-1.610165906
NR2F6	1.56459553	LOC106990525	-1.609884288
TP53RK	1.564252603	SMURF2	-1.609807054
PLPPR3	1.562720963	OSBP2	-1.609485408
TMEM150A	1.558678059	LOC101103766	-1.609170212
PHB2	1.558351072	CST7	-1.609170212
SREBF2	1.555386178	SMARCA2	-1.608873875
RRP7A	1.553012771	CSAD	-1.608671212
FDX1	1.552553739	LOC114114074	-1.608253162
HRAS	1.552270044	DSEL	-1.607770326
MCM7	1.552200768	P2RY8	-1.607032256
IFT22	1.55163285	LOC101119572	-1.607032256
LOC114110265	1.55158434	LOC114117766	-1.607032256
DLGAP3	1.550638604	BMP2	-1.60686477
SLC25A39	1.550086714	HEG1	-1.606801813

TMEM126A	1.549547648	ZMYM5	-1.606233495
AGBL2	1.548683499	NOD2	-1.605850732
LOC101103182	1.54847623	TRIM56	-1.605736172
SLC4A11	1.54847623	DIO2	-1.605510806
ELAVL2	1.548253083	LOC114112673	-1.605326488
LOC114108997	1.547751255	NKX3-1	-1.604975202
HRH2	1.547751255	FZD7	-1.604662469
QPCTL	1.547625673	SEC24D	-1.603797603
NME4	1.547192203	GNG8	-1.603348879
LOC101117343	1.547158634	LOC114113812	-1.602775461
C2H9orf43	1.547158634	LOC114115280	-1.602775461
LOC101113599	1.546998632	LOC114110120	-1.602775461
TUFM	1.546655635	LOC100037664	-1.602775461
LOC106991980	1.546037105	WNT10B	-1.602775461
RYK	1.545641533	TNFAIP3	-1.602105209
LOC105604300	1.545070908	TIMD4	-1.601481436
LOC114113005	1.544497839	CCDC191	-1.601460757
SCN8A	1.544497839	RASL12	-1.601146756
NHLRC1	1.544273799	HACD1	-1.600674842
ZNF414	1.543289546	ADAMTSL1	-1.600546346
H2AFZ	1.543154541	EEF2K	-1.599163871
LOC114108814	1.543107994	ATP8B1	-1.598923941
HYOU1	1.54298184	LRIG3	-1.598365744
LOC105602770	1.542254033	LOC105610487	-1.598041732
AIFM3	1.542254033	SPAG9	-1.596085722
LOC114110125	1.541259023	YOD1	-1.595052549
CA14	1.541259023	SEMA5A	-1.5939665
NEK2	1.540975108	FAM3C	-1.593810057
SSRP1	1.540865788	RFX7	-1.593031541
LOC101102413	1.54008487	GATA5	-1.592376311
ADCK1	1.53969905	RASGRP2	-1.59149026
ACAD9	1.539458561	ARID4A	-1.589750714
DLX4	1.538678409	GOLGA4	-1.589242799
KCNH4	1.538678409	EPM2A	-1.588019575
LOC114112887	1.538678409	LAMC1	-1.587811323
ONECUT1	1.538678409	HCLS1	-1.586595378
LOC101116968	1.538678409	LUZP2	-1.586342213
CIAPIN1	1.536433868	LOC114111428	-1.586342213
LOC106990587	1.534824763	GATA2	-1.585337558
LOC114110144	1.534824763	ADRB2	-1.585229815
LOC114117542	1.534824763	LOC105610195	-1.584388704
LOC105608080	1.534824763	CD300LF	-1.584388704
VSIG8	1.534824763	GFAP	-1.58385038
LOC114116375	1.534824763	PER2	-1.582494572
SLC12A5	1.534824763	LOC114115624	-1.582492187

MYL7	1.534824763	ICAM5	-1.581494713
LOC105606390	1.534824763	CKB	-1.580793879
RANGAP1	1.53389964	SYNE1	-1.579644362
CDPF1	1.532758093	NF1	-1.57936237
NSMCE3	1.532630513	KMT2E	-1.579281715
MARCH9	1.530878207	ADAM19	-1.579265872
LOC101111367	1.530025316	MIGA1	-1.579072888
ECHS1	1.529559781	OLFML1	-1.578433804
TMEM201	1.528609791	LOC101108321	-1.578094075
INCENP	1.525966704	RFESD	-1.576874014
EFCAB2	1.525761394	CPEB2	-1.576801871
NUDT14	1.525580561	SLC26A7	-1.576399903
GADD45A	1.525308101	ZNF704	-1.575785197
LOC106990963	1.524515335	MINDY2	-1.574469852
MZT2B	1.523896491	CSPG4	-1.573165324
TRABD	1.523734655	PDE1C	-1.573023774
C2H9orf40	1.523708187	LOC105610993	-1.572854607
EXOSC7	1.523069354	PIK3AP1	-1.572443725
MYOM3	1.522961732	THBS3	-1.571892367
RTKN2	1.522738891	ATP2A2	-1.571840818
FBP1	1.522738891	CEMIP2	-1.571805291
GPR88	1.521372043	PIK3R6	-1.571793972
SARS2	1.520234591	FHDC1	-1.571467126
RNASEH2B	1.520047749	LOC101109219	-1.570127215
LOC101113696	1.518078521	STAB1	-1.569962727
DUT	1.51803374	RPS6KA3	-1.569353861
MRPL21	1.517817597	LOC114110097	-1.568226842
MRPL23	1.517743421	ELK4	-1.567736847
TSGA10	1.517596016	LOC114110110	-1.567304619
LOC114108791	1.517446666	PTPN11	-1.566692265
DMXL2	1.517064463	ITK	-1.566656063
MRPL41	1.515986496	SEC23A	-1.566401892
KIAA0319L	1.514402619	LOC114117770	-1.565956808
LOC101110577	1.513818013	LOC101105614	-1.565834268
TUBB4B	1.513751575	DLC1	-1.565705511
RNASEH2C	1.512370513	CNTFR	-1.565475658
FEN1	1.51187732	ARAP3	-1.564332406
SIGMAR1	1.51140241	TMEM107	-1.563337733
STK11IP	1.510881705	IKZF1	-1.562754215
LOC101111670	1.510312193	STON1	-1.561922801
CCDC71L	1.510022079	GPR155	-1.561576776
LOC101121590	1.509438557	DENND1C	-1.561418606
PGAM1	1.509005711	ABL2	-1.561385458
PPP1R1A	1.508111683	CFL2	-1.560428873
LOC105607442	1.508049112	DACT3	-1.560396625

YIF1B	1.507499418	SLC45A4	-1.559642389
SIVA1	1.50664437	USP25	-1.559361809
LOC114116647	1.502140912	KIAA0754	-1.558522016
THAP4	1.502137396	PPL	-1.558393035
SLC25A1	1.500334354	SERINC4	-1.558001348
NCAPH2	1.500088239	LOC105606870	-1.556481104
EBF4	1.499270565	LOC114117316	-1.556233165
C5H19orf24	1.499026263	LOC105608665	-1.555548342
LOC114116394	1.498851024	LOC114117629	-1.555395173
TMEM11	1.498672066	TSPAN12	-1.555132067
WASHC1	1.498493479	LOC114116409	-1.554926381
SUV39H1	1.498464882	KIAA1328	-1.554322356
CMTM5	1.497833808	FNBP1	-1.553970742
RIMS1	1.497321935	ANKRD2	-1.553589055
LOC101103401	1.496319152	FIGN	-1.553575098
ACSM1	1.496319152	HIP1	-1.553356625
TMEM200A	1.496242591	WWTR1	-1.553286698
NOLC1	1.494938865	PARP8	-1.551770378
DEPDC7	1.494139439	IGSF6	-1.551754931
SPTBN4	1.492637666	NFATC2	-1.550029596
RCC2	1.492181111	CHRD	-1.549783316
NOL12	1.491745416	SMG1	-1.549063638
RNF126	1.491555869	EPDR1	-1.547162929
LOC101104501	1.490846316	LOC114118399	-1.546680881
TRIM28	1.488571521	LIX1L	-1.54654038
TMEM176A	1.48623758	RAB27B	-1.545965113
MOGS	1.485072153	MYH10	-1.545818749
HSPD1	1.484680186	NXPH4	-1.545806917
ABHD6	1.484489495	AXL	-1.545266647
RND1	1.483880014	KYNU	-1.544989466
LOC101109157	1.483491361	LOC105615181	-1.544989466
FMN1	1.483491361	ABTB2	-1.543771686
C17H12orf49	1.482305059	LOC114109073	-1.543219042
RBP1	1.481753214	NACC2	-1.542983394
TMCC2	1.481035742	LOC101118851	-1.542078281
LOC114109653	1.480140068	CSF1R	-1.54170935
LOC114115656	1.479340963	ADGRF5	-1.541035183
C3H12orf10	1.477815627	AKT3	-1.540169721
HDHD5	1.477402177	LOC105610817	-1.539981186
APOO	1.47709247	LOC101112306	-1.539836318
LOC114110277	1.476616291	TRPC4	-1.539759882
GPT2	1.474801667	REEP3	-1.539260534
CHKB	1.474479124	SFMBT2	-1.539165132
ALG12	1.474361583	SDC1	-1.538661039
KCNE5	1.472652445	ZNF521	-1.53857754

ZNF684	1.472652445	LOC114110610	-1.538280735
KIAA0319	1.472652445	KIRREL3	-1.538116579
ACSS1	1.471333795	BARX1	-1.538061096
ME1	1.47079268	TMEM88B	-1.53805065
HSP90AB1	1.470418137	ITPR1	-1.537017935
COQ4	1.468336852	SPIN4	-1.536881252
DGKQ	1.468107219	NT5DC3	-1.536569897
RASL10B	1.467823198	KIF5C	-1.536569897
LOC114115233	1.467593581	CEP85L	-1.535751203
CHCHD6	1.466409198	UBN2	-1.53418915
CSDC2	1.46636	LOC101111337	-1.533973131
LOC114110457	1.465086489	KCNE3	-1.533935492
LOC101122683	1.465086489	FGD6	-1.533036562
ZSWIM1	1.464592265	CERS6	-1.532374878
LYPD1	1.464008679	LOC114113062	-1.532335708
LOC114114607	1.463952018	LOC106991345	-1.531485563
TXNL4B	1.462947133	CARD10	-1.531106386
STMN3	1.46270667	LOC105613077	-1.530959648
LOC114111346	1.462396522	LOC114117881	-1.530942191
AEN	1.460645181	CD2	-1.530510419
LOC114116894	1.460133077	RASL11A	-1.530361922
RPL28	1.458680553	ZFP36	-1.530193513
CENPM	1.458510964	CYTH4	-1.529721224
GFRA3	1.458333777	MAP1B	-1.528693996
FASN	1.458318022	SPTBN1	-1.52864232
TCF19	1.457487724	LOC114108620	-1.528574258
CHCHD10	1.456002908	LOC114109697	-1.528500492
LOC101120481	1.45526801	AKAP11	-1.528376511
LOC101121595	1.454858227	ARHGEF37	-1.527755102
ARMC6	1.454774163	TCIM	-1.527738011
MANF	1.45415051	SPN	-1.527511498
POLR2E	1.45235773	C1QC	-1.527485957
MIS18A	1.451838621	GPR182	-1.527285212
LOC101112635	1.451712622	SEMA3B	-1.527258062
FAM136A	1.451333174	VWF	-1.526704227
ALKBH7	1.450154766	DTNA	-1.526135312
DEDD2	1.449766846	SH3RF3	-1.52495921
LOC101110918	1.44928131	OXR1	-1.524744647
SLC2A8	1.449199486	TRIM44	-1.524665642
FAM83D	1.449181913	FLNB	-1.523958036
CRB1	1.448971725	PDZRN3	-1.523955148
HSPA8	1.447967104	ASPN	-1.52385727
PLIN2	1.447199193	SMYD4	-1.523308505
LOC105609758	1.445982079	EPS15	-1.522971253
CLUH	1.445427655	UBL3	-1.522820154

TARBP2	1.444958222	BOD1L1	-1.522773436
SCD	1.444902113	UST	-1.522558086
LOC114118751	1.444683648	ZBTB18	-1.522220238
SPC24	1.444400058	KCTD10	-1.521795211
VILL	1.443779201	CCRL2	-1.521475933
LIMK1	1.443546875	LOC105611303	-1.520697151
HELLS	1.442569083	STAP1	-1.520697151
LOC106991919	1.442404225	MFSD6	-1.520265838
DIPK1B	1.442124097	DIP2B	-1.519515883
PEX11G	1.441853507	KIAA0355	-1.519044957
TFPT	1.441310153	PDLIM3	-1.518782314
LOC114109555	1.44019024	THAP5	-1.518338358
CPSF4	1.439423824	NKTR	-1.518229408
EIF3B	1.43791564	IL6ST	-1.516864191
MTLN	1.437337066	IL16	-1.516468582
MBD3	1.4369244	PTGER4	-1.515800483
LOC114114052	1.436845566	ZNF568	-1.515254528
CASKIN1	1.436845566	FEZ2	-1.514146572
APLN	1.43665817	ARL13B	-1.513820132
CNKSR1	1.436620068	EFR3A	-1.513436044
SDSL	1.436620068	GABPA	-1.513183542
TMUB1	1.436416385	FGD3	-1.511924712
TYMS	1.435157123	CPT1A	-1.51176881
WDR46	1.433949982	IL18	-1.51038712
LOC114116593	1.43389395	MYCT1	-1.51016678
DEFB112	1.433670645	ARMCX3	-1.509886837
TMEM51	1.433470214	RALGAPA1	-1.509883959
HTR7	1.433025571	GPR63	-1.509426151
WWOX	1.432935305	LOC114110253	-1.50831887
PISD	1.432620692	EBF1	-1.507737786
FKBP4	1.431469734	SECISBP2L	-1.507499324
TBRG4	1.431428378	CDS2	-1.507212004
LOC114116877	1.430991808	UHMK1	-1.50627816
ST6GALNAC4	1.430602469	LMOD3	-1.506164037
LMNB1	1.430498166	LOC105604204	-1.506164037
CDC34	1.430230704	VNN2	-1.505656171
PIGU	1.425030559	PIK3R5	-1.504785077
ZDHHC16	1.423766212	GPM6A	-1.503605631
RPL26L1	1.423290084	KLF10	-1.503129669
MRPL57	1.423278025	GGTA2P	-1.50195978
LOC101119989	1.422515423	MFAP3	-1.501673702
LOC114108828	1.42241435	IFT81	-1.50148633
TSPAN17	1.42193242	CYYR1	-1.501409183
CCDC58	1.421378276	LOC101107908	-1.501390214
EIF4A1	1.418035391	RAB27A	-1.500921865

GPAT3	1.417530291	LOC101113741	-1.500823279
RAB32	1.417264223	BTBD7	-1.500530658
WDR25	1.416733054	MFSD8	-1.500244419
LOC114117876	1.416316856	WSCD2	-1.500194338
RGS12	1.415827092	OGFRL1	-1.499648923
RFC4	1.415576157	HERC3	-1.499305486
NTF4	1.41509492	IL2RG	-1.498840701
LOC101121420	1.41505292	C13H20orf194	-1.497483357
SERGEF	1.413628282	HSD11B1	-1.49726605
AFG1L	1.411353138	ITSN1	-1.496549427
RRM2	1.411344957	RGS3	-1.495341125
GTF2IRD1	1.410129	JAK2	-1.494645604
LYRM9	1.409965727	LOC106991294	-1.49455494
XKR5	1.408523788	ELMO1	-1.494497099
NOXA1	1.408523788	COL4A1	-1.494265018
FUOM	1.407831979	CCDC88B	-1.492560946
CYGB	1.407352884	PIK3CD	-1.492470019
BRAT1	1.407293415	PIP4K2A	-1.492237871
ARMH4	1.403832403	PAPPA	-1.491617235
IRS1	1.402948496	PPP1R9A	-1.49134351
LOC114115368	1.402720981	TMOD2	-1.490437772
AGAP3	1.402617762	GXYLT1	-1.489804806
TFCP2L1	1.402604368	LOC101109746	-1.489202851
CD3EAP	1.402037406	APPL2	-1.488475653
SLC35B1	1.401683412	SYNJ1	-1.487875731
LOC101103001	1.400986759	CHD5	-1.487622921
GET4	1.40053461	PLXDC2	-1.48760869
ANAPC5	1.400289874	CTSK	-1.486847284
ZNF593	1.397864779	PPM1L	-1.486677457
DNMT3B	1.397057395	HIVEP3	-1.486065975
RRS1	1.397042732	LRRC8C	-1.485582713
SPEF1	1.396253566	LONRF3	-1.484580973
TMEM161A	1.396174843	CD55	-1.483954465
BRI3BP	1.396155736	IL13RA2	-1.483936623
ARL6IP4	1.395932151	ITPRIPL2	-1.483033917
LRP3	1.395577832	CYS1	-1.482963304
AIMP2	1.395519569	MASP2	-1.482935573
KIF19	1.394877562	LOC105606943	-1.482302376
RAD54L	1.39391094	CDH5	-1.481943921
FAM162A	1.393494238	LOC105603399	-1.481729178
RAPSN	1.393323	HTR6	-1.480835978
ZNF358	1.392949723	MAP4K3	-1.48082762
NDUFAF8	1.392912851	PAM	-1.480757141
CCNJL	1.392540364	SLC35D2	-1.480425935
CLEC1A	1.392443512	LOC105605886	-1.4787899

CELA3B	1.391715658	LBH	-1.478678553
LOC114114536	1.391692645	TM6SF1	-1.477933632
CCNE1	1.391148581	CILP2	-1.477837759
SREBF1	1.390520299	TMEM232	-1.477837759
ANAPC2	1.390503691	NFKBIZ	-1.477001229
METTL1	1.39042987	EDN1	-1.476910107
TMEM185A	1.389999704	PLEKHA3	-1.476837398
LOC114112992	1.389784181	SVIL	-1.476730008
LOC101120681	1.389430888	MMP17	-1.476410327
TFAP4	1.389381664	RASSF8	-1.475781545
FAAP100	1.389201547	GRK3	-1.474187062
MTHFD1	1.388114763	FKBP14	-1.473784774
LOC105611292	1.387537909	LOC105615677	-1.472382315
PGD	1.386508818	PLEKHG1	-1.472294111
LOC114116609	1.385242368	NRCAM	-1.471997018
LOC114111456	1.385242368	KIAA1755	-1.470739598
GRIP1	1.382967363	SNX16	-1.470648602
SPIRE2	1.382942186	CEP170	-1.469976687
LOC114110676	1.380275761	PTAR1	-1.469774489
FARSA	1.380180245	RHOBTB3	-1.468968767
MRPL2	1.380091208	ZBTB7C	-1.468477002
ZNF462	1.378472068	MAP4K5	-1.465959589
RCC1	1.378404577	PTPN14	-1.465891743
HERPUD1	1.378339233	AGTPBP1	-1.465681024
TMEM163	1.378097852	TNFAIP8	-1.464964615
TEKT2	1.376196156	FRRS1L	-1.464886573
SLC52A2	1.375869192	JADE1	-1.464792119
TIMM17A	1.374890833	RBPJ	-1.463794617
LOC101104866	1.373660692	LOC105616742	-1.463744418
CCDC167	1.373073211	NEURL3	-1.46336826
NIPSNAP1	1.373012522	LOC106991947	-1.46336826
CREG2	1.371766144	ABCB1	-1.46336207
SLC12A7	1.370980906	GPBAR1	-1.462881785
HDGF	1.370445984	XYLB	-1.462561885
POC1A	1.370416465	NLGN3	-1.459833881
TSFM	1.370386551	KCNJ2	-1.459487708
MAD2L1	1.369406653	GBP6	-1.458862157
LOC114113987	1.368994717	PPP1R12B	-1.458531267
HEXIM2	1.367697074	PCDH1	-1.458530728
CEBPZOS	1.367315834	LOC114116350	-1.458128385
MANEAL	1.367259196	LPGAT1	-1.457936327
SNAP29	1.366775027	WASHC4	-1.457422775
UFSP2	1.366162988	FER	-1.456105523
LOC106991494	1.366156192	LOC114115243	-1.455847074
LOC114118295	1.364457201	TSPAN18	-1.455154422

ALDH1L1	1.364259451	LGI4	-1.454419214
LOC114109439	1.363759433	MOG	-1.453667078
MRPS34	1.362942745	TBC1D12	-1.452423051
ERFE	1.362626684	IQGAP1	-1.452296545
MTX1	1.361772817	FERMT2	-1.452225306
SNX8	1.360907898	ABI1	-1.452007747
ING1	1.359901406	JAM2	-1.451504978
DTNB	1.359044797	SREK1IP1	-1.451267589
MED12L	1.35904135	RC3H1	-1.451197314
HYLS1	1.358889579	PLIN4	-1.449067576
LOC114111250	1.35860639	SSPN	-1.447688102
CHST15	1.357864482	TRIM2	-1.446780618
LOC114118070	1.3555698	FLNA	-1.446459167
SHANK1	1.355026111	ASIC4	-1.443718047
NAT10	1.35493692	RP2	-1.443332477
DCP1B	1.354699601	LOC114113883	-1.442846587
FAM133A	1.354059594	LOC105608011	-1.442237259
OGFOD3	1.353965103	GCA	-1.441971834
NCDN	1.353509438	TRAPPC8	-1.441938064
THYN1	1.353063415	ATP9A	-1.441874281
MPIG6B	1.352547359	C25H1orf198	-1.44169918
SPIB	1.352547359	ZC2HC1A	-1.441049779
JAGN1	1.351798373	LOC101113619	-1.440858228
STOML1	1.351501751	GPR1	-1.440858228
TOMM34	1.349798688	LOC114110278	-1.440858228
ATP2A1	1.349538962	LOC114110418	-1.440322727
KRT222	1.349538962	LOC105607533	-1.440322727
ANKS3	1.349018551	TBC1D8B	-1.439536155
TMEM88	1.347701534	C1R	-1.43913567
SPOCK1	1.346463155	ANKRD33	-1.438795156
C14H19orf33	1.345201796	LOC114113232	-1.438648762
MRPL24	1.345106143	FUCA2	-1.438315569
PRMT6	1.34489915	FAM126A	-1.438242974
LOC101116687	1.344675886	TENM2	-1.436783949
LY6E	1.344656759	IRF4	-1.436054562
DRC3	1.343005473	LIF	-1.435512369
LOC114109632	1.342918063	LOC105610169	-1.434140776
MARK1	1.342514376	ELK3	-1.434054662
HMGA1	1.342187457	SPATA6	-1.433926027
ABHD17A	1.341630023	LOC101120999	-1.433666019
POP5	1.341149707	NETO1	-1.433666019
LOC101111215	1.340252924	ZBTB16	-1.433484791
PTTG1	1.339831475	GAB3	-1.432962438
INTS1	1.339452572	KDM1B	-1.432651597
TMEM98	1.33760032	PODXL	-1.432577881

TSPAN5	1.336527853	MORC3	-1.431319897
PSMB1	1.336520731	MYADM	-1.431266102
TFB1M	1.336065745	RTN4RL1	-1.431224455
MFSD3	1.3360004	GPCPD1	-1.430879766
SLC26A6	1.335184312	HIGD1B	-1.430411056
GEMIN8	1.335136722	KIF3A	-1.42961296
NUDT1	1.334669928	SPAG1	-1.428737432
ARHGEF28	1.334451482	TM4SF1	-1.42856047
ZNF511	1.33423443	NCKAP1L	-1.427845909
PRELID1	1.333273186	GLUD1	-1.42724975
DDX56	1.332898784	PHACTR1	-1.426323769
LOC105602911	1.332809948	RAB29	-1.426013637
CENPP	1.33280939	NKG7	-1.425690331
RAPGEF4	1.332660884	HCFC2	-1.425214022
SLC22A23	1.332321283	ANGPTL2	-1.42490725
CCHCR1	1.332249526	YAP1	-1.424847917
SUPT3H	1.331871783	ASPH	-1.424624136
KNTC1	1.331782011	ABHD5	-1.423470307
BCKDHB	1.331220529	NOTCH1	-1.423347885
NXPE4	1.331141712	UNC5D	-1.42284983
CHTOP	1.330990148	ST7	-1.422692436
C12H1orf112	1.330924764	LOC105603415	-1.422164198
TSEN34	1.330694197	BCAM	-1.422123367
MRPL36	1.330649072	NRROS	-1.420276377
FAM184A	1.33043277	TCF7L1	-1.420101913
CDK5R1	1.32961362	CRPPA	-1.41984964
ADCK2	1.329146954	SEL1L	-1.4198312
IGSF3	1.328683108	PBXIP1	-1.419617284
DRG2	1.327053208	SFT2D2	-1.419489689
WDR31	1.32675485	ASH1L	-1.419314561
AAAS	1.326382159	COLEC12	-1.419049567
PSMB2	1.326188705	IL10RA	-1.418807972
MRM1	1.32592048	HIF3A	-1.418505133
FAM110A	1.325152511	TLR2	-1.418478908
LOC114114048	1.323051722	LOC101109728	-1.418081313
FARS2	1.322949331	SPATA13	-1.417633652
RFXANK	1.322835543	SERPING1	-1.417410741
GSR	1.322827565	MACF1	-1.416370073
LOC114113841	1.322778065	C26H4orf47	-1.416307676
PAXIP1	1.322583221	GSTP1	-1.4159631
LHCGR	1.322233976	OSBPL8	-1.415896061
CEP83	1.321803941	SRGAP3	-1.41421681
LOC114118293	1.321641183	LOC105607146	-1.413565974
ADRM1	1.321583384	LOC105612882	-1.413005361
LOC101117650	1.321355849	HELB	-1.412634312

AJAP1	1.321197044	GSKIP	-1.412124209
PIR	1.320936179	BLNK	-1.411443366
DNAJC9	1.319905394	SH2D1A	-1.411340877
NELFE	1.319544554	LOC101115486	-1.410972586
TEAD2	1.319322605	PLXNA2	-1.410948936
CENPU	1.319236677	LOC101111006	-1.409674389
STXBP2	1.319007118	KLHL38	-1.409278995
STXBP5L	1.318240648	MAN1A2	-1.409074246
ALPK2	1.317652501	LOC101108528	-1.408890587
TMEM82	1.316243267	GRIN2D	-1.408527984
RNASEH1	1.315815787	DNAJB14	-1.408375334
SPHK2	1.315199322	ACVR1	-1.40734036
LOC101113001	1.314375522	DQB	-1.407309179
RIOX2	1.314148046	PRICKLE1	-1.406788817
CLBA1	1.313180968	SDC2	-1.404984021
LOC101120607	1.313154606	UTP15	-1.404898977
BRMS1	1.313112976	GCHFR	-1.404841374
GABRB3	1.312669767	LOC105611708	-1.404407841
FBXO24	1.312669767	TEAD1	-1.404383241
THEM6	1.311551207	ACAD11	-1.404292364
ECSIT	1.311426958	CASP8	-1.404251966
LOC114112974	1.31133962	PARVG	-1.403995875
POLRMT	1.310898349	LOC101108647	-1.403878767
SIPA1L2	1.309380159	PID1	-1.40290455
ZNF274	1.309008697	TMEM123	-1.402608495
LOC114112520	1.308783682	ZKSCAN8	-1.401794442
LOC101115252	1.308578299	TLR4	-1.401641107
FUS	1.308527214	LOC105613248	-1.401157223
LOC114114506	1.308321192	PDE9A	-1.40067674
ATP13A1	1.308116503	NUBPL	-1.399873551
HAAO	1.307789739	LOC114112896	-1.399679354
RBM19	1.30775192	PIKFYVE	-1.399661046
TMEM259	1.307640919	ADA2	-1.399491755
ETHE1	1.307220937	LOC105608222	-1.39842207
TNNI2	1.307202712	SUSD6	-1.39823293
ELOF1	1.307083679	RFK	-1.398134776
PHB	1.306043683	PPP3CA	-1.397319194
JAK3	1.305893913	S1PR1	-1.39720648
GMNN	1.305797112	HNMT	-1.396930711
GNAZ	1.305689533	CD47	-1.396472619
LOC101123112	1.305335984	SOX18	-1.396322062
MRPL20	1.305242586	SACS	-1.396304572
ZNF579	1.305025075	LOC101122398	-1.396205871
PSMD3	1.304793966	TNFSF9	-1.393968934
KCNIP4	1.304721973	LOC101102480	-1.393625509

DDX54	1.30463605	LOC101116121	-1.392922015
BYSL	1.304383923	F11R	-1.392399566
SLC43A3	1.303271986	ATG2B	-1.392006782
SMOX	1.302833475	SPTLC2	-1.391669315
EMC9	1.302339924	NIPAL3	-1.391570058
LOC105606907	1.302332981	GIMAP6	-1.391425033
FRAT2	1.301864808	PCSK6	-1.391256328
COL6A5	1.301604736	MEF2C	-1.390922284
PYCR3	1.301119917	FBXO30	-1.390053202
PTK2B	1.300911449	BTBD19	-1.390053202
DNLZ	1.300703503	NFIB	-1.389712485
MDH2	1.30056764	IL13RA1	-1.388786646
LOC114114855	1.300285682	SLC2A4	-1.387932514
LOC101109915	1.300285682	DOCK2	-1.387800151
LOC105603102	1.299267707	SIPA1L1	-1.387700693
SLC7A5	1.298495844	TSPAN9	-1.387298197
SDHB	1.298201275	MBTD1	-1.385212942
WDR74	1.297918068	ADAMTS8	-1.385106902
USP35	1.297630835	AGRN	-1.384818248
FAH	1.297308341	C4H7orf57	-1.384682409
PRR36	1.297140284	LOC105610483	-1.384655567
LRRC75A	1.296707972	RCOR3	-1.383712462
NSDHL	1.296577307	LY96	-1.3831015
ASMTL	1.2960001	LOC101108171	-1.382820727
LOC114118084	1.295952184	ECSCR	-1.382799913
SNRPD3	1.295227002	GMFG	-1.382604971
ADAMTS14	1.295196944	CCDC69	-1.382466313
ANKRD45	1.293779597	RAB20	-1.382459327
SYNGR1	1.292852639	TUBB4A	-1.381864659
CLPTM1L	1.292478026	LOC105605978	-1.381864659
HDDC2	1.292343198	COL4A2	-1.381854289
CCDC86	1.292177238	LOC101122545	-1.381675288
GRPEL1	1.291451547	ZHX1	-1.38105855
HHAT	1.290840658	SMIM13	-1.380905957
TMED1	1.290754751	FNDC3A	-1.380565493
NT5E	1.290677709	GNB3	-1.380505501
EPCAM	1.28877601	LOC114109030	-1.380351336
TIMM44	1.286619907	CDC42EP3	-1.38021411
IL1A	1.28536588	DUSP8	-1.380139954
TSTA3	1.285207487	LHFPL6	-1.379237889
RPL7A	1.285149739	SGCE	-1.378883117
CENPA	1.284931633	STARD13	-1.378104034
ZNHIT2	1.28484123	TANC1	-1.378022974
NT5C3B	1.284724608	AQP1	-1.377879
PUS1	1.284688217	SLC40A1	-1.377767806

ZNF428	1.284670368	RBL2	-1.377565205
TIMM22	1.284600602	ELMSAN1	-1.377404996
GAK	1.284374436	PTER	-1.377155727
DET1	1.283608022	SEPT7	-1.377054555
HMGN2	1.283606676	LOC114114916	-1.376464328
LOC114114556	1.283410939	SERINC1	-1.376353282
TBL3	1.28276013	MAN2A1	-1.374821176
LOC105612442	1.281851415	SLC9B2	-1.374728012
LOC105612761	1.281851415	DENND5B	-1.374716405
APOE	1.279977867	ST6GALNAC3	-1.374474608
MRPL30	1.279850518	LOC114116073	-1.373450183
IGSF9	1.279519474	CLK4	-1.373098101
LOC106990971	1.279519474	SLC36A4	-1.372632425
LOC114109706	1.279519474	MYSM1	-1.372492501
LOC114118007	1.279519474	PDZD7	-1.371498282
IGLON5	1.279446972	WAS	-1.371304211
C1H3orf33	1.279241963	PTPN6	-1.371000624
PKDCC	1.278209173	GUCY1B1	-1.370657383
IQCN	1.277809377	RRAS	-1.370563427
NMNAT3	1.277410322	GLI3	-1.368697205
LOC105610456	1.277232426	MRAP2	-1.367747753
SPG7	1.27678466	PPP2R2C	-1.3673981
TRMT2A	1.275013185	LOC101115646	-1.367070278
OSCP1	1.274762693	SLAMF6	-1.367012594
NDUFAF3	1.274395709	LRP4	-1.366824876
PTRH2	1.274109249	SHROOM4	-1.366576097
POLR3H	1.273795391	TMEM65	-1.366510067
NDP	1.27352672	PPP1R3D	-1.366482215
RPL22L1	1.273499311	SLCO2B1	-1.365975122
LOC105605360	1.272546133	DDHD2	-1.365431328
PCCB	1.272034309	LOC105611269	-1.365376367
LOC114117998	1.271846771	SPDL1	-1.365105057
CROCC	1.271189446	ARRDC5	-1.362921893
KLHDC9	1.270187216	ASAP2	-1.362682839
ERCC2	1.270106504	NR3C1	-1.362661128
SOWAHB	1.269739771	CD247	-1.362570763
MAN1B1	1.268759041	STXBP4	-1.362482896
LOC106990580	1.267996566	ANXA5	-1.362386738
SMARCB1	1.267909103	ARL4C	-1.362363229
REX1BD	1.267525184	LOC114113049	-1.362363229
SLC26A11	1.267395645	SLC34A3	-1.362363229
EPOP	1.26716218	LOC105616457	-1.361827055
MYO10	1.26714014	TSHZ3	-1.36163462
DND1	1.267096864	TTC26	-1.360638793
C5H5orf63	1.265943694	PTP4A3	-1.36054488

ZSCAN2	1.265848865	TYROBP	-1.359719752
PNCK	1.265495423	SMAD1	-1.359574525
CCNE2	1.265068822	VAMP7	-1.359131336
FAM173A	1.265013568	ADAMTS17	-1.358656847
CIAO3	1.264724604	MAPK8	-1.358100353
RNPEP	1.264329382	TMEM52	-1.35805294
TRMT112	1.264067012	LOC101104528	-1.358051659
LOC105606974	1.263813353	SFXN3	-1.357941214
SLC25A22	1.263587172	SHOC2	-1.357079012
LOC114117236	1.263123486	TENT4B	-1.356966006
NAGPA	1.262965617	PUS7L	-1.356822437
DUSP15	1.262638014	GDF11	-1.356137101
LOC101115315	1.262561337	FUT8	-1.355636721
ZXDC	1.262457882	RAC2	-1.355333367
RRP12	1.260742165	HEBP2	-1.35520117
LOC101110181	1.260522228	ZFYVE16	-1.353574142
MMP25	1.260522228	LOC101102857	-1.352913589
SLX1A	1.260488704	TRIM45	-1.352607144
CHEK1	1.2602242	CD99	-1.352604114
CALR	1.259565761	PRKAA1	-1.352289585
WDR54	1.258776982	SYT1	-1.352273443
DTX2	1.258529652	ACKR3	-1.351872011
MRPL37	1.258520646	LOC114116116	-1.351053005
PNMA1	1.258036594	RAB33B	-1.350795456
HSD3B1	1.257891564	HPCAL4	-1.350676014
MGAT3	1.257549723	LOC105604257	-1.349268128
PRR3	1.257539397	PPP1R3E	-1.348716514
NT5M	1.256556523	LOC114110986	-1.348716514
MMACHC	1.256428321	LOC105610030	-1.348716514
LOC105607964	1.255986503	IRX3	-1.34861367
SLC29A2	1.255428334	AIF1	-1.348182697
DLGAP5	1.254929238	CDADC1	-1.347998458
POP7	1.254181271	SMPDL3A	-1.347982907
AK2	1.253873334	THAP6	-1.347945088
PSMC1	1.253806376	ASAP3	-1.347475761
LOC101115461	1.253646778	VDR	-1.347403479
LOC101118248	1.253580237	UGP2	-1.347322174
ZNF524	1.252805674	LOC101111694	-1.347322068
HSPA6	1.252801792	MEGF9	-1.347051111
ABCB8	1.252434446	CCL19	-1.346789769
CACYBP	1.25211481	SUGCT	-1.346487107
HSD17B10	1.251426109	TMF1	-1.345658248
SRSF7	1.251151978	ARL16	-1.345074947
LOC114116050	1.251018792	ABLIM1	-1.345037117
SNRPB	1.250801035	GOPC	-1.34488135

DDX4	1.250385183	KRT17	-1.344869077
CABP1	1.250330989	LOC105613434	-1.344869077
EIF3I	1.249928351	AHNAK	-1.344826214
FBXO10	1.249406439	ZDHHC2	-1.344529725
PPP1R14B	1.249204043	PHKG1	-1.344044946
LOC101117851	1.249062893	KAT2B	-1.343689365
RALYL	1.248506926	INO80D	-1.343071996
LPCAT3	1.248074061	SNAP23	-1.342259406
NR2C2AP	1.247885574	GM2A	-1.342242943
GMPPB	1.247684036	SMIM17	-1.341608461
SLC26A2	1.247439467	NPC2	-1.341251829
ZNF18	1.246781815	DDX6	-1.340897595
MSH5	1.246059434	SH3PXD2A	-1.340456374
SDCBP2	1.246059434	RNF43	-1.339997556
LOC114118455	1.246059434	DDX3X	-1.339955135
LOC101120512	1.246059434	KIAA1958	-1.339612945
LIPT2	1.245005262	BDP1	-1.33870735
B3GNTL1	1.244841612	TBCEL	-1.337916692
LOC101109919	1.244841612	MYH15	-1.337316977
TRAPPC12	1.243737888	ATG4A	-1.337034896
JUP	1.243502837	LOC114114809	-1.336730967
LOC101111154	1.243463475	LOC101120489	-1.336730967
GATD1	1.242915127	LOC106990463	-1.336730967
LOC101115658	1.242732253	PARP4	-1.336483009
MRPL46	1.242694431	MSN	-1.335649092
DHPS	1.241893853	GPR132	-1.334544395
SPAG5	1.241877361	CSF3R	-1.334388354
ARHGDIG	1.241461455	PLEC	-1.334205692
OXT	1.240844116	ADD3	-1.334058261
LOC114112963	1.240429563	PCYT1B	-1.334036997
NEIL2	1.240337608	NRG1	-1.333704393
NCAPG2	1.240329615	CRISPLD1	-1.333178582
SYT9	1.23985389	CSRP1	-1.332701363
LOC105616153	1.239488519	PDE4A	-1.332427075
EIF5B	1.239353202	ZDHHC17	-1.332390153
RGS16	1.237610212	LOC114117015	-1.332203168
NDUFV1	1.237429663	KCND2	-1.332203168
TXNRD2	1.237423571	RB1	-1.331779347
THOC3	1.236627964	LOC101106384	-1.331494198
STK36	1.236604523	LOC114118863	-1.331252687
GPAT4	1.236463777	ANKRD12	-1.330687159
LOC114116379	1.236003425	ITGB7	-1.329961634
FAM78A	1.235855296	NPR3	-1.329523665
LOC114112817	1.235555086	CCDC73	-1.328622195
DMAP1	1.23446926	C14H16orf87	-1.328573953

ZNF444	1.234065742	ITFG1	-1.328057501
SLC22A17	1.233074187	LOC114115898	-1.327313381
CORO6	1.232824086	C5H5orf24	-1.32726373
ACD	1.231839852	FAM177A1	-1.327125646
TREX1	1.231682523	DPP8	-1.326431219
CAPN10	1.231202679	CRACR2B	-1.326198327
NDUFAB1	1.23103499	ADGRL4	-1.326071998
PEX14	1.229867088	FAT4	-1.325336588
DHRS7B	1.229642341	MYBPC2	-1.3251972
PCLAF	1.229507756	PICALM	-1.323928052
TRAPPC9	1.229447211	CPD	-1.323418295
ACADS	1.229104156	PAPLN	-1.323365743
LOC101117015	1.228783379	UBR2	-1.323275371
MRPL14	1.228692093	VCAN	-1.322622568
CA8	1.227777961	TNNT3	-1.322608711
PHC1	1.226673128	MIB1	-1.322302276
LOC101116812	1.226625737	ZBTB47	-1.322056944
PDE8B	1.226086251	NET1	-1.322031407
GPRASP1	1.225493034	TRPM7	-1.321776792
DDX28	1.225280531	LCK	-1.321714652
BLVRA	1.225126295	SENP7	-1.32099391
PNPLA6	1.224331143	IKZF2	-1.320810897
PSMB5	1.224137079	LOC101122953	-1.320527577
CDK4	1.223947279	LOC101102001	-1.320246156
LOC114110249	1.222429565	MN1	-1.320238367
NR1H3	1.222129869	NMNAT2	-1.319610783
LOC101117527	1.222019002	LOC114108840	-1.318293846
SERHL2	1.222014551	ZC3H6	-1.317953515
GRB14	1.221687202	PTPRU	-1.317948689
MUS81	1.221545426	LOC114117878	-1.317705935
SPNS1	1.220694454	MOSMO_2	-1.317381131
TMEM129	1.219761651	SMURF1	-1.317062351
SFT2D3	1.218104895	CPNE1	-1.317000883
CFAP410	1.217912063	NECTIN4	-1.316354527
SMPD2	1.217896181	EFNA1	-1.316328917
NLE1	1.21755414	ZNF41	-1.31618793
MIPEP	1.216758028	LOC114113014	-1.31581189
HIP1R	1.216696032	CRACR2A	-1.315089138
KPNA2	1.216471524	SCUBE2	-1.314900877
NCLN	1.215632609	PHACTR4	-1.314883624
NUBP2	1.214832861	GPC3	-1.314018739
MRPS16	1.21448752	ACSS2	-1.313535499
NEDD9	1.21377578	KLF3	-1.313421049
RGS2	1.213684881	ABHD15	-1.312566362
SAE1	1.213471493	LOC105609211	-1.312451593

FREM2	1.213463143	SLC8A1	-1.312331358
PPP3CC	1.213354757	HERPUD2	-1.312090842
ATP7B	1.213329156	TOB2	-1.311103209
RAVER1	1.213135792	GLIS3	-1.310207863
MFSD13A	1.212894651	COPZ2	-1.310075655
NQO2	1.211782393	LOC114111751	-1.310054196
INTS9	1.211524257	CFB	-1.309248281
ACTN2	1.210675582	KIAA1147	-1.308968658
LOC114115023	1.210405771	KCNA3	-1.307705313
REPIN1	1.210291641	AFF1	-1.307674152
ZNF575	1.210152092	LOC114117229	-1.307513959
HEMK1	1.209774735	TC2N	-1.306813196
LOC106990145	1.209660918	LOC114118362	-1.306123913
STK25	1.208554516	CCNT2	-1.305956113
RPUSD1	1.208521118	PLSCR4	-1.30557409
RASD1	1.207469772	RAB8B	-1.305537055
TSSK2	1.206169143	LOC101122274	-1.303755423
LOC114113004	1.206092476	NTM	-1.30296781
DGCR6L	1.206003438	IQGAP3	-1.302933973
GREB1	1.205909426	TNFAIP8L2	-1.302007876
SLC37A1	1.205403787	RC3H2	-1.301076397
HSF1	1.204969129	HID1	-1.300812326
HTATIP2	1.20341376	GALNT18	-1.300086568
POLR2I	1.203231027	LOC114113926	-1.299956101
GSTCD	1.202263509	PPP1R3C	-1.299622478
OSGIN2	1.202246588	LOC114112164	-1.298997904
PNKP	1.201386576	INPP5F	-1.298304669
PSMA7	1.201238831	KIAA1217	-1.298135387
LOC105604472	1.20054766	LOC105603050	-1.297754954
GRINA	1.200220056	VGLL2	-1.296993107
COLGALT2	1.200123281	CATSPERG	-1.296361991
INSIG1	1.199951711	OSTM1	-1.296276367
ZNF169	1.199907933	FAM81A	-1.295861378
CLP1	1.199897216	AGA	-1.295721171
NDUFS4	1.199892573	PCMTD2	-1.295055266
TIAM1	1.199877877	IGFALS	-1.294479366
LOC101114597	1.199205354	ATL2	-1.294320895
NOP58	1.198408097	TXNDC16	-1.293541237
LOC114112251	1.198203617	GNG2	-1.293509737
UFSP1	1.198013162	SPTY2D1	-1.293229414
LOC101102276	1.197904418	MORN3	-1.29311771
TRAPPC3L	1.19771087	STXBP6	-1.290716676
MIGA2	1.197520904	CSF2RB	-1.290483139
LOC114117231	1.197473371	UVRAG	-1.290454491
CENPO	1.19536395	ARMC4	-1.290081936

SLC27A2	1.19477125	NCOA3	-1.289904751
LOC105607745	1.19477125	ATRNL1	-1.289895209
LOC101117055	1.194396828	PLPP6	-1.289497277
LOC114110140	1.19427677	MBLAC2	-1.289225318
PFAS	1.193824562	WWC2	-1.28918357
CINP	1.193439325	CABP7	-1.289182227
NSUN5	1.192972197	LOC101120749	-1.289182227
PPP6R2	1.192663446	VCPIP1	-1.289099681
FBXW9	1.192606731	ATP13A3	-1.288402204
LOC101117112	1.192473248	MXRA7	-1.288025491
NUBP1	1.192280581	MTM1	-1.287137923
DGAT1	1.192234848	LOC106990836	-1.287137923
GTPBP6	1.192185521	UNC5B	-1.287081853
SMPD4	1.191868624	ANK3	-1.286779246
CPSF1	1.191773536	LOC105606379	-1.286609499
LOC114113967	1.191362636	ARHGEF12	-1.286047822
LOC114116832	1.190778245	ILDR2	-1.285919987
SDC4	1.190367968	TM7SF3	-1.284858858
GEMIN5	1.189498732	KLF6	-1.284728555
CACFD1	1.189148394	LRRIQ3	-1.284704248
EMC8	1.189074272	TTC39A	-1.283465287
DKC1	1.189058988	LOC114111231	-1.283249918
NUP93	1.188976503	JMJD1C	-1.283033752
PRR7	1.187348676	SOX13	-1.28215604
PACS1	1.1871694	SEC22C	-1.282109581
SDHAF1	1.186146242	C1QTNF4	-1.282043442
PAOX	1.186002359	SAMD11	-1.281986184
LOC105604385	1.185844915	AHNAK2	-1.281934374
SNAPC2	1.185733598	TRPC1	-1.281905016
E2F7	1.185219398	LOC114108671	-1.280958901
LOC114110455	1.184245676	WDR97	-1.279523775
CLMP	1.183269137	C1QTNF3	-1.279141976
FRMD1	1.183198597	LOC101109111	-1.27899101
LOC101118761	1.183012655	NCOA2	-1.278616324
MPG	1.182406778	DIPK2B	-1.278457041
CCDC130	1.181501177	MRTFB	-1.27840893
LOC105612199	1.181283045	IQCK	-1.278007163
LOC114115629	1.181134971	ADAT1	-1.277925601
LOC105608442	1.180261541	SLC35D1	-1.277726727
EBNA1BP2	1.179783116	LOC101117622	-1.27739013
RPP40	1.179404927	RBMS3	-1.27712568
TIMM8A	1.179052393	USP32	-1.276554491
TIMM29	1.178383958	STX7	-1.275906175
LOC114113985	1.178257825	LOC101117013	-1.275800609
PHPT1	1.177681199	LOC114108651	-1.275800609

SLC30A3	1.177488043	WDR44	-1.27559399
AURKAIP1	1.176389433	KATNAL1	-1.274929982
LOC114113988	1.176230728	TIPRL	-1.273607775
LOC105615359	1.176230728	CDC14B	-1.273379787
DNASE1	1.1759932	ACRBP	-1.272995641
NR6A1	1.175303883	FKBP9	-1.272009607
ARHGAP33	1.17524892	ATRX	-1.270562736
LOC105603374	1.174971425	FZD1	-1.270523271
MCM10	1.17478449	FGR	-1.270474675
C12H1orf74	1.173924514	COL17A1	-1.269976644
MAGOHB	1.173447455	LOC114117254	-1.269665366
LOC114116198	1.173433773	SNPH	-1.268884201
ENKD1	1.173211624	LOC114118024	-1.26886164
LOC114113056	1.17320679	SNTB2	-1.268673537
ZNF628	1.172328986	LOC101114579	-1.267680857
POMGNT1	1.172267503	LOC114112017	-1.267610459
LMF1	1.172029503	FGFR2	-1.267588464
LOC101106288	1.170685133	ZFHX3	-1.267213602
NUDT2	1.169617	TACC2	-1.266875285
EFTUD2	1.16879148	COG3	-1.266141106
SPATA2L	1.168727258	LOC101104372	-1.265877035
LOC105611012	1.167802717	PRKCE	-1.264737318
FAM49A	1.167707887	ATXN7	-1.264653378
LOC101115345	1.166047684	HAVCR2	-1.264250744
LOC101118849	1.165916069	VASH1	-1.263843099
KIAA0895L	1.165530951	LOC114109071	-1.26110755
DCAKD	1.165411827	RCN1	-1.261067299
TMEM250	1.164931952	LRIF1	-1.260602017
RPUSD2	1.164225293	TRANK1	-1.260279104
CLASRP	1.164020787	FBXO8	-1.260125226
DAD1	1.163571916	LOC114110307	-1.259806866
DOK6	1.163354051	GPR153	-1.25962157
HSPA5	1.162375101	BOC	-1.259500115
LOC105603395	1.16203227	SERINC3	-1.259360672
MINDY1	1.161902389	LOC106990930	-1.259182387
ATXN7L2	1.160939948	MTURN	-1.258267657
PIDD1	1.160772383	MCUR1	-1.25769979
FAM71E1	1.15881424	CIITA	-1.257531651
LOC114116222	1.15881424	TRAPPC6B	-1.256583351
KIAA0895	1.15881424	NEK6	-1.256198195
SOCS1	1.158259037	RASSF5	-1.256097956
CASP3	1.158148371	S1PR4	-1.255652416
FAHD1	1.157206398	GPR183	-1.254657259
GTF3A	1.157015337	LIN7C	-1.253175499
ZNF777	1.156939742	GSAP	-1.251749375

STIP1	1.156171437	ETV6	-1.251534217
LOC114114089	1.155714233	LOC106991500	-1.25152498
LOC114116131	1.155324972	LOC106990188	-1.250838876
LOC101110178	1.154549309	GNA15	-1.250838876
IMPDH1	1.154262589	EMCN	-1.250235633
TCP11	1.15425282	ESYT2	-1.249969333
SEC11A	1.154166934	TMEM200B	-1.249692324
VARS2	1.153566523	LNPK	-1.248658216
TIMM10	1.152750414	LOC105612390	-1.248633684
B3GAT3	1.152648998	SLIT2	-1.248194169
METTL22	1.152378333	LRG1	-1.247534275
LSM6	1.152106378	GAL3ST4	-1.247382177
NYAP1	1.151904755	LTB	-1.246310301
LOC106990378	1.151753515	LOC114114820	-1.245706625
LOC114113921	1.151044437	ZC3H12D	-1.245706625
BOP1	1.149923909	ANKRD61	-1.245706625
DTNBP1	1.14907637	IRS4	-1.245706625
ZNHIT3	1.148951664	EPN2	-1.245630162
LOC101115554	1.148624911	SRPX2	-1.245413637
SLC25A6	1.1482881	SUFU	-1.245219676
FAM72A	1.14814838	HERC5	-1.244694823
PITX1	1.148115113	PGGHG	-1.243903649
DEAF1	1.147780243	FAM43A	-1.242610986
ACKR1	1.147718019	ITGA8	-1.241840948
OSBPL10	1.147026641	LOC101116622	-1.241517037
RPA3	1.146253716	EPHA1	-1.240237635
MMP9	1.145173522	TPM1	-1.239849658
OGG1	1.143323898	MOB1B	-1.239767403
HMGB2	1.142827372	BCL6	-1.239669565
SPP1_1	1.142579506	SYTL4	-1.23885014
BID	1.141946143	TIMP3	-1.238310102
RBM42	1.141560326	ITGAL	-1.237546425
LOC114110148	1.14103376	ITGB8	-1.237540558
PDP2	1.140471189	C7H15orf48	-1.237540558
PES1	1.140461957	LOC105614551	-1.23736668
MAPKAPK3	1.138195557	HOXA9	-1.237208651
TSHZ2	1.137126485	EVI2A	-1.236685899
RORC	1.137070581	TCAF2	-1.236173807
RAB36	1.136274809	FZD8	-1.235287879
FMC1	1.136167718	SIRPA	-1.234227646
LOC101106227	1.136106868	LGALS3	-1.233645938
LRRC27	1.135976687	RARG	-1.233572137
PDK1	1.135519219	LOC101114275	-1.233564122
LOC114118052	1.135105988	DYNLT3	-1.233558471
ASB9	1.134996814	ITSN2	-1.233273228

PATZ1	1.134543438	LOC105603910	-1.233111636
LOC105601981	1.13428853	MYOF	-1.232324624
LOC114118727	1.134214144	LOC114117545	-1.231560285
ENC1	1.133982029	SNX7	-1.231003514
TXNRD3	1.133503977	TRIM16	-1.229251736
LOC114112968	1.133300732	BNIP2	-1.229171606
MORN4	1.132748721	PPIP5K1	-1.228960653
RPS9	1.132732554	KPNA5	-1.228232158
LOC105603538	1.132707817	C25H10orf71	-1.227923874
PDE6C	1.132707817	PHTF2	-1.227668699
PELP1	1.132680814	UHRF1BP1L	-1.227094631
ANKRD13B	1.13175155	LOC105602268	-1.226358081
PGK1	1.131054155	UPB1	-1.226264768
CDO1	1.130980063	CMKLR1	-1.225486276
RPL18	1.130704286	OTULINL	-1.225426538
CHPF	1.130136003	FGF11	-1.225383831
MRTO4	1.130130452	BCL2L15	-1.225121449
LOC105607169	1.129677882	INSC	-1.225121449
MKX	1.129366748	PRXL2C	-1.225030215
DYNLRB2	1.129210955	CPQ	-1.224941056
LIG3	1.128747297	SAMHD1	-1.223680278
TMEM223	1.12872646	CSF1	-1.223675659
LOC101118433	1.12829599	LINGO1	-1.223458958
LOC443015	1.12799202	TRAF3IP1	-1.223226437
CCT7	1.127991998	ATXN3	-1.223223103
SPPL2B	1.127655451	GNB5	-1.223163537
HMCES	1.126304987	ANKRD29	-1.223113823
LOC101110022	1.126080329	DCAF17	-1.222837034
ASTN2	1.125793592	MIER1	-1.222603399
NCAPG	1.125284304	FAM214B	-1.222391136
NUP85	1.125149224	LOC114114509	-1.221791546
ALOX12	1.124696644	LCA5	-1.22161547
FARSB	1.124352597	LOC114116816	-1.221526137
FANCA	1.124211314	CTDSPL	-1.221493909
PRDX5	1.124101263	APBB1IP	-1.221342536
LOC101114018	1.124028672	ZBTB8A	-1.220527467
BEX2	1.122795652	ENPP2	-1.220445209
CTPS1	1.122091337	LMO3	-1.22032099
LOC101106975	1.121349536	PLS3	-1.220220331
HOXC8	1.121125128	CACNA1S	-1.220195496
CNPY3	1.120047842	SUSD1	-1.220195496
ZNF48	1.119982109	PIK3R3	-1.219929139
PSMD4	1.119501304	TIGD2	-1.219926055
MYH7B	1.119278857	C1QA	-1.218403982
PIMREG	1.118369015	LOC105604727	-1.217848878

PLEKHA8	1.1183684	MAGEE2	-1.216476049
ATG101	1.118165272	TP53I11	-1.215819253
PVR	1.117799361	ULK2	-1.215753114
LEMD2	1.117762699	PRSS23	-1.214267198
BTBD2	1.117586816	TCF4	-1.213454363
PYM1	1.116687372	LOC101112480	-1.213127883
SH2D3A	1.116526552	CCN1	-1.21271759
CCDC106	1.116286082	MPEG1	-1.211587375
DOCK5	1.116013616	LOC101105651	-1.211192671
VGF	1.114632985	SAMSN1	-1.211187949
AMDHD2	1.114316842	SERP2	-1.211077728
PRELID3A	1.113972089	WSCD1	-1.210984667
PSMB8	1.113788342	COL4A3	-1.210510091
LOC105608522	1.112563455	HEYL	-1.210402965
SLC8B1	1.112373936	PKD2	-1.210042847
IMPDH2	1.112080412	LOC101106743	-1.209388218
DCTD	1.111829891	ADAM10	-1.207815699
NOP9	1.111829891	XRN1	-1.207617713
TIMM50	1.111498468	CCP110	-1.207274224
LOC105604728	1.111452813	TTC28	-1.206432903
TMEM69	1.111056123	KCP	-1.205823862
AARS2	1.110592502	LOC101122123	-1.205795226
SDF2L1	1.110199009	IKBIP	-1.205514843
MESD	1.110193684	DLL4	-1.204535008
PFDN2	1.110024341	LOC114113868	-1.203924375
NDUFS8	1.109995747	LOC106990331	-1.203304496
KRI1	1.109711471	TUT7	-1.202370573
PRDX4	1.108906073	ACVRL1	-1.201985418
PGR	1.108855913	LOC114114813	-1.201724716
SEMA4B	1.108855913	LOC114117333	-1.201724716
NDUFS7	1.108544703	CD96	-1.201375641
POLL	1.108125727	ADM5	-1.200814261
LSM3	1.107551101	LOC114110248	-1.200734651
GLI4	1.106581689	BRMS1L	-1.200062248
ACSF3	1.106432232	PIK3CG	-1.199858083
DUS1L	1.106302	DAGLA	-1.199508452
TSPYL5	1.105952029	CNR1	-1.199458257
LOC105606441	1.105486364	TIE1	-1.199304128
KIF18A	1.104597334	DUSP2	-1.198791797
DAP3	1.104158582	RHOD	-1.198642762
HAX1	1.103905664	ING3	-1.198215692
PARD6B	1.10348469	CEP290	-1.19811369
RPL29	1.103014344	LOC105613342	-1.198081524
RAVER2	1.102967177	LOC114116910	-1.198015849
SERAC1	1.102852999	SMCO3	-1.197897291

TSACC	1.102685053
ERAL1	1.102476351
PTPMT1	1.101860914
LPIN2	1.101730763
SPON2	1.101304425
LOC114112987	1.100571957
LDHA	1.100507972
PPP2R1A	1.100438671
VRK1	1.100403603
CDCA2	1.100319227
GLYCTK	1.100136562

GLDN	-1.197828392
NME9	-1.197004239
SLC9A9	-1.196032439
ADAMTS9	-1.196022356
ATAD2B	-1.195971127
RGS1	-1.195222487
FRMD4B	-1.194966635
TANK	-1.194039481
FAM210B	-1.193668961
LOC114118082	-1.193257908
FGD5	-1.192936773
SH3BGRL2	-1.192103355
ALG2	-1.191593039
GRAMD1B	-1.191488839
STOM	-1.190220762
EHD4	-1.190101438
B4GALNT3	-1.189874066
GRAMD2A	-1.189874066
FAM13C	-1.189606304
SMAD9	-1.189469369
RASAL3	-1.189026352
PTEN	-1.188620128
QKI	-1.188321488
N4BP2	-1.18826567
KIAA1107	-1.188171086
LMNTD1	-1.187720444
LOC114108673	-1.187649232
TMEM158	-1.187071649
LOC101116298	-1.187061813
UBE2J1	-1.186868492
CLEC11A	-1.18634861
ZBTB11	-1.185830228
OAT	-1.18518167
SCNN1B	-1.184918288
RILP	-1.184576941
EPS8	-1.184330831
ITGAX	-1.184208583
CACNA1D	-1.18361164
C5H19orf71	-1.183567956
STX2	-1.183358521
AS3MT	-1.183043097
ITGA11	-1.182370064
ARL15	-1.181555937
PKHD1L1	-1.181254825
FSIP1	-1.179825746

ENAH	-1.179731702
CRTAP	-1.179180661
ARSI	-1.178952555
LOC114109611	-1.178564806
MARF1	-1.178346133
LOC114117965	-1.177942285
AGTR2	-1.177942285
C1H21orf91	-1.177422093
CREBRF	-1.177417224
TCAF1	-1.177396378
LOC114110567	-1.177315887
ITGAM	-1.176489484
LOC101112291	-1.176371092
LOC101110545	-1.175915465
INPP5D	-1.175645902
TACSTD2	-1.175597151
SEMA4A	-1.174572907
PLEKHG5	-1.174473647
RNF38	-1.174028184
ZNHIT6	-1.173264557
MTPN	-1.173058436
LOC106990829	-1.17281855
LOC101105810	-1.172734451
RAB2B	-1.172362704
LOC114113811	-1.1722396
KLF4	-1.17202596
FAS	-1.17185221
LOC101117485	-1.17185221
CMYA5	-1.171312286
CTIF	-1.170915488
MYL6	-1.170610192
LOC114109543	-1.170596216
LOC114111048	-1.169461998
LOC105611671	-1.168687926
DNAJC3	-1.168474935
BTBD3	-1.168051024
COL6A1	-1.167858587
RSBN1L	-1.167258573
LOC101111832	-1.166888132
ADAM9	-1.166574524
BBS5	-1.166473698
LOC105610540	-1.166392001
UNC80	-1.166392001
FBLN1	-1.166046838
SPSB4	-1.165839966

MEF2D	-1.16548283
FAM214A	-1.165080164
VIPR2	-1.164934797
CDCP1	-1.164700703
RTN4	-1.164469055
KLF12	-1.164434229
HOXA1	-1.16425734
CBFA2T3	-1.163752285
NRARP	-1.163416807
CLCN5	-1.163307716
EYA1	-1.163110046
TMEM154	-1.161704933
ZC3H11A	-1.160511747
KITLG	-1.159693031
LOC101103233	-1.158843389
LOC114117238	-1.158711979
KRAS	-1.157822036
WSB1	-1.157469353
PCDH10	-1.157378986
ARID4B	-1.15591328
GFOD1	-1.155589601
IL34	-1.155064361
PAK1	-1.154399138
PTPN12	-1.154398693
LOC114116374	-1.154284543
CBL	-1.154018439
TREX2	-1.153761227
LOC114114429	-1.153293752
CD93	-1.153022431
PRKCB	-1.153003412
CNOT6L	-1.152949768
CD9	-1.152596059
TMEM182	-1.152545959
SERTAD2	-1.152470582
ZNF774	-1.152384845
LRCH3	-1.151685563
PJA2	-1.150561962
SH3BP5	-1.150483284
ANKH	-1.150370566
PARVA	-1.150241163
LYSMD2	-1.149765109
TNFRSF25	-1.149420894
HELZ	-1.148996668
RABEP1	-1.148848232
PAMR1	-1.148707215

TMTC2	-1.148548784
C2	-1.148456219
ZSCAN23	-1.147572869
GPR157	-1.147572869
LOC101112936	-1.147270539
DZIP3	-1.14713931
TSC22D1	-1.146919266
FERMT3	-1.146880971
SNCAIP	-1.146718484
SKP1_2	-1.146384376
PCF11	-1.145739667
STRN3	-1.145565335
MTSS2	-1.14529519
AHCYL2	-1.144953358
ASB5	-1.144399989
GEM	-1.144190352
DENND6A	-1.143683095
GGH	-1.143598018
LMOD1	-1.143014559
CLCF1	-1.142727385
PDLIM1	-1.142482266
LOC114115283	-1.142470223
ZNF114	-1.141117586
C24H16orf72	-1.141112415
LOC101104745	-1.140942287
LOC101117364	-1.140942287
LOC105604630	-1.140579817
DNAJC12	-1.140480483
RNF217	-1.140280336
ACSL4	-1.13998541
LOC101118793	-1.139485807
PLD1	-1.138816344
ETS1	-1.138811119
CORO1C	-1.138808826
SPTBN5	-1.138478682
LOC101120322	-1.138244688
ISLR	-1.137401326
KCNAB1	-1.137192798
LOC105604882	-1.137153095
SERTAD4	-1.136478621
AGO3	-1.136425003
SEPT8	-1.135124741
ARHGAP4	-1.135103712
C11H17orf107	-1.134722978
NEXMIF	-1.134388916

SLAMF8	-1.134388916
GAS6	-1.134355717
DHRS3	-1.1324155
SDC3	-1.131030569
PHF20L1	-1.130858756
TGFB1	-1.129765772
CDNF	-1.129417456
NLRP3	-1.12916795
RNASE13	-1.12916795
SLC16A14	-1.128983655
PDE4DIP	-1.128193035
USP53	-1.127731952
NOD1	-1.127490407
DLG1	-1.127064375
ZNF132	-1.12605873
CARNMT1	-1.125918549
TGFBR1	-1.125905573
SLC30A1	-1.125593552
GRAMD2B	-1.125322896
ST3GAL2	-1.125057077
SLC25A16	-1.124894808
ZNF25	-1.124690157
RHPN2	-1.123997361
CALCOCO1	-1.123965028
CDC42BPG	-1.123780321
RPS6KL1	-1.123159589
SOCS4	-1.123012465
MYO1C	-1.12238106
COL13A1	-1.122138711
CLASP2	-1.12164255
MAPK4	-1.121301486
CD99L2	-1.121301055
CALML4	-1.121017563
MCU	-1.120873245
MCL1	-1.120574288
TRMT13	-1.119894885
SLC35A5	-1.119672708
TET3	-1.119272998
GON7	-1.119087279
INTS6	-1.118921114
RAB22A	-1.118666908
LAPTM5	-1.118416757
ZZEF1	-1.118049254
