# Supplementary material for: Ovarian activation delays in peripubertal ewe lambs infected with Haemonchus contortus can be avoided by supplementing protein in their diets
Source: BMC Vet Res. 2021 Nov 3;17:344. doi: 10.1186/s12917-021-03020-7 (PMC8565066; doi:10.1186/s12917-021-03020-7)
Supplement: Supplementary file 6 — Additional file 6. Full list of differentially expressed genes in control infected vs supplemented infected. [file 12917_2021_3020_MOESM6_ESM.pdf]

Ovarian activation delays in peripubertal ewe lambs infected with *Haemonchus contortus* can be avoided by supplementing protein in their diets

Paula Suarez-Henriques, Camila de Miranda e Silva-Chaves, Ricardo Cardoso-Leite,

Danielle G. Gomes-Caldas, Luciana Morita-Katiki, Siu Mui-Tsai, Helder Louvandini

Additional file 6. Full list of differentially expressed genes in control infected vs supplemented infected

Up-regulated FDR p-value ≤ 0.05 **Down-regulated FDR p-value ≤0.05**

Gene ID	Log fold change	Gene ID	Log fold change
LOC114111207	13.18399101	LOC114108772	-13.44933118
MGAM	12.31905165	LOC101121777	-12.77918542
LOC114113605	12.14560596	LOC114113596	-12.06126329
LOC114114475	12.07372978	LOC114114910	-11.9961834
LOC114116592	11.9392055	ADAD2	-11.69792094
LOC105605968	11.71995455	FOXR1	-11.40980676
LOC114114036	11.56380878	POU5F1	-11.39064134
LOC101114750	11.19321822	FIGLA	-10.98059255
MAGOH	11.16976964	CSMD1	-10.9612366
LOC114113045	10.98954597	NOBOX	-10.76683504
LOC101120732	10.86853867	LOC105607182	-10.71384677
DIO1	10.8440577	LOC114113671	-10.68267039
LEPR	10.74704651	LOC114109667	-10.4245256
LOC114108756	10.44239521	MYT1L	-10.41464805
SCN7A	10.3824584	LOC114114905	-10.37662558
CCDC190	10.23208908	LOC101113054	-10.30677591
SOSTDC1	10.1465888	LOC114112675	-10.30677591
LOC105603423	10.13048385	CCDC42	-10.27577307
LRRC15	10.10598424	MED4	-10.25472808
LOC114116112	9.206068764	TMSB15B	-10.21169412
ASB10	7.867907138	HSPA1A	-10.189686
COL8A1	7.651651372	LOC114110588	-10.15603123
FNDC1	7.002518178	LHX8	-10.14463621
LOC101114319	6.812386483	OPALIN	-10.12157257
LOC101122744	6.788432441	KLK12	-10.09813422
LOC105607367	6.74974722	CDHR5	-10.0743088
PRKG2	6.62432056	FBN3	-10.01296478
CLDN1	6.498707568	PLA2G1B	-9.974865984
LRP1B	6.436629848	CORIN	-9.948896101
ACAN	6.329546166	LOC101111769	-9.935733723
LOC106991302	6.304916187	ASTL	-9.935733723
LOC114117879	6.296297272	MAPK15	-9.854133293
KCNA6	6.281536796	LOC114115662	-9.840072955
MAP6	6.1972481	GLT1D1	-9.840072955
LOC114114489	6.054501745	STK31	-9.825874237
CAV3	6.018806383	DNAH10	-9.797050575
C1QL3	5.978200413	FMR1NB	-9.782419876
SPINT1	5.940757532	SEZ6L	-9.737615902
LRRC58	5.821742686	LOC114109057	-9.691375787
ELN	5.750748467	LOC114109046	-9.643604407

RNF183	5.746120782	TDRD5	-9.627322508
SUSD5	5.708184079	MOS	-9.627322508
CLCA2	5.670022311	LOC101105265	-9.627322508
PI15	5.653518471	ESRP1	-9.627322508
LOC105602432	5.631968899	HS3ST3A1	-9.610854757
GRIP2	5.506311418	FRMD5	-9.59419686
CDH8	5.47400988	LOC114109676	-9.577344376
SLC6A2	5.469414066	CYP19	-9.507894046
HOXC10	5.421492123	KCNC2	-9.489996203
TRIM66	5.371502964	SALL3	-9.453520308
LOC105612842	5.345396253	LOC106990690	-9.416098212
MCF2L2	5.301113602	HAL	-9.377679515
LOC114113180	5.278516322	PCLO	-9.377679515
C7	5.242055614	LOC101111382	-9.358079573
LOC101117431	5.22863565	ATP1A3	-9.358079573
CDA	5.202770795	FATE1	-9.358079573
PRR15L	5.159475064	OXTR	-9.338209681
LOC114116917	5.14299534	NLRP14	-9.318062299
LOC101118459	5.126731447	ADAD1	-9.318062299
GLP1R	5.10316644	LOC101102411	-9.318062299
LOC114111483	5.088767129	SKOR1	-9.318062299
ITIH4	5.078595108	RSPH14	-9.297629567
SORCS1	5.059999556	CCDC105	-9.276903287
FMOD	5.042140741	AWAT1	-9.2558749
LOC101108519	5.029797283	ADRB3	-9.2558749
HNF4A	5.015874685	FOXJ1	-9.212875653
LTBP2	4.994008221	DPEP1	-9.122830229
SCG3	4.957079172	LOC114112978	-9.122830229
LOC101103260	4.880046157	DCHS2	-9.122830229
LOC101107282	4.871506495	LOC114109518	-9.104684282
SLC8A2	4.85147736	DDX25	-9.075608319
MFSD4A	4.851342674	ATP2C2	-9.075608319
ANOS1	4.816991776	TMEM52B	-9.051404804
LOC114117319	4.815314039	LOC105610274	-9.026788298
CCDC150	4.798666782	GABRG2	-9.001744461
RGL3	4.798666782	SALL4	-8.92389385
SEMA3E	4.762216223	RGR	-8.92389385
LOC114112975	4.754032986	LOC114108622	-8.92389385
BMX	4.739688167	LOC101103165	-8.92389385
SRPK3	4.730413392	LOC101109652	-8.92389385
LOC105614868	4.694535544	HRK	-8.896981253
TRIM63	4.694535544	LOC101117443	-8.869557059
SLC6A17	4.673939725	LOC105615770	-8.869557059
SGCA	4.64902021	LOC101120595	-8.869557059
FN1	4.630027763	LOC105603234	-8.869557059
HOXD10	4.602141398	LOC114114062	-8.813093395
TNC	4.561805542	LOC101119111	-8.813093395
LOC101122263	4.553271984	LOC114109005	-8.813093395

LOC105611006	4.548402341	LOC101117493	-8.784010646
SBSPON	4.544805989	ACTL8	-8.754329547
P2RY12	4.542859544	GAP43	-8.754329547
LOX	4.541726605	TRIM67	-8.754329547
SFRP1	4.536741041	LOC105604251	-8.724024958
LOC114117807	4.513248664	LOC101120961	-8.693070121
SERPINF1	4.504756302	CLEC1B	-8.693070121
COL24A1	4.489456704	UBE2U	-8.693070121
SELL	4.475275356	MAEL	-8.661436519
RET	4.45968754	LOC114115688	-8.661436519
GPR34	4.455716065	GABRA5	-8.661436519
RYR2	4.443749735	LOC114111539	-8.629093715
MFAP5	4.436534665	KRT77	-8.629093715
LOXL4	4.42328131	DNAAF1	-8.629093715
SNX20	4.420233763	LOC114110833	-8.629093715
LOC105613001	4.417321487	NR0B2	-8.629093715
STMN2	4.398470486	PIWIL2	-8.629093715
SIGLEC1	4.398432165	LOC114110304	-8.629093715
LOC105612542	4.39771997	RBMXL2	-8.629093715
HRCT1	4.394862863	TRIM50	-8.629093715
LOC114113140	4.391818387	RHOV	-8.59600918
LOC114117594	4.390028598	CPB2	-8.562148092
LGI2	4.372760534	SLC10A4	-8.562148092
LOC114110252	4.359209178	LOC101103335	-8.562148092
LOC101122488	4.352256482	NUTM1	-8.562148092
THSD7B	4.33095962	BFSP2	-8.562148092
AP5Z1	4.328251433	LOC101103644	-8.52747312
CCDC9B	4.326434821	CCDC188	-8.52747312
NPPC	4.304261532	WNT7A	-8.491944172
KAZALD1	4.29930421	RTL10	-8.491944172
LOC105605457	4.287500664	SLFNL1	-8.491944172
PLA2G5	4.280525776	LOC114115291	-8.491944172
LOC114113974	4.267192267	LOC105604203	-8.491944172
CRLF1	4.266662931	LOC105604801	-8.491944172
LOC114110654	4.259220254	LOC114112907	-8.491944172
PLCD4	4.231704766	LHFPL4	-8.491944172
NOXO1	4.231704766	DSCAML1	-8.491944172
CCL1	4.231704766	TCL1A	-8.491944172
CCDC80	4.221781249	CRH	-8.491944172
LOC101106041	4.212166965	SOHLH1	-8.488292104
FHL5	4.187298485	MLN	-8.455518121
SH3RF2	4.181223607	LOC105603863	-8.418148487
CDH13	4.177255656	BNC1	-8.418148487
OSCAR	4.155304801	GPR84	-8.418148487
CFI	4.148234878	ACHE	-8.418148487
HBB	4.139814769	FAM71F1	-8.418148487

DLK1	4.134690946	CRYBB2	-8.418148487
LOC101103244	4.1315134	CCDC181	-8.418148487
ADRB1	4.128911818	LOC101118216	-8.418148487
THRB	4.128717833	CNTN3	-8.379785082
KCNA5	4.123762002	DNAAF3	-8.340373603
ANGPTL1	4.1001006	CCDC87	-8.340373603
LOC105603244	4.095968211	LOC106990997	-8.340373603
CHI3L1	4.090856996	LOC114115313	-8.340373603
LOC101119226	4.081395176	KRT28	-8.340373603
CCN3_2	4.079001701	LOC105608334	-8.340373603
NR4A3	4.074631619	LOC101109545	-8.340373603
LOC101102227	4.074631619	SHISAL2A	-8.340373603
LNPEP	4.073985315	ELANE	-8.340373603
LOC105616533	4.06293882	MYPN	-8.340373603
LOC114115282	4.061032999	RBFOX1	-8.299855174
LOC114114528	4.060630572	GSTO2	-8.299855174
ADGRL3	4.057560564	PABPC1L	-8.299855174
LOC114113918	4.056857459	SMIM32	-8.258165809
LOC105616901	4.053392505	LOC105601887	-8.258165809
LOC114112092	4.046705956	CRYBG2	-8.258165809
AOC2	4.046705956	CERS3	-8.258165809
STS	4.036218521	LOC105612031	-8.258165809
SLC38A5	4.006919355	LOC101102519	-8.258165809
LOC114114563	4.006919355	GBX2	-8.258165809
LOC105606212	4.006919355	RNF208	-8.251541415
PKP1	4.006211888	INHA	-8.062709998
LOC101117706	3.999125951	GCG	-7.956999461
LOC106990096	3.993698409	ZAR1	-7.555178824
LOC101107463	3.989178729	ZP3	-7.522251795
LOC114117243	3.989178729	LOC101117395	-7.490597194
RPL3L	3.989178729	NPM2	-7.402969572
IL17B	3.987402431	MINAR2	-7.401501955
LOC101113122	3.986851894	IHH	-7.355141382
FGF2	3.982800204	CD164L2	-7.293297909
LOC114114043	3.979515934	PHACTR3	-7.248924904
HTR2B	3.976795184	LOC114115650	-7.144464004
FCER1A	3.959531395	CKM	-7.104203843
GHSR	3.959531395	NLRP5	-7.096788434
LOC114110089	3.955579549	LOC106990495	-7.092048172
LOC114113034	3.929262004	TPT1_1	-7.057802182
PRDM16	3.902932162	JSRP1	-7.053540041
TIGD4	3.902790947	GALNT14	-7.020459415
TMEM71	3.901651199	LCN8	-7.019119191
LOC114113672	3.900530417	ACTL7B	-7.003941434
PDE3A	3.900199069	LOC101109939	-6.960439858
HAND1	3.89834389	ZP4	-6.90207521

CASQ2	3.89834389	CCNO	-6.868710934
LOC106990575	3.89834389	ATG9B	-6.86326852
SGCG	3.897208228	LOC105609277	-6.772816906
CCN3_1	3.896235168	LOC105604950	-6.744914276
LOC101108520	3.887257425	FAM167B	-6.52948361
S100A1	3.887081326	LOC114116088	-6.527677593
DPT	3.875852601	GPX2	-6.442092471
LOC101105989	3.875135398	LOC101111664	-6.421703691
FOXC2	3.86336977	HSD17B1	-6.353286646
LOC114110605	3.857566615	SLC47A2	-6.324838815
TGFB3	3.852439499	KHDC3L	-6.310948231
VAT1L	3.850319224	TAFA3	-6.300199556
EFHD1	3.84959404	FAM83A	-6.27737725
SLIT3	3.839958297	LOC105613214	-6.245206339
LOC114113913	3.834445924	LOC101121371	-6.240731146
FBN1	3.831844411	LOC114114998	-6.221002824
CNTN2	3.825656975	AMH	-6.196871357
NGFR	3.820425895	CHADL	-6.196386318
HTR1B	3.817167354	LOC105607956	-6.145856216
LOC101112671	3.816726071	ZGLP1	-6.10460407
LOC114115665	3.813283965	KCNJ5	-6.09349187
GFRA1	3.803073725	B3GNT4	-6.067150426
ND6	3.801403337	CCDC60	-6.067150426
LOC105608837	3.801403337	LOC114113051	-6.020363422
GPR156	3.795883899	GALNT9	-5.966131317
GXYLT2	3.794303225	LOC114113227	-5.930700038
FOSB	3.791375059	OOEP	-5.902078038
NFASC	3.791254308	LOC101106720	-5.90159786
CDH7	3.7831464	INSRR	-5.893137465
MCTP2	3.774809017	UTS2R	-5.875258328
MYOM1	3.774792721	PADI6	-5.824817962
LOC114108991	3.774778517	LOC114118734	-5.824754927
HOXC11	3.774736207	FST	-5.821499631
LOC101123341	3.772828997	ELAVL3	-5.800328099
TEKT3	3.758691402	LOC114115359	-5.798455722
SLITRK6	3.758007628	SLC1A6	-5.729425292
PARM1	3.755090684	LOC114113186	-5.679449627
BCL2	3.753273905	NELL1	-5.665973214
LOC114110464	3.749938924	LOC101106086	-5.612965211
RAMP1	3.748655615	LOC105610869	-5.553374924
SHISA3	3.742539533	BSPRY	-5.551658817
LOC114114545	3.734960805	LOC101111397	-5.538661544
LOC101122501	3.731423478	GSTA1	-5.533447692
CHAD	3.72357918	BBOX1	-5.53063043
HRC	3.709389967	LOC105610712	-5.509971623
REEP1	3.707662561	STC2	-5.479923843

LOC101104661	3.703523561	LOC105602721	-5.465641216
LOC114115405	3.700943259	PIP5KL1	-5.443310877
LOC114109650	3.700943259	LOC114109675	-5.422144186
LOC114114537	3.697476713	MYBPC3	-5.400877253
SHOC1	3.697476713	FAM163A	-5.377522891
RBM20	3.693886546	LOC114109433	-5.37022511
BTC	3.693827906	LOC101123158	-5.350363849
CCN4	3.693161082	LOC101121518	-5.330732324
RGS5	3.691450474	INSL3	-5.317405443
ACTC1	3.688443543	MAPK8IP1	-5.269466892
LY75	3.686162056	KIF17	-5.269038115
GJC3	3.662386803	RHBG	-5.254079565
PODN	3.65832319	ARL14EPL	-5.251013726
SRL	3.654715217	NTRK1	-5.232151311
CNTN1	3.653893837	CXCL14	-5.22614568
LOC101111069	3.644402186	SH3GL2	-5.203929354
LPCAT2	3.636445835	F2	-5.193848873
TMEM38A	3.634875423	LOC106991580	-5.188998477
LOC114108767	3.63111142	LOC101106541	-5.171736783
LOC114110816	3.63111142	LOC105607437	-5.171736783
LOC114118858	3.63111142	ZIC3	-5.148636616
LOC114114600	3.629576659	LOC114116813	-5.148636616
DQA	3.629465511	GCNT3	-5.141890073
CYB561	3.628352539	CLCNKA	-5.134418648
PI16	3.624803741	LOC105608949	-5.116356971
FAM180B	3.624136881	LOC101111695	-5.114358647
LOC106990432	3.623785379	PLPPR1	-5.112729075
LOC101109482	3.623785379	MSMB	-5.10447332
LOC101104401	3.616575402	LOC114112981	-5.102887947
ADRA1A	3.611713054	LOC105601897	-5.095729226
NTRK3	3.609981061	LOC114108759	-5.071189065
DDR2	3.60472935	SNX22	-5.052722296
LRRC39	3.600232118	HKDC1	-5.052722296
ATP6AP1L	3.599715369	IL17RB	-5.052240261
KBTBD11	3.595956633	LOC114108618	-5.046030128
FGF7	3.593738411	MGARP	-5.044132607
PROX1	3.590151044	LOC114113648	-4.985804382
FGL1	3.584298649	LOC114109368	-4.973960092
EFEMP1	3.583086861	TTC36	-4.936269422
SOX7	3.580377363	LOC101102259	-4.936192049
PTGER3	3.57598506	LOC114117971	-4.927169804
KCNMB1	3.573113601	KLK4	-4.927169804
LOC105610709	3.57225392	LIME1	-4.903849245
TNFSF13B	3.566096623	LOC114116367	-4.890648389
LOC114108725	3.56508682	GDF5	-4.885985729
LOC114110615	3.560245545	DNAH11	-4.882154277

RGS20	3.558362595	LOC114114534	-4.866050849
TNFSF8	3.551433056	LOC114109349	-4.866050849
LOC114114280	3.546126399	LOC105612144	-4.866050849
CELA1	3.546126399	LOC105602330	-4.866050849
LOC105604745	3.546126399	COLQ	-4.860806713
SERPINB5	3.546126399	LOC101122984	-4.855576481
FZD6	3.539412272	LOC101118990	-4.854861775
POPDC2	3.534864136	NLRP9	-4.836903622
PRRX1	3.533314686	MYL6B	-4.814085189
LOC114117356	3.529993646	NLRP13	-4.809065389
SNCG	3.525343555	FOXL2	-4.804626827
LOC114116179	3.51911901	LOC114115315	-4.80222865
DOCK8	3.518765904	LOC105616091	-4.802227638
LOC101123419	3.515718296	WNK3	-4.778025507
TPSB2	3.514421842	LOC105605002	-4.759195515
HCAR1	3.513005519	LOC101121119	-4.751054551
FBLN2	3.509623316	RIPPLY2	-4.735449743
TMTC1	3.498764083	ZACN	-4.730273651
DDO	3.498107576	GTSF1	-4.713309259
LOC114110437	3.498107576	SHANK2	-4.7080352
HOGA1	3.49672865	SHISA8	-4.696107013
RASA2	3.494158759	PRRT3	-4.692904017
LOC114116078	3.481155937	LOC101104943	-4.692904017
FAM151A	3.480408298	LOC101108295	-4.690275348
LOC114116827	3.480408298	LOC114112724	-4.685574112
LOC105607091	3.480408298	KCNE1	-4.669282262
LOC114111010	3.480408298	MMP1	-4.668565365
LOC114115572	3.478328834	HYDIN	-4.665430256
GASK1B_1	3.476667454	LOC114116953	-4.665430256
GJA5	3.472820088	TNNI3	-4.663162732
LOC114114058	3.464048089	GRHL1	-4.659923515
GRIA3	3.458222864	LOC114111372	-4.654540612
LOC114116061	3.45816972	LOC105602163	-4.654540612
DKK2	3.454440523	CADPS	-4.628102946
FAP	3.451702455	LOC114108609	-4.625919237
NAV3	3.446149769	PNLDC1	-4.618546451
MAP3K7CL	3.446057782	LOC105610401	-4.615129133
LOC101118470	3.439365006	C6H4orf19	-4.615129133
COL4A5	3.436657466	RIPK4	-4.615129133
LOC114114030	3.43563291	DMRTA2	-4.615129133
FFAR4	3.433204467	DRC7	-4.615013837
ADAMTSL5	3.427178777	ALDOB	-4.609869774
LOC105616215	3.42566646	LOC105607655	-4.591838336
WIF1	3.42498554	LOC114112736	-4.591838336
LOC114112977	3.421188696	DCLK3	-4.590287395
HAS2	3.41774735	KIAA1211L	-4.583588674

ITGA7	3.413483514	TMEM266	-4.570674635
PDGFRL	3.411660249	LARGE2	-4.564028942
SLC24A5	3.410861124	LOC114115623	-4.548884058
LOC105605293	3.39871028	PHYHIP	-4.545628568
LOC114108813	3.39871028	CHRNA2	-4.54462673
LOC101122940	3.396348861	CITED1	-4.540950833
GPR21	3.396239232	GLIS1	-4.539714098
ITGA1	3.395956321	LOC114116861	-4.532921339
TSTD1	3.395152466	LOC106991313	-4.517308201
MPPED2	3.393740381	LOC105602473	-4.514289756
LOC101109425	3.392090453	SLC35G6	-4.514289756
LOC114113690	3.385293383	NXPH2	-4.514289756
DPY19L3	3.381717411	LOC101119941	-4.514289756
NRK	3.377819356	LOC114115326	-4.512256233
LOC114114096	3.377016956	GRHL3	-4.489991343
LOC105616812	3.377016956	LOC105603132	-4.488786867
LOC114115593	3.377016956	OVGP1	-4.47690143
RGS7BP	3.376461637	LOC105611998	-4.472566757
LOC101112335	3.376235192	ATP6V1C2	-4.470373283
KLHL29	3.374522611	AURKC	-4.457114736
RNASEL	3.370741388	TVP23A	-4.455788945
CD109	3.363601036	LOC105603748	-4.445744606
LOC101112822	3.360154094	ANO5	-4.445744606
ADRA2A	3.355168991	OTX1	-4.445744606
THSD4	3.35384826	LOC101108898	-4.445744606
MYO18B	3.35028857	TDRD1	-4.445251205
CALHM5	3.344117055	LOC114116087	-4.443011749
LOC114118067	3.340944407	NMB	-4.440386681
PPARGC1A	3.338629389	LOC105610334	-4.432334763
LOC114114853	3.338284551	CLEC2L	-4.426415177
HAND2	3.337246043	CHDH	-4.413044755
PPFIA4	3.335943257	TLE6	-4.408017565
LOC114113976	3.331449956	LOC114116771	-4.388057221
LOC114113068	3.331130065	MRO	-4.381686258
HDAC9	3.323292894	PLCH1	-4.357143982
LOC105602647	3.321858499	NEFM	-4.354809052
LIMCH1	3.32087622	CAMK2B	-4.352959389
C3H9orf50	3.317996921	DRC1	-4.352959389
LOC101117577	3.315623886	LOC101122710	-4.345442271
SCML4	3.312106615	FOXG1	-4.345442271
LOC101102057	3.310951128	FAM189A2	-4.325921648
FAM124B	3.310835233	KCNK12	-4.322811192
HSF5	3.297052643	LOC114115610	-4.322629883
LOC101107224	3.285750257	LOC114110984	-4.320919989
FBXO15	3.284396666	SCN5A	-4.307876613
LOC114118363	3.280782738	HES6	-4.306613881

ART4	3.278699145	LOC114114056	-4.304228619
CDKL1	3.275416994	NKAIN3	-4.304228619
LOC114116678	3.271790656	OGDHL	-4.302172386
RAB17	3.269895738	LOC105611558	-4.27700347
PRUNE2	3.269311843	LOC114108815	-4.254490869
ZSCAN31	3.269222586	NSUN7	-4.254249534
CCDC89	3.269222586	CEACAM19	-4.253794126
EDIL3	3.26790751	LOC114114547	-4.253794126
KCNC1	3.263948034	LTF	-4.253794126
GLT8D2	3.263913172	SHISA9	-4.252978991
TMEM26	3.259646363	PIPOX	-4.252978991
DNHD1	3.257779946	LOC101122577	-4.252978991
CLDN10	3.257624104	LOC114114850	-4.252978991
ATP8B4	3.24948641	LOC106991918	-4.243578202
SVEP1	3.2490431	SHISA6	-4.229067479
LOC114116862	3.248575417	LOC114116870	-4.222832368
MID1	3.245770288	MTHFD2L	-4.218564875
ADARB1	3.24462556	LOC101108627	-4.212868259
CHMP4C	3.237218346	LOC101103584	-4.192648031
MRC1	3.23679563	LOC114118758	-4.192194329
CCM2L	3.235773183	REC8	-4.187181776
LOC114117315	3.235756851	SAXO1	-4.166443677
DHRS9	3.235756851	RSPO2	-4.164418744
LOC114116172	3.235756851	UPP1	-4.156632518
OLR1	3.230065918	PLCH2	-4.15418139
PIANP	3.218549736	SLC16A8	-4.15418139
FAM217A	3.207991915	LOC114113253	-4.15418139
UNC13C	3.197956084	AMBP	-4.15418139
COL5A2	3.195417226	LOC114113601	-4.15418139
LGR5	3.185419778	NOX5	-4.1473062
XDH	3.185419778	TAS1R3	-4.1473062
LOC105613828	3.185419778	LOC114109549	-4.1473062
KMO	3.185419778	TDO2	-4.1473062
CCR8	3.183380509	SLC5A5	-4.1473062
PPP1R14C	3.179660469	CLIC6	-4.146824501
LOC105604156	3.179338231	LOC105608648	-4.143449079
METTL24	3.179238601	TMIE	-4.143449079
CNGA3	3.173416658	LOC105610844	-4.141167492
FAM20A	3.141999239	CALY	-4.122492731
AQP7	3.139881903	LOC114117885	-4.091653353
CD80	3.139544804	CYP17A1	-4.085999904
COL15A1	3.138398808	LOC114114001	-4.084685231
ADAMTS1	3.135356911	CYP17	-4.082057137
LOC114116365	3.1332627	LOC114115115	-4.068528931
CXCL12	3.123370546	SLITRK1	-4.062042725
CHML	3.116099004	TEKT1	-4.062042725

CD44	3.110224318	APOB	-4.054380642
LOC101112834	3.104789945	ENPEP	-4.035096505
GFI1	3.09655511	NEFL	-4.032346626
TMEM255A	3.096170175	LOC114117581	-4.032327314
FRMD6	3.092662787	LOC105602037	-4.032327314
FBXO48	3.084223776	MOGAT1	-4.032327314
RASSF9	3.081662448	SV2A	-4.02804926
LGR6	3.079149057	CDH2	-4.024475441
DLX5	3.079149057	LOC106991743	-4.023425805
TNS4	3.079149057	LOC105615197	-4.014525076
NIPAL1	3.079149057	CRISP2	-4.0010217
COLEC10	3.079149057	STC1	-3.995079844
B3GALT2	3.079028963	NR5A2	-3.984426805
NEK11	3.078316916	CPNE5	-3.983466922
LOC101120386	3.077893037	ALOX15	-3.977432973
LOC100144429	3.077275764	DNAH12	-3.971208359
LOC114118855	3.077138292	LOC114117299	-3.971208359
STK32A	3.070618105	LOC114116443	-3.941429397
LOC101110521	3.067165556	FAM83F	-3.936324888
CNTNAP1	3.067106102	SCRT2	-3.933632502
FLT3	3.066903656	LOC105604908	-3.933632502
FCMR	3.066903656	LOC114113830	-3.933632502
GAS2	3.066519065	NKX2-1	-3.933632502
HECTD2	3.058920609	EPHA8	-3.933632502
LOC114113733	3.056698308	FMO1	-3.933632502
IGF1	3.055545914	KPNA7	-3.930083574
MEDAG	3.054044243	GCLC	-3.925054474
NDRG4	3.042449231	ADAMDEC1	-3.919273217
C1QL1	3.039903185	EIF4E1B	-3.910904095
RNF150	3.037575908	HMGCS2	-3.907385148
SORBS1	3.036155312	CCDC13	-3.892503777
HAPLN1	3.030037214	MOV10L1	-3.889366517
PCDH15	3.02799555	HS6ST2	-3.875071093
POSTN	3.02761219	SLC16A3	-3.866402008
GALNT16	3.027222367	LOC114109056	-3.860209275
LOC101115508	3.026696549	CPAMD8	-3.848976715
SHCBP1L	3.025419026	LOC105616575	-3.845934127
SLC24A1	3.025419026	LOC101102096	-3.840607253
MEGF10	3.023856613	LOC105603379	-3.840021477
NUDT9	3.018529395	LOC101118224	-3.839842466
NHLRC3	3.017207775	SSTR1	-3.81250082
DTWD2	3.017094171	NPVF	-3.79744095
ARHGAP15	3.00930156	FDXR	-3.79327622
AKAP6	3.006181537	C2H2orf72	-3.785873805
LOC105612625	3.005492971	LOC105604541	-3.785873805
NRIP2	3.004420471	LOC114109553	-3.776242557

RASSF10	3.000776796	SPRN	-3.776018114
C3AR1	2.999813831	LOC114117603	-3.774139004
LOC114113956	2.998647177	CDH12	-3.772561694
RERGL	2.997198385	TMEM191C	-3.770587766
FXYD7	2.997049215	LOC105604928	-3.770587766
LOC114110414	2.997049215	LOC114109337	-3.770587766
STAC2	2.994406054	GPR3	-3.770587766
LOC105611786	2.988008976	LOC114110815	-3.769615767
LOC114109038	2.986067059	ADGRB1	-3.766887325
LOC105612432	2.986067059	LOC105609280	-3.766157619
ADAM12	2.983233277	CHAC1	-3.765681169
CCDC102B	2.978450557	LOC443320	-3.764571196
CAMK2A	2.978450557	FGFR4	-3.760377045
CRIP1	2.972780305	INHBB	-3.757249965
ARHGAP29	2.97077571	ALPL	-3.756427596
LOC114117257	2.964423372	LOC105603166	-3.755997257
PADI4	2.964423372	LOC114111330	-3.753345885
ADAMTS15	2.961482691	LOC114109701	-3.748504171
LOC105611988	2.954916572	GPT	-3.740472579
ABCD2	2.952434395	SCN10A	-3.722842393
LOC114116372	2.946945693	IGSF11	-3.711636248
LOC101111669	2.946882441	LOC114118386	-3.710140766
PNMA2	2.946882441	LOC114110143	-3.708705611
SOWAHC	2.94319696	TNFRSF13C	-3.706818418
LOC100134870	2.942757845	LOC106990570	-3.705659298
PTGIR	2.93747207	GDF9	-3.703467444
SSC5D	2.935139593	CCDC155	-3.702550774
CEACAM16	2.934333245	TNNT2	-3.697190192
LOC114109535	2.930504203	LOC105605961	-3.696995846
ANKRD6	2.927247022	LOC105609125	-3.696995846
BEAN1	2.925871008	LOC114116384	-3.692730374
SLC22A3	2.919595762	LOC106990117	-3.691160002
HOXA10	2.915325064	PLEKHH1	-3.684699051
RNF125	2.913029691	LOC114115412	-3.682267594
FSTL1	2.907832035	GPR162	-3.677042811
LDLRAD4	2.905861881	LOC114111236	-3.677042811
ZNF831	2.903447434	LOC114109099	-3.67317509
NPAS4	2.903447434	LOC114113201	-3.67317509
LOC105608584	2.896742932	TEX15	-3.670729288
S100B	2.892968834	BTLA	-3.670729288
CREB5	2.887424626	EMID1	-3.665547259
HSPB6	2.884793581	LOC114113975	-3.653397621
NCALD	2.881879062	RIMKLA	-3.650472734
ABCA10	2.881553698	LOC114112250	-3.621232686
PAQR7	2.88099571	UNC79	-3.619447266
FBXL22	2.880587435	LOC105606717	-3.619447266

LOC114117274	2.873858424	LOC114113260	-3.619447266
LOC114111267	2.871963744	LOC101120447	-3.619447266
MALL	2.861698079	LOC114110131	-3.609271479
LOC106991530	2.860935777	NOS2	-3.609039855
DOCK10	2.850111359	TMEM179	-3.608592793
LOC105605028	2.849623402	LOC106991070	-3.603138878
TP53INP2	2.848513808	LRRN4CL	-3.589513424
CCR4	2.839780161	LOC105611015	-3.588521493
PERM1	2.839780161	NOTUM	-3.585883255
ABRA	2.839780161	CDCA3	-3.579906236
NAALADL1	2.838474252	DHCR24	-3.576033197
ZNF502	2.838383418	CCL25	-3.567508963
SLC9A7	2.838383418	LOC114117782	-3.562218295
DMRT2	2.838220715	SLC44A3	-3.542827065
NTN4	2.833096488	LOC105603076	-3.542827065
TMEM59L	2.831054604	C2CD6	-3.537492273
PCNX2	2.828845036	ALDH8A1	-3.537492273
LOC101116441	2.826932555	MYBL2	-3.523041551
ECM2	2.825895087	LOC114112722	-3.52288744
RASGRP1	2.825426317	LOC105604743	-3.52288744
P2RY13	2.824384026	LOC105612436	-3.520534299
FCHO2	2.821724546	FABP3	-3.515051449
CHODL	2.819601932	TCTEX1D4	-3.50134476
FILIP1	2.817620671	RTL1	-3.50134476
LOC105616380	2.816957617	LOC105606685	-3.49781498
ANO3	2.815623241	F10	-3.496366481
NUDT13	2.815451882	PROM1	-3.475566481
PDE5A	2.813168326	MTFP1	-3.474651031
PTPRB	2.812081081	MCRIP2	-3.471015347
DSC2	2.810609633	C15H11orf87	-3.470496028
MSC	2.808107684	BAIAP3	-3.458944385
LOC114111493	2.805104007	LOC106991954	-3.457483279
LEKR1	2.805104007	DNAI2	-3.455697944
C1S	2.803676525	PHYHIPL	-3.450599781
SESN3	2.801186175	LOC105601991	-3.450599781
LOC105607925	2.797843424	TMEM229A	-3.450599781
COL3A1	2.797079261	AK9	-3.450599781
P2RX1	2.795894449	LOC105615767	-3.450599781
NEXN	2.792420209	LOC101114089	-3.450599781
GASK1B_2	2.790252166	CFAP157	-3.450599781
CLEC12A	2.786736706	LOC105612071	-3.450599781
KCNT2	2.780451303	LOC114113194	-3.450599781
SUSD2	2.779705901	SLC29A4	-3.450074223
ANKS6	2.777277093	CHGA	-3.447103708
LOC114109398	2.773172961	TACR3	-3.443391839
BST1	2.772523531	LOC101122142	-3.442419352

C1QTNF2	2.772083982	LOC101110855	-3.441617883
VSIG10L	2.769542783	PPARG	-3.430225094
ACAP2	2.769479186	CDH1	-3.425849676
RASL10A	2.76927325	LOC114112870	-3.425849676
KL	2.768401129	LOC101104074	-3.425849676
C3H12orf75	2.768233327	LOC114116336	-3.423073532
LOC101113086	2.768208374	LOC114115022	-3.423073532
CTNNA3	2.768111728	LOC101118452	-3.423073532
LYZ	2.762117065	C11H17orf97	-3.420525688
LOC101108006	2.761731214	ATF7IP2	-3.411515173
GPR37	2.75663676	CCDC116	-3.411325019
PLEKHD1	2.753793095	TRIM9	-3.410385316
GPR17	2.751184607	JAKMIP1	-3.410385316
LOC114118743	2.751184607	LOC114113912	-3.408559543
LOC101110777	2.751184607	CFAP52	-3.408559543
LOC101112891	2.751062636	PCSK4	-3.408559543
SEMA3G	2.74513547	ANKLE1	-3.400925746
GPR20	2.744916946	DNASE1L3	-3.394893082
CD7	2.744182206	PC	-3.386406561
EPB41L3	2.741671079	DNA2	-3.377884098
BCHE	2.737595267	EPS8L2	-3.368222761
TMEM220	2.735182819	LOC114116963	-3.360594786
IL2RA	2.734994532	LOC114115006	-3.358136501
LOC114118016	2.731896457	LOC114111295	-3.358136501
BRINP3	2.731669172	PLSCR5	-3.355103877
KCNQ4	2.729578102	DIRAS1	-3.355103877
ADIRF	2.728176234	LOC114118691	-3.355103877
SLC41A2	2.725763412	LOC101117799	-3.355103877
LOC101112688	2.724337327	LOC114112835	-3.355103877
CNN1	2.723740115	GLIPR1L2	-3.355103877
TBX21	2.722885285	LOC114112825	-3.355103877
SYNM	2.722299001	LOC114117857	-3.355103877
LOC105602254	2.721579294	LOC114118888	-3.355103877
WFDC1	2.721236909	GBGT1	-3.344353972
MFAP4	2.720568119	MSLN	-3.335863275
SULF1	2.719857764	PPIF	-3.335154357
LOC105613002	2.719682596	ORC1	-3.32978634
CHST3	2.712027834	SLAIN1	-3.32950141
LOC114110341	2.71058206	FAM155A	-3.326779652
LOC105605766	2.710021427	CREB3L3	-3.322445544
ZNF366	2.704289234	TMEM262	-3.30939428
LOC114115581	2.703341122	IQANK1	-3.30939428
ANKRD31	2.703341122	AZIN2	-3.30939428
DLGAP1	2.703341122	TRHDE	-3.309242486
LOC105610178	2.703341122	NPTX2	-3.306974375
LOC101118212	2.703341122	ESPN	-3.300352807

LOC105609508	2.703341122	EMILIN3	-3.296002721
LOC114114542	2.703341122	SLC5A8	-3.292472426
LOC101118024	2.703341122	SSTR3	-3.284916649
LOC114111026	2.698619324	CHRNB4	-3.282267045
LOC105610161	2.698619324	EPHX2	-3.279568793
RXFP4	2.696986017	HCN2	-3.26959822
FAM83E	2.696986017	TTR	-3.265534825
LOC114110991	2.696986017	SAP25	-3.265123998
IL2RB	2.695537356	RBPJL	-3.2593389
LOC101119706	2.695184813	LOC114113256	-3.2593389
MYOCD	2.693830489	RPRML	-3.2593389
ADAMTSL3	2.688730497	SLC45A1	-3.2593389
EOGT	2.685062448	TMIGD2	-3.2593389
CILP	2.683911112	DUPD1	-3.2593389
MLKL	2.678064436	LOC114110618	-3.2593389
MST1R	2.677338973	GAPDHS	-3.257132603
COL21A1	2.675873097	HPN	-3.25635733
IL15	2.675229684	UNCX	-3.255560769
LOC114114762	2.674040304	CALB2	-3.25395975
ABHD16B	2.671966224	MRPS25	-3.242652186
RUNX1T1	2.671053088	MAP7D2	-3.233687021
ADPRH	2.669897005	LOC105609715	-3.228537571
SAMD4A	2.666208842	LOC105606524	-3.222452939
MICAL2	2.665482326	LOC105611989	-3.222452939
ADAMTS12	2.664627041	LOC114116353	-3.222452939
LOC105605321	2.661303199	F5	-3.222185153
LOC101115931	2.661303199	MYO15A	-3.219931279
LOC114112171	2.661303199	CTNNA2	-3.21208182
LOC101110922	2.661303199	SGPP2	-3.20893791
ARSB	2.657395067	GSTA1-1	-3.204814952
LRRC10B	2.656410108	LOC101117691	-3.204306935
CAMK1G	2.655677445	CHRNA3	-3.202906355
LAIR1	2.654352192	PLS1	-3.200581953
LOC114116423	2.653734168	MESP1	-3.197015016
SRPX	2.653475155	CHST11	-3.194107268
MAP2	2.651757737	LOC106991666	-3.194015852
KRT5	2.650495852	BHMT	-3.190798533
LOC101119773	2.647092371	FAM162B	-3.185043152
ATL1	2.646504858	LRRC38	-3.182561645
MEGF6	2.644961537	LOC105612366	-3.182561645
CXCR6	2.640827033	TNNC2	-3.182561645
LOC114113970	2.640287493	DBF4B	-3.177597409
LOC114117877	2.638033182	EEF1B2	-3.166509301
LOC101115787	2.634875357	HMGB3	-3.164137888
LOC114114463	2.633228979	SLC25A10	-3.159350324
LTB4R2	2.632971919	LOC105616340	-3.15496302

LOC101123290	2.630885731	LOC101123010	-3.154103181
LOC105616288	2.629956462	LOC114116205	-3.15327485
ST18	2.629956462	LOC114115731	-3.15327485
LOC105610651	2.629956462	LOC114115603	-3.15327485
LOC114116965	2.629956462	LOC114118689	-3.15327485
PNOC	2.629867943	MYBPC1	-3.144198239
LDB2	2.627418529	LOC114111030	-3.144198239
INPP4B	2.626912684	ZNF674	-3.143510571
LOC114109118	2.62584266	ACSBG1	-3.121252425
ANTXR1	2.625816302	LRRC2	-3.120623972
LOC105611362	2.625360438	UBE2QL1	-3.119205654
PRR22	2.625360438	GPRIN2	-3.116750847
EGR2	2.624221357	CCNI2	-3.113310059
LOC101111764	2.622996185	LOC105609364	-3.113310059
LOC105604270	2.622996185	CARD14	-3.113310059
SCN2B	2.622907289	DUSP9	-3.113310059
TPO	2.618953143	C16H5orf49	-3.110833908
LOC105604676	2.616831729	LOC101117785	-3.108258608
PPP2R2B	2.616311049	GJB4	-3.104786761
BGN	2.615962742	PHOX2A	-3.098340938
COL5A1	2.614655736	LOC101122645	-3.096092759
CPZ	2.613939486	NCKAP5	-3.094250977
LOC105603904	2.612802001	LOC114115267	-3.087927469
RAB7B	2.612733795	LOC114118467	-3.087927469
SHE	2.612448879	FOXO6	-3.084252029
LOC101121244	2.608713713	LOC114108805	-3.083955673
CX3CR1	2.606063009	LOC114109441	-3.083005469
ST6GAL1	2.605107612	LOC105614699	-3.083005469
MXRA5	2.603629348	NLRP2	-3.083005469
WDFY4	2.602517317	PCSK1	-3.077573637
CGREF1	2.602445232	MIF	-3.076715522
VAV3	2.601522445	FKBP6	-3.074974511
NLGN4X	2.600580909	LOC105606568	-3.069634471
ND3	2.594289522	RMI2	-3.066889223
GRID2	2.594193069	LOC101108413	-3.064268331
CLEC5A	2.594193069	LOC114109422	-3.064185545
TNFSF18	2.59333153	INPP5J	-3.054676147
DUXB	2.591451761	NUDT11	-3.052050633
ADGRD1	2.588317045	LOC105609012	-3.052050633
MAF	2.587219949	LOC105609925	-3.049282546
ITGA2	2.585366127	LOC114110481	-3.045280036
IRAK3	2.58506654	FAM222A	-3.042181657
LOC101105553	2.584490292	FAM57B	-3.038790012
LOC101108745	2.583644218	LOC114109344	-3.038790012
LOC114114519	2.583644218	SLC17A7	-3.038790012
IPCEF1	2.583644218	LOC101119591	-3.038790012

PNMT	2.583644218	MCEMP1	-3.038790012
LOC105603273	2.583644218	LOC114109578	-3.038790012
TNFRSF13B	2.583644218	SCG2	-3.038790012
LOC114116356	2.583254577	KRT36	-3.038790012
LOC114116412	2.583247754	LOC114111034	-3.038790012
ROR1	2.582682358	C8B	-3.038790012
SETBP1	2.580212236	LOC101112469	-3.038790012
LOC101101976	2.579498576	LOC101113487	-3.038160653
KANK1	2.577341428	FAM83G	-3.030285481
NCS1	2.575083251	FZD5	-3.029876262
LOC101121256	2.57420379	LOC106991659	-3.024945666
PTPN22	2.57420379	FAM169A	-3.022982896
LOC105604226	2.573203361	LOC101122753	-3.022578967
STUM	2.567787424	REC114	-3.022578967
AMZ1	2.563657741	LOC114114985	-3.022578967
LOC105604471	2.563246704	LOC114114490	-3.020811562
LOC114118385	2.563229821	LOC105602963	-3.02041703
CALN1	2.55765773	LOC105611804	-3.02041703
GCNT4	2.555420071	HK2	-3.019901598
ALDH1L2	2.554431132	KRT19	-3.017682847
MOCOS	2.553328166	LOC105612323	-3.006867724
FER1L5	2.552638	NLRP8	-3.000831272
NUP210L	2.552638	LOC101111541	-2.998999991
LOC101105731	2.552638	PTPRN2	-2.99563296
SLC39A2	2.552638	PDZD4	-2.988074227
LOC114115675	2.552638	PLAC1	-2.988074227
LOC114116118	2.552638	LOC114112700	-2.987889119
LAMA3	2.550105596	STYXL1	-2.987765662
ITGB4	2.549951214	LOC114113171	-2.979648971
ADCY7	2.549291353	LOC114108727	-2.976282037
ITGB3	2.549211525	LOC105611014	-2.974117969
WHRN	2.544036218	KLC3	-2.971976456
ADA	2.543621137	LRRC46	-2.966740023
OLFM1	2.543574648	PKMYT1	-2.966282149
OLFML2B	2.543403926	LOC105608946	-2.966020983
ITGB1BP2	2.543188688	LOC114110609	-2.962985302
LOC101105017	2.543188688	TRPV6	-2.954989692
RNF144B	2.539688872	LRRC73	-2.954469092
NIPAL4	2.538611732	MYO5B	-2.95343064
XYLT1	2.537814231	CRYBB3	-2.952242585
RCAN3	2.535724735	NGEF	-2.950119438
ELF4	2.535009082	PRR15	-2.948513424
EYA4	2.531275864	LOC114113257	-2.945127052
LOC105613245	2.524615142	BEGAIN	-2.93424355
PTPRC	2.524462772	FSHR	-2.930942909
LOC101114456	2.523793498	OIP5	-2.930839955

EVI2B	2.522514598	SLC35G1	-2.925176489
MILR1	2.522006993	ANKRD37	-2.92256188
JPH2	2.520530273	ANKS1B	-2.914431061
LOC101112726	2.520403038	LOC114108680	-2.914431061
MGP	2.520260229	LOC101104222	-2.914431061
SLC7A2	2.519694149	LOC105602877	-2.914431061
LOC114110650	2.519089717	FBXL16	-2.914431061
LOC114112840	2.519089717	LIG1	-2.90959644
FBLN5	2.510790321	RASSF7	-2.908421887
MYCBP2	2.509008514	LOC114109660	-2.908421887
RHOJ	2.507004646	MAL2	-2.906164603
EDNRB	2.505414422	LOC114114854	-2.902464954
ITPRID2	2.502307876	UBE2S	-2.90231934
LYPD6	2.502180281	LOC106990546	-2.901706351
LOC101122306	2.501800572	E2F2	-2.901680584
KLF13	2.501754696	PRR29	-2.900355448
RAPGEF5	2.501622187	INA	-2.896207408
CUBN	2.501565908	PAH	-2.896207408
TESPA1	2.501565908	LOC114109125	-2.895527901
SLC22A4	2.501565908	LOC114117509	-2.892360128
TJP3	2.501565908	ENO1	-2.890040968
LOC105604792	2.501099821	LOC114116606	-2.889755418
PTGS2	2.500486691	AP3B2	-2.889755418
LOC114111015	2.499141361	LOC114111311	-2.887412932
RNF24	2.496791608	MCM5	-2.870207112
LOC101114082	2.492259974	LOC101110185	-2.869377979
OXCT1	2.492228734	LOC101115605	-2.868272612
NPTXR	2.491551858	LOC114110420	-2.86550452
LOC105601852	2.491127205	CRHR2	-2.864911682
LOC105602828	2.490797993	GPR35	-2.861698966
CFAP54	2.490367046	NR1H4	-2.859295557
LOC101106330	2.489138952	CSRNP3	-2.857566661
LOC101103178	2.488759004	CHRNB2	-2.850924684
NEK1	2.48748905	GTF2A1L	-2.850924684
DUSP4	2.483981531	PAPSS2	-2.846447926
LOC101106166	2.483510311	CAMK2N2	-2.842617016
IL33	2.481225567	LOC105608303	-2.842617016
OSMR	2.480008379	TPH2	-2.842617016
LOC101122689	2.479292436	LOC105606413	-2.842617016
BMPER	2.47715212	KRTCAP3	-2.840972685
MMRN2	2.476240536	FZD9	-2.834496491
HGF	2.475856289	LOC105603181	-2.834450081
TMEM116	2.474275017	PMM1	-2.833545562
CADM3	2.471916483	LOC114118047	-2.831349102
LOC101102967	2.470939982	LOC114118700	-2.826187921
FCGR1A	2.469417736	LOC101110277	-2.826187921

LOC105608279	2.466612767	NMRK2	-2.826187921
ZNF333	2.465448776	SLC22A16	-2.826187921
CLEC6A	2.464896865	LOC114113588	-2.826187921
NKD1	2.463609562	DNAH2	-2.826187921
SYP	2.46219066	LOC114109532	-2.826187921
APOBEC2	2.460153114	LOC114112919	-2.824745075
STON2	2.458956479	CEP72	-2.822587574
TMEM252	2.458706516	ADAM11	-2.822098421
TNFSF10	2.457795174	EML5	-2.816938714
PRND	2.457289108	CDCA5	-2.816673315
LOC106991067	2.454459981	LOC105610279	-2.814498633
VSTM5	2.454459981	MSI1	-2.811846503
LOC114113240	2.453230497	DNAH9	-2.80528575
EPHA7	2.452574814	LOC105612338	-2.79717572
CACNB2	2.452164295	GGT6	-2.79717572
DOCK4	2.451278145	LOC105609938	-2.793886247
KCTD12	2.450870169	LOC101112093	-2.793886247
EHD3	2.450423696	MATN4	-2.793886247
PDE4C	2.450261238	LOC114117596	-2.793886247
SBSN	2.450124649	FOXP3	-2.793886247
CPEB4	2.449143447	CHRDL2	-2.793798348
SETD6	2.447479654	PKP2	-2.79211986
LOC101121563	2.447143677	CDCA7	-2.791134195
LOC101115808	2.444633961	POMC	-2.787593219
MYRIP	2.443881205	ERP27	-2.779120427
PLN	2.440591522	DIRAS2	-2.7783326
PECAM1	2.439412388	ERICH3	-2.7783326
PDE1A	2.435920834	LYPD6B	-2.7783326
LAT2	2.435580053	FAM227A	-2.7783326
CPXM2	2.43454762	RUFY4	-2.7783326
SNX10	2.433891199	PCDHAC1	-2.7783326
PQLC3	2.433098922	LOC114114024	-2.7783326
ALOX5	2.430439698	MS4A10	-2.7783326
LOC101121825	2.429992367	LOC105613571	-2.7783326
FAM180A	2.429017368	FAM221A	-2.7783326
XCL1	2.428644305	RADX	-2.7783326
MTMR11	2.427139409	LRRC4	-2.7783326
RASGRF2	2.42683188	LOC105606289	-2.7783326
LRRK2	2.426579449	LAMA1	-2.777424433
LOC101119638	2.426553974	KRT80	-2.777381242
PRSS35	2.426371007	PRRT1B	-2.777381242
PRRG4	2.424812646	LOC105610886	-2.777128999
MSRB3	2.421840593	COL6A6	-2.773498241
ACER2	2.419242787	LOC105605590	-2.76996389
PRKG1	2.41728655	SERPINA1	-2.768940032
CSRP2	2.416725044	LCN6	-2.766871131

IL1R1	2.415544532	GNB1L	-2.762970265
BATF	2.415003314	NTNG2	-2.761812693
LOC101123625	2.415003314	GNMT	-2.759945434
LOC105605117	2.414534776	C12H1orf100	-2.759945434
LOC114115664	2.414534776	PHF24	-2.755459781
SPAG17	2.414534776	PCBP3	-2.753890484
LOC105603539	2.414534776	DNAJC27	-2.753076121
MEOX2	2.412431676	STK32B	-2.75300482
THBS1	2.410935979	TFAP2E	-2.752596
LOC105603604	2.408347705	LOC105604110	-2.752596
NPY1R	2.407439918	LOC101102714	-2.752596
LOC105611518	2.407330675	LOC114114551	-2.749056693
IL1B	2.407330675	TOX2	-2.747768799
ZNF577	2.404878179	PHGDH	-2.744448347
CFAP206	2.404364033	RGS11	-2.743939156
LOC101107098	2.401858659	SH2D5	-2.743451753
TSKS	2.399635283	ESR2	-2.743451753
LOC101108705	2.398694558	BICDL1	-2.740016454
COL11A1	2.39807989	FAM166B	-2.735916294
GPR83	2.396737732	KLK1	-2.734483375
LRAT	2.396663007	KIF22	-2.729730046
FOXN2	2.39467056	TTN	-2.726925761
TFAP2A	2.394543255	KRT18	-2.723940797
GATA3	2.392609277	LOC114110058	-2.722820258
LOC101112639	2.389288226	ALDOC	-2.717062367
CLIC2	2.388697755	LOC106990346	-2.717061515
LOC105603436	2.388448397	LRP8	-2.71291769
FBN2	2.38577525	LOC114114548	-2.711204073
RGS18	2.384307328	LOC114112236	-2.709943331
RINL	2.384302996	LOC114114480	-2.707581088
CD3G	2.384299309	CNTD2	-2.707581088
LOC114114896	2.383478083	CATIP	-2.699354115
PDE3B	2.382318091	FBXO5	-2.696054767
PTPRJ	2.382073399	EFHC1	-2.69254855
CCDC88A	2.381357002	LOC114112899	-2.691465898
PKD1L3	2.378427061	ETNK2	-2.687886888
LOC101120001	2.37597512	MVD	-2.686961941
SHISAL1	2.372127958	CXXC4	-2.686899627
ALX1	2.371050936	SH2D4A	-2.686242466
LOC101108102	2.37046669	LOC101117129	-2.684215563
DAPP1	2.369812646	OTOA	-2.680668491
LOC101107486	2.368967776	LOC101118307	-2.677136502
LOC114118066	2.368967776	BNIP3	-2.676301423
FAM241A	2.368815691	SPACA4	-2.67504742
LPXN	2.368574691	LOC114110490	-2.67504742
LOC101103973	2.360614429	ADGRE2	-2.67504742

PDE4D	2.358520144	BMP5	-2.67504742
LOC105612674	2.358206456	LOC101110202	-2.67504742
PTHLH	2.357845911	OSGIN1	-2.67411615
PAK3	2.357713681	ACBD7	-2.671939888
PTCHD1	2.357377118	C3H2orf81	-2.671939888
LOC101119832	2.357377118	KCND3	-2.671939888
CNTNAP4	2.35632066	LOC101106395	-2.671882848
SHC4	2.35632066	LOC105605445	-2.6709257
THBS4	2.354342915	CLDN15	-2.67042257
ITGA5	2.351840752	TDRD10	-2.670092671
AKAP12	2.351704085	HDC	-2.658835685
ROCK1	2.351476539	LHB	-2.653562008
KLHL28	2.350949092	UPK1B	-2.651129881
LOC105604063	2.349455256	LOC106991576	-2.650808206
LOC101117971	2.349285679	RECQL4	-2.650424438
SULF2	2.349106854	LOC101108787	-2.648883736
GUCY1A1	2.348647921	CDT1	-2.643857277
CPE	2.3473202	LOC101117021	-2.636963828
CC2D2B	2.345246533	BCL2L10	-2.636963828
PDZRN4	2.343161788	VWA3A	-2.636963828
SYNPO2	2.342618499	SLC8A3	-2.636963828
KCNMA1	2.342155136	LOC105609379	-2.634384329
HOXB3	2.342086716	PTGES	-2.632840446
COL12A1	2.341896852	LOC114115236	-2.62804495
LOC101118514	2.3398702	LOC114116105	-2.62804495
THSD7A	2.338035576	LOC114115655	-2.62804495
MRVI1	2.337408857	DNAH7	-2.62570525
SMAD7	2.333517374	PALM3	-2.622042213
LOC114110119	2.33202989	LOC105611997	-2.617146321
GOLIM4	2.33172701	SLC15A1	-2.617146321
NCF1	2.331196562	ZNF875	-2.617146321
KCNT1	2.330160434	RPP25	-2.613770869
ARSJ	2.329455107	LOC105611533	-2.611766176
LACC1	2.325903549	LOC114113943	-2.610419878
TRMT9B	2.325577197	COCH	-2.607960728
LOC114115264	2.325241994	SMG9	-2.607948123
STK38L	2.324983386	SLITRK4	-2.602465683
ZNF554	2.321914486	CARMIL2	-2.601392894
LOC105603417	2.321914486	STRA6	-2.600494321
LOC101110403	2.321914486	TMEM233	-2.593092427
CCDC3	2.321302404	TFR2	-2.592050714
LST1	2.320612734	CLDN3	-2.590857696
SYNC	2.320485746	LOC105607520	-2.586521857
LPAR3	2.320322609	GLP2R	-2.583349409
POLK	2.31773913	PCDH20	-2.582173153
PLCB2	2.316301854	KMT5C	-2.580886721

APBB2	2.312790513	ELOVL4	-2.580745718
LOC105603695	2.312597792	LOC106991901	-2.58061952
TBX18	2.310361781	LOC105602434	-2.58061952
CXCR3	2.30989183	LOC114118361	-2.58061952
MCAM	2.309634368	LOC114114884	-2.58061952
LOC101119426	2.307423152	TFF2	-2.58061952
PLXNA4	2.304899567	RPGR	-2.579123445
NTRK2	2.304723849	MCM2	-2.576345778
RELN	2.304495274	ZYG11A	-2.574216325
RSPO1	2.302971826	TKTL1	-2.574206381
JAZF1	2.300652594	MAT1A	-2.570319293
FAM13B	2.300383836	NPAS3	-2.570319293
PIP5K1B	2.300251575	NEURL1	-2.568729896
GNG12	2.297874824	TLL2	-2.566220337
MUSTN1	2.297841941	FCGBP	-2.566127094
ANGPT1	2.297645557	LLGL2	-2.565495022
ACTN1	2.296988177	ST8SIA5	-2.56439445
TRIM36	2.293596507	KCNH1	-2.56439445
FOXC1	2.29357874	YBX2	-2.560748556
EDNRA	2.288656342	CDK7	-2.555504047
CAMK2G	2.28546678	XKRX	-2.544500161
LOC554335	2.285176593	ADM	-2.536881548
KIAA1549L	2.284459316	LOC114117260	-2.534790345
SPARC	2.283653867	SLC25A53	-2.531969342
KCNQ3	2.283511839	LOC114109026	-2.529969588
LOC114111673	2.281749042	RTL9	-2.529969588
DMD	2.281467333	RGS17	-2.529969588
LOC114117355	2.281437393	LOC114115365	-2.527709201
SMOC2	2.281024136	ASF1B	-2.525769257
TMEM156	2.27678722	CHRNA7	-2.524808308
GALNT17	2.275308822	LOC114116820	-2.521984942
CALCRL	2.274627509	LOC114118859	-2.521984942
GPC6	2.273461365	PITX3	-2.521984942
NTF3	2.272363229	FAM167A	-2.521984942
PLPP1	2.271752265	LOC105610222	-2.521984942
LOC105612707	2.27092978	RPL23A	-2.52141618
MCTP1	2.270891026	ZCWPW1	-2.51578832
MSX2	2.268879957	CA1_1	-2.506199936
RIMBP2	2.266684588	GPC2	-2.506199936
FAM84A	2.266180996	LOC105602343	-2.506199936
OGN	2.26576192	S100A3	-2.506199936
KCNE4	2.26431077	LOC114109042	-2.506199936
PTGIS	2.263432493	LOC106991069	-2.506199936
RPS6KA6	2.261778109	LOC114112904	-2.506199936
SEMA3D	2.258669518	LOC106991594	-2.506199936
OPRL1	2.258498369	INHBA	-2.504242927

LOC114109035	2.258237023	RIC3	-2.500473606
DSTN	2.257418449	REEP4	-2.500457003
GPR62	2.257207205	LOC114112176	-2.496338993
GLS	2.255788657	THOP1	-2.496334867
FXYD1	2.252749686	LOC105606461	-2.494914824
LOC114115224	2.251348785	PSD	-2.493074416
TEK	2.249589751	NPM3	-2.490519615
TSPAN2	2.248738345	LOC101106791	-2.488502266
HOXA3	2.248355366	RHCG	-2.487963248
LOC114110611	2.247609909	LOC114110160	-2.484322772
CCSER2	2.24725047	AURKB	-2.484070526
IL20RA	2.245431604	CDC6	-2.482604363
ADHFE1	2.244347976	LOC106991804	-2.480750955
PTPRZ1	2.242905105	ARHGAP22	-2.478705522
TGFB2	2.240661002	VEGFA	-2.478292108
SORCS2	2.239762777	LOC114115385	-2.475746598
NKAIN2	2.237872669	LOC105605507	-2.472794874
GABRG1	2.237872669	C5H19orf57	-2.47060217
LGALS12	2.237872669	KIF21B	-2.461445949
LOC114116676	2.237823909	LOC101108158	-2.460258876
ZC3HAV1L	2.234766678	SLC6A20	-2.457461847
LOC101108001	2.232867439	KIF12	-2.451839151
PRELP	2.231251665	LOC114117543	-2.4504372
SCN3B	2.22959181	LOC114116706	-2.449117083
LOC105614722	2.227768978	SMC1B	-2.449117083
LOC114116957	2.225155857	BSN	-2.446335306
PLEKHA6	2.224813394	BRINP2	-2.445518603
LOC101105840	2.222937597	LOC114118712	-2.444212566
LOC106991117	2.222937597	CYP11A1	-2.443084613
HOXA2	2.222937597	CHST13	-2.442049773
LOC105615501	2.222937597	GRM8	-2.441272278
LOC114110426	2.222937597	COL9A1	-2.439742549
LOC114117773	2.222589464	TICRR	-2.433291031
LOC101116756	2.22252609	BEX5	-2.427823705
ICOS	2.219474653	LOC114116631	-2.426646504
NHSL1	2.218156796	LOC105605730	-2.425958965
LOC105610529	2.21573675	HMOX1	-2.424792987
XCR1	2.214650721	TEDC2	-2.419419018
CASS4	2.214650721	ST3GAL4	-2.418532339
CYTIP	2.213492427	EYA2	-2.41625134
RAP1GAP2	2.213492427	LOC114110476	-2.414072823
LOC105604044	2.213113621	LOC106991640	-2.413736655
LOC105609509	2.213113621	C7H15orf61	-2.409182137
TGFBI	2.213013001	ADAM8	-2.403855758
FAR2	2.212890227	LOC114109326	-2.399894142
EOMES	2.211953864	CITED4	-2.399345462

SASH3	2.211333121	CCDC197	-2.397434728
PLCE1	2.210654303	TBPL2	-2.397042776
EEF1A1	2.209114537	KCNJ12	-2.397042776
ADAMTS13	2.209092625	ALX3	-2.397042776
LOC114118409	2.208208081	LOC105604036	-2.397042776
LOXL2	2.20413657	IL11	-2.397042776
PKIB	2.202028027	WWC1	-2.395738607
LOC114110128	2.200320469	ARG2	-2.393792901
TAGAP	2.199794048	TMEM198	-2.390421407
AVIL	2.199794048	OBSL1	-2.388872998
KDM5A	2.197857349	TIMELESS	-2.38829566
PCP4	2.197662502	UHRF1	-2.385263526
SLA	2.1968736	FOXP2	-2.38237379
LOC114112946	2.193008644	LOC114118000	-2.3822452
LOC101112084	2.192954117	BAIAP2L1	-2.381163854
LOC101106534	2.192458352	EXO1	-2.380967344
DST	2.192317576	MT3	-2.379696418
LCP2	2.186033385	LOC101116286	-2.375253225
LOC114118466	2.185560881	LOC114110487	-2.37249507
LRRC31	2.185560881	LOC114110812	-2.366296234
LOC106990881	2.185560881	TSPAN33	-2.362057059
NDST3	2.184329291	TG	-2.358362762
LOC105606280	2.184329291	LOC106990141	-2.356354894
LOC101116336	2.183395556	PDE6B	-2.352133526
LOC101113965	2.183332998	LOC105609492	-2.351081014
ITGA9	2.183071807	C11H17orf53	-2.350308437
LOC105609969	2.179641992	DSP	-2.346974601
DGKG	2.178319838	CNRIP1	-2.345992596
WDR47	2.177986584	LOC101111401	-2.34583533
FCHSD2	2.177932354	C2H2orf88	-2.338019107
XKR4	2.177845633	LOC101123533	-2.338019107
COL1A2	2.176229975	PUSL1	-2.332549164
KDM7A	2.174995018	LOC101102327	-2.331824132
UTRN	2.174399139	CPM	-2.331699481
LOC114114616	2.172184117	LOC106991772	-2.331139882
RBPMS2	2.171606549	CCDC38	-2.328037309
LOC114117866	2.17078052	SRGN	-2.322293098
LOC101102231	2.17078052	TREM1	-2.314939055
SYPL2	2.17062577	DPYSL5	-2.314939055
LOC114117972	2.168726721	LOC101106282	-2.314939055
AK5	2.165759032	C11H17orf50	-2.314939055
HIVEP2	2.162791123	TMEM92	-2.314939055
LOC114117779	2.162757964	CHRNA5	-2.314939055
SORBS2	2.161171513	INHBE	-2.314939055
ADRA1D	2.160111741	LOC114113250	-2.314939055
LOC114117808	2.15951715	SCAP	-2.311701708

HES4	2.159046431	SURF2	-2.310648532
LOC105607326	2.158643589	ASPHD2	-2.307448655
CLEC4A	2.156193448	LOC114116062	-2.301011982
PAQR6	2.156191761	SLC27A6	-2.301011982
BCAT1	2.153356882	PRKAA2	-2.299322651
CACNA1C	2.152512499	TMEM132A	-2.2992865
LOC105611136	2.152462473	CBARP	-2.297494583
PLD4	2.15225831	CCDC85C	-2.295290805
NRGN	2.150065329	NKAIN1	-2.294945596
ITGAV	2.148553565	HHIP	-2.294640174
CCR5	2.147514588	CDK5R2	-2.289039427
RHBDL3	2.146001207	LOC114116607	-2.28901852
LOC106991852	2.14567328	SBK1	-2.288920543
AEBP1	2.145184524	KRT3	-2.287240334
LOC101102310	2.144889415	PLEKHA7	-2.286013223
LOC114108849	2.144400502	LOC101111058	-2.28575743
PLCL1	2.143466903	E2F8	-2.284861536
ESM1	2.143185777	LOC105611500	-2.2825462
ITGA3	2.141853487	LOC101105609	-2.281483034
FRRS1	2.141157752	PLPPR2	-2.2803055
DUSP27	2.138510163	BCL11A	-2.279813893
SGIP1	2.137586632	AKR7A2	-2.276143159
SELP	2.137446982	CNIH2	-2.27563141
FHL1	2.137290065	NXNL2	-2.27563141
NLRC3	2.136640319	SELENOH	-2.274683819
TCP11L1	2.135039484	LOC114118731	-2.272892884
LOC101120574	2.134765157	UPK3B	-2.272892884
EMP1	2.133990441	ADAMTS7	-2.271336805
GANC	2.133503566	TK1	-2.268831872
LOC101109388	2.13293352	LOC114116623	-2.265712543
PLCG2	2.132249097	PVALB	-2.265138989
LOC101112038	2.132062397	LIN7B	-2.262500752
OBSCN	2.131280681	LOC105607692	-2.261575407
LOXHD1	2.131280681	PHKA2	-2.261421721
LOC114114539	2.128995566	FBXL21P	-2.260245394
RHOH	2.128420266	CCDC148	-2.260245394
CRYAB	2.128361747	LOC114109372	-2.260245394
COL8A2	2.127936415	LOC114117323	-2.260245394
PPFIBP1	2.127614463	CDC45	-2.260023487
RGS6	2.127342196	MSMO1	-2.259259044
CD84	2.127137631	PMEL	-2.258780694
SGCD	2.126473593	LOC114114502	-2.258081986
FAM131B	2.126416169	LOC114109428	-2.258081986
LOC105605337	2.126382756	PPP4R4	-2.256366912
LOC105615470	2.12476106	ENTPD2	-2.250875636
KLHL3	2.12264376	COL7A1	-2.250518459

STEAP1	2.121704928	LOC106991936	-2.249655699
PHLDB2	2.120539552	TUBA4A	-2.247271492
PROB1	2.120091419	TMEM120A	-2.242871185
STEAP2	2.119011266	LRFN1	-2.241903059
FCGR3A	2.118359325	ZNF536	-2.238872744
RNASE6	2.11833526	POLA2	-2.238819133
LOC114117554	2.11832025	CTXN1	-2.23714529
HLF	2.117910292	LOC114111040	-2.236581914
MAMDC2	2.117729587	SLC27A1	-2.23411281
LOC105603376	2.116666876	STAR	-2.232336655
UGT8	2.116666876	EIPR1	-2.232009923
LOC101108131	2.116603544	ARHGEF16	-2.231851455
NUAK1	2.115653653	MGAT5B	-2.231851455
ERBB3	2.113874833	LOC114110303	-2.231531182
TCAP	2.112654738	LOC105615212	-2.231531182
TNS1	2.109140616	LOC105610729	-2.231531182
FGL2	2.106985885	LOC106991287	-2.231531182
LOC101112356	2.106524834	PKIA	-2.231414617
APP	2.106236691	TUB	-2.231353622
SYT12	2.106189316	CDKN1A	-2.231144007
LOC101112584	2.104790389	APOA5	-2.230197773
GYG2	2.104351377	LOC105606686	-2.230197773
DEPTOR	2.10379716	ATP5MC1_1	-2.227419847
TRDMT1	2.103249236	LOC105611883	-2.225505917
MAOB	2.103204875	NR5A1	-2.224120196
SLC46A3	2.102748155	DCTPP1	-2.222929266
HAPLN3	2.102160403	LOC101105090	-2.218267759
ND5	2.098810349	PFKFB3	-2.216843087
RDH10	2.098623172	GAPDH	-2.216182285
TFPI2	2.098417669	TMCO2	-2.213835249
METRN	2.098254926	GNRH2	-2.212477219
COL28A1	2.098006274	ISG15	-2.211286015
LOC114118418	2.095730053	COL9A2	-2.210502251
LOC105613405	2.095550473	RUVBL1	-2.209543283
THY1	2.094919622	LOC105608705	-2.20951992
NOG	2.093548588	SCP2D1	-2.208875005
KIAA0408	2.092326273	LOC114109673	-2.208875005
LOC105612017	2.091432948	GRK1	-2.208875005
LOC114117625	2.090449257	LOC101122717	-2.207310426
PDK4	2.090258175	KIF1A	-2.206468547
CACNA2D1	2.08973093	CRELD2	-2.202881589
CARNS1	2.089389538	LOC105602107	-2.201835725
LOC101106844	2.089066631	DOCK3	-2.200840816
LOC114114031	2.089066631	CD320	-2.200697065
GPER1	2.089066631	RASAL1	-2.197915412
LOC105612881	2.08553169	EIF4EBP1	-2.197168935

PCGF5	2.085501737	TREM2	-2.196627691
ADCY5	2.083975106	STRADB	-2.194213239
LOC101121762	2.082682688	LOC105613573	-2.193163113
MFAP3L	2.080477347	PGP	-2.193054961
OLFML2A	2.079579407	LOC105602517	-2.190658855
PCMTD1	2.078685662	OIT3	-2.189307349
CD244	2.077309473	LOC101117419	-2.189295905
HTR1D	2.076584119	LOC105605231	-2.186653474
SKIL	2.076267255	RFC3	-2.18586925
FLNC	2.075053108	ROR2	-2.185411316
HEY1	2.074916647	NLRP6	-2.184875085
LOC114114565	2.074878122	SYT15	-2.181302587
MMP28	2.074550021	DNALI1	-2.179100816
BFSP1	2.073399465	SRM	-2.176543587
LOC105612575	2.072966896	LOC105602076	-2.175186874
QPRT	2.072885588	CDH4	-2.175186874
LOC101109747	2.070884134	DDC	-2.175186874
TENT5B	2.070012088	CACNG7	-2.173939819
LOC105616290	2.069897123	LOC105601929	-2.171478916
SYTL2	2.068413906	POPDC3	-2.171101223
LAMA4	2.066524061	TOMM40	-2.170889184
PMEPA1	2.066046473	GNLY	-2.170460912
C1QTNF5	2.060963118	GAMT	-2.169939532
LOC101103472	2.060415009	SRRM5	-2.168692227
LOC101116085	2.059024932	LOC101102735	-2.168188433
CD86	2.058865435	TSSK3	-2.168188433
LOC101114216	2.058072113	FAM186B	-2.165621061
LOC105605770	2.057834651	GJA1	-2.164077453
CRIM1	2.056881348	ATP6V1E2	-2.16347232
MTMR7	2.055883083	MYO15B	-2.160078578
PTAFR	2.054836702	DPF1	-2.160078578
LOC114110105	2.054661762	ISYNA1	-2.153581473
SEC14L4	2.053087095	XRCC3	-2.14529193
LOC114114863	2.050759616	EXOSC5	-2.14269997
PIWIL3	2.050759616	MAP1LC3C	-2.142414762
CRABP1	2.050446231	GRIN1	-2.14149294
RGS9	2.049366257	BMPR1B	-2.140860588
SLITRK5	2.049219711	MCM4	-2.133763944
MGAT4A	2.048241488	LOC114110430	-2.127081571
USF3	2.047034997	TM7SF2	-2.126715651
IL1RN	2.046785571	LOC114116930	-2.124131184
DAAM2	2.04627665	CACNA1E	-2.124060842
COL14A1	2.045828941	LOC114110477	-2.123981644
SKAP2	2.044011587	CFAP44	-2.121050032
LOC101107504	2.042316638	LOC114109729	-2.120714689
TMED8	2.042034551	SHMT2	-2.119132425

DUSP19	2.038278077	LOC114118406	-2.119048309
LOC106990281	2.037907832	LOC105611550	-2.116980278
LOC105602261	2.036795414	TMEM144	-2.116552296
LOC101120630	2.036605879	OLIG1	-2.116552296
KLHL42	2.036268598	LOC114110669	-2.116552296
TLCD2	2.034794115	SLC18A2	-2.113626617
CIDEB	2.034505512	LOC105604901	-2.113353115
C1QTNF6	2.03383795	AP1M2	-2.113353115
SETD7	2.029255535	LOC114114902	-2.113353115
MAPRE2	2.028191154	AKR1B1	-2.112365708
AFAP1	2.02782608	MRPL40	-2.112046578
RGS14	2.026178866	STARD10	-2.109888449
BMPR2	2.026078922	C14H19orf48	-2.109763088
KLF7	2.025109759	DHCR7	-2.10951404
SPARCL1	2.024150551	MORN2	-2.109340947
SCN2A	2.023815812	UCN2	-2.109104894
STIM2	2.022409739	LOC101103439	-2.109104894
HEPH	2.022109276	C6	-2.109104894
CD274	2.020979982	LOC114108718	-2.109104894
UTP14A	2.019953592	CYP2A6	-2.109104894
RNF168	2.019797579	C22H10orf90	-2.109104894
AOC1	2.01905695	LOC114117239	-2.109104894
ABCA4	2.017777318	LOC114114824	-2.109104894
KCNJ10	2.017777318	MCMDC2	-2.109104894
RBM24	2.017289091	LOC105602454	-2.109104894
ALDH1A3	2.017048943	IPO4	-2.106524142
F3	2.017045019	ARHGAP44	-2.105604872
S100A4	2.016244919	LOC105602212	-2.10440951
UBASH3A	2.016180458	CDCA8	-2.103932199
CREBL2	2.014643245	CHAF1A	-2.103873103
SLC1A4	2.014599069	BPNT1	-2.103613468
LOC114108830	2.014131712	LOC114118389	-2.103190428
LOC101109513	2.013533979	CENPT	-2.103050594
LOC101117786	2.011923375	TMEM177	-2.101843629
LOC114116396	2.01140765	ANGEL1	-2.101014421
MAS1	2.01140765	SGO1	-2.097103461
LOC114115866	2.01140765	MRPL55	-2.096977897
LOC105609992	2.01140765	LOC101111911	-2.096504614
GRIA4	2.01131701	HSPBP1	-2.095723086
COL4A6	2.010014722	ENTPD6	-2.094552215
TBC1D32	2.009237106	STAC3	-2.094390166
QPCT	2.007608472	SPATA22	-2.094390166
DHH	2.005812683	LOC114117580	-2.094390166
SPOCK2	2.005622546	CCKBR	-2.094390166
COBLL1	2.003556807	PEAK3	-2.094390166
ATP7A	2.003367747	ABCC6	-2.094390166

NEBL	2.003202438	GPX1	-2.092090399
LRCH1	2.002634853	MOK	-2.091542518
GPX3	2.002585395	ZFAND2A	-2.090213998
C1QTNF7	2.001971782	CMBL	-2.08995678
LOC105608435	2.001941192	DDX39A	-2.088614036
LOC114116440	2.001941192	LOC105607154	-2.088393117
LOC114112697	2.001941192	UCK2	-2.087163497
PDZD3	2.001941192	A4GALT	-2.086040262
LOC105609002	2.000624749	LMNB2	-2.085093367
PRDM10	2.000474893	CCDC134	-2.083757369
PLEKHG4	2.000417061	LOC114114526	-2.083542412
LOC101119648	2.000016412	LOC105609898	-2.082074742
FRMD7	1.998133914	PSMC3IP	-2.07915997
NLRP1	1.997268752	POLE	-2.079072304
FOS	1.996807801	PLK1	-2.076870936
P2RY6	1.996669822	WRAP73	-2.071413664
CDK14	1.996334053	SRRM3	-2.069798703
CDH19	1.99421752	NACA	-2.066890355
LOC114116177	1.993925162	LOC114114095	-2.066578884
WIPF1	1.993461485	PLEK	-2.064755559
CALD1	1.993083866	ANKRD9	-2.064377871
MET	1.99306841	CHST8	-2.063938852
VSTM2L	1.991702383	BLM	-2.063876581
FAM129C	1.990545341	CENPV	-2.063753276
ATP10A	1.990045566	RIMS4	-2.062421506
SEPT5	1.989315545	MND1	-2.061825782
LOC101123578	1.988982138	LOC114111193	-2.061436275
CCPG1	1.988229996	SEC14L2	-2.058363851
NELL2	1.98613903	TGFBR3L	-2.056509853
LOC101121570	1.986096044	TMEM229B	-2.056495952
ROCK2	1.984819659	LOC114115994	-2.056455225
TFEC	1.98432551	RYR3	-2.05601357
MMRN1	1.981210538	LOC101106024	-2.055433341
PEAK1	1.980921894	LOC101120838	-2.055433341
PDCD4	1.980569654	CNMD	-2.055231623
LOC105604155	1.979502814	TRAPPC6A	-2.054225282
DKK3	1.978554494	TMEM37	-2.051794942
PDLIM5	1.978435314	LAPTM4B	-2.051793595
B4GALT1	1.976809292	PRKCG	-2.051791637
PLEKHA1	1.97637322	PEX16	-2.051263316
ZNF461	1.972641411	PCYT2	-2.049147612
CD24	1.97049532	TMEFF1	-2.047884485
IMPG2	1.969830768	NDOR1	-2.045947842
CLIC4	1.968047177	SKA3	-2.045588326
FAM160B1	1.964854192	HES7	-2.044298833
BANK1	1.962933263	LOC101114167	-2.044298833

LOC105613357	1.962189524	LOC114109091	-2.043333628
STAT3	1.961889116	LUZP6	-2.041751759
CD74	1.959959338	MXD3	-2.041489719
TMEM245	1.959771088	BUD23	-2.035363585
LOC114113122	1.958337419	TMEM54	-2.034161782
NOTCH3	1.956759001	LOC105612888	-2.033800885
FBLIM1	1.955734614	ABO	-2.033599557
DIAPH2	1.955226488	SPATS1	-2.032548335
LOC114116588	1.954992953	POLD1	-2.030558431
ASB4	1.954992953	B3GALT1	-2.030316578
LOC105606473	1.954992953	CELSR2	-2.029567692
CACNA1B	1.954992953	LOC114113979	-2.0271499
PHC3	1.953043034	LOC105603051	-2.0271499
LOC114114564	1.952107094	MCM3	-2.026505141
ABHD3	1.949646457	SHISA2	-2.025743235
MECOM	1.949031114	LOC105610397	-2.024869871
TACC1	1.943966006	HIPK4	-2.021884769
NUDT4	1.943933328	GRM4	-2.021884769
DYRK3	1.943243855	LRP2	-2.021884769
PDGFD	1.942764117	HAS1	-2.021884769
NIPAL2	1.942725366	ELOVL6	-2.019038711
CD302	1.941129057	FDPS	-2.018793141
KLHL30	1.940965254	ROPN1L	-2.018300475
LOC105606370	1.940599138	PEMT	-2.017427579
CAV1	1.940011972	RDM1	-2.015161546
PPP1R12A	1.939359606	SLC16A1	-2.012316924
LOC106990149	1.938710194	CATSPER2	-2.010930056
NHS	1.937482825	LRRC8D	-2.010926722
EML1	1.935039049	STMN1	-2.010093793
MATK	1.932606504	SNRPA	-2.009583536
LOC101111868	1.932517477	SERPINA14	-2.009264468
KLF9	1.930625997	MISP3	-2.009264468
LOC114110142	1.929674332	GPR137C	-2.008477753
LOC101104568	1.92932934	VWCE	-2.006297361
MYBL1	1.929326236	PPM1J	-2.006297361
LOC101109492	1.92910809	PRKAG3	-2.004932751
NR3C2	1.928932741	SPC25	-2.002722425
CCL21	1.92882478	LOC114118072	-2.002261895
TAGLN	1.926619493	LOC114110578	-1.998923052
ADAMTS5	1.925910713	ADGRG1	-1.997501785
MBNL3	1.925469743	TRAP1	-1.996429865
TNK1	1.925005182	KRT7	-1.993673238
MYL9	1.924218172	GRIK3	-1.99161013
RCAN2	1.924146918	KIAA1257	-1.99161013
MYH11	1.922836914	LOC114110462	-1.99161013
CNST	1.922581693	LOC105615257	-1.99161013

PTGR2	1.922254875	LOC105602706	-1.99161013
LOC101123120	1.920800385	UBE2C	-1.989657191
ENDOD1	1.920566638	LOC106991047	-1.989550931
LAMA5	1.919867802	LOC114118856	-1.989217727
LOC101103090	1.919801456	CERS4	-1.984863636
LOC101104012	1.919690941	CMSS1	-1.984842919
LOC101123612	1.919660532	PRKAR2B	-1.984734988
LPP	1.919444582	RPL18A	-1.983557519
LOC114113971	1.915656707	LOC105615393	-1.98188577
ADAMTS18	1.915429542	LOC114116891	-1.98159317
LOC101104297	1.914353793	MGMT	-1.978848624
PTGS1	1.909248497	TVP23B	-1.976721678
COL22A1	1.909035667	SKA1	-1.975944272
PLEKHA2	1.908781344	CRMP1	-1.974132656
FAM217B	1.907881643	VPS37D	-1.972749311
ARL5A	1.905479739	GTSE1	-1.971366591
COL6A3	1.904761968	NUP205	-1.9704381
PENK	1.904350877	PCK1	-1.970031216
LOC101120326	1.903943446	SHC2	-1.970005504
OSBPL3	1.903678769	LOC114118506	-1.969623091
TNFRSF11B	1.902954462	LOC101107475	-1.969469723
TGFBR2	1.902580038	FBXO17	-1.966752358
CSGALNACT2	1.901144204	HOPX	-1.966415229
TES	1.899419128	MARCKSL1	-1.963334275
LOC101106372	1.899017796	DNASE1L2	-1.961977055
LOC114109079	1.89808206	KIFC1	-1.961534829
F13A1	1.897456091	TECR	-1.960823652
FBXL20	1.897349046	B3GALT6	-1.959264095
IGSF10	1.897068615	SAPCD2	-1.959080995
YPEL2	1.896702366	TROAP	-1.958820186
DMPK	1.896317483	YDJC	-1.958352353
CACNA1G	1.896098594	LOC101105400	-1.954676314
LOC101122496	1.895746392	APBA2	-1.954676314
PLVAP	1.895569925	NUDT16L1	-1.953904598
CD4	1.895362623	ALDOA	-1.9519779
LIMA1	1.89521997	SCRN2	-1.950135717
SCIMP	1.89388265	RUNDC3A	-1.949843175
LOC114116895	1.891094987	RRP9	-1.947100991
ZNF157	1.891094987	FAAH	-1.946883765
LOC106991039	1.890772828	GPX4	-1.946881228
LOC114110571	1.890562501	TRIB2	-1.945503982
LOC101114959	1.890201521	DTYMK	-1.945118069
ESR1	1.889943254	ECI1	-1.944743877
PGM5	1.889420238	TMEM97	-1.942134535
VIPR1	1.888996041	TRIP13	-1.941542777
TFPI	1.888528972	FAM234B	-1.941420238

TRPM6	1.888041623	LOC114114570	-1.940982013
LOC105608393	1.88694037	SERPINB12	-1.940257409
MBNL1	1.885169569	LOC105603310	-1.940257409
C15H11orf96	1.884918922	HSD17B14	-1.940257409
PPIC	1.882031928	CLCN1	-1.940257409
LOC114116407	1.881624707	LINGO2	-1.940257409
SHOX2	1.881240853	LOC101120174	-1.940257409
LOC114114549	1.878044229	LOC114116191	-1.938186441
ZMAT1	1.877297981	KCNF1	-1.93652226
TMEM169	1.877297981	DUSP26	-1.936464897
LOC114116378	1.877297981	LOC105609026	-1.935656856
CDRT1	1.877297981	ENDOG	-1.935365762
LOC114117632	1.877297981	ZMYND10	-1.934570923
ARHGAP24	1.876643578	GPR68	-1.934570923
HOXA6	1.87641126	MVK	-1.934359312
LOC101111623	1.876304179	LOC114113887	-1.930853244
ANKRD50	1.875870976	PLIN5	-1.926968722
LOC105605611	1.875850772	SLCO3A1	-1.926961536
PRNP	1.874511597	SPATA21	-1.924832235
PLCB1	1.874439031	CATSPER3	-1.924832235
PPP1R1B	1.874046292	LOC105610832	-1.922617813
NPR1	1.873512456	MSH4	-1.921163174
TUBE1	1.871752098	ABHD11	-1.919517531
ALDH1A2	1.871560866	DEGS2	-1.916251937
SEMA3F	1.871396466	FA2H	-1.915396843
LOC101114551	1.871195053	ZPBP2	-1.915396843
CLOCK	1.870712107	PSD2	-1.915396843
SLC3A1	1.869467395	KCNK7	-1.915396843
KIAA0040	1.869291761	CDC20	-1.912565468
RASGRP4	1.867299079	NOCT	-1.912340311
TRPC6	1.867188628	EMG1	-1.911527014
ARRB1	1.867004294	NRTN	-1.911283655
UEVLD	1.865637453	PRDX1	-1.910136297
PLOD2	1.864881315	KLHL25	-1.909365586
DNAL1	1.864614381	ISLR2	-1.908762779
TBC1D9	1.863200318	ADI1	-1.908388338
HOXD11	1.860416064	LOC114112996	-1.907507498
ID4	1.860096491	RANBP1	-1.905072736
ANKS4B	1.859654007	GCAT	-1.905067676
LOC105603814	1.859464334	LOC114111217	-1.904443001
C3H12orf60	1.859193954	LOC105616822	-1.903474114
LOC105613784	1.858052399	PSMB3	-1.902860011
LOC101109733	1.858052399	CCDC85A	-1.901943218
SAV1	1.856585217	DAPL1	-1.900981883
TINAGL1	1.853790132	MARVELD3	-1.900981883
KCNB1	1.853233434	RHBDD1	-1.89936177

GTF3C4	1.851665606	LOC101103967	-1.898277201
P4HA3	1.85160471	VAX2	-1.898277201
BATF2	1.851463742	ENO2	-1.89644791
CDK17	1.85009103	CPS1	-1.896105399
MARCH3	1.848617671	CCNF	-1.894162434
SLC51B	1.847903214	ACCSL	-1.893791166
DAAM1	1.847590766	MTG1	-1.893251551
HEY2	1.846359448	LOC114117644	-1.892193128
GABRA2	1.846128252	LOC106991653	-1.892193128
LOC105615521	1.846128252	LOC114114826	-1.891571677
CPNE8	1.845010805	ZNF365	-1.889505561
DIXDC1	1.844813948	MRPS18B	-1.889324614
CD22	1.844474298	LOC114110282	-1.889189648
ITGB1	1.843726844	GAL3ST1	-1.88872422
SH2D4B	1.842298208	IQCG	-1.885338633
SCAI	1.842298208	PCBD1	-1.883224837
CCDC7	1.842298208	LOC105612127	-1.882914279
C11H17orf67	1.842298208	GINS1	-1.879701527
NHSL2	1.842238019	E2F1	-1.874196023
CLIP1	1.840585005	DEPP1	-1.874097346
PILRA	1.840321824	ALYREF	-1.870745077
LOC105607239	1.839871061	FSD1	-1.87005487
OMD	1.839018316	LOC114109536	-1.867597353
SERPINI1	1.838885076	LOC105608370	-1.867597353
C5AR1	1.838884563	NTMT1	-1.866347997
KLHL24	1.838088273	SLC5A6	-1.865850244
STX1B	1.835789289	NOP56	-1.865839964
LOC114113898	1.834184154	LOC101111394	-1.865452935
TLR7	1.834053594	LOC101117087	-1.864484002
LOC114114004	1.834053594	LOC105607734	-1.863924572
STAC	1.834021441	CHTF18	-1.862388595
SLC4A8	1.833395252	LOC106991502	-1.860560574
COL18A1	1.8333198	SLC16A9	-1.86029791
LOC105606919	1.832474488	SLC39A5	-1.859052536
ATP2B3	1.83083235	SLC41A3	-1.858052485
JCAD	1.83000686	AURKA	-1.857006784
VAV1	1.829306552	LOC105607546	-1.856312798
PGAP1	1.828979596	LOC101111922	-1.856142108
ITGA4	1.828309386	SSPO	-1.854812748
CHP2	1.825709506	LOC105604106	-1.854812748
LOC114110944	1.825531452	F2RL3	-1.854812748
CDC37L1	1.82535339	FITM2	-1.854446688
LOC101122014	1.824220925	LOC114112936	-1.854229179
TRPV4	1.824214829	TMEM151B	-1.85324822
LOC114114000	1.82421327	RPS29	-1.853021476
LOC114110969	1.824212643	LOC105603203	-1.851952101

LOC101108887	1.823489751	NSD2	-1.851315143
PALLD	1.822931568	ACOT7	-1.848872002
HOXD3	1.821692232	LOC105605798	-1.847675583
PROS1	1.821515526	GLDC	-1.846371072
LOC105605988	1.820285848	GATA4	-1.842999824
FRK	1.820285848	EXOSC4	-1.842394037
LOC101115905	1.81971267	RHBDL2	-1.841958769
SKAP1	1.819640744	LIPT1	-1.838706183
HSPA12A	1.818748832	C1H1orf194	-1.838514605
EFNA5	1.817817781	KATNB1	-1.837650233
HTRA1	1.817540237	PIH1D3	-1.836834074
ACTA2	1.815701043	EXOSC6	-1.834953561
LOC105611082	1.81431246	CYTL1	-1.83423217
PDCD1LG2	1.81385113	PFKL	-1.831932539
MAFB	1.813706061	TACC3	-1.830687397
CCNYL1	1.811372679	ROBO2	-1.829904312
CRISPLD2	1.811017645	GPI	-1.829741182
MB	1.810545625	BUB1	-1.829556022
LOC114114527	1.810309305	EBF3	-1.82800765
COL1A1	1.810055196	SH2B2	-1.827176148
IRF5	1.809817701	MRPS2	-1.826767974
LOC101120395	1.809372165	WDR5	-1.82650688
UBE2H	1.808721894	GABRB2	-1.826486066
LOC105608991	1.808578017	LOC114109018	-1.826273561
LRP6	1.807863424	FAM169B	-1.824901009
C3H2orf40	1.807639432	LOC114117637	-1.824901009
CAST	1.807177739	DBF4	-1.821819017
ABCC3	1.80655493	CARS2	-1.816928877
RECK	1.806148602	TMC4	-1.816726407
SHPRH	1.805606285	CKS2	-1.81641051
ARHGAP30	1.804678278	LOC101120455	-1.816363941
SNX18	1.804497013	MPV17L2	-1.816001435
NEGR1	1.8035824	SUSD4	-1.815971122
SLC2A12	1.801451656	CCNB2	-1.81531195
SOX6	1.801427883	FAM110B	-1.814938211
GJA4	1.7974783	RAD51AP1	-1.813606693
AIF1L	1.797045181	WNT11	-1.813351431
USP43	1.796731207	TRAIP	-1.813052235
LOC114111361	1.796731207	TCEA3	-1.811346362
L1CAM	1.796495207	PPCDC	-1.809377982
GIPC2	1.796398312	KCNIP1	-1.809078753
JAG1	1.796038424	RUVBL2	-1.808854569
CCL5	1.795701825	CNTN4	-1.808275382
SLCO2A1	1.794639084	LHPP	-1.80737164
CELSR1	1.793854073	C2CD4B	-1.806960771
FGFR3	1.793452836	PTCH2	-1.806464046

LOC114109610	1.793248516	LOC114110579	-1.806018656
CORO2A	1.792671923	PHETA1	-1.805985171
LOC114111468	1.792540935	NAA38	-1.805905439
ABCA6	1.792418042	CENPW	-1.805848316
F8	1.792259758	ABCD1	-1.804842286
LOC105603639	1.789641216	ORC6	-1.803463163
LOC101110257	1.789606352	SLC5A2	-1.803272308
LOC114114546	1.789606352	GINS4	-1.802478667
PLA2R1	1.789455538	KCNA4	-1.80121626
TRAF5	1.787873816	CEP78	-1.80087664
DNAJB4	1.787563705	NOB1	-1.800536261
HSPA2	1.786246883	LOC114116101	-1.800417958
LOC105606290	1.786134916	LOC114110643	-1.800417958
PTPRR	1.785921669	DEUP1	-1.800417958
ARNT	1.785802634	PLA2G2F	-1.800417958
FAM185A	1.785484347	SLC25A13	-1.799763506
LOC106990140	1.78507257	RTN1	-1.799689224
ABLIM2	1.785044512	STK32C	-1.798127196
LOC114116381	1.7848259	DDR1	-1.795452161
KLHL20	1.784692429	DGKD	-1.793400394
SMIM14	1.783500978	LOC101112491	-1.790522554
HYKK	1.783190117	LOC101116059	-1.790287796
CLDND2	1.783090743	LOC105607560	-1.789463527
HEATR5A	1.782709486	PGLS	-1.785500781
LOC114114015	1.78259618	FANCD2	-1.783629378
TPPP	1.781739131	LOC114109085	-1.782734979
IFIT1	1.781496976	KIF2C	-1.782085031
THEMIS2	1.781048974	CGN	-1.781289823
TAFA5	1.780299274	MLXIPL	-1.781220828
HS2ST1	1.779857928	LOC101114535	-1.780005922
F2RL2	1.779857928	FIGNL1	-1.776947903
LGALSL	1.777508917	PEX7	-1.776753553
EPSTI1	1.777126988	CCND2	-1.775682485
FNIP1	1.776576043	LOC114115318	-1.775425436
CAPN3	1.775376538	SQLE	-1.775334564
SLC35F1	1.774962091	GPNMB	-1.774943659
TENT5A	1.774529251	GPSM1	-1.774428522
RUBCNL	1.774000845	TRAF4	-1.771224082
ANK1	1.773387276	WDYHV1	-1.769951028
ZNF185	1.771515462	LAMC2	-1.769679904
OVAR-DRB1	1.768383467	NDUFA4L2	-1.768059403
SLC4A7	1.76707011	ZMAT4	-1.767355173
DPY19L4	1.766388696	TAFA4	-1.763547529
RASAL2	1.766185447	SLC27A4	-1.763229323
CAV2	1.765906498	LOC105613348	-1.762725834
RDH5	1.765682678	LOC105605165	-1.762725834

LOC105607593	1.765036062	KLHDC4	-1.760640977
LOC101106719	1.764398805	B4GALNT4	-1.759507465
SSTR2	1.763744452	LOC114112674	-1.758123114
SLMAP	1.763732928	EZH2	-1.757507928
ZEB1	1.761374933	MAPK13	-1.757098242
CDKL5	1.760846213	LOC114110821	-1.756982333
IGF2R	1.760500321	CHAF1B	-1.756804514
MX2	1.760396353	LOC105603432	-1.755534187
FKBP7	1.759396361	TOR2A	-1.755335964
LOC101115927	1.759173169	ZMYND19	-1.755211732
LOC101123244	1.758581404	ZFPM1	-1.754318933
BTK	1.75815505	CD226	-1.753293845
PHACTR2	1.75748851	DOHH	-1.752705821
LOC114112948	1.757157347	LOC114118505	-1.752580797
GALNT5	1.756884796	SCARB1	-1.752533589
LOC114110806	1.756806909	RABL6	-1.752176406
CH25H	1.756394166	SLC30A2	-1.751744155
LOC105603536	1.755614139	MTCP1	-1.748996528
LOC114110574	1.755614139	LOC101112434	-1.748996528
TMEM61	1.753905169	TBX15	-1.748996528
PRR16	1.753245245	TMEM212	-1.748996528
IL7R	1.751241061	LOC114113146	-1.748996528
LMBRD2	1.75075594	GFRA4	-1.748996528
THBD	1.750696435	LOC101109422	-1.748996528
NFAT5	1.75023997	LOC101116841	-1.748996528
GCGR	1.749677918	MYRFL	-1.748996528
NAB1	1.749480472	PSRC1	-1.746149231
PAG1	1.74867078	PRR5	-1.745947523
LOC105608443	1.748329146	NUDT8	-1.744755104
ST6GALNAC2	1.748295654	GNA14	-1.743824198
ANLN	1.747573166	EEF2KMT	-1.742542885
FBXO32	1.747568956	LOC114117767	-1.742423219
NRXN1	1.747370975	NETO2	-1.74098065
KCNJ3	1.746332899	LOC114117970	-1.739299003
LOC114112473	1.746138119	IL1RAPL1	-1.739299003
MID2	1.746093671	TIMM17B	-1.73810772
TAL1	1.745735188	LRFN5	-1.736962722
LOC105607466	1.74550352	GUCA1A	-1.736583151
FAM43B	1.744002396	WDR34	-1.735526152
TCP11L2	1.741458742	LOC101116828	-1.735058474
SYTL3	1.73876974	RAB15	-1.734736342
SMOC1	1.738737463	KCNK2	-1.734016043
PCOLCE	1.738663676	MRPL12	-1.733725715
NTNG1	1.73813387	MACROD1	-1.733129202
CASQ1	1.735952342	SESN2	-1.732398554
MARCH1	1.734554457	IGFN1	-1.731158314

BCAS1	1.734418481	IGF2BP3	-1.731158314
PALMD	1.733707074	PSMB9	-1.729735012
CHRNE	1.732553195	AMACR	-1.729308951
CYBB	1.731349701	LOC114110581	-1.729269398
RXRG	1.7298064	DCPS	-1.728295409
GFRA2	1.729377856	GFER	-1.725321177
ATP2A3	1.729225915	LOC105611240	-1.723810862
LYST	1.728397366	TAZ	-1.722474719
TAC3	1.727733857	COQ6	-1.721733174
TRAF1	1.727707734	MCOLN1	-1.721228262
SYNPO	1.725849416	ZBTB45	-1.72051042
LOC101112990	1.725779519	EEF1D	-1.72041014
B3GNT7	1.72450523	HPDL	-1.719037804
RETN	1.724395826	MELK	-1.718390669
HCN3	1.723311424	LOC114114137	-1.716420228
DSG2	1.723311424	LNX2	-1.716326394
PCP4L1	1.7184581	CCDC34	-1.71613526
MADCAM1	1.718161306	NME3	-1.715411594
LOC114113871	1.718161306	STX1A	-1.713882367
ARL4D	1.716765729	LOC105605439	-1.710464156
LOC101122718	1.716731329	LOC105610613	-1.710464156
HCRTR1	1.716483589	LOC105607506	-1.710464156
SOX5	1.716195119	APRT	-1.710278828
CDC42BPA	1.714271849	TMEM176B	-1.709902527
EGFL8	1.7139874	TONSL	-1.707489757
PRRX2	1.713317891	LOC105616404	-1.70569452
ANO6	1.712856483	EFHC2	-1.705484321
GNB4	1.710797455	HDAC10	-1.705229419
SGK1	1.710667771	NXT1	-1.704866919
LOC114114846	1.710593613	CSNK1G2	-1.704589243
VLDLR	1.710442972	LOC114110838	-1.703672248
CSGALNACT1	1.710418249	SMPDL3B	-1.703672248
CD59	1.710375335	PITPNM3	-1.703672248
LOC114117609	1.710112629	LOC101123403	-1.703672248
SYN1	1.709993891	WEE2	-1.703672248
LOC114117268	1.709825204	PRMT5	-1.703568646
DES	1.709357671	EEFSEC	-1.703271121
EFNB2	1.709162751	RASD2	-1.703193996
ZFC3H1	1.708995825	DHODH	-1.702586453
CTSW	1.708559193	MRPL38	-1.702216026
PLP1	1.707606789	CDC25A	-1.701252342
PTGES3L	1.706955424	FBL	-1.70101725
LOC114113048	1.705819362	LOC101117955	-1.700785932
WDR66	1.705819362	LOC114110474	-1.699243476
CHRM3	1.705819362	LOC101108147	-1.699176452
CCDC171	1.705325463	LOC105603708	-1.699176452

LOC101102642	1.704938788	SURF6	-1.699119793
LOC101118645	1.704506373	ODC1	-1.698223416
CCDC186	1.704334875	TSSK6	-1.697151252
DLL1	1.702832352	TRMT1	-1.696811887
PROCR	1.701613928	C13H20orf27	-1.696132874
LOC101123376	1.700720632	CFAP45	-1.695255532
LOC114114830	1.699942591	C4BPA	-1.695255532
LOC114109386	1.698109244	LOC114115334	-1.695255532
LOC101121769	1.698109244	MFSD2A	-1.694868593
CASK	1.697941999	WDR91	-1.694101623
PGF	1.69741245	LPAR2	-1.693049768
SYK	1.696358205	KRT8	-1.691543494
TUBA8	1.696129966	ADRA2B	-1.68975983
TLL1	1.69586229	KCNJ4	-1.689411658
LOC101108113	1.695858926	LOC114112112	-1.689411658
LGR4	1.695250285	MOCS1	-1.68935596
DPH6	1.694955535	CPEB1	-1.688224796
HPGD	1.690788285	LOC101111035	-1.688103627
LTBP1	1.690515131	LOC101103726	-1.687690876
AFAP1L2	1.69050877	NXPH3	-1.68761682
LOC101112543	1.69031159	RGS9BP	-1.686324067
LOC114113838	1.690268837	LRFN2	-1.686324067
G6PC	1.687635178	TTLL4	-1.685752914
IGFBP7	1.687055427	LOC114116722	-1.684463201
SP7	1.686830402	CHCHD4	-1.684131449
CLEC7A_1	1.686295894	LOC105603710	-1.683597673
AHR	1.685973948	ELOVL2	-1.683597673
ACTG2	1.685570908	LRRC3B	-1.683143765
FILIP1L	1.684610004	EPN1	-1.682940442
THBS2	1.683908757	SYTL1	-1.68163468
RAB3B	1.683714659	SCAND1	-1.68154975
PSTPIP2	1.683361662	KCNH2	-1.681193463
CCDC88C	1.683087191	DGKI	-1.680440485
SPEG	1.682491804	TAMM41	-1.680371509
PDLIM7	1.682329514	ASXL3	-1.67774224
SLC20A2	1.682223608	PMPCA	-1.675769161
ERG	1.682080697	LOC105616860	-1.675475791
DYNC1LI2	1.681925295	LOC114115351	-1.675475791
GPR146	1.681687252	DCDC1	-1.675475791
MYLK	1.681265481	KIF5A	-1.675475791
BROX	1.681008399	FOXA3	-1.675475791
ASB12	1.680434442	ATPAF2	-1.674879767
LMO7	1.679548122	LOC114110055	-1.674866065
XIRP1	1.679288373	FGF9	-1.674755698
KLHL14	1.679288373	MRPL28	-1.674484951
UACA	1.677041656	ABAT	-1.673516317

CERCAM	1.67655499	LOC101115115	-1.673226497
LOC101117657	1.676497758	C13H20orf96	-1.67317018
LOC101122475	1.674880672	ALDH4A1	-1.672301475
SNTA1	1.674346329	SLC2A1	-1.669620687
CPT1C	1.673454168	NT5DC2	-1.669495564
AKAP9	1.673105151	LOC105602007	-1.669409504
WDR17	1.670751401	DDX11	-1.669356282
LOC101113728	1.669942747	LOC101114310	-1.668953594
RAPGEF6	1.669784	LSM4	-1.668608319
LOC105609220	1.669146625	PSTK	-1.6675059
TULP4	1.668279718	CAMSAP3	-1.665885994
LOC101112168	1.667975693	NOP14	-1.665665222
LOC114109442	1.667734221	STAG3	-1.665238989
LOC105613298	1.667734221	IFRD2	-1.66497899
LOC114108820	1.667472945	LOC114114091	-1.664787168
RARB	1.667400372	LOC114114903	-1.6643341
LOC114114763	1.667022717	LOC105608597	-1.6643341
PTPRE	1.666022834	C6H4orf48	-1.660618795
PARPBP	1.66516318	TKT	-1.659646891
MEIS1	1.664958889	TSSC4	-1.65811197
PCDH19	1.664548315	SHD	-1.657731411
LOC114109713	1.664156382	JMJD7	-1.654917058
LOC101114469	1.663438203	METTL27	-1.654565228
GDAP1	1.662716357	TIGD6	-1.654165617
ADAMTSL2	1.662614107	LOC101105860	-1.649033211
PDE4B	1.661582856	SYT2	-1.644918458
SIRPB2	1.661577762	LOC106991803	-1.6438658
LOC101116845	1.66092504	PAXX	-1.643560502
SCARF1	1.660677639	CLEC19A	-1.641953747
ZBTB32	1.659344044	PDSS1	-1.641808472
SOX8	1.656573863	TPI1	-1.641712269
IL1RL1	1.656451688	KIF4A	-1.637050647
FYB1	1.655857642	RPP21	-1.634299644
CYLD	1.654704499	PGBD5	-1.633397205
KIF25	1.653226075	LOC114114101	-1.631674573
CGNL1	1.652690478	LOC114109437	-1.631674573
TMEM68	1.651426115	CA13	-1.631674573
RFX5	1.651276897	BIN1	-1.631560334
IL12RB2	1.650701029	LOC114113893	-1.630882362
SLC44A5	1.650701029	FAM181B	-1.630702036
CCL22	1.650701029	PABPC4	-1.630199614
MAP3K20	1.650579759	AHCY	-1.629979278
EPAS1	1.649637603	RFC2	-1.628448489
CRYBG1	1.648852342	MYC	-1.627975896
LOC101118600	1.64861018	MKI67	-1.627641912
ALKAL2	1.64861018	STAMBPL1	-1.627538049

CD200R1	1.648120571	CLCN2	-1.625665308
TTC32	1.647902098	CBLN2	-1.623355567
PTN	1.647150687	NNAT	-1.621193507
MRGPRF	1.646913544	PAGR1	-1.621148117
LEPROT	1.646514092	LOC105614707	-1.620738815
LOC114115302	1.646225845	SPR	-1.620150088
PWWP2B	1.646022148	LOC101121054	-1.619743107
FRMD4A	1.645711022	RRP1	-1.617830403
LOC114114073	1.645005351	BLVRB	-1.617724135
COL20A1	1.645003645	MEIOC	-1.61665205
TF	1.644646782	CSKMT	-1.615999045
LOC101105484	1.6441147	LOC114108599	-1.615595002
NBEAL1	1.643346496	ANO4	-1.614774943
SOX9	1.642769771	FAT2	-1.614511707
CXHXorf21	1.642108016	LOC101108817	-1.613765835
LOC101105533	1.642108016	LOC101109593	-1.613740281
ARSK	1.6420996	B9D1	-1.611905543
ACVR2A	1.640563727	NOL6	-1.611759676
PMAIP1	1.639526274	RAP1GAP	-1.61113504
RNF112	1.639460355	ATP13A2	-1.611113999
ITPKB	1.639278907	MST1	-1.610624902
PLAGL1	1.637025696	ADGRA3	-1.604184371
USP49	1.636266479	LOC114108707	-1.60382538
BICC1	1.635928057	GABBR2	-1.603799247
PARD3B	1.634282915	MRPL13	-1.601872104
LOC101106528	1.633705234	CLPP	-1.601546389
CYP2J	1.633349997	CENPS	-1.60008703
LYSMD3	1.633126578	LOC114116429	-1.599893601
NFIA	1.633096651	XIRP2	-1.599893601
AMOT	1.631602312	SRD5A1	-1.599005349
LOC101103343	1.630590886	MYCBPAP	-1.597222956
LOC101103720	1.630567668	LOC105607806	-1.596278643
ADGRG6	1.629683746	FPGS	-1.594656822
ETFBKMT	1.629024967	SLC28A1	-1.594350365
HMCN1	1.627969854	SLC6A4	-1.593791494
LOC101120993	1.627550827	CFAP100	-1.593542487
ARHGEF6	1.626789955	LOC105613621	-1.593542487
ADAM33	1.626187093	THAP7	-1.593216271
ADAP2	1.625543943	ZNF296	-1.592531284
XPNPEP2	1.625170577	FAM83H	-1.589580812
GULP1	1.624191274	PCNA	-1.588759146
NAPB	1.623841527	PRMT1	-1.588119688
YY1AP1	1.622808538	LOC114114003	-1.586760272
TMOD3	1.622633318	LOC101116852	-1.585940936
SERTM1	1.622374426	LOC105602080	-1.585306635
HIPK3	1.622000669	NOC2L	-1.583678356

SCRN1	1.621758532	BTBD6	-1.583636467
KLHL6	1.621616963	LONP1	-1.583136337
LOC114114566	1.62102027	LOC101105019	-1.581977078
MLLT3	1.620780695	LOC114112914	-1.581977078
KHDRBS3	1.620718074	KLC2	-1.581355878
GDPD5	1.619662559	TPBGL	-1.580733561
CLCC1	1.618414737	LOC105607931	-1.580733561
SEL1L3	1.618069674	RAD51	-1.580472561
LOC114116167	1.617708866	VARS	-1.580438236
RHOB	1.617409235	BRCA1	-1.579791492
LOC114114793	1.617233761	CCDC154	-1.579501438
ZBTB1	1.617230528	POMT2	-1.578123749
EEA1	1.616279574	LOC101106040	-1.577943325
CD3E	1.616018093	TALDO1	-1.577672187
ZBTB46	1.615380425	BEND6	-1.577432354
BACH1	1.615279373	SETD9	-1.576604134
MANBA	1.614786837	SLC7A1	-1.576587277
CXCL9	1.613809119	LOC105609312	-1.574895968
ALCAM	1.613481237	TDP1	-1.572612287
LOC114114876	1.613269601	ASPSCR1	-1.571757153
SMAD6	1.612902757	RPUSD4	-1.567973442
SLC27A5	1.612656892	PLEKHJ1	-1.567683778
C24H16orf54	1.612389182	LOC101121639	-1.567600948
RHOQ	1.611514524	NAA10	-1.56694116
STK17B	1.610643022	BIRC5	-1.566241874
XG	1.610587777	TNFRSF6B	-1.566206992
SH3TC1	1.61025993	GADD45GIP1	-1.56602677
ATP2B4	1.610165906	PLXNC1	-1.564788169
LOC106990525	1.609884288	NR2F6	-1.56459553
SMURF2	1.609807054	TP53RK	-1.564252603
OSBP2	1.609485408	PLPPR3	-1.562720963
LOC101103766	1.609170212	TMEM150A	-1.558678059
CST7	1.609170212	PHB2	-1.558351072
SMARCA2	1.608873875	SREBF2	-1.555386178
CSAD	1.608671212	RRP7A	-1.553012771
LOC114114074	1.608253162	FDX1	-1.552553739
DSEL	1.607770326	HRAS	-1.552270044
LOC101119572	1.607032256	MCM7	-1.552200768
LOC114117766	1.607032256	IFT22	-1.55163285
P2RY8	1.607032256	LOC114110265	-1.55158434
BMP2	1.60686477	DLGAP3	-1.550638604
HEG1	1.606801813	SLC25A39	-1.550086714
ZMYM5	1.606233495	TMEM126A	-1.549547648
NOD2	1.605850732	AGBL2	-1.548683499
TRIM56	1.605736172	SLC4A11	-1.54847623
DIO2	1.605510806	LOC101103182	-1.54847623

LOC114112673	1.605326488	ELAVL2	-1.548253083
NKX3-1	1.604975202	LOC114108997	-1.547751255
FZD7	1.604662469	HRH2	-1.547751255
SEC24D	1.603797603	QPCTL	-1.547625673
GNG8	1.603348879	NME4	-1.547192203
WNT10B	1.602775461	LOC101117343	-1.547158634
LOC100037664	1.602775461	C2H9orf43	-1.547158634
LOC114115280	1.602775461	LOC101113599	-1.546998632
LOC114113812	1.602775461	TUFM	-1.546655635
LOC114110120	1.602775461	LOC106991980	-1.546037105
TNFAIP3	1.602105209	RYK	-1.545641533
TIMD4	1.601481436	LOC105604300	-1.545070908
CCDC191	1.601460757	LOC114113005	-1.544497839
RASL12	1.601146756	SCN8A	-1.544497839
HACD1	1.600674842	NHLRC1	-1.544273799
ADAMTSL1	1.600546346	ZNF414	-1.543289546
EEF2K	1.599163871	H2AFZ	-1.543154541
ATP8B1	1.598923941	LOC114108814	-1.543107994
LRIG3	1.598365744	HYOU1	-1.54298184
LOC105610487	1.598041732	LOC105602770	-1.542254033
SPAG9	1.596085722	AIFM3	-1.542254033
YOD1	1.595052549	CA14	-1.541259023
SEMA5A	1.5939665	LOC114110125	-1.541259023
FAM3C	1.593810057	NEK2	-1.540975108
RFX7	1.593031541	SSRP1	-1.540865788
GATA5	1.592376311	LOC101102413	-1.54008487
RASGRP2	1.59149026	ADCK1	-1.53969905
ARID4A	1.589750714	ACAD9	-1.539458561
GOLGA4	1.589242799	DLX4	-1.538678409
EPM2A	1.588019575	KCNH4	-1.538678409
LAMC1	1.587811323	LOC114112887	-1.538678409
HCLS1	1.586595378	ONECUT1	-1.538678409
LOC114111428	1.586342213	LOC101116968	-1.538678409
LUZP2	1.586342213	CIAPIN1	-1.536433868
GATA2	1.585337558	LOC105606390	-1.534824763
ADRB2	1.585229815	LOC114117542	-1.534824763
CD300LF	1.584388704	LOC114116375	-1.534824763
LOC105610195	1.584388704	LOC114110144	-1.534824763
GFAP	1.58385038	VSIG8	-1.534824763
PER2	1.582494572	LOC105608080	-1.534824763
LOC114115624	1.582492187	MYL7	-1.534824763
ICAM5	1.581494713	SLC12A5	-1.534824763
CKB	1.580793879	LOC106990587	-1.534824763
SYNE1	1.579644362	RANGAP1	-1.53389964
NF1	1.57936237	CDPF1	-1.532758093
KMT2E	1.579281715	NSMCE3	-1.532630513

ADAM19	1.579265872	MARCH9	-1.530878207
MIGA1	1.579072888	LOC101111367	-1.530025316
OLFML1	1.578433804	ECHS1	-1.529559781
LOC101108321	1.578094075	TMEM201	-1.528609791
RFESD	1.576874014	INCENP	-1.525966704
CPEB2	1.576801871	EFCAB2	-1.525761394
SLC26A7	1.576399903	NUDT14	-1.525580561
ZNF704	1.575785197	GADD45A	-1.525308101
MINDY2	1.574469852	LOC106990963	-1.524515335
CSPG4	1.573165324	MZT2B	-1.523896491
PDE1C	1.573023774	TRABD	-1.523734655
LOC105610993	1.572854607	C2H9orf40	-1.523708187
PIK3AP1	1.572443725	EXOSC7	-1.523069354
THBS3	1.571892367	MYOM3	-1.522961732
ATP2A2	1.571840818	FBP1	-1.522738891
CEMIP2	1.571805291	RTKN2	-1.522738891
PIK3R6	1.571793972	GPR88	-1.521372043
FHDC1	1.571467126	SARS2	-1.520234591
LOC101109219	1.570127215	RNASEH2B	-1.520047749
STAB1	1.569962727	LOC101113696	-1.518078521
RPS6KA3	1.569353861	DUT	-1.51803374
LOC114110097	1.568226842	MRPL21	-1.517817597
ELK4	1.567736847	MRPL23	-1.517743421
LOC114110110	1.567304619	TSGA10	-1.517596016
PTPN11	1.566692265	LOC114108791	-1.517446666
ITK	1.566656063	DMXL2	-1.517064463
SEC23A	1.566401892	MRPL41	-1.515986496
LOC114117770	1.565956808	KIAA0319L	-1.514402619
LOC101105614	1.565834268	LOC101110577	-1.513818013
DLC1	1.565705511	TUBB4B	-1.513751575
CNTFR	1.565475658	RNASEH2C	-1.512370513
ARAP3	1.564332406	FEN1	-1.51187732
TMEM107	1.563337733	SIGMAR1	-1.51140241
IKZF1	1.562754215	STK11IP	-1.510881705
STON1	1.561922801	LOC101111670	-1.510312193
GPR155	1.561576776	CCDC71L	-1.510022079
DENND1C	1.561418606	LOC101121590	-1.509438557
ABL2	1.561385458	PGAM1	-1.509005711
CFL2	1.560428873	PPP1R1A	-1.508111683
DACT3	1.560396625	LOC105607442	-1.508049112
SLC45A4	1.559642389	YIF1B	-1.507499418
USP25	1.559361809	SIVA1	-1.50664437
KIAA0754	1.558522016	LOC114116647	-1.502140912
PPL	1.558393035	THAP4	-1.502137396
SERINC4	1.558001348	SLC25A1	-1.500334354
LOC105606870	1.556481104	NCAPH2	-1.500088239

LOC114117316	1.556233165	EBF4	-1.499270565
LOC105608665	1.555548342	C5H19orf24	-1.499026263
LOC114117629	1.555395173	LOC114116394	-1.498851024
TSPAN12	1.555132067	TMEM11	-1.498672066
LOC114116409	1.554926381	WASHC1	-1.498493479
KIAA1328	1.554322356	SUV39H1	-1.498464882
FNBP1	1.553970742	CMTM5	-1.497833808
ANKRD2	1.553589055	RIMS1	-1.497321935
FIGN	1.553575098	ACSM1	-1.496319152
HIP1	1.553356625	LOC101103401	-1.496319152
WWTR1	1.553286698	TMEM200A	-1.496242591
PARP8	1.551770378	NOLC1	-1.494938865
IGSF6	1.551754931	DEPDC7	-1.494139439
NFATC2	1.550029596	SPTBN4	-1.492637666
CHRD	1.549783316	RCC2	-1.492181111
SMG1	1.549063638	NOL12	-1.491745416
EPDR1	1.547162929	RNF126	-1.491555869
LOC114118399	1.546680881	LOC101104501	-1.490846316
LIX1L	1.54654038	TRIM28	-1.488571521
RAB27B	1.545965113	TMEM176A	-1.48623758
MYH10	1.545818749	MOGS	-1.485072153
NXPH4	1.545806917	HSPD1	-1.484680186
AXL	1.545266647	ABHD6	-1.484489495
LOC105615181	1.544989466	RND1	-1.483880014
KYNU	1.544989466	FMN1	-1.483491361
ABTB2	1.543771686	LOC101109157	-1.483491361
LOC114109073	1.543219042	C17H12orf49	-1.482305059
NACC2	1.542983394	RBP1	-1.481753214
LOC101118851	1.542078281	TMCC2	-1.481035742
CSF1R	1.54170935	LOC114109653	-1.480140068
ADGRF5	1.541035183	LOC114115656	-1.479340963
AKT3	1.540169721	C3H12orf10	-1.477815627
LOC105610817	1.539981186	HDHD5	-1.477402177
LOC101112306	1.539836318	APOO	-1.47709247
TRPC4	1.539759882	LOC114110277	-1.476616291
REEP3	1.539260534	GPT2	-1.474801667
SFMBT2	1.539165132	CHKB	-1.474479124
SDC1	1.538661039	ALG12	-1.474361583
ZNF521	1.53857754	KCNE5	-1.472652445
LOC114110610	1.538280735	ZNF684	-1.472652445
KIRREL3	1.538116579	KIAA0319	-1.472652445
BARX1	1.538061096	ACSS1	-1.471333795
TMEM88B	1.53805065	ME1	-1.47079268
ITPR1	1.537017935	HSP90AB1	-1.470418137
SPIN4	1.536881252	COQ4	-1.468336852
KIF5C	1.536569897	DGKQ	-1.468107219

NT5DC3	1.536569897	RASL10B	-1.467823198
CEP85L	1.535751203	LOC114115233	-1.467593581
UBN2	1.53418915	CHCHD6	-1.466409198
LOC101111337	1.533973131	CSDC2	-1.46636
KCNE3	1.533935492	LOC114110457	-1.465086489
FGD6	1.533036562	LOC101122683	-1.465086489
CERS6	1.532374878	ZSWIM1	-1.464592265
LOC114113062	1.532335708	LYPD1	-1.464008679
LOC106991345	1.531485563	LOC114114607	-1.463952018
CARD10	1.531106386	TXNL4B	-1.462947133
LOC105613077	1.530959648	STMN3	-1.46270667
LOC114117881	1.530942191	LOC114111346	-1.462396522
CD2	1.530510419	AEN	-1.460645181
RASL11A	1.530361922	LOC114116894	-1.460133077
ZFP36	1.530193513	RPL28	-1.458680553
CYTH4	1.529721224	CENPM	-1.458510964
MAP1B	1.528693996	GFRA3	-1.458333777
SPTBN1	1.52864232	FASN	-1.458318022
LOC114108620	1.528574258	TCF19	-1.457487724
LOC114109697	1.528500492	CHCHD10	-1.456002908
AKAP11	1.528376511	LOC101120481	-1.45526801
ARHGEF37	1.527755102	LOC101121595	-1.454858227
TCIM	1.527738011	ARMC6	-1.454774163
SPN	1.527511498	MANF	-1.45415051
C1QC	1.527485957	POLR2E	-1.45235773
GPR182	1.527285212	MIS18A	-1.451838621
SEMA3B	1.527258062	LOC101112635	-1.451712622
VWF	1.526704227	FAM136A	-1.451333174
DTNA	1.526135312	ALKBH7	-1.450154766
SH3RF3	1.52495921	DEDD2	-1.449766846
OXR1	1.524744647	LOC101110918	-1.44928131
TRIM44	1.524665642	SLC2A8	-1.449199486
FLNB	1.523958036	FAM83D	-1.449181913
PDZRN3	1.523955148	CRB1	-1.448971725
ASPN	1.52385727	HSPA8	-1.447967104
SMYD4	1.523308505	PLIN2	-1.447199193
EPS15	1.522971253	LOC105609758	-1.445982079
UBL3	1.522820154	CLUH	-1.445427655
BOD1L1	1.522773436	TARBP2	-1.444958222
UST	1.522558086	SCD	-1.444902113
ZBTB18	1.522220238	LOC114118751	-1.444683648
KCTD10	1.521795211	SPC24	-1.444400058
CCRL2	1.521475933	VILL	-1.443779201
STAP1	1.520697151	LIMK1	-1.443546875
LOC105611303	1.520697151	HELLS	-1.442569083
MFSD6	1.520265838	LOC106991919	-1.442404225

DIP2B	1.519515883	DIPK1B	-1.442124097
KIAA0355	1.519044957	PEX11G	-1.441853507
PDLIM3	1.518782314	TFPT	-1.441310153
THAP5	1.518338358	LOC114109555	-1.44019024
NKTR	1.518229408	CPSF4	-1.439423824
IL6ST	1.516864191	EIF3B	-1.43791564
IL16	1.516468582	MTLN	-1.437337066
PTGER4	1.515800483	MBD3	-1.4369244
ZNF568	1.515254528	LOC114114052	-1.436845566
FEZ2	1.514146572	CASKIN1	-1.436845566
ARL13B	1.513820132	APLN	-1.43665817
EFR3A	1.513436044	SDSL	-1.436620068
GABPA	1.513183542	CNKSR1	-1.436620068
FGD3	1.511924712	TMUB1	-1.436416385
CPT1A	1.51176881	TYMS	-1.435157123
IL18	1.51038712	WDR46	-1.433949982
MYCT1	1.51016678	LOC114116593	-1.43389395
ARMCX3	1.509886837	DEFB112	-1.433670645
RALGAPA1	1.509883959	TMEM51	-1.433470214
GPR63	1.509426151	HTR7	-1.433025571
LOC114110253	1.50831887	WWOX	-1.432935305
EBF1	1.507737786	PISD	-1.432620692
SECISBP2L	1.507499324	FKBP4	-1.431469734
CDS2	1.507212004	TBRG4	-1.431428378
UHMK1	1.50627816	LOC114116877	-1.430991808
LOC105604204	1.506164037	ST6GALNAC4	-1.430602469
LMOD3	1.506164037	LMNB1	-1.430498166
VNN2	1.505656171	CDC34	-1.430230704
PIK3R5	1.504785077	PIGU	-1.425030559
GPM6A	1.503605631	ZDHHC16	-1.423766212
KLF10	1.503129669	RPL26L1	-1.423290084
GGTA2P	1.50195978	MRPL57	-1.423278025
MFAP3	1.501673702	LOC101119989	-1.422515423
IFT81	1.50148633	LOC114108828	-1.42241435
CYYR1	1.501409183	TSPAN17	-1.42193242
LOC101107908	1.501390214	CCDC58	-1.421378276
RAB27A	1.500921865	EIF4A1	-1.418035391
LOC101113741	1.500823279	GPAT3	-1.417530291
BTBD7	1.500530658	RAB32	-1.417264223
MFSD8	1.500244419	WDR25	-1.416733054
WSCD2	1.500194338	LOC114117876	-1.416316856
OGFRL1	1.499648923	RGS12	-1.415827092
HERC3	1.499305486	RFC4	-1.415576157
IL2RG	1.498840701	NTF4	-1.41509492
C13H20orf194	1.497483357	LOC101121420	-1.41505292
HSD11B1	1.49726605	SERGEF	-1.413628282

ITSN1	1.496549427	AFG1L	-1.411353138
RGS3	1.495341125	RRM2	-1.411344957
JAK2	1.494645604	GTF2IRD1	-1.410129
LOC106991294	1.49455494	LYRM9	-1.409965727
ELMO1	1.494497099	NOXA1	-1.408523788
COL4A1	1.494265018	XKR5	-1.408523788
CCDC88B	1.492560946	FUOM	-1.407831979
PIK3CD	1.492470019	CYGB	-1.407352884
PIP4K2A	1.492237871	BRAT1	-1.407293415
PAPPA	1.491617235	ARMH4	-1.403832403
PPP1R9A	1.49134351	IRS1	-1.402948496
TMOD2	1.490437772	LOC114115368	-1.402720981
GXYLT1	1.489804806	AGAP3	-1.402617762
LOC101109746	1.489202851	TFCP2L1	-1.402604368
APPL2	1.488475653	CD3EAP	-1.402037406
SYNJ1	1.487875731	SLC35B1	-1.401683412
CHD5	1.487622921	LOC101103001	-1.400986759
PLXDC2	1.48760869	GET4	-1.40053461
CTSK	1.486847284	ANAPC5	-1.400289874
PPM1L	1.486677457	ZNF593	-1.397864779
HIVEP3	1.486065975	DNMT3B	-1.397057395
LRRC8C	1.485582713	RRS1	-1.397042732
LONRF3	1.484580973	SPEF1	-1.396253566
CD55	1.483954465	TMEM161A	-1.396174843
IL13RA2	1.483936623	BRI3BP	-1.396155736
ITPRIPL2	1.483033917	ARL6IP4	-1.395932151
CYS1	1.482963304	LRP3	-1.395577832
MASP2	1.482935573	AIMP2	-1.395519569
LOC105606943	1.482302376	KIF19	-1.394877562
CDH5	1.481943921	RAD54L	-1.39391094
LOC105603399	1.481729178	FAM162A	-1.393494238
HTR6	1.480835978	RAPSN	-1.393323
MAP4K3	1.48082762	ZNF358	-1.392949723
PAM	1.480757141	NDUFAF8	-1.392912851
SLC35D2	1.480425935	CCNJL	-1.392540364
LOC105605886	1.4787899	CLEC1A	-1.392443512
LBH	1.478678553	CELA3B	-1.391715658
TM6SF1	1.477933632	LOC114114536	-1.391692645
CILP2	1.477837759	CCNE1	-1.391148581
TMEM232	1.477837759	SREBF1	-1.390520299
NFKBIZ	1.477001229	ANAPC2	-1.390503691
EDN1	1.476910107	METTL1	-1.39042987
PLEKHA3	1.476837398	TMEM185A	-1.389999704
SVIL	1.476730008	LOC114112992	-1.389784181
MMP17	1.476410327	LOC101120681	-1.389430888
RASSF8	1.475781545	TFAP4	-1.389381664

GRK3	1.474187062	FAAP100	-1.389201547
FKBP14	1.473784774	MTHFD1	-1.388114763
LOC105615677	1.472382315	LOC105611292	-1.387537909
PLEKHG1	1.472294111	PGD	-1.386508818
NRCAM	1.471997018	LOC114111456	-1.385242368
KIAA1755	1.470739598	LOC114116609	-1.385242368
SNX16	1.470648602	GRIP1	-1.382967363
CEP170	1.469976687	SPIRE2	-1.382942186
PTAR1	1.469774489	LOC114110676	-1.380275761
RHOBTB3	1.468968767	FARSA	-1.380180245
ZBTB7C	1.468477002	MRPL2	-1.380091208
MAP4K5	1.465959589	ZNF462	-1.378472068
PTPN14	1.465891743	RCC1	-1.378404577
AGTPBP1	1.465681024	HERPUD1	-1.378339233
TNFAIP8	1.464964615	TMEM163	-1.378097852
FRRS1L	1.464886573	TEKT2	-1.376196156
JADE1	1.464792119	SLC52A2	-1.375869192
RBPJ	1.463794617	TIMM17A	-1.374890833
LOC105616742	1.463744418	LOC101104866	-1.373660692
NEURL3	1.46336826	CCDC167	-1.373073211
LOC106991947	1.46336826	NIPSNAP1	-1.373012522
ABCB1	1.46336207	CREG2	-1.371766144
GPBAR1	1.462881785	SLC12A7	-1.370980906
XYLB	1.462561885	HDGF	-1.370445984
NLGN3	1.459833881	POC1A	-1.370416465
KCNJ2	1.459487708	TSFM	-1.370386551
GBP6	1.458862157	MAD2L1	-1.369406653
PPP1R12B	1.458531267	LOC114113987	-1.368994717
PCDH1	1.458530728	HEXIM2	-1.367697074
LOC114116350	1.458128385	CEBPZOS	-1.367315834
LPGAT1	1.457936327	MANEAL	-1.367259196
WASHC4	1.457422775	SNAP29	-1.366775027
FER	1.456105523	UFSP2	-1.366162988
LOC114115243	1.455847074	LOC106991494	-1.366156192
TSPAN18	1.455154422	LOC114118295	-1.364457201
LGI4	1.454419214	ALDH1L1	-1.364259451
MOG	1.453667078	LOC114109439	-1.363759433
TBC1D12	1.452423051	MRPS34	-1.362942745
IQGAP1	1.452296545	ERFE	-1.362626684
FERMT2	1.452225306	MTX1	-1.361772817
ABI1	1.452007747	SNX8	-1.360907898
JAM2	1.451504978	ING1	-1.359901406
SREK1IP1	1.451267589	DTNB	-1.359044797
RC3H1	1.451197314	MED12L	-1.35904135
PLIN4	1.449067576	HYLS1	-1.358889579
SSPN	1.447688102	LOC114111250	-1.35860639

TRIM2	1.446780618	CHST15	-1.357864482
FLNA	1.446459167	LOC114118070	-1.3555698
ASIC4	1.443718047	SHANK1	-1.355026111
RP2	1.443332477	NAT10	-1.35493692
LOC114113883	1.442846587	DCP1B	-1.354699601
LOC105608011	1.442237259	FAM133A	-1.354059594
GCA	1.441971834	OGFOD3	-1.353965103
TRAPPC8	1.441938064	NCDN	-1.353509438
ATP9A	1.441874281	THYN1	-1.353063415
C25H1orf198	1.44169918	SPIB	-1.352547359
ZC2HC1A	1.441049779	MPIG6B	-1.352547359
LOC101113619	1.440858228	JAGN1	-1.351798373
LOC114110278	1.440858228	STOML1	-1.351501751
GPR1	1.440858228	TOMM34	-1.349798688
LOC105607533	1.440322727	ATP2A1	-1.349538962
LOC114110418	1.440322727	KRT222	-1.349538962
TBC1D8B	1.439536155	ANKS3	-1.349018551
C1R	1.43913567	TMEM88	-1.347701534
ANKRD33	1.438795156	SPOCK1	-1.346463155
LOC114113232	1.438648762	C14H19orf33	-1.345201796
FUCA2	1.438315569	MRPL24	-1.345106143
FAM126A	1.438242974	PRMT6	-1.34489915
TENM2	1.436783949	LOC101116687	-1.344675886
IRF4	1.436054562	LY6E	-1.344656759
LIF	1.435512369	DRC3	-1.343005473
LOC105610169	1.434140776	LOC114109632	-1.342918063
ELK3	1.434054662	MARK1	-1.342514376
SPATA6	1.433926027	HMGA1	-1.342187457
LOC101120999	1.433666019	ABHD17A	-1.341630023
NETO1	1.433666019	POP5	-1.341149707
ZBTB16	1.433484791	LOC101111215	-1.340252924
GAB3	1.432962438	PTTG1	-1.339831475
KDM1B	1.432651597	INTS1	-1.339452572
PODXL	1.432577881	TMEM98	-1.33760032
MORC3	1.431319897	TSPAN5	-1.336527853
MYADM	1.431266102	PSMB1	-1.336520731
RTN4RL1	1.431224455	TFB1M	-1.336065745
GPCPD1	1.430879766	MFSD3	-1.3360004
HIGD1B	1.430411056	SLC26A6	-1.335184312
KIF3A	1.42961296	GEMIN8	-1.335136722
SPAG1	1.428737432	NUDT1	-1.334669928
TM4SF1	1.42856047	ARHGEF28	-1.334451482
NCKAP1L	1.427845909	ZNF511	-1.33423443
GLUD1	1.42724975	PRELID1	-1.333273186
PHACTR1	1.426323769	DDX56	-1.332898784
RAB29	1.426013637	LOC105602911	-1.332809948

NKG7	1.425690331	CENPP	-1.33280939
HCFC2	1.425214022	RAPGEF4	-1.332660884
ANGPTL2	1.42490725	SLC22A23	-1.332321283
YAP1	1.424847917	CCHCR1	-1.332249526
ASPH	1.424624136	SUPT3H	-1.331871783
ABHD5	1.423470307	KNTC1	-1.331782011
NOTCH1	1.423347885	BCKDHB	-1.331220529
UNC5D	1.42284983	NXPE4	-1.331141712
ST7	1.422692436	CHTOP	-1.330990148
LOC105603415	1.422164198	C12H1orf112	-1.330924764
BCAM	1.422123367	TSEN34	-1.330694197
NRROS	1.420276377	MRPL36	-1.330649072
TCF7L1	1.420101913	FAM184A	-1.33043277
CRPPA	1.41984964	CDK5R1	-1.32961362
SEL1L	1.4198312	ADCK2	-1.329146954
PBXIP1	1.419617284	IGSF3	-1.328683108
SFT2D2	1.419489689	DRG2	-1.327053208
ASH1L	1.419314561	WDR31	-1.32675485
COLEC12	1.419049567	AAAS	-1.326382159
IL10RA	1.418807972	PSMB2	-1.326188705
HIF3A	1.418505133	MRM1	-1.32592048
TLR2	1.418478908	FAM110A	-1.325152511
LOC101109728	1.418081313	LOC114114048	-1.323051722
SPATA13	1.417633652	FARS2	-1.322949331
SERPING1	1.417410741	RFXANK	-1.322835543
MACF1	1.416370073	GSR	-1.322827565
C26H4orf47	1.416307676	LOC114113841	-1.322778065
GSTP1	1.4159631	PAXIP1	-1.322583221
OSBPL8	1.415896061	LHCGR	-1.322233976
SRGAP3	1.41421681	CEP83	-1.321803941
LOC105607146	1.413565974	LOC114118293	-1.321641183
LOC105612882	1.413005361	ADRM1	-1.321583384
HELB	1.412634312	LOC101117650	-1.321355849
GSKIP	1.412124209	AJAP1	-1.321197044
BLNK	1.411443366	PIR	-1.320936179
SH2D1A	1.411340877	DNAJC9	-1.319905394
LOC101115486	1.410972586	NELFE	-1.319544554
PLXNA2	1.410948936	TEAD2	-1.319322605
LOC101111006	1.409674389	CENPU	-1.319236677
KLHL38	1.409278995	STXBP2	-1.319007118
MAN1A2	1.409074246	STXBP5L	-1.318240648
LOC101108528	1.408890587	ALPK2	-1.317652501
GRIN2D	1.408527984	TMEM82	-1.316243267
DNAJB14	1.408375334	RNASEH1	-1.315815787
ACVR1	1.40734036	SPHK2	-1.315199322
DQB	1.407309179	LOC101113001	-1.314375522

PRICKLE1	1.406788817	RIOX2	-1.314148046
SDC2	1.404984021	CLBA1	-1.313180968
UTP15	1.404898977	LOC101120607	-1.313154606
GCHFR	1.404841374	BRMS1	-1.313112976
LOC105611708	1.404407841	GABRB3	-1.312669767
TEAD1	1.404383241	FBXO24	-1.312669767
ACAD11	1.404292364	THEM6	-1.311551207
CASP8	1.404251966	ECSIT	-1.311426958
PARVG	1.403995875	LOC114112974	-1.31133962
LOC101108647	1.403878767	POLRMT	-1.310898349
PID1	1.40290455	SIPA1L2	-1.309380159
TMEM123	1.402608495	ZNF274	-1.309008697
ZKSCAN8	1.401794442	LOC114112520	-1.308783682
TLR4	1.401641107	LOC101115252	-1.308578299
LOC105613248	1.401157223	FUS	-1.308527214
PDE9A	1.40067674	LOC114114506	-1.308321192
NUBPL	1.399873551	ATP13A1	-1.308116503
LOC114112896	1.399679354	HAAO	-1.307789739
PIKFYVE	1.399661046	RBM19	-1.30775192
ADA2	1.399491755	TMEM259	-1.307640919
LOC105608222	1.39842207	ETHE1	-1.307220937
SUSD6	1.39823293	TNNI2	-1.307202712
RFK	1.398134776	ELOF1	-1.307083679
PPP3CA	1.397319194	PHB	-1.306043683
S1PR1	1.39720648	JAK3	-1.305893913
HNMT	1.396930711	GMNN	-1.305797112
CD47	1.396472619	GNAZ	-1.305689533
SOX18	1.396322062	LOC101123112	-1.305335984
SACS	1.396304572	MRPL20	-1.305242586
LOC101122398	1.396205871	ZNF579	-1.305025075
TNFSF9	1.393968934	PSMD3	-1.304793966
LOC101102480	1.393625509	KCNIP4	-1.304721973
LOC101116121	1.392922015	DDX54	-1.30463605
F11R	1.392399566	BYSL	-1.304383923
ATG2B	1.392006782	SLC43A3	-1.303271986
SPTLC2	1.391669315	SMOX	-1.302833475
NIPAL3	1.391570058	EMC9	-1.302339924
GIMAP6	1.391425033	LOC105606907	-1.302332981
PCSK6	1.391256328	FRAT2	-1.301864808
MEF2C	1.390922284	COL6A5	-1.301604736
BTBD19	1.390053202	PYCR3	-1.301119917
FBXO30	1.390053202	PTK2B	-1.300911449
NFIB	1.389712485	DNLZ	-1.300703503
IL13RA1	1.388786646	MDH2	-1.30056764
SLC2A4	1.387932514	LOC101109915	-1.300285682
DOCK2	1.387800151	LOC114114855	-1.300285682

SIPA1L1	1.387700693	LOC105603102	-1.299267707
TSPAN9	1.387298197	SLC7A5	-1.298495844
MBTD1	1.385212942	SDHB	-1.298201275
ADAMTS8	1.385106902	WDR74	-1.297918068
AGRN	1.384818248	USP35	-1.297630835
C4H7orf57	1.384682409	FAH	-1.297308341
LOC105610483	1.384655567	PRR36	-1.297140284
RCOR3	1.383712462	LRRC75A	-1.296707972
LY96	1.3831015	NSDHL	-1.296577307
LOC101108171	1.382820727	ASMTL	-1.2960001
ECSCR	1.382799913	LOC114118084	-1.295952184
GMFG	1.382604971	SNRPD3	-1.295227002
CCDC69	1.382466313	ADAMTS14	-1.295196944
RAB20	1.382459327	ANKRD45	-1.293779597
LOC105605978	1.381864659	SYNGR1	-1.292852639
TUBB4A	1.381864659	CLPTM1L	-1.292478026
COL4A2	1.381854289	HDDC2	-1.292343198
LOC101122545	1.381675288	CCDC86	-1.292177238
ZHX1	1.38105855	GRPEL1	-1.291451547
SMIM13	1.380905957	HHAT	-1.290840658
FNDC3A	1.380565493	TMED1	-1.290754751
GNB3	1.380505501	NT5E	-1.290677709
LOC114109030	1.380351336	EPCAM	-1.28877601
CDC42EP3	1.38021411	TIMM44	-1.286619907
DUSP8	1.380139954	IL1A	-1.28536588
LHFPL6	1.379237889	TSTA3	-1.285207487
SGCE	1.378883117	RPL7A	-1.285149739
STARD13	1.378104034	CENPA	-1.284931633
TANC1	1.378022974	ZNHIT2	-1.28484123
AQP1	1.377879	NT5C3B	-1.284724608
SLC40A1	1.377767806	PUS1	-1.284688217
RBL2	1.377565205	ZNF428	-1.284670368
ELMSAN1	1.377404996	TIMM22	-1.284600602
PTER	1.377155727	GAK	-1.284374436
SEPT7	1.377054555	DET1	-1.283608022
LOC114114916	1.376464328	HMGN2	-1.283606676
SERINC1	1.376353282	LOC114114556	-1.283410939
MAN2A1	1.374821176	TBL3	-1.28276013
SLC9B2	1.374728012	LOC105612761	-1.281851415
DENND5B	1.374716405	LOC105612442	-1.281851415
ST6GALNAC3	1.374474608	APOE	-1.279977867
LOC114116073	1.373450183	MRPL30	-1.279850518
CLK4	1.373098101	IGSF9	-1.279519474
SLC36A4	1.372632425	LOC114118007	-1.279519474
MYSM1	1.372492501	LOC114109706	-1.279519474
PDZD7	1.371498282	LOC106990971	-1.279519474

WAS	1.371304211	IGLON5	-1.279446972
PTPN6	1.371000624	C1H3orf33	-1.279241963
GUCY1B1	1.370657383	PKDCC	-1.278209173
RRAS	1.370563427	IQCN	-1.277809377
GLI3	1.368697205	NMNAT3	-1.277410322
MRAP2	1.367747753	LOC105610456	-1.277232426
PPP2R2C	1.3673981	SPG7	-1.27678466
LOC101115646	1.367070278	TRMT2A	-1.275013185
SLAMF6	1.367012594	OSCP1	-1.274762693
LRP4	1.366824876	NDUFAF3	-1.274395709
SHROOM4	1.366576097	PTRH2	-1.274109249
TMEM65	1.366510067	POLR3H	-1.273795391
PPP1R3D	1.366482215	NDP	-1.27352672
SLCO2B1	1.365975122	RPL22L1	-1.273499311
DDHD2	1.365431328	LOC105605360	-1.272546133
LOC105611269	1.365376367	PCCB	-1.272034309
SPDL1	1.365105057	LOC114117998	-1.271846771
ARRDC5	1.362921893	CROCC	-1.271189446
ASAP2	1.362682839	KLHDC9	-1.270187216
NR3C1	1.362661128	ERCC2	-1.270106504
CD247	1.362570763	SOWAHB	-1.269739771
STXBP4	1.362482896	MAN1B1	-1.268759041
ANXA5	1.362386738	LOC106990580	-1.267996566
LOC114113049	1.362363229	SMARCB1	-1.267909103
ARL4C	1.362363229	REX1BD	-1.267525184
SLC34A3	1.362363229	SLC26A11	-1.267395645
LOC105616457	1.361827055	EPOP	-1.26716218
TSHZ3	1.36163462	MYO10	-1.26714014
TTC26	1.360638793	DND1	-1.267096864
PTP4A3	1.36054488	C5H5orf63	-1.265943694
TYROBP	1.359719752	ZSCAN2	-1.265848865
SMAD1	1.359574525	PNCK	-1.265495423
VAMP7	1.359131336	CCNE2	-1.265068822
ADAMTS17	1.358656847	FAM173A	-1.265013568
MAPK8	1.358100353	CIAO3	-1.264724604
TMEM52	1.35805294	RNPEP	-1.264329382
LOC101104528	1.358051659	TRMT112	-1.264067012
SFXN3	1.357941214	LOC105606974	-1.263813353
SHOC2	1.357079012	SLC25A22	-1.263587172
TENT4B	1.356966006	LOC114117236	-1.263123486
PUS7L	1.356822437	NAGPA	-1.262965617
GDF11	1.356137101	DUSP15	-1.262638014
FUT8	1.355636721	LOC101115315	-1.262561337
RAC2	1.355333367	ZXDC	-1.262457882
HEBP2	1.35520117	RRP12	-1.260742165
ZFYVE16	1.353574142	LOC101110181	-1.260522228

LOC101102857	1.352913589	MMP25	-1.260522228
TRIM45	1.352607144	SLX1A	-1.260488704
CD99	1.352604114	CHEK1	-1.2602242
PRKAA1	1.352289585	CALR	-1.259565761
SYT1	1.352273443	WDR54	-1.258776982
ACKR3	1.351872011	DTX2	-1.258529652
LOC114116116	1.351053005	MRPL37	-1.258520646
RAB33B	1.350795456	PNMA1	-1.258036594
HPCAL4	1.350676014	HSD3B1	-1.257891564
LOC105604257	1.349268128	MGAT3	-1.257549723
LOC105610030	1.348716514	PRR3	-1.257539397
LOC114110986	1.348716514	NT5M	-1.256556523
PPP1R3E	1.348716514	MMACHC	-1.256428321
IRX3	1.34861367	LOC105607964	-1.255986503
AIF1	1.348182697	SLC29A2	-1.255428334
CDADC1	1.347998458	DLGAP5	-1.254929238
SMPDL3A	1.347982907	POP7	-1.254181271
THAP6	1.347945088	AK2	-1.253873334
ASAP3	1.347475761	PSMC1	-1.253806376
VDR	1.347403479	LOC101115461	-1.253646778
UGP2	1.347322174	LOC101118248	-1.253580237
LOC101111694	1.347322068	ZNF524	-1.252805674
MEGF9	1.347051111	HSPA6	-1.252801792
CCL19	1.346789769	ABCB8	-1.252434446
SUGCT	1.346487107	CACYBP	-1.25211481
TMF1	1.345658248	HSD17B10	-1.251426109
ARL16	1.345074947	SRSF7	-1.251151978
ABLIM1	1.345037117	LOC114116050	-1.251018792
GOPC	1.34488135	SNRPB	-1.250801035
LOC105613434	1.344869077	DDX4	-1.250385183
KRT17	1.344869077	CABP1	-1.250330989
AHNAK	1.344826214	EIF3I	-1.249928351
ZDHHC2	1.344529725	FBXO10	-1.249406439
PHKG1	1.344044946	PPP1R14B	-1.249204043
KAT2B	1.343689365	LOC101117851	-1.249062893
INO80D	1.343071996	RALYL	-1.248506926
SNAP23	1.342259406	LPCAT3	-1.248074061
GM2A	1.342242943	NR2C2AP	-1.247885574
SMIM17	1.341608461	GMPPB	-1.247684036
NPC2	1.341251829	SLC26A2	-1.247439467
DDX6	1.340897595	ZNF18	-1.246781815
SH3PXD2A	1.340456374	LOC101120512	-1.246059434
RNF43	1.339997556	LOC114118455	-1.246059434
DDX3X	1.339955135	MSH5	-1.246059434
KIAA1958	1.339612945	SDCBP2	-1.246059434
BDP1	1.33870735	LIPT2	-1.245005262

TBCEL	1.337916692	LOC101109919	-1.244841612
MYH15	1.337316977	B3GNTL1	-1.244841612
ATG4A	1.337034896	TRAPPC12	-1.243737888
LOC101120489	1.336730967	JUP	-1.243502837
LOC106990463	1.336730967	LOC101111154	-1.243463475
LOC114114809	1.336730967	GATD1	-1.242915127
PARP4	1.336483009	LOC101115658	-1.242732253
MSN	1.335649092	MRPL46	-1.242694431
GPR132	1.334544395	DHPS	-1.241893853
CSF3R	1.334388354	SPAG5	-1.241877361
PLEC	1.334205692	ARHGDIG	-1.241461455
ADD3	1.334058261	OXT	-1.240844116
PCYT1B	1.334036997	LOC114112963	-1.240429563
NRG1	1.333704393	NEIL2	-1.240337608
CRISPLD1	1.333178582	NCAPG2	-1.240329615
CSRP1	1.332701363	SYT9	-1.23985389
PDE4A	1.332427075	LOC105616153	-1.239488519
ZDHHC17	1.332390153	EIF5B	-1.239353202
LOC114117015	1.332203168	RGS16	-1.237610212
KCND2	1.332203168	NDUFV1	-1.237429663
RB1	1.331779347	TXNRD2	-1.237423571
LOC101106384	1.331494198	THOC3	-1.236627964
LOC114118863	1.331252687	STK36	-1.236604523
ANKRD12	1.330687159	GPAT4	-1.236463777
ITGB7	1.329961634	LOC114116379	-1.236003425
NPR3	1.329523665	FAM78A	-1.235855296
CCDC73	1.328622195	LOC114112817	-1.235555086
C14H16orf87	1.328573953	DMAP1	-1.23446926
ITFG1	1.328057501	ZNF444	-1.234065742
LOC114115898	1.327313381	SLC22A17	-1.233074187
C5H5orf24	1.32726373	CORO6	-1.232824086
FAM177A1	1.327125646	ACD	-1.231839852
DPP8	1.326431219	TREX1	-1.231682523
CRACR2B	1.326198327	CAPN10	-1.231202679
ADGRL4	1.326071998	NDUFAB1	-1.23103499
FAT4	1.325336588	PEX14	-1.229867088
MYBPC2	1.3251972	DHRS7B	-1.229642341
PICALM	1.323928052	PCLAF	-1.229507756
CPD	1.323418295	TRAPPC9	-1.229447211
PAPLN	1.323365743	ACADS	-1.229104156
UBR2	1.323275371	LOC101117015	-1.228783379
VCAN	1.322622568	MRPL14	-1.228692093
TNNT3	1.322608711	CA8	-1.227777961
MIB1	1.322302276	PHC1	-1.226673128
ZBTB47	1.322056944	LOC101116812	-1.226625737
NET1	1.322031407	PDE8B	-1.226086251

TRPM7	1.321776792	GPRASP1	-1.225493034
LCK	1.321714652	DDX28	-1.225280531
SENP7	1.32099391	BLVRA	-1.225126295
IKZF2	1.320810897	PNPLA6	-1.224331143
LOC101122953	1.320527577	PSMB5	-1.224137079
LOC101102001	1.320246156	CDK4	-1.223947279
MN1	1.320238367	LOC114110249	-1.222429565
NMNAT2	1.319610783	NR1H3	-1.222129869
LOC114108840	1.318293846	LOC101117527	-1.222019002
ZC3H6	1.317953515	SERHL2	-1.222014551
PTPRU	1.317948689	GRB14	-1.221687202
LOC114117878	1.317705935	MUS81	-1.221545426
MOSMO_2	1.317381131	SPNS1	-1.220694454
SMURF1	1.317062351	TMEM129	-1.219761651
CPNE1	1.317000883	SFT2D3	-1.218104895
NECTIN4	1.316354527	CFAP410	-1.217912063
EFNA1	1.316328917	SMPD2	-1.217896181
ZNF41	1.31618793	NLE1	-1.21755414
LOC114113014	1.31581189	MIPEP	-1.216758028
CRACR2A	1.315089138	HIP1R	-1.216696032
SCUBE2	1.314900877	KPNA2	-1.216471524
PHACTR4	1.314883624	NCLN	-1.215632609
GPC3	1.314018739	NUBP2	-1.214832861
ACSS2	1.313535499	MRPS16	-1.21448752
KLF3	1.313421049	NEDD9	-1.21377578
ABHD15	1.312566362	RGS2	-1.213684881
LOC105609211	1.312451593	SAE1	-1.213471493
SLC8A1	1.312331358	FREM2	-1.213463143
HERPUD2	1.312090842	PPP3CC	-1.213354757
TOB2	1.311103209	ATP7B	-1.213329156
GLIS3	1.310207863	RAVER1	-1.213135792
COPZ2	1.310075655	MFSD13A	-1.212894651
LOC114111751	1.310054196	NQO2	-1.211782393
CFB	1.309248281	INTS9	-1.211524257
KIAA1147	1.308968658	ACTN2	-1.210675582
KCNA3	1.307705313	LOC114115023	-1.210405771
AFF1	1.307674152	REPIN1	-1.210291641
LOC114117229	1.307513959	ZNF575	-1.210152092
TC2N	1.306813196	HEMK1	-1.209774735
LOC114118362	1.306123913	LOC106990145	-1.209660918
CCNT2	1.305956113	STK25	-1.208554516
PLSCR4	1.30557409	RPUSD1	-1.208521118
RAB8B	1.305537055	RASD1	-1.207469772
LOC101122274	1.303755423	TSSK2	-1.206169143
NTM	1.30296781	LOC114113004	-1.206092476
IQGAP3	1.302933973	DGCR6L	-1.206003438

TNFAIP8L2	1.302007876	GREB1	-1.205909426
RC3H2	1.301076397	SLC37A1	-1.205403787
HID1	1.300812326	HSF1	-1.204969129
GALNT18	1.300086568	HTATIP2	-1.20341376
LOC114113926	1.299956101	POLR2I	-1.203231027
PPP1R3C	1.299622478	GSTCD	-1.202263509
LOC114112164	1.298997904	OSGIN2	-1.202246588
INPP5F	1.298304669	PNKP	-1.201386576
KIAA1217	1.298135387	PSMA7	-1.201238831
LOC105603050	1.297754954	LOC105604472	-1.20054766
VGLL2	1.296993107	GRINA	-1.200220056
CATSPERG	1.296361991	COLGALT2	-1.200123281
OSTM1	1.296276367	INSIG1	-1.199951711
FAM81A	1.295861378	ZNF169	-1.199907933
AGA	1.295721171	CLP1	-1.199897216
PCMTD2	1.295055266	NDUFS4	-1.199892573
IGFALS	1.294479366	TIAM1	-1.199877877
ATL2	1.294320895	LOC101114597	-1.199205354
TXNDC16	1.293541237	NOP58	-1.198408097
GNG2	1.293509737	LOC114112251	-1.198203617
SPTY2D1	1.293229414	UFSP1	-1.198013162
MORN3	1.29311771	LOC101102276	-1.197904418
STXBP6	1.290716676	TRAPPC3L	-1.19771087
CSF2RB	1.290483139	MIGA2	-1.197520904
UVRAG	1.290454491	LOC114117231	-1.197473371
ARMC4	1.290081936	CENPO	-1.19536395
NCOA3	1.289904751	SLC27A2	-1.19477125
ATRNL1	1.289895209	LOC105607745	-1.19477125
PLPP6	1.289497277	LOC101117055	-1.194396828
MBLAC2	1.289225318	LOC114110140	-1.19427677
WWC2	1.28918357	PFAS	-1.193824562
CABP7	1.289182227	CINP	-1.193439325
LOC101120749	1.289182227	NSUN5	-1.192972197
VCPIP1	1.289099681	PPP6R2	-1.192663446
ATP13A3	1.288402204	FBXW9	-1.192606731
MXRA7	1.288025491	LOC101117112	-1.192473248
MTM1	1.287137923	NUBP1	-1.192280581
LOC106990836	1.287137923	DGAT1	-1.192234848
UNC5B	1.287081853	GTPBP6	-1.192185521
ANK3	1.286779246	SMPD4	-1.191868624
LOC105606379	1.286609499	CPSF1	-1.191773536
ARHGEF12	1.286047822	LOC114113967	-1.191362636
ILDR2	1.285919987	LOC114116832	-1.190778245
TM7SF3	1.284858858	SDC4	-1.190367968
KLF6	1.284728555	GEMIN5	-1.189498732
LRRIQ3	1.284704248	CACFD1	-1.189148394

TTC39A	1.283465287	EMC8	-1.189074272
LOC114111231	1.283249918	DKC1	-1.189058988
JMJD1C	1.283033752	NUP93	-1.188976503
SOX13	1.28215604	PRR7	-1.187348676
SEC22C	1.282109581	PACS1	-1.1871694
C1QTNF4	1.282043442	SDHAF1	-1.186146242
SAMD11	1.281986184	PAOX	-1.186002359
AHNAK2	1.281934374	LOC105604385	-1.185844915
TRPC1	1.281905016	SNAPC2	-1.185733598
LOC114108671	1.280958901	E2F7	-1.185219398
WDR97	1.279523775	LOC114110455	-1.184245676
C1QTNF3	1.279141976	CLMP	-1.183269137
LOC101109111	1.27899101	FRMD1	-1.183198597
NCOA2	1.278616324	LOC101118761	-1.183012655
DIPK2B	1.278457041	MPG	-1.182406778
MRTFB	1.27840893	CCDC130	-1.181501177
IQCK	1.278007163	LOC105612199	-1.181283045
ADAT1	1.277925601	LOC114115629	-1.181134971
SLC35D1	1.277726727	LOC105608442	-1.180261541
LOC101117622	1.27739013	EBNA1BP2	-1.179783116
RBMS3	1.27712568	RPP40	-1.179404927
USP32	1.276554491	TIMM8A	-1.179052393
STX7	1.275906175	TIMM29	-1.178383958
LOC101117013	1.275800609	LOC114113985	-1.178257825
LOC114108651	1.275800609	PHPT1	-1.177681199
WDR44	1.27559399	SLC30A3	-1.177488043
KATNAL1	1.274929982	AURKAIP1	-1.176389433
TIPRL	1.273607775	LOC114113988	-1.176230728
CDC14B	1.273379787	LOC105615359	-1.176230728
ACRBP	1.272995641	DNASE1	-1.1759932
FKBP9	1.272009607	NR6A1	-1.175303883
ATRX	1.270562736	ARHGAP33	-1.17524892
FZD1	1.270523271	LOC105603374	-1.174971425
FGR	1.270474675	MCM10	-1.17478449
COL17A1	1.269976644	C12H1orf74	-1.173924514
LOC114117254	1.269665366	MAGOHB	-1.173447455
SNPH	1.268884201	LOC114116198	-1.173433773
LOC114118024	1.26886164	ENKD1	-1.173211624
SNTB2	1.268673537	LOC114113056	-1.17320679
LOC101114579	1.267680857	ZNF628	-1.172328986
LOC114112017	1.267610459	POMGNT1	-1.172267503
FGFR2	1.267588464	LMF1	-1.172029503
ZFHX3	1.267213602	LOC101106288	-1.170685133
TACC2	1.266875285	NUDT2	-1.169617
COG3	1.266141106	EFTUD2	-1.16879148
LOC101104372	1.265877035	SPATA2L	-1.168727258

PRKCE	1.264737318	LOC105611012	-1.167802717
ATXN7	1.264653378	FAM49A	-1.167707887
HAVCR2	1.264250744	LOC101115345	-1.166047684
VASH1	1.263843099	LOC101118849	-1.165916069
LOC114109071	1.26110755	KIAA0895L	-1.165530951
RCN1	1.261067299	DCAKD	-1.165411827
LRIF1	1.260602017	TMEM250	-1.164931952
TRANK1	1.260279104	RPUSD2	-1.164225293
FBXO8	1.260125226	CLASRP	-1.164020787
LOC114110307	1.259806866	DAD1	-1.163571916
GPR153	1.25962157	DOK6	-1.163354051
BOC	1.259500115	HSPA5	-1.162375101
SERINC3	1.259360672	LOC105603395	-1.16203227
LOC106990930	1.259182387	MINDY1	-1.161902389
MTURN	1.258267657	ATXN7L2	-1.160939948
MCUR1	1.25769979	PIDD1	-1.160772383
CIITA	1.257531651	KIAA0895	-1.15881424
TRAPPC6B	1.256583351	LOC114116222	-1.15881424
NEK6	1.256198195	FAM71E1	-1.15881424
RASSF5	1.256097956	SOCS1	-1.158259037
S1PR4	1.255652416	CASP3	-1.158148371
GPR183	1.254657259	FAHD1	-1.157206398
LIN7C	1.253175499	GTF3A	-1.157015337
GSAP	1.251749375	ZNF777	-1.156939742
ETV6	1.251534217	STIP1	-1.156171437
LOC106991500	1.25152498	LOC114114089	-1.155714233
GNA15	1.250838876	LOC114116131	-1.155324972
LOC106990188	1.250838876	LOC101110178	-1.154549309
EMCN	1.250235633	IMPDH1	-1.154262589
ESYT2	1.249969333	TCP11	-1.15425282
TMEM200B	1.249692324	SEC11A	-1.154166934
LNPK	1.248658216	VARS2	-1.153566523
LOC105612390	1.248633684	TIMM10	-1.152750414
SLIT2	1.248194169	B3GAT3	-1.152648998
LRG1	1.247534275	METTL22	-1.152378333
GAL3ST4	1.247382177	LSM6	-1.152106378
LTB	1.246310301	NYAP1	-1.151904755
LOC114114820	1.245706625	LOC106990378	-1.151753515
ANKRD61	1.245706625	LOC114113921	-1.151044437
ZC3H12D	1.245706625	BOP1	-1.149923909
IRS4	1.245706625	DTNBP1	-1.14907637
EPN2	1.245630162	ZNHIT3	-1.148951664
SRPX2	1.245413637	LOC101115554	-1.148624911
SUFU	1.245219676	SLC25A6	-1.1482881
HERC5	1.244694823	FAM72A	-1.14814838
PGGHG	1.243903649	PITX1	-1.148115113

FAM43A	1.242610986	DEAF1	-1.147780243
ITGA8	1.241840948	ACKR1	-1.147718019
LOC101116622	1.241517037	OSBPL10	-1.147026641
EPHA1	1.240237635	RPA3	-1.146253716
TPM1	1.239849658	MMP9	-1.145173522
MOB1B	1.239767403	OGG1	-1.143323898
BCL6	1.239669565	HMGB2	-1.142827372
SYTL4	1.23885014	SPP1_1	-1.142579506
TIMP3	1.238310102	BID	-1.141946143
ITGAL	1.237546425	RBM42	-1.141560326
C7H15orf48	1.237540558	LOC114110148	-1.14103376
ITGB8	1.237540558	PDP2	-1.140471189
LOC105614551	1.23736668	PES1	-1.140461957
HOXA9	1.237208651	MAPKAPK3	-1.138195557
EVI2A	1.236685899	TSHZ2	-1.137126485
TCAF2	1.236173807	RORC	-1.137070581
FZD8	1.235287879	RAB36	-1.136274809
SIRPA	1.234227646	FMC1	-1.136167718
LGALS3	1.233645938	LOC101106227	-1.136106868
RARG	1.233572137	LRRC27	-1.135976687
LOC101114275	1.233564122	PDK1	-1.135519219
DYNLT3	1.233558471	LOC114118052	-1.135105988
ITSN2	1.233273228	ASB9	-1.134996814
LOC105603910	1.233111636	PATZ1	-1.134543438
MYOF	1.232324624	LOC105601981	-1.13428853
LOC114117545	1.231560285	LOC114118727	-1.134214144
SNX7	1.231003514	ENC1	-1.133982029
TRIM16	1.229251736	TXNRD3	-1.133503977
BNIP2	1.229171606	LOC114112968	-1.133300732
PPIP5K1	1.228960653	MORN4	-1.132748721
KPNA5	1.228232158	RPS9	-1.132732554
C25H10orf71	1.227923874	PDE6C	-1.132707817
PHTF2	1.227668699	LOC105603538	-1.132707817
UHRF1BP1L	1.227094631	PELP1	-1.132680814
LOC105602268	1.226358081	ANKRD13B	-1.13175155
UPB1	1.226264768	PGK1	-1.131054155
CMKLR1	1.225486276	CDO1	-1.130980063
OTULINL	1.225426538	RPL18	-1.130704286
FGF11	1.225383831	CHPF	-1.130136003
BCL2L15	1.225121449	MRTO4	-1.130130452
INSC	1.225121449	LOC105607169	-1.129677882
PRXL2C	1.225030215	MKX	-1.129366748
CPQ	1.224941056	DYNLRB2	-1.129210955
SAMHD1	1.223680278	LIG3	-1.128747297
CSF1	1.223675659	TMEM223	-1.12872646
LINGO1	1.223458958	LOC101118433	-1.12829599

TRAF3IP1	1.223226437	LOC443015	-1.12799202
ATXN3	1.223223103	CCT7	-1.127991998
GNB5	1.223163537	SPPL2B	-1.127655451
ANKRD29	1.223113823	HMCES	-1.126304987
DCAF17	1.222837034	LOC101110022	-1.126080329
MIER1	1.222603399	ASTN2	-1.125793592
FAM214B	1.222391136	NCAPG	-1.125284304
LOC114114509	1.221791546	NUP85	-1.125149224
LCA5	1.22161547	ALOX12	-1.124696644
LOC114116816	1.221526137	FARSB	-1.124352597
CTDSPL	1.221493909	FANCA	-1.124211314
APBB1IP	1.221342536	PRDX5	-1.124101263
ZBTB8A	1.220527467	LOC101114018	-1.124028672
ENPP2	1.220445209	BEX2	-1.122795652
LMO3	1.22032099	CTPS1	-1.122091337
PLS3	1.220220331	LOC101106975	-1.121349536
SUSD1	1.220195496	HOXC8	-1.121125128
CACNA1S	1.220195496	CNPY3	-1.120047842
PIK3R3	1.219929139	ZNF48	-1.119982109
TIGD2	1.219926055	PSMD4	-1.119501304
C1QA	1.218403982	MYH7B	-1.119278857
LOC105604727	1.217848878	PIMREG	-1.118369015
MAGEE2	1.216476049	PLEKHA8	-1.1183684
TP53I11	1.215819253	ATG101	-1.118165272
ULK2	1.215753114	PVR	-1.117799361
PRSS23	1.214267198	LEMD2	-1.117762699
TCF4	1.213454363	BTBD2	-1.117586816
LOC101112480	1.213127883	PYM1	-1.116687372
CCN1	1.21271759	SH2D3A	-1.116526552
MPEG1	1.211587375	CCDC106	-1.116286082
LOC101105651	1.211192671	DOCK5	-1.116013616
SAMSN1	1.211187949	VGF	-1.114632985
SERP2	1.211077728	AMDHD2	-1.114316842
WSCD1	1.210984667	PRELID3A	-1.113972089
COL4A3	1.210510091	PSMB8	-1.113788342
HEYL	1.210402965	LOC105608522	-1.112563455
PKD2	1.210042847	SLC8B1	-1.112373936
LOC101106743	1.209388218	IMPDH2	-1.112080412
ADAM10	1.207815699	NOP9	-1.111829891
XRN1	1.207617713	DCTD	-1.111829891
CCP110	1.207274224	TIMM50	-1.111498468
TTC28	1.206432903	LOC105604728	-1.111452813
KCP	1.205823862	TMEM69	-1.111056123
LOC101122123	1.205795226	AARS2	-1.110592502
IKBIP	1.205514843	SDF2L1	-1.110199009
DLL4	1.204535008	MESD	-1.110193684

LOC114113868	1.203924375	PFDN2	-1.110024341
LOC106990331	1.203304496	NDUFS8	-1.109995747
TUT7	1.202370573	KRI1	-1.109711471
ACVRL1	1.201985418	PRDX4	-1.108906073
LOC114114813	1.201724716	SEMA4B	-1.108855913
LOC114117333	1.201724716	PGR	-1.108855913
CD96	1.201375641	NDUFS7	-1.108544703
ADM5	1.200814261	POLL	-1.108125727
LOC114110248	1.200734651	LSM3	-1.107551101
BRMS1L	1.200062248	GLI4	-1.106581689
PIK3CG	1.199858083	ACSF3	-1.106432232
DAGLA	1.199508452	DUS1L	-1.106302
CNR1	1.199458257	TSPYL5	-1.105952029
TIE1	1.199304128	LOC105606441	-1.105486364
DUSP2	1.198791797	KIF18A	-1.104597334
RHOD	1.198642762	DAP3	-1.104158582
ING3	1.198215692	HAX1	-1.103905664
CEP290	1.19811369	PARD6B	-1.10348469
LOC105613342	1.198081524	RPL29	-1.103014344
LOC114116910	1.198015849	RAVER2	-1.102967177
SMCO3	1.197897291	SERAC1	-1.102852999
GLDN	1.197828392	TSACC	-1.102685053
NME9	1.197004239	ERAL1	-1.102476351
SLC9A9	1.196032439	PTPMT1	-1.101860914
ADAMTS9	1.196022356	LPIN2	-1.101730763
ATAD2B	1.195971127	SPON2	-1.101304425
RGS1	1.195222487	LOC114112987	-1.100571957
FRMD4B	1.194966635	LDHA	-1.100507972
TANK	1.194039481	PPP2R1A	-1.100438671
FAM210B	1.193668961	VRK1	-1.100403603
LOC114118082	1.193257908	CDCA2	-1.100319227
FGD5	1.192936773	GLYCTK	-1.100136562
SH3BGRL2	1.192103355		
ALG2	1.191593039		
GRAMD1B	1.191488839		
STOM	1.190220762		
EHD4	1.190101438		
B4GALNT3	1.189874066		
GRAMD2A	1.189874066		
FAM13C	1.189606304		
SMAD9	1.189469369		
RASAL3	1.189026352		
PTEN	1.188620128		
QKI	1.188321488		
N4BP2	1.18826567		
KIAA1107	1.188171086		

LMNTD1	1.187720444
LOC114108673	1.187649232
TMEM158	1.187071649
LOC101116298	1.187061813
UBE2J1	1.186868492
CLEC11A	1.18634861
ZBTB11	1.185830228
OAT	1.18518167
SCNN1B	1.184918288
RILP	1.184576941
EPS8	1.184330831
ITGAX	1.184208583
CACNA1D	1.18361164
C5H19orf71	1.183567956
STX2	1.183358521
AS3MT	1.183043097
ITGA11	1.182370064
ARL15	1.181555937
PKHD1L1	1.181254825
FSIP1	1.179825746
ENAH	1.179731702
CRTAP	1.179180661
ARSI	1.178952555
LOC114109611	1.178564806
MARF1	1.178346133
LOC114117965	1.177942285
AGTR2	1.177942285
C1H21orf91	1.177422093
CREBRF	1.177417224
TCAF1	1.177396378
LOC114110567	1.177315887
ITGAM	1.176489484
LOC101112291	1.176371092
LOC101110545	1.175915465
INPP5D	1.175645902
TACSTD2	1.175597151
SEMA4A	1.174572907
PLEKHG5	1.174473647
RNF38	1.174028184
ZNHIT6	1.173264557
MTPN	1.173058436
LOC106990829	1.17281855
LOC101105810	1.172734451
RAB2B	1.172362704
LOC114113811	1.1722396
KLF4	1.17202596

FAS	1.17185221
LOC101117485	1.17185221
CMYA5	1.171312286
CTIF	1.170915488
MYL6	1.170610192
LOC114109543	1.170596216
LOC114111048	1.169461998
LOC105611671	1.168687926
DNAJC3	1.168474935
BTBD3	1.168051024
COL6A1	1.167858587
RSBN1L	1.167258573
LOC101111832	1.166888132
ADAM9	1.166574524
BBS5	1.166473698
UNC80	1.166392001
LOC105610540	1.166392001
FBLN1	1.166046838
SPSB4	1.165839966
MEF2D	1.16548283
FAM214A	1.165080164
VIPR2	1.164934797
CDCP1	1.164700703
RTN4	1.164469055
KLF12	1.164434229
HOXA1	1.16425734
CBFA2T3	1.163752285
NRARP	1.163416807
CLCN5	1.163307716
EYA1	1.163110046
TMEM154	1.161704933
ZC3H11A	1.160511747
KITLG	1.159693031
LOC101103233	1.158843389
LOC114117238	1.158711979
KRAS	1.157822036
WSB1	1.157469353
PCDH10	1.157378986
ARID4B	1.15591328
GFOD1	1.155589601
IL34	1.155064361
PAK1	1.154399138
PTPN12	1.154398693
LOC114116374	1.154284543
CBL	1.154018439
TREX2	1.153761227

LOC114114429	1.153293752
CD93	1.153022431
PRKCB	1.153003412
CNOT6L	1.152949768
CD9	1.152596059
TMEM182	1.152545959
SERTAD2	1.152470582
ZNF774	1.152384845
LRCH3	1.151685563
PJA2	1.150561962
SH3BP5	1.150483284
ANKH	1.150370566
PARVA	1.150241163
LYSMD2	1.149765109
TNFRSF25	1.149420894
HELZ	1.148996668
RABEP1	1.148848232
PAMR1	1.148707215
TMTC2	1.148548784
C2	1.148456219
GPR157	1.147572869
ZSCAN23	1.147572869
LOC101112936	1.147270539
DZIP3	1.14713931
TSC22D1	1.146919266
FERMT3	1.146880971
SNCAIP	1.146718484
SKP1_2	1.146384376
PCF11	1.145739667
STRN3	1.145565335
MTSS2	1.14529519
AHCYL2	1.144953358
ASB5	1.144399989
GEM	1.144190352
DENND6A	1.143683095
GGH	1.143598018
LMOD1	1.143014559
CLCF1	1.142727385
PDLIM1	1.142482266
LOC114115283	1.142470223
ZNF114	1.141117586
C24H16orf72	1.141112415
LOC101104745	1.140942287
LOC101117364	1.140942287
LOC105604630	1.140579817
DNAJC12	1.140480483

RNF217	1.140280336
ACSL4	1.13998541
LOC101118793	1.139485807
PLD1	1.138816344
ETS1	1.138811119
CORO1C	1.138808826
SPTBN5	1.138478682
LOC101120322	1.138244688
ISLR	1.137401326
KCNAB1	1.137192798
LOC105604882	1.137153095
SERTAD4	1.136478621
AGO3	1.136425003
SEPT8	1.135124741
ARHGAP4	1.135103712
C11H17orf107	1.134722978
SLAMF8	1.134388916
NEXMIF	1.134388916
GAS6	1.134355717
DHRS3	1.1324155
SDC3	1.131030569
PHF20L1	1.130858756
TGFB1	1.129765772
CDNF	1.129417456
NLRP3	1.12916795
RNASE13	1.12916795
SLC16A14	1.128983655
PDE4DIP	1.128193035
USP53	1.127731952
NOD1	1.127490407
DLG1	1.127064375
ZNF132	1.12605873
CARNMT1	1.125918549
TGFBR1	1.125905573
SLC30A1	1.125593552
GRAMD2B	1.125322896
ST3GAL2	1.125057077
SLC25A16	1.124894808
ZNF25	1.124690157
RHPN2	1.123997361
CALCOCO1	1.123965028
CDC42BPG	1.123780321
RPS6KL1	1.123159589
SOCS4	1.123012465
MYO1C	1.12238106
COL13A1	1.122138711
CLASP2	1.12164255
MAPK4	1.121301486

CD99L2	1.121301055
CALML4	1.121017563
MCU	1.120873245
MCL1	1.120574288
TRMT13	1.119894885
SLC35A5	1.119672708
TET3	1.119272998
GON7	1.119087279
INTS6	1.118921114
RAB22A	1.118666908
LAPTM5	1.118416757
