# Supplementary material for: Ovarian activation delays in peripubertal ewe lambs infected with Haemonchus contortus can be avoided by supplementing protein in their diets
Source: BMC Vet Res. 2021 Nov 3;17:344. doi: 10.1186/s12917-021-03020-7 (PMC8565066; doi:10.1186/s12917-021-03020-7)
Supplement: Supplementary file 7 — Additional file 7: Figure S1. Enriched terms in up-regulated genes between Supplemented Not Infected vs Control Not Infected. [file 12917_2021_3020_MOESM7_ESM.pdf]

Ovarian activation delays in peripubertal ewe lambs infected with *Haemonchus contortus* can be avoided by supplementing protein in their diets

Paula Suarez-Henriques, Camila de Miranda e Silva-Chaves, Ricardo Cardoso-Leite, Danielle G. Gomes-Caldas, Luciana Morita-Katiki, Siu Mui Tsai, Helder Louvandini

Additional file 7.

Figure 1. Enriched terms in up-regulated genes between Supplemented Not Infected vs Control Not Infected

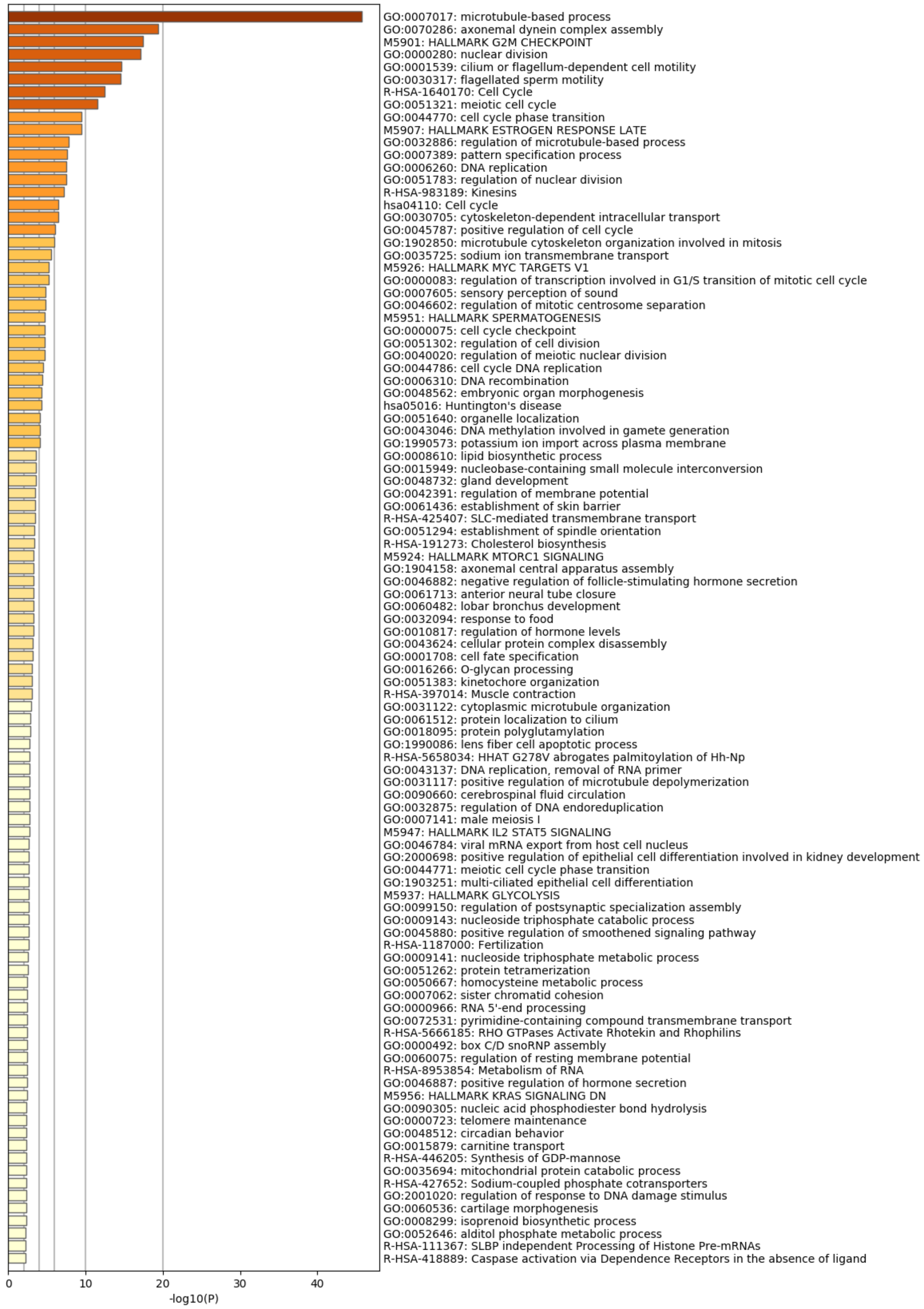

***The hypergeometric accumulated p-values and enrichment factors were calculated and used for filtering. The significant terms that remained after filtering were hierarchically grouped in a tree based on Statistical similarity Kappa. The score Kappa 0.3 was applied as threshold to fuse the tree in terms grouping.**
