# Supplementary material for: Ovarian activation delays in peripubertal ewe lambs infected with Haemonchus contortus can be avoided by supplementing protein in their diets
Source: BMC Vet Res. 2021 Nov 3;17:344. doi: 10.1186/s12917-021-03020-7 (PMC8565066; doi:10.1186/s12917-021-03020-7)

Ovarian activation delays in peripubertal ewe lambs infected with *Haemonchus contortus* can be avoided by supplementing protein in their diets

Paula Suarez-Henriques, Camila de Miranda e Silva-Chaves, Ricardo Cardoso-Leite, Danielle G. Gomes-Caldas, Luciana Morita-Katiki, Siu Mui Tsai, Helder Louvandini

Additional file 9.

Enriched terms subset in up-regulated genes between Control Not Infected vs Supplemented Not Infected

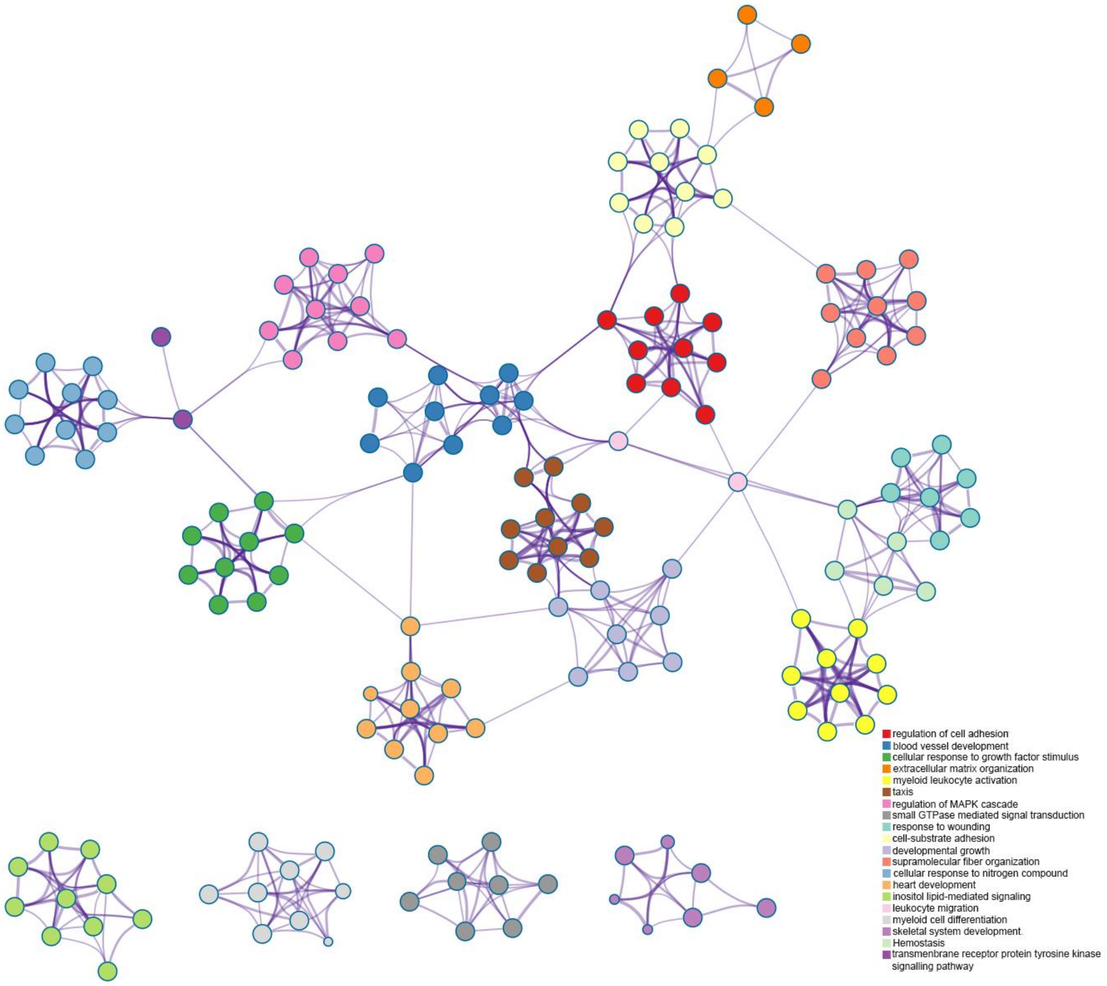

Supplement: Supplementary file 9 — Additional file 9. Enriched terms subset in up-regulated genes between Control Not Infected vs Supplemented Not Infected. [file 12917_2021_3020_MOESM9_ESM.pdf]
