# Supplementary material for: Ovarian activation delays in peripubertal ewe lambs infected with Haemonchus contortus can be avoided by supplementing protein in their diets
Source: BMC Vet Res. 2021 Nov 3;17:344. doi: 10.1186/s12917-021-03020-7 (PMC8565066; doi:10.1186/s12917-021-03020-7)

Ovarian activation delays in peripubertal ewe lambs infected with *Haemonchus contortus* can be avoided by supplementing protein in their diets

Paula Suarez-Henriques, Camila de Miranda e Silva-Chaves, Ricardo Cardoso-Leite, Danielle G. Gomes-Caldas, Luciana Morita-Katiki, Siu Mui Tsai, Helder Louvandini

Additional file 11. Enriched terms in the list of differentially up-regulated genes between the groups Control Infected vs Supplemented Infected.

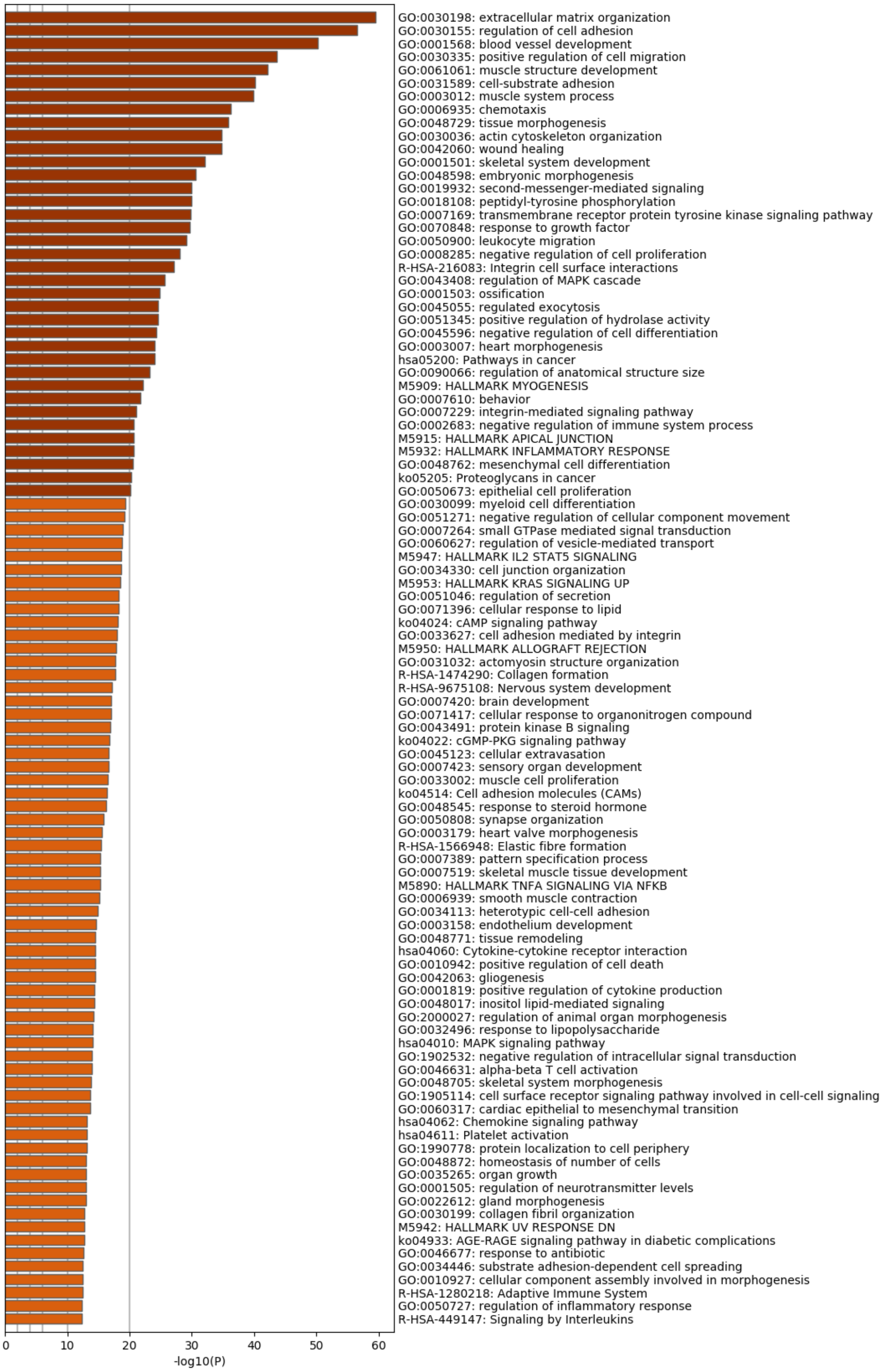

Supplement: Supplementary file 11 — Additional file 11. Enriched terms in the list of differentially up-regulated genes between the groups Control Infected vs Supplemented Infected. [file 12917_2021_3020_MOESM11_ESM.pdf]
