# Supplementary material for: Ovarian activation delays in peripubertal ewe lambs infected with Haemonchus contortus can be avoided by supplementing protein in their diets
Source: BMC Vet Res. 2021 Nov 3;17:344. doi: 10.1186/s12917-021-03020-7 (PMC8565066; doi:10.1186/s12917-021-03020-7)
Supplement: Supplementary file 12 — Additional file 12. Average liveweight and age (± standard deviation) for each group at the beginning and end of the experiment. [file 12917_2021_3020_MOESM12_ESM.pdf]

Ovarian activation delays in peripubertal ewe lambs infected with *Haemonchus contortus* can be avoided by supplementing protein in their diets

Paula Suarez-Henriques, Camila de Miranda e Silva-Chaves, Ricardo Cardoso-Leite, Danielle G. Gomes-Caldas, Luciana Morita-Katiki, Siu Mui Tsai, Helder Louvandini

Additional file 12

Average liveweight and age (± standard deviation) for each group at the beginning and end of the experiment

	Control protein Not Infected	Control protein Infected	Supplemented protein Not infected	Supplemented protein Infected
**Group's mean weigth (kg)at the beginning of the experiment**	22.2 (±5.5)	23.5 (±3.9)	21.5 (±7.2)	22.1 (±5.6)
**Group's mean weight (kg) at the end of the experiment**	29.5 (±4.2)	31.7 (±4.4)	28.1 (±11.4)	29.1 (±6.4)
**Group's mean age (days) at the start of experiment**	220 (±5.4)	217 (±9.6)	215 (± 5.9)	208 (±7.7)
**Group's mean age (days)at the end of the experiment**	299 (±5.4)	296 (±9.6)	294 (±5.9)	287 (±7.7)
