# Supplementary material for: Ovarian activation delays in peripubertal ewe lambs infected with Haemonchus contortus can be avoided by supplementing protein in their diets
Source: BMC Vet Res. 2021 Nov 3;17:344. doi: 10.1186/s12917-021-03020-7 (PMC8565066; doi:10.1186/s12917-021-03020-7)
Supplement: Supplementary file 13 — Additional file 13. Composition of the control protein and supplemented protein diets. [file 12917_2021_3020_MOESM13_ESM.pdf]

Ovarian activation delays in peripubertal ewe lambs infected with *Haemonchus contortus* can be avoided by supplementing protein in their diets

Paula Suarez-Henriques, Camila de Miranda e Silva-Chaves, Ricardo Cardoso-Leite, Danielle G. Gomes-Caldas, Luciana Morita-Katiki, Siu Mui Tsai, Helder Louvandini

Additional file 13

Composition of the control protein and supplemented protein diets

	Control protein diet	Supplemented protein diet
Forage feed		
Tifton-85 hay	60%	60%
Concentrated feed		
Ground corn	32%	16%
Soybean meal	8%	24%
Crude protein (% total diet)	12%	19%
