# Supplementary material for: Ovarian activation delays in peripubertal ewe lambs infected with Haemonchus contortus can be avoided by supplementing protein in their diets
Source: BMC Vet Res. 2021 Nov 3;17:344. doi: 10.1186/s12917-021-03020-7 (PMC8565066; doi:10.1186/s12917-021-03020-7)
Supplement: Supplementary file 14 — Additional file 14. Bromatological composition (g kg-1 of dry matter at 100 °C) and energy (MJ) of the diets’ ingredients. [file 12917_2021_3020_MOESM14_ESM.pdf]

Ovarian activation delays in peripubertal ewe lambs infected with *Haemonchus contortus* can be avoided by supplementing protein in their diets

Paula Suarez-Henriques, Camila de Miranda e Silva-Chaves, Ricardo Cardoso-Leite, Danielle G. Gomes-Caldas, Luciana Morita-Katiki, Siu Mui Tsai, Helder Louvandini

Additional file 14.

Bromatological composition (g kg^{-1} of dry matter at 100°C) and energy (MJ) of the diets' ingredients

	**Concentrated feed Supplemented Protein**	**Concentrated feed Control protein**	**Tifton-85 Hay**
**Dry matter at 105°C**	897.11	896.50	877.90
**Crude Protein**	317.69	153.04	104.20
**Energy**	0.018	0.018	0.019
**Fiber in neutral detergent**	246.26	168.34	727.90
**Fiber in acid detergent**	188.75	131.89	386.80
**Ethereal extract**	14.88	23.19	20.70
**Mineral matter**	99.60	84.63	66.87
**Lignin**	26.93	25.75	
**Phosphorus**	10.21	8.39	
