# Supplementary material for: Ovarian activation delays in peripubertal ewe lambs infected with Haemonchus contortus can be avoided by supplementing protein in their diets
Source: BMC Vet Res. 2021 Nov 3;17:344. doi: 10.1186/s12917-021-03020-7 (PMC8565066; doi:10.1186/s12917-021-03020-7)
Supplement: Supplementary file 15 — Additional file 15. Bromatological analysis of the diet. [file 12917_2021_3020_MOESM15_ESM.pdf]

Ovarian activation delays in peripubertal ewe lambs infected with helminths can be avoided by supplementing protein in their diets

Paula Suarez-Henriques, Camila de Miranda e Silva-Chaves, Ricardo Cardoso-Leite, Danielle G. Gomes-Caldas, Luciana Morita-Katiki, Siu Mui Tsai, Helder Louvandini

Additional file 15.

Bromatological analysis of the diet

We calculated dry matter in the diet after drying it out in an electric kiln (Odontobrás 1.6, Brazil) at 105°C for 24 hours. The mineral matter was calculated after burning the diet sample out in a muffle furnace (JUNG, Brazil) at 550°C for five hours and 30 minutes. Crude protein's amount was established with a micro-Kjeldahl method of vapour distillation (Tecnal, Brazil) using 15 ml of sodium hydroxide with volatilised nitrogen (NaOH 12N) collected in the boric acid solution at 2% (H3BO3) and quantified by titration with a sulfuric acid solution (H2SO4 0.005N) with multiplication by conversion factor 6,25. The ethereal extract was quantified with petroleum ether by heating it in a fat extractor (Soxhlet- TE-044-8/50 – Tecnal, Brazil). The quantification of fibre in neutral detergent was obtained after washing the diet samples in fibre quantification bags Ankom F57 (Ankom Technology Corp., USA) at 90°C for 1 hour in neutral detergent solution [1] . Then, alpha-amylase and sodium sulfite were added (TE-149 – Tecnal, Brazil), followed by two washes with distilled water plus alpha-amylase at 90°C for five minutes and another acetone wash for five minutes. These bags were put in the electric kiln at 105°C (Odontobrás, Brazil) for four hours and then in a dryer. They were weighed in an analytical balance (UY220, Japan) and transferred to porcelain crucibles incinerated in a muffle furnace (JUNG, Brasil) at 530°C for 3 hours and 30 minutes. The fibre's neutral detergent calculation (FDN) was performed after correction for ashes. The fibre in acid detergent (FDA) was estimated after the diet samples were washed with an acid detergent solution at 90°C for 60 minutes, followed by four rinses in distilled water at 90°C for five minutes and another five minutes wash with acetone. The bags with diet samples were put in an electric kiln at 105°C for four hours, then in a dryer until reaching 25 °C for their weighing. The lignin was predicted using the FDA bags; both were calculated after being corrected for ashes. After the bags were weighted, were put in a sulfuric acid solution at 72% for three hours. They were rinsed with distilled water at 90°C for five minutes and washed with acetone for another five minutes. Bags with diet were put

in an electric kiln at 105°C for four hours, then were weighed and put in porcelain crucibles to burn out in a muffle furnace at 530°C for three hours and 30 minutes. After the combustion, the quantification of energy in the diet ingredients was done in the calorimeter bomb 6200(Parr Instrument Company – USA).

References

1. Van Soest PJ, Robertson JB, Lewis BA. Methods for Dietary Fiber, Neutral Detergent Fiber, and Nonstarch Polysaccharides in Relation to Animal Nutrition. J Dairy Sci. 1991;74:3583–97. doi:10.3168/jds.S0022-0302(91)78551-2.
