# Supplementary material for: Ovarian activation delays in peripubertal ewe lambs infected with Haemonchus contortus can be avoided by supplementing protein in their diets
Source: BMC Vet Res. 2021 Nov 3;17:344. doi: 10.1186/s12917-021-03020-7 (PMC8565066; doi:10.1186/s12917-021-03020-7)
Supplement: Supplementary file 16 — Additional file 16. List of ovarian RNA samples, their concentration, 260/280 and 260/230 absorbance ratios. [file 12917_2021_3020_MOESM16_ESM.pdf]

Ovarian activation delays in peripubertal ewe lambs infected with *Haemonchus contortus* can be avoided by supplementing protein in their diets

Paula Suarez-Henriques, Camila de Miranda e Silva-Chaves, Ricardo Cardoso-Leite,

Danielle G. Gomes-Caldas, Luciana Morita-Katiki, Siu Mui Tsai, Helder Louvandini

Additional file 16

List of ovarian RNA samples, their concentration, 260/280 and 260/230 absorbance ratios

Sample/ Animal ID	RNA concentration (ng/μL)	Ratio 260/280	Ratio 260/230
B3	532	1.92	2
B4	275	1.9	2.11
B8	84	1.85	1.99
A9	405	1.95	1.85
A18	193	1.9	1.84
A19	224	1.88	2.12
A20	812	1.95	2
B21	245	1.87	2.21
B23	176	1.91	1.78
B24	287	1.88	1.97
A28	564	1.94	2.17
A32	360	1.88	1.66
A33	486	1.88	1.66
A35	485	1.86	1.9
A40	176	1.83	2.03
C22	317	1.92	1.84
A34	57	1.71	1.5
