# Supplementary material for: Ovarian activation delays in peripubertal ewe lambs infected with Haemonchus contortus can be avoided by supplementing protein in their diets
Source: BMC Vet Res. 2021 Nov 3;17:344. doi: 10.1186/s12917-021-03020-7 (PMC8565066; doi:10.1186/s12917-021-03020-7)
Supplement: Supplementary file 17 — Additional file 17. EM estimation algorithm procedure. [file 12917_2021_3020_MOESM17_ESM.pdf]

Ovarian activation delays in peripubertal ewe lambs infected with *Haemonchus contortus* can be avoided by supplementing protein in their diets

Paula Suarez-Henriques, Camila de Miranda e Silva-Chaves, Ricardo Cardoso-Leite, Danielle G. Gomes-Caldas, Luciana Morita-Katiki, Siu Mui Tsai, Helder Louvandini

Additional file 17 - EM estimation algorithm procedure

The EM algorithm was inspired by the RSEM [1] and eXpress (https://pachterlab.github.io/eXpress/manual.html) methods. It successively estimates the abundance of genes/transcripts according to these abundances. For a gene with two transcripts, where one of the transcripts is twice longer than the other turns the longer transcript twice more abundant, which means that two of these three reads originate from the longer transcript. The final read comes from the shorter transcript in the exon common to both transcripts. The longer transcript has a second exon which also generates two reads. So, the longer transcript is twice more abundant, and because it is twice longer, it generates four times the number of reads. If the shorter transcript were twice more abundant than the longer transcript, there would be equal numbers of reads of each transcript.

A mapping is a set of transcripts to which one read can map. A maximization expectancy procedure estimated the transcript abundance. In the cases mentioned on the previous paragraph, some reads had the mapping a1= ft1; t2g (these are reads mapped in a y non-unique way), and some reads had the mapping a2 = ft2g (these are reads mapped uniquely). On both examples, the mapping counting a1 is three because three reads are shared between the transcripts. The mapping counting a2 is two in the first example and one in the second example.

1.When the process starts, the transcripts abundances are evenly distributed under the assumption that all the transcripts are equally expressed.

2. Expectancy step: the transcript assumed abundances were used to calculate each transcript's expected counting, meaning the expected number of reads that should be assigned to a given transcript. This process was done passing over all the mappings that include a given transcript and assigning a proportion of the total counting of that mapping to the transcript. The proportion corresponds to the proportion of total abundance of transcripts in the mapping due to the target.

3. Maximisation step: the counting assigned to each transcript were used to re-compute the transcript abundance. This step was done passing over all the targets. Each target divides the proportion of counting currently assigned to the transcript (=total counting per transcript divided by the total number of reads) by the target's length. It is expected that longer transcripts proportionately generate more reads.

4. Repetition of step 2 until convergence: once the algorithm converged, each mapped read in a non-unique way is randomly assigned to a transcript according to the same mapping's transcript abundances. The column of total reads of transcripts shows these assignments. The values of RPKM and TPM are assigned to each transcript.

References

1. Li B, Dewey CN. RSEM: Accurate transcript quantification from RNA-Seq data with or without a reference genome. BMC Bioinformatics. 2011;12:323. doi:10.1186/1471-2105-12-323.
