# Supplementary material for: Ovarian activation delays in peripubertal ewe lambs infected with Haemonchus contortus can be avoided by supplementing protein in their diets
Source: BMC Vet Res. 2021 Nov 3;17:344. doi: 10.1186/s12917-021-03020-7 (PMC8565066; doi:10.1186/s12917-021-03020-7)
Supplement: Supplementary file 18 — Additional file 18. Statistical methodology used in the Enrichment analysis. [file 12917_2021_3020_MOESM18_ESM.pdf]

Ovarian activation delays in peripubertal ewe lambs infected with *Haemonchus contortus* can be avoided by supplementing protein in their diets

Paula Suarez-Henriques, Camila de Miranda e Silva-Chaves, Ricardo Cardoso-Leite, Danielle G. Gomes-Caldas, Luciana Morita-Katiki, Siu Mui Tsai, Helder Louvandini

Additional file 18.

Statistical methodology used in the Enrichment analysis

Considering *N* as the total number of genes (the whole genome as background), the number of genes in a pathway as *k* and, our input gene list containing *M* genes, among which *n* genes are found in the same *k* given pathway.

The enrichment factor shows how many folds more individual pathway members were found in our input gene list in comparison to what would have been anticipated randomly:

Enrichment Factor: *nMkN* = *nNkM*

If our *M* input genes were selected by chance out of the pool of *N* genes, the probability that we obtained *n* genes from the given *k* pathway would be:

(kn)(N-kM-n)

This expression above is known as hypergeometric distribution. The p-value(log10p) was defined as the probability to obtain *n* or more pathway members, creating an accumulative hypergeometric distribution:

p = Σi = *nmin*(*M,K*)(in)(*N-kM-i*)

A more negative p-value indicated that the observed enrichment was less due to randomness (Zar, 1988). The software used the BH-adjustment (Benjamini and Hochberg, 1995) to address the statistical problem of multiple testing, the Q number of pathways and finding significative p-values only due to the large number of the pathways queried against. For the BH-adjustment to be calculated, all p-values were firstly classified from small to large. If a p-value ranked i, it would

be expected that pQ pathways would be found with an equal or better p-value at random under the Bonferroni correction. Because only i pathways were observed, part of our observations would be false (false discovery rate - FDR).

$$\min(pQi, 1)$$

The z – score is the Z- standard deviation away from the anticipated counts(Kim and Volsky, 2005). Although it is just an approximate form, its formula is as follows:

$$Z = (n-MkN)\sigma$$
