# Supplementary material for: Ovarian activation delays in peripubertal ewe lambs infected with Haemonchus contortus can be avoided by supplementing protein in their diets
Source: BMC Vet Res. 2021 Nov 3;17:344. doi: 10.1186/s12917-021-03020-7 (PMC8565066; doi:10.1186/s12917-021-03020-7)
Supplement: Supplementary file 19 — Additional file 19. Protein Network analysis. [file 12917_2021_3020_MOESM19_ESM.pdf]

Ovarian activation delays in peripubertal ewe lambs infected with *Haemonchus contortus* can be avoided by supplementing protein in their diets

Paula Suarez-Henriques, Camila de Miranda e Silva-Chaves, Ricardo Cardoso-Leite, Danielle G. Gomes-Caldas, Luciana Morita-Katiki, Siu Mui Tsai, Helder Louvandini

Additional file 19.

Protein Network analysis

Because analysing gene lists in the context of proteins interactions may help to enlighten complex biochemical processes or components of signals' translation that rule biological processes, we used an algorithm of complex identification built in Metascape. The MCODE algorithm infers results that are easier to interpret in a biological context. It extracts protein complexes that are built-in the more extensive network. A functional enrichment is also performed to combine the three ontological more significant terms to annotate the possible biological roles for each MCODE complex.

The protein pathways provide a complementary way to identify dynamically, the groups of protein that are probably functionally relevant. The connected proteins inside a PPI (protein-protein interaction) network are probable collaborators, forming a translation signal of a pathway or molecular complex that execute related biological processes. The genes that do not interact with other hits are filtered to generate a sub-list with a better ratio between the signal to noise.The processes and pathways described in the enrichment analysis were automatically applied to the proteins network, and the more enriched terms characterise their biological significance.

The algorithm Molecular Complex Detection (MCODE) was applied to identify regions with pathways densely connected. Each component of the region was more probably associated with a functional unity or a complex than the rest of the network. The enrichment analysis of the pathways/biological processes was automatically applied to the proteins in the network to understand the role of each of the identified MCODE components (minimum size of three proteins), and the more enriched terms characterised their biological significance.

Only the up-regulated gene list generated by the comparison Supplemented not Infected vs Control not Infected originated a protein network. The three connected proteins are part of the Hedgehog (or Smoothened) signalling pathway (Figure 9).

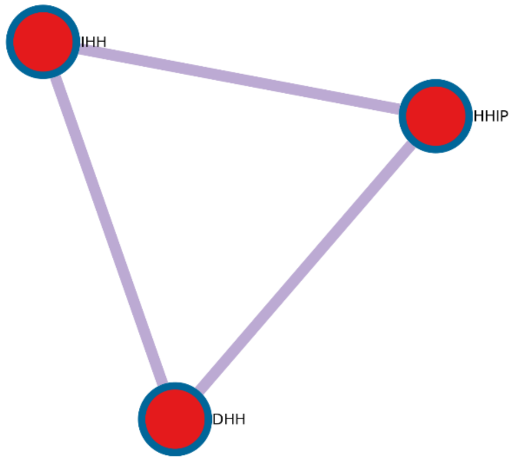

Figure 9. Protein network generated in the up-regulated gene list of the comparison Supplemented not Infected vs Control not Infected. HHIP= Hedgehog interacting protein, IHH= Indian Hedgehog protein, DHH= Desert Hedgehog protein.
